# Upstream Interventions in Clinical Settings Focused on Nutrition to Prevent Obesity During the First 1000 Days: A Scoping Review

**DOI:** 10.1016/j.advnut.2025.100529

**Published:** 2025-10-09

**Authors:** Courtney T Luecking, Chelsea L Kracht, Mackenzie J Ferrante, Kameron J Moding, Elizabeth Kielb, Laura J Rolke, Brooke E Wagner, Jordan Colella, Katherine E Speirs, Cayla Robinson, Cody D Neshteruk

**Affiliations:** 1 Department of Dietetics and Human Nutrition, Martin-Gatton College of Agriculture, Food and Environment, University of Kentucky, Lexington, KY, United States; 2 Pennington Biomedical Research Center, Baton Rouge, LA, United States; 3 Division of Physical Activity and Weight Management, Department of Internal Medicine, University of Kansas Medical Center, Kansas City, KS, United States; 4 Department of Pediatrics, Jacobs School of Medicine and Biomedical Sciences, University at Buffalo, Buffalo, NY, United States; 5 Department of Nutritional Sciences, School of Environmental and Biological Sciences, Rutgers University, New Brunswick, NJ, United States; 6 Department of Human Development and Family Science, Purdue University, West Lafayette, IN, United States; 7 Department of Population Health Sciences, Duke University School of Medicine, Durham, NC, United States; 8 Duke Center for Childhood Obesity Research, Duke University School of Medicine, Durham, NC, United States; 9 Human Development and Family Science, John and Doris Norton School of Human Ecology, University of Arizona, Tucson, AZ, United States; 10 Libraries, University of Kentucky, Lexington, KY, United States

**Keywords:** maternal and child health, public health, healthcare, infant, toddler, lactation, complementary feeding, food insecurity, nutrition security

## Abstract

Nutritional exposures during pregnancy and the first 2 y of a child’s life influence growth and risk for obesity. Upstream interventions that involve policy, systems, and/or environmental approaches may support equitable nutrition and healthy growth early in life. Yet little is known about the application of these interventions in clinical settings. This scoping review characterized the breadth, generalizability, and methodological rigor of nutrition-focused, upstream interventions for obesity prevention during the first 1000 d in clinical settings. Eight databases were searched in November 2022 for policy, systems, and/or environmental approaches conducted during the first 1000 d. Titles, abstracts, and full texts were independently screened in duplicate, with conflicts resolved by a third reviewer. Extracted elements included study characteristics, reach, effectiveness, adoption, implementation, maintenance, and the Downs and Black study quality checklist. Of the 73,969 records identified, 185 reports representing 126 studies were included. Studies frequently involved combinations of system (98%), environmental (56%), and/or individual (87%) approaches in prenatal care (35%), hospital (22%), or primary care (21%) settings. Over half (62%) were conducted with socially disadvantaged families. More studies reported positive effects on feeding practices (71%), child diet (70%), breastfeeding (67%), and maternal diet (62%), compared with child (31%) or maternal (48%) weight and growth outcomes. Variation in outcome reporting and measurement limited the ability to make conclusions regarding effectiveness. Maintenance of upstream approaches was seldom reported. Study quality ranged from poor to good. Results suggest the promise of scaling adoption of policy, systems, and/or environmental approaches that enhance standard clinical care by incorporating nutrition-focused elements to support healthy feeding behaviors and growth. To achieve equitable nutrition and healthy growth early during the first 1000 d, implementation and evaluation of upstream policy efforts that integrate social and health care within and in collaboration with agencies beyond clinical settings may be warranted.

This trial was registered at Open Science Framework Registry as osf.io/bqck5 (https://doi.org/10.17605/OSF.IO/SXZMK).

**Statement of Significance**

This scoping review provides a summary of nutrition-focused and weight-related policy, system, environmental approaches in clinical settings and highlights opportunities to equitably promote healthy growth early in life.

## Introduction

Nutritional exposures during pregnancy and the first 2 y of a child’s life (i.e., the first 1000 d) influence growth, development, and lifelong risk for health conditions including obesity [[1], [2], [3], [4], [5]]. Exposures in utero and postnatally can influence the structure and function of infants’ organs and biological and behavioral systems involved with energy regulation [[6], [7], [8], [9]]. During the prenatal period, maternal nutritional status, diabetes mellitus, and gestational weight gain can influence fetal growth, preterm birth, birthweight, and subsequent risk for obesity [7,10,11]. After birth, differences in feeding patterns (e.g., breast or bottle, frequency, amount) and energy and protein content of human milk and infant formula can impact infants’ growth patterns and risk for obesity [4,12]. Finally, the timing of the introduction of complementary (i.e., solid) foods and parental feeding practices is linked with risk for obesity [13,14]. Any of these factors can contribute to rapid weight gain in the first 2 y of life, which is a “strong” risk factor for obesity in childhood and beyond [15].

Current nutrition-related recommendations for healthy growth and development include consuming a nutritious diet and gaining an adequate amount of weight during pregnancy, exclusively feeding human milk for the first 6 mo of life and continuing as mutually desired by mother and child until 2 y or beyond, responding to infants’ hunger and satiety cues, and introducing a variety of nutrient dense foods after 6 mo of age [[16], [17], [18], [19], [20]]. However, among pregnant people, the quality of dietary intake is typically low, and many have either insufficient or excess gestational weight gain [18,[21], [22], [23]]. Globally, 48% of infants are exclusively fed human milk the first 6 mo of life and approximately one-third of infants are fed something other than human milk or infant formula before 4 mo of age [24]. The quality and diversity of these complementary foods is typically low, characterized by little consumption of vegetables and whole grains and increased consumption of added sugar and sodium [[24], [25], [26]].

Previous reviews have identified promising obesity prevention interventions addressing these behaviors during the first 1000 d [[27], [28], [29], [30]], but the focus on individual behaviors of parents or children, rather than upstream or midstream interventions that influence conditions within which behaviors occur, limits public health impact [31]. Characteristics that distinguish upstream and midstream interventions include strategies that are part of ongoing, long-term plans to produce and sustain behavior change at a community or population, rather than individual level [32]. Upstream and midstream interventions typically involve policy, systems, and/or environmental (PSE) approaches to change the political, social, physical, and economic context in ways that ultimately facilitate or impede people’s behaviors to promote health [33,34]. Policy change can occur at national, regional, local, and/or organizational levels. Examples include passing laws or ordinances, or institutionalizing new procedures or rules. Systems change focuses on modifying infrastructure or processes within and between organizations. Examples include implementing a new program across a healthcare system or initiating new screening and referral protocols. Environmental change modifies physical, social, or economic factors that influence people’s practices and behaviors. Examples include adding signage to public spaces, campaigns to influence social norms on a topic, and financial incentives or disincentives [32,35]. The impact of upstream and midstream interventions on larger groups of people has the potential to drive or correct health inequities [36,37].

The healthcare setting (e.g., prenatal and primary care) provides great potential for supporting healthy nutrition for all, particularly groups who experience nutrition- and obesity-related disparities (i.e., people with lower income, racially and ethnically minoritized groups, and rural communities) [[38], [39], [40]]. Indeed, globally, >80% of pregnant people access prenatal care [41]. In the United States, 90% of young children aged 0–4 y attend well-child visits [42]. However, there are differences in visit adherence between high and low-middle-income countries as well as by family income, race and ethnicity, and urbanicity [41,43,44]. The American Academy of Pediatrics recommends that pediatricians and other health care providers promote and advocate for programs that support early-life nutrition [3]. Two reviews have synthesized evidence specifically for obesity prevention interventions delivered by health professionals during the first 1000 d [45,46]. Although there is promising evidence regarding the effectiveness of interventions focused on the individual behaviors of parents or children [27,45], there is generally a lack of synthesis of evidence of effective, feasible, and sustainable strategies to implement at scale through clinical settings [46,47].

PSE approaches in healthcare (i.e., clinical settings) that foster structural, sustainable change may provide equitable, long-term support for healthy nutrition behaviors for larger groups of people [31,48]. In fact, priorities in healthcare interventions have shifted to include these upstream approaches that address underlying conditions that impact population health [37]. However, to date, there is a lack of synthesis of the application of PSE approaches to improve nutrition environments and health outcomes through clinical settings [27]. Rather, reports of PSE approaches have focused on community settings [[49], [50], [51], [52]].

This scoping review addresses these identified gaps in the literature through characterizing the breadth of nutrition-focused PSE approaches in clinical settings for obesity prevention during the first 1000 d. Secondary aims included examining the generalizability and methodological quality of such interventions. Assessing current PSE approaches in the clinical setting to promote healthy weight will equitably promote healthy growth early in life by highlighting gaps in our knowledge and opportunities for future intervention and investigation.

## Methods

This review adhered to the PRISMA for Searching [53] and PRISMA for Scoping Reviews reporting guidelines [54]. The review protocol was registered in the Open Science Framework Registry (Registration identifier: osf.io/bqck5) prior to conducting the search.

### Information sources/search strategy

Two research librarians created the search strategy in Medline PubMed (Supplemental Table 1) and translated it to 7 additional databases: Web of Science Core Collection and CAB Abstracts, Cumulative Index to Nursing and Allied Health Literature with Full Text, Agricola via EBSCOHost, Cochrane Database of Systematic Reviews including CENTRAL, ProQuest’s Dissertations and Theses Global, Embase via Elsevier, and Google Scholar. Search strategies for each database reside in SearchRXIV [55]. Keywords and subject headings encompassed the concepts of the first 1000 d, nutrition, weight, environment, and policies. Because no prior reviews on this topic could be identified, all databases were searched from inception to 10 November, 2022. Due to the variety of populations, intervention approaches, and outcomes encompassed in the research question, the librarians crafted a comprehensive search strategy that was anticipated to have a high yield. Supplemental search strategies included contacting authors of clinical trial postings and conference abstracts for additional publications, and forward and backward citation chaining of included texts using the SpiderCite tool from Systematic Research-accelerator [56]. The SpiderCite tool can identify both cited and citing articles as well as potential gray literature not indexed in databases, which can result in a high-yield supplemental search [57]. Full database coverages, data management, and search tools can be found in the registered protocol (Open Science Framework Registry, osf.io/bqck5) that was established prior to the review [58].

### Eligibility criteria

Eligibility criteria included sources for which a full text was available in English and that: *1*) used an experimental or quasi-experimental study design, *2*) had ≥1 PSE intervention approach to support access to or availability of healthy foods and beverages, increase acceptability or consumption of healthy foods and beverages, promote positive feeding practices, or limit unhealthy foods and beverages, *3*) focused on any period of time between conception and age 2 (e.g., prenatal, infancy, toddler), *4*) included conduct within a clinical setting (e.g., hospital, prenatal or pediatric primary care), and *5*) examined nutrition (e.g., food insecurity, dietary intake, breastfeeding, feeding practices, complementary feeding) or weight-related outcomes (e.g., adiposity, growth trajectory) during the first 1000 d. Exclusion criteria were operationalized as references that: *1*) intervened with populations outside of the first 1000 d (e.g., prior to conception [59]), *2*) interventions focused on undernutrition, supplements, or formula composition [60], *3*) interventions that recruited participants through a clinical setting but did not deliver the intervention through the clinical setting [61], *4*) interventions that did not include a PSE approach (e.g., delivered by a research team rather than embedded within a clinical setting and/or implemented by clinical providers [62]), *5*) interventions conducted in nonclinical settings (e.g., home visiting programs or the Special Supplemental Nutrition Program for Women, Infants, and Children (WIC) [63]), and *6*) interventions that did not report any maternal or child nutrition- or weight-related outcomes (e.g., provision of services [64]). Supplemental Table 2 provides detailed inclusion and exclusion criteria.

### Study selection

All references were first exported to EndNote [65] for removal of non-English articles and duplicates. References were then uploaded to Covidence [66], a systematic review software, to check for remaining duplicates, screen titles and abstracts, and review full text. Prior to commencing screening and subsequent full-text review, all reviewers completed training and passed a pilot test of applying inclusion/exclusion criteria. Titles and abstracts, followed by full texts, were independently screened in duplicate, with conflicts resolved by a third reviewer.

### Data extraction and quality assessment

Data extraction was informed by an extension of the RE-AIM (reach, effectiveness, adoption, implementation, maintenance) framework, one of the most frequently applied frameworks for planning and evaluating clinical, public health, and community interventions [67,68]. This extension promotes a more balanced assessment of internal and external validity, allowing for commentary on issues or dimensions that could influence equity of public health impact [67,69]. An extraction template was developed for this project, piloted by the team, and refined prior to data extraction. Extracted elements included general study characteristics (i.e., study design, comparators, country, timing of study, length of intervention, use of behavior theories or implementation models) and each dimension of RE-AIM (Table 1) [67]. Prior to data extraction, included full-text sources that reported on the same study were grouped together; all texts in each grouping were reviewed, and data points were aggregated as a single entry.

**TABLE 1:** Data points extracted for RE-AIM [67] dimensions.

Dimension	Data points
Reach	Number, proportion, and representatives of individuals who participated Inclusion/exclusion criteria
Effectiveness	Directionality and statistical significance of impact on outcomes of interest Unintended consequences Evaluation of heterogeneity of effects
Adoption	Number and representativeness of settings Interventionists responsible for implementation
Implementation	Intervention components Policy, systems, and/or environmental approaches Reporting evaluation of delivery as intended (i.e., fidelity), adaptations, and cost
Maintenance	Reporting sustainability of intervention beyond the study period Reporting evaluation of longer-term effects

Abbreviation: RE-AIM, reach, effectiveness/efficacy, adoption, implementation, maintenance.

Scoping reviews do not require assessment of risk of bias [54]; however, to identify gaps and opportunities regarding the evaluation of PSE approaches, the methodological quality of included studies was assessed using the Downs and Black Checklist [70]. This 27-item checklist evaluates the reporting, external validity, internal validity (bias and confounding), and power of randomized and nonrandomized study designs. As with previous reviews [71], rather than the 5-point scale, the power question was modified to assess whether a power analysis was reported. Randomized studies have a maximum score of 28, and nonrandomized studies have a maximum score of 25. The quality of studies was categorized as excellent (26–28), good (20–25), fair (15–19), or poor (≤14) [72]. For full texts grouped together, a primary source was identified for checklist completion (e.g., primary outcome paper rather than a protocol paper). Quality assessment was only completed for peer-reviewed articles with reported outcomes because other information types (i.e., conference abstracts, clinical trial listings, protocol papers) did not contain sufficient details for evaluation. Reviewers independently extracted information and completed the Downs and Black Checklist. A second reviewer checked all extracted data and quality checklists.

### Synthesis of results

Descriptive statistics were calculated to describe study characteristics. To characterize the breadth of PSE approaches (aim 1), frequencies and percentages were used to report life stage(s) intervened upon, intervention components, study designs, comparators, measured nutrition- and obesity-related outcomes, and duration of studies. Data visualization was completed using the R programming language within the RStudio integrated development environment [73,74]. An UpSet plot was generated using the UpSetR package (v1.4.0) [75,76]. To report on the generalizability (aim 2) and methodological quality (aim 3) of included studies, frequencies and central tendencies were calculated. Numerical analysis was used along with matrices of qualitative information to generate a narrative summary.

## Results

**FIGURE 1:**
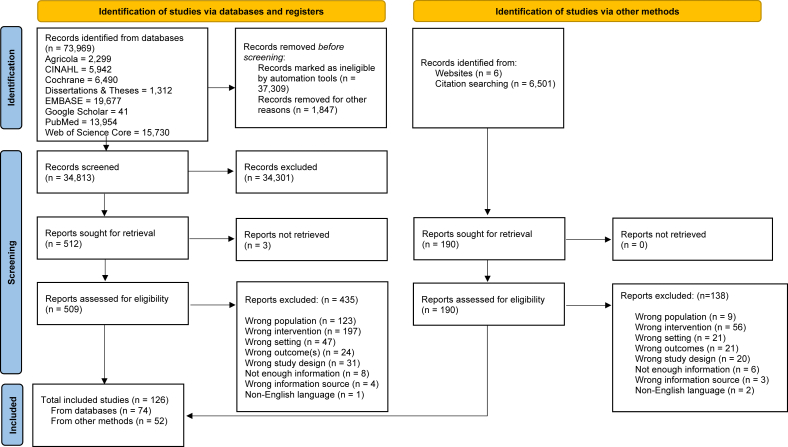
PRISMA diagram for study identification, screening, and inclusion. *From:* M.J. Page, J.E. McKenzie, P.M. Bossuyt, I. Boutron, T.C. Hoffmann, C.D. Mulrow, et al. The PRISMA 2020 statement: an updated guideline for reporting systematic reviews. BMJ. 372 (2021) n71, https://doi.org/10.1136/bmj.n71. For more information, visit: http://www.prisma-statement.org/.

After deduplication and removal of items marked ineligible by automation tools, 34,813 records were screened at the title/abstract phase, and 509 full texts were reviewed for inclusion, including 190 additional full texts from supplemental searches. Ultimately, 185 reports representing 126 unique studies/interventions (herein: studies) in clinical settings were included (Figure 1). Reports were most frequently excluded during full-text review because the intervention did not have ≥1 PSE component (n = 253, 44%) or the population intervened upon was outside the scope of the first 1000 d (n = 132, 23%) (Supplemental Table 3). Studies were reported in a variety of sources, including clinical trial listings (7/126, 6%), conference abstracts (7/126, 6%), and dissertations (3/126, 2%), with most reported in peer-reviewed manuscripts (109/126, 87%).

### Aim 1. Breadth of included studies about nutrition-focused PSE approaches

#### Study characteristics

A summary of study characteristics is provided in Table 2. Studies were most frequently conducted during early infancy (child aged 0–3 mo, 94/126, 75%), with some spanning both prenatal and early infancy (44/126, 35%). The fewest number of studies were conducted during toddlerhood (child aged 12–24 mo, 18/126, 14%). Four studies (3%) had an intervention approach that spanned the full 1000 d period [[77], [78], [79], [80]]. The duration of interventions varied from a 15-min video about breastfeeding added to hospital admission procedures [81] to a 6-y intervention that began in pregnancy and continued until age 5 [79]. The median duration of intervention was 8 mo.

**TABLE 2:** Characteristics of included studies of clinically based nutrition-focused policy, system, and/or environmental interventions for obesity prevention during the first 1000 d (*n* = 126).

Characteristic	*N*	(%)
Life stage intervened upon^1^		
Prenatal	71	56
0–3 mo	94	75
3–6 mo	53	42
6–12 mo	33	26
12–24 mo	18	14
Continuity across the first 1000 d	4	3
Intervention components^1^		
Policy	19	15
System	124	98
Environment	70	56
Physical environment^2^	35	50
Social environment^2^	51	73
Economic environment^2^	6	9
Individual	110	87
Intervention approaches^3^		
P-only	1	1
S-only	12	9
P + S	1	1
S + E	1	1
S + I	41	33
E + I	1	1
P + S + I	1	1
S + E + I	52	41
P + S + E	1	1
P + S + E + I	15	12
Study design		
Randomized controlled trial	53	42
Nonrandomized controlled trial	23	18
Retrospective cohort	16	13
Repeated cross-sectional	13	10
Cohort	10	8
Single group, pre-post design	6	5
Other^4^	5	4
Comparator		
Standard of care	81	64
No control	13	10
No intervention	10	8
Attention control	2	2
Delayed	2	2
Other^5^	18	14
Nutrition-related outcomes evaluated^1^		
Breastfeeding	90	71
Child growth/obesity	52	41
Maternal growth/obesity	35	28
Feeding practices	28	22
Maternal diet	20	16
Child diet	16	13
Food security	3	2
Year study initiated		
1980s	1	1
1990s	8	6
2000s	43	34
2010s	51	40
2020s	4	3
Not reported	19	15

1 Studies could be counted in > 1 response category.

2 Denominator based upon studies that included an environmental component (*n* = 70).

3 E, Environmental; I, Individual; P, Policy; S, System.

4 Other study designs include: time series, stepped wedge, randomized controlled trial with quasi-experimental.

5 Other comparators include: historical control, enhanced standard care, participant as own control, and national dataset.

**FIGURE 2:**
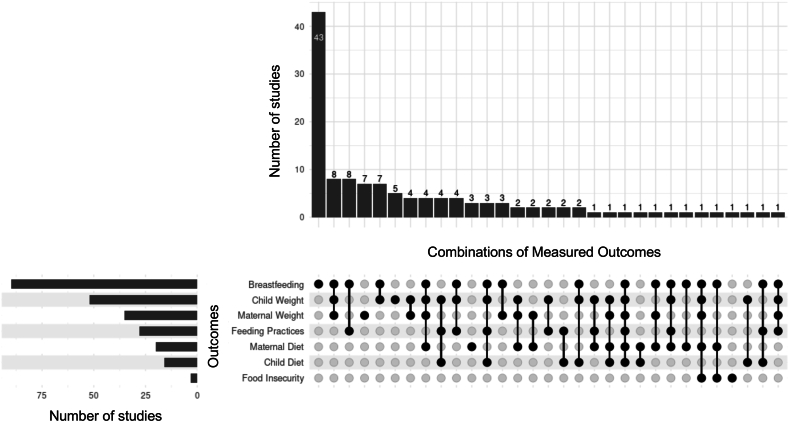
Visualization of the density and intersection of measured nutrition- and weight-related outcomes of nutrition-focused upstream interventions for obesity prevention in clinical settings during the first 1000 d.

Over half of the studies (73/126, 58%) utilized a quasi-experimental design. A small proportion of studies (18/126, 14%) were a pilot and/or feasibility trial. Most studies included a comparison group (113/126, 90%), which was typically standard of care (81/113, 72%). It was less common to mention theoretical models for the intervention (24/126, 19%), such as application of behavior change theories (e.g., social cognitive theory [78,[82], [83], [84], [85], [86]], Transtheoretical Model [87,88]), intervention design models (e.g., PRECEDE PROCEED [89,90]), and/or implementation frameworks (e.g., RE-AIM [91], Theoretical Domains Framework [92]). The most frequently evaluated nutrition-related outcomes were breastfeeding (90/126, 71%) and child growth/obesity (52/126, 41%) (Figure 2). Breastfeeding outcomes typically encompassed exclusivity (53/90, 59%) or initiation (23/90, 26%).

#### Intervention approaches

An overview of intervention approaches is shown in Table 2. Almost all studies included a systems approach (124/126, 98%), meaning nutrition-related factors were incorporated or changed via rules, infrastructure, processes, or procedures within the clinical setting or between the clinical setting and another organization or sector. Environmental approaches to incorporate or change nutrition-related factors through services, physical surroundings, social influences, and/or financial incentives or disincentives were included in about half of the studies (70/126, 56%). Policy approaches were infrequently used (19/126, 15%). Studies rarely utilized a single approach (policy-only, 1/126, 0.8%; system-only, 12/126, 10%; environmental-only, 0/126). Instead, studies usually (113/126, 90%) employed combinations of PSE approaches with individual-focused components.

The most common policy approach aimed to promote breastfeeding in hospitals through adoption of the Ten Steps to Successful Breastfeeding (13/19, 68%). This policy approach also included systems, physical (i.e., structural changes, programs, or services) and social (i.e., support, attitudes, or actions) environmental, and individual-focused approaches. Other policies occurred within primary care settings and included systems and/or environmental approaches, such as requiring medical staff to offer group care to prenatal patients instead of individual care [93], requiring training for weight-related prenatal care and documentation of efforts [90], or including a lactation specialist at the first infant well-check visit [94,95]. One policy focused on efforts to create an infant-friendly community through connecting the hospital and community as a multisectoral approach to improve infant and young child feeding practices [96].

Systems, environmental, and individual approaches were often used together (52/126, 41%). The social environment was the most common environmental approach (37/52, 71%), and it was often incorporated through a systems change to standard care to include group care models like CenteringPregnancy (*n =* 14) or group well-child care (*n =* 2). Other approaches supported breastfeeding through peer support from counselors or postpartum groups (*n =* 10) and during well-child visits (*n =* 3). Doula programs were also incorporated for prenatal, in-hospital, and postpartum support [97,98]. One study integrated screening and referral for a nutrition education program into standard of care; the prenatal program included classes which provided ∼20 pounds of fresh, donated produce along with recipe cards and social support from community health workers [85].

Studies also applied combinations of systems and individual approaches (41/126, 33%) that included routine weighing and nutrition counseling during pregnancy [89,99,100], screening and/or referral processes [101,102], access to accredited lactation consultants for individualized support in hospital or pediatric primary care [[103], [104], [105], [106], [107]], and integrating nutrition-focused interactions into routine primary prenatal or pediatric care visits (n = 24). One study applied data integration for care coordination between pediatric primary care providers and WIC nutritionists [108].

Physical environment approaches (35/126, 28%) included creating new clinical spaces and services for gestational weight gain [[109], [110], [111]], general nutrition [112], or breastfeeding support [[113], [114], [115]] as well as adding health promotion materials to waiting room spaces [116,117] or offering a new platform to facilitate telehealth [91]. Five studies provided breast pumps and supplies [[118], [119], [120], [121], [122]]. Studies less frequently incorporated economic environment approaches (6/126, 5%). Examples include providing generic formula for families feeding formula who have food insecurity [123], vouchers or other price reductions for community lifestyle programs for pregnant people [86,124], vouchers for fruits and vegetables during pregnancy and postpartum [125], equipment to help families optimize their lifestyle during pregnancy and the child’s first year [126], and cell phones to connect with certified lactation counselors [127].

### Aim 2. Generalizability of included studies about nutrition-focused PSE approaches

An overview of elements depicting the generalizability of the included studies is shown in Table 3 [67]. Specific details for each study regarding the reach, effectiveness, adoption, implementation, and maintenance of interventions are shown in Supplemental Table 4.

**TABLE 3:** Dimensions of RE-AIM [67] summarizing the generalizability of clinically based nutrition-focused policy, system, and/or environmental interventions for obesity prevention during the first 1000 d (*n* = 126).

Characteristic	N	(%)
Reach		
	Median	Range
Final sample size, people	376	17–39,272
Priority population groups^1^	78	62
People with lower income	52	41
Racially or ethnically minoritized groups	49	39
People with overweight or obesity	19	15
Rural	9	7
Low education	8	6
Other^2^	10	8
Included male caregivers	12	10
Reported representativeness of sample	37	29
	Median	Range
Rate of attrition, (%)	12	0–67
Effectiveness		
Reported positive outcome^1^^,^^3^		
Breastfeeding (n = 78)	52	67
Child growth/obesity (n = 42)	13	31
Maternal growth/obesity (n = 27)	13	48
Feeding practices (n = 24)	17	71
Maternal diet (n = 13)	8	62
Child diet (n = 10)	7	70
Food security (n = 2)	2	100
Reported unintended outcomes	18	14
Adoption		
Clinical setting		
Prenatal care	44	35
Pediatric primary care	26	21
Hospital	28	22
Clinical + community	19	15
Prenatal + pediatric primary care	8	6
Clinical, not specified	1	1
Conducted within single clinic site	48	38
Reported representativeness of setting	22	17
Implementation		
Reported assessment of fidelity	61	48
Reported assessment of cost	10	8
Maintenance		
Intervention sustained beyond study	26	21
Reported sustainability of impact	31	25

Abbreviation: RE-AIM, reach, effectiveness/efficacy, adoption, implementation, maintenance.

1 Studies could be counted in >1 response category.

2 Other priority population groups include immigrants, adolescent mothers, tribal, indigenous, or aboriginal people.

3 Sample sizes exclude ongoing studies.

#### Reach

Some studies included people with lower income (52/126, 41%) or from minoritized racial and/or ethnic groups (49/126, 39%). Fewer studies included people from rural areas (n = 9), adolescent mothers (n = 3), or tribal, indigenous, or aboriginal people (n = 1). Twelve studies (10%) explicitly mentioned including male caregivers. Nearly one-third of studies (37/126, 29%) reported the representativeness of the sample compared with the target population, with 9% (11/126) of studies explicitly reporting sample selection methods. The median analytic sample size was 376 mother–infant dyads, with samples as small as 17 parents in a pilot study about brief responsive feeding education during routine well-baby visits [128] and as large as 39,272 infants when looking at Baby-Friendly Hospital Initiatives in 33 hospitals across 4 states of the United States [129]. The median rate of attrition was 12% but reached as high as 67% [124].

#### Effectiveness

Studies utilized a variety of measures to evaluate maternal and child outcomes. Breastfeeding outcomes included initiation, duration, exclusivity, partial or any, and current at varying time points. Child growth/obesity was reported by birth weight, macrosomia, (excess) weight gain, weight-for-length, weight-for-age, BMI (in kg/m^2^), z-scores, and percent fat mass at varying time points. Maternal growth/obesity was often reported as gestational weight gain (total and/or weekly) and/or weight gain related to recommendations based on prenatal BMI. Changes in maternal and child dietary intake outcomes ranged from specific eating behaviors (e.g., breakfast consumption) to intake of specific foods or beverages (e.g., sugar-sweetened beverages, fruits and vegetables, ultraprocessed foods), more general food frequency questionnaires, and diet quality scores (e.g., dietary diversity and alignment with national dietary guidelines). Changes in feeding practices were based upon questionnaires about infant and child feeding practices, complementary feeding, timing of introduction of complementary foods, parental feeding style, and bottle feeding.

Due to the variety of outcome measures, effectiveness is summarized as the directionality of change reported. The majority reported positive impact on child feeding practices (17/24, 71%) as well as breastfeeding (52/78, 67%). However, fewer studies reported positive impact on children’s growth/obesity (13/42, 31%). Two of the 3 studies assessing food security reported positive outcomes [85,130], and results for the third study are forthcoming [131]. A small number of studies reported negative outcomes for breastfeeding (n = 5) [105,119,[132], [133], [134]], feeding practices (n = 3) [114,128,135], maternal growth/obesity (n = 2) [125,134], and child growth/obesity (n = 1) [136]. Nineteen studies (15%) are ongoing or have yet to publish results. Eighteen (14%) studies reported the presence or absence of unintended consequences (e.g., birth outcomes).

#### Adoption

Most studies (108/126, 86%) were conducted in high-income countries including the United States (n = 66), Australia (n = 10), and the United Kingdom (n = 5). Fifteen studies were conducted in low- and middle-income countries across 4 continents, with 4 studies conducted in Brazil. More than half of studies (78/126, 62%) were conducted in prenatal or primary care settings, with 8 starting in prenatal care and continuing into primary care. Half of the studies (64/126, 51%) were conducted at >1 clinical site (e.g., multiple hospitals), with 17% (22/126) of studies reporting the representativeness of participating sites. Fourteen studies (11%) implemented interventions across clinical and community sites. Hospital-community [85,96,137,138] and hospital-home visiting [98,139] collaborations consistently focused on breastfeeding. Primary care-community collaborations focused on food insecurity [123], healthy weight gain during pregnancy [86], screening for infant obesity [102], and improving weaning practices [140]. Collaborations between primary care and the Special Supplemental Nutrition Program for WIC focused on responsive feeding through group well-child care [141] and care coordination [108]. One primary care-community-home visiting program focused on maternal nutrition during pregnancy [142].

#### Implementation and maintenance

Those responsible for implementing interventions varied widely and usually included >1 member of a multidisciplinary team of physicians, midwives, nurse practitioners, nurses, International Board-Certified lactation consultants, dietitians, doulas, and/or administrative staff. Paraprofessionals like peer counselors or community health workers were rarely involved (14/126, 11%) (Supplemental Table 4). Approximately half of the studies (61/126) reported on the fidelity with which the intervention was implemented. Fewer studies reported on adaptations to the implementation approach (11/126, 9%) and cost of implementation (10/126, 8%). Some studies (26/126, 21%) reported continuation of the intervention beyond the evaluation period, but it was unclear whether the majority continued beyond the trial period (89/126, 71%). Similarly, some studies (31/126, 25%) reported longer-term impact of the intervention (e.g., breastfeeding rates 3–6 mo after ending participation in the intervention) or plans to evaluate as such.

### Aim 3. Methodological quality of included studies about nutrition-focused PSE approaches

The quality of the primary source of included studies, where quality could be assessed (n = 99) is reported in Table 4 [70,[78], [79], [80],^82^,^85^,[87], [88], [89], [90],[93], [94], [95], [96], [97], [98], [99], [100],[102], [103], [104], [105], [106], [107], [108], [109], [110], [111], [112], [113], [114], [115], [116], [117], [118], [119], [120], [121],[123], [124], [125],[127], [128], [129], [130],[132], [133], [134],[136], [137], [138], [139], [140], [141],[143], [144], [145], [146], [147], [148], [149], [150], [151], [152], [153], [154], [155], [156], [157], [158], [159], [160], [161], [162], [163], [164], [165], [166], [167], [168], [169], [170], [171], [172], [173], [174], [175], [176], [177], [178], [179], [180], [181], [182], [183], [184], [185], [186]]. Detailed information by study can be found in Supplemental Table 5. Most studies were classified as fair (47/99, 47%) or good (40/99, 40%). Twelve studies (12%) were classified as poor, and no studies scored in the excellent category. On the basis of median scores, studies scored higher on reporting (9/11 points) and bias elements (5/7 points) but scored lower on confounding (3/6 points) and external validity (1/3 points) elements. Lower quality scores were often attributed to a lack of reporting regarding the: 1) representativeness of the participants invited to participate compared with the entire population, 2) representativeness of the sample from the population recruited from, 3) adequate adjustment for confounding in main analyses, and 4) adverse events that may be a consequence of an intervention. Additionally, 63% (62/99) of studies for which quality could be assessed did not use or report randomization for intervention groups. Approximately half of the studies (46/99) reported a power calculation. Most studies (107/126, 85%) reported their funding source (Supplemental Table 6).

**TABLE 4:** Quality of included studies as assessed by the Downs and Black Checklist [70] (*n* = 99^1^).

Maximum points available	Reporting 11 points	External validity 3 points	Internal validity – bias 7 points	Internal validity – confounding 6 points	Power 1 point	Total	Quality rating^2^
First author, year							
Randomized studies (maximum score = 28 points)							
Rybak et al. [143], 2023	11	3	6	5	0	25	Good
Hopkinson et al. [114], 2009	10	1	6	6	1	24	Good
Parat et al. [144], 2019	11	1	6	5	1	24	Good
Su et al. [106], 2007	11	1	6	5	1	24	Good
Garmendia et al. [145], 2020	11	1	5	5	1	23	Good
Gross et al. [78], 2016	11	0	6	5	1	23	Good
Milinco et al. [104], 2020	9	1	6	6	1	23	Good
Simpson et al. [86], 2021	11	2	5	4	1	23	Good
Chapman et al. [121], 2013	9	0	6	6	1	22	Good
Daley et al. [100], 2015	10	2	5	4	1	22	Good
Döring et al. [82], 2016	10	1	5	5	1	22	Good
French et al. [146], 2012	10	1	5	5	1	22	Good
Paul et al. [139], 2012	9	1	5	6	1	22	Good
Bonuck et al. [118], 2014	10	0	5	5	1	21	Good
Brownfoot et al. [99], 2016	10	0	5	5	1	21	Good
Ferreira et al. [117], 2018	11	1	5	3	1	21	Good
Gagnon et al. [147], 1997	10	0	5	5	1	21	Good
Hoffman et al. [148], 2021	10	1	5	4	1	21	Good
Kramer et al. [149], 2001	10	1	4	5	1	21	Good
Maingi et al. [96], 2018	10	1	5	4	1	21	Good
Savage et al. [108], 2022	10	1	4	5	1	21	Good
Ickovics et al. [150], 2016	10	0	4	5	1	20	Good
Patel et al. [127], 2018	9	0	5	5	1	20	Good
Sangalli et al. [116], 2021	8	2	5	4	1	20	Good
Vlasblom et al. [135], 2020	9	1	4	5	1	20	Good
Wang et al. [151], 2019	8	3	5	3	1	20	Good
Ekström et al. [152], 2014	10	0	5	3	1	19	Fair
Rasmussen et al. [119], 2011	9	1	4	5	0	19	Fair
Sanders et al. [153], 2021	9	0	5	4	1	19	Fair
Tubay et al. [154], 2019	9	0	4	5	1	19	Fair
Anderson et al. [155], 2005	8	1	5	4	0	18	Fair
Chapman et al. [120], 2004	8	1	4	5	0	18	Fair
Ekström et al. [156], 2012	9	1	5	3	0	18	Fair
Schroeder et al. [157], 2015	8	1	4	4	0	17	Fair
Pugh et al. [138], 2002	8	0	4	4	0	16	Fair
Winterburn et al. [158], 2000	6	0	4	4	1	15	Fair
Nonrandomized studies (maximum score = 25 points)							
Tanner-Smith et al. [159], 2013	10	3	5	4	0	22	Good
Kettrey et al. [136], 2020	9	3	5	3	1	21	Good
Machuca et al. [88], 2016	10	2	5	4	0	21	Good
Mottl-Santiago et al. [97], 2007	10	1	5	4	1	21	Good
Nommsen-Rivers et al. [98], 2009	10	1	5	4	1	21	Good
Taveras et al. [80], 2021	11	1	5	3	1	21	Good
Corriveau et al. [95], 2013	8	2	6	3	1	20	Good
De Jersey et al. [89], 2022	9	2	6	3	0	20	Good
Gomes et al. [87], 2019	9	3	5	3	0	20	Good
Haby et al. [124], 2015	10	1	5	4	0	20	Good
Heberlein et al. [130], 2016	10	1	5	4	0	20	Good
Malta et al. [160], 2021	10	1	5	3	1	20	Good
Merten et al. [161], 2005	9	2	5	4	0	20	Good
Tarrant et al. [162], 2015	10	1	5	3	1	20	Good
Beck et al. [123], 2014	9	3	4	3	0	19	Fair
Chae et al. [163], 2017	9	1	5	3	1	19	Fair
Flax et al. [164], 2022	10	1	4	3	1	19	Fair
Gross et al. [165], 2022	9	2	4	4	0	19	Fair
Hawkins et al. [166], 2015	10	1	5	3	0	19	Fair
Merewood et al. [129], 2019	10	2	4	3	0	19	Fair
Minkovitz et al. [167], 2001	8	1	5	5	0	19	Fair
Mustila et al. [79], 2013	11	1	5	2	0	19	Fair
Rosen-Carole et al. [168], 2016	10	2	5	2	0	19	Fair
Sharma et al. [85], 2018	10	0	5	4	0	19	Fair
Trotman et al. [169], 2015	10	0	5	4	0	19	Fair
Trudnak et al. [134], 2011	9	1	5	4	0	19	Fair
Walton et al. et al. [170], 2015	11	0	5	2	1	19	Fair
Wilkinson et al. [112], 2018	10	1	5	3	0	19	Fair
Zielinski et al. [93], 2014	9	1	5	4	0	19	Fair
Budge et al. [141], 2023	9	2	4	3	0	18	Fair
Chwah et al. [171], 2016	10	1	4	3	0	18	Fair
Grossman et al. [172], 2009	9	3	3	2	1	18	Fair
Kair et al. [133], 2013	8	3	5	2	0	18	Fair
Robertson et al. [173], 2009	10	1	5	2	0	18	Fair
Rosen et al. [174], 2008	8	1	5	3	1	18	Fair
Tarrant et al. [175], 2011	9	1	4	4	0	18	Fair
Watt et al. [125], 2015	8	0	5	4	1	18	Fair
Witt et al. [107], 2021	9	1	5	3	0	18	Fair
Brodribb et al. [132], 2013	8	2	5	2	0	17	Fair
Feldman-Winter et al. [176], 2010	10	0	4	2	1	17	Fair
Kinnunen et al. [177], 2007	9	1	5	2	0	17	Fair
Kistin et al. [137], 1994	9	1	4	3	0	17	Fair
Taveras et al. [178], 2011	9	1	4	3	0	17	Fair
Donkoh et al. [179], 2013	8	1	4	2	1	16	Fair
Hannula et al. [113], 2014	9	1	4	2	0	16	Fair
Olayiwola et al. [111], 2013	8	1	5	2	0	16	Fair
Witt et al. [94], 2012	8	1	5	1	1	16	Fair
Graça et al. [180], 2011	9	2	3	1	0	15	Fair
Gregory et al. [109], 2016	9	0	4	2	0	15	Fair
Klima et al. [181], 2009	8	0	4	3	0	15	Fair
McGiveron et al. [110], 2015	8	0	4	3	0	15	Fair
Brumley et al. [182], 2016	8	1	3	2	0	14	Poor
Chiurco et al. [103], 2015	8	0	3	2	0	13	Poor
Hoch et al. [90], 2023	6	1	4	1	1	13	Poor
Nickel et al. [183], 2011	6	1	5	1	0	13	Poor
Scott et al. [105], 2015	6	1	4	2	0	13	Poor
Grant et al. [184], 2018	5	0	4	3	0	12	Poor
Holmes et al. [185], 2012	5	0	5	2	0	12	Poor
Metwally et al. [140], 2022	7	1	4	0	0	12	Poor
Redsell et al. [102], 2017	5	1	3	3	0	12	Poor
Abrahams et al. [186], 2009	5	0	4	1	0	10	Poor
Alberdi et al. [115], 2018	5	0	4	1	0	10	Poor
Hale et al. [128], 2023	4	0	5	1	0	10	Poor

1 Downs and Black Checklist not completed for studies reported only in clinical trials registry, conference abstract, or protocol paper (*n* = 27).

2 Quality rating: excellent (26–28), good (20–25), fair (15–19), and poor (≤14).

## Discussion

This scoping review characterized the breadth, generalizability, and methodological rigor of nutrition-focused PSE interventions for obesity prevention during the first 1000 d.

Interventions frequently involved combinations of systems, environmental, and/or individual-based approaches to enhance standard care in prenatal care, primary care, and hospital settings, namely, to improve breastfeeding and early infant growth. Approximately half of the studies were conducted with socially disadvantaged families. Studies generally reported positive effects for feeding practices, child diet, breastfeeding, and maternal diet, whereas generally reporting null effects for child or maternal measures of growth or obesity. The sustained implementation and impact of interventions were infrequently reported or evaluated. Study quality was mainly classified as either fair or good, not excellent. These results suggest that to expand reach and impact, particularly among historically underserved groups who experience nutrition- and obesity-related disparities, there is a need for implementation of nutrition-focused PSE approaches through clinical settings that begin further upstream to address social needs and improve community conditions. There is also a need for higher-quality evaluation and reporting of the approaches, particularly for the effectiveness of these approaches.

Upstream interventions involving PSE change are promising approaches to address conditions contributing to health disparities due to the potential for addressing drivers of health, scalability, and ultimately population-level impact [37,187]. Findings from this review show that systems approaches to incorporate nutrition education counseling by physicians, midwives, nurses, lactation consultants, and/or registered dietitian nutritionists into standard visits for existing care models were common and feasible. This approach was more frequently utilized in pediatric primary care settings, whereas prenatal care settings more frequently incorporated social environmental approaches (i.e., group/peer support) with systems change to add education or counseling. Hospital settings were more likely to include policy change along with systems, environmental, and individual-focused approaches. Integrating nutrition-focused interventions into health systems through standard of care could be an efficient, effective service model [188]. The difference in approaches across clinical settings may reflect the need to select and tailor strategies based on the setting [189,190], and it is also a call for research to test strategies less frequently used.

Adoption and implementation of PSE approaches in clinical settings will require context-specific consideration of potential facilitators and barriers related to leadership and governance (extent of integration into health policies and strategies), financing for initiatives, information systems and communication across stakeholders, availability and capability of the healthcare workforce, access to necessary supplies/technology, and service delivery mechanisms [188,191]. Most of the studies in this review were conducted in the United States. The health care system in the United States consists of a mix of private and public, nonprofit and for-profit insurers and providers. This greatly differs from other high- and middle-income countries represented in this review that have universal health insurance and national care systems [192]. Coordination, or the lack thereof, of regulation, financing, and services across federal/national, state/regional, and local contributors to health systems may influence the adoption, implementation, and ultimately scale of PSE approaches to address health issues [193]. Ultimately, the unique health priorities and economic, political, and social conditions of each country need to be considered. In addition to considering country-specific context, potential facilitators and barriers to implement PSE approaches within practice settings need to be considered. For example, a checklist is available for planning implementation of “food as medicine” programs in healthcare settings that is based upon previously identified barriers and facilitators of these programs [194]. Criteria include such factors as identifying a champion at multiple levels of the healthcare organization or system, identifying organizational and community partners, building close-looped communication and data sharing systems, and shifting responsibilities within clinical teams so that everyone has a clear role in supporting the program. Future implementation research will provide greater detail about how to best integrate PSE approaches in clinical settings [195].

Systematic changes to organizational practices and policies can ensure reach to all patients accessing clinical care, and yet systematic application of nutrition education or counseling alone may not yield consistent, clinically relevant change [196]. Adherence to prenatal and well-child visits varies widely, particularly among socially disadvantaged families [41,43,197], and the length of visits is often <20 min, and little time is spent on nutrition counseling [[198], [199], [200]], highlighting missed opportunities. Furthermore, the availability of resources (e.g., personnel, training, time, and reimbursement) is usually lacking, potentially limiting widespread adoption of such approaches [[201], [202], [203]]. Before the potential of nutrition-focused efforts in clinical settings can be realized, barriers to access to care and reimbursement models may need to be addressed [[204], [205], [206]], and specific emphasis on maternal nutrition, in addition to the child, may be necessary [207,208]. Additionally, although these approaches elevate the standard of clinical care to include nutrition- and growth-focused support, the greatest impact will occur when multiple PSE approaches are implemented that focus on both individuals and communities to integrate social and health care [35,187,209,210].

Few studies were implemented for the full duration of the 1000 d period. In considering the rapid nutrition transitions during this critical period of growth and development, there were notable gaps in ongoing support, with a small proportion of studies addressing feeding practices or children’s dietary intake beyond breast or bottle feeding, and few interventions occurred during toddlerhood. The paucity of interventions during toddlerhood in clinical settings may be related to the nuance of recommendations for complementary feeding [211] and the decreased frequency in well-child checks from 7 visits between age 0 and 12 mo to 3 visits between age 12 and 24 mo [212]. Studies in this sample that intervened during toddlerhood typically had intervention periods that started during infancy and continued into toddlerhood and had additional touch points during or between clinical visits through a technology (e.g., app or text) or group support medium, suggesting that continuity of care may be critical for intervening during the later portion of this critical period of development. Continuity of care can help address gaps while supporting diverse patient populations [213,214]. Collaboration among hospital or prenatal/pediatric care settings and community-based organizations is an example of systems change to bridge gaps in continuity of care [215]. Utilizing group care models or including doulas, community health workers, or peer counselors in the care model are examples of systems and social environment changes that can help families navigate health care systems, improve culturally competent care, and extend education and social support beyond the clinical encounter and throughout the 1000 d period [216,217]. Policy efforts further upstream can formalize cross-sector efforts and reimbursement for services to provide more equitable access to clinical lactation and care services during the first 1000 d [[218], [219], [220]].

Access to quality healthcare is an important contributor to health, and yet the social and economic contexts within which people live and receive care have even greater influence on healthcare utilization and health outcomes [37,[221], [222], [223]]. An individual’s education, economic and housing stability, neighborhood and built environment, and social and community context (i.e., social determinants of health) influence the access to and resources for taking part or benefiting from interventions [224]. As such, integration of health care and social services that address social drivers of health (e.g., housing, income support) may be necessary to achieve impact on obesity prevention [225,226]. Incorporating actions into clinical practice that address social drivers of health like food insecurity may be a useful PSE approach [227], as food insecurity may have cumulative effects on children’s dietary intake, risk for obesity, and general health [228]. Globally, food insecurity is on the rise [229], yet only 3 studies in this sample addressed and measured food insecurity [85,130,131]. Food insecurity was addressed and assessed in prenatal care settings, but not in other clinical settings. Several countries recommend screening for food insecurity and referring to emergency food services and/or nutrition assistance programs as part of clinical practice [[230], [231], [232]]. Findings from this review reinforce the need for implementing these recommendations. There is also a need for connecting people with services and robust evaluation to determine whether equitable benefit is experienced with intervention [35,233,234]. There may also be opportunities to explore the effects of PSE changes for “food as medicine” initiatives during the first 1000 d in which healthcare systems either closely coordinate or integrate food and nutrition interventions as a way to complement nutrition assistance programs [235]. Examples of these types of initiatives include healthy produce prescriptions/farmacies [236,237] and medically tailored groceries or meals [238].

Results from this review highlight several opportunities to enhance the methodological quality and reporting of studies conducted in clinical settings. Randomization is typically viewed as a foundation for high-quality study design [239], and some studies in this review used a cluster randomized control design. Study designs across prenatal care and pediatric primary care settings were similar, in that most utilized a randomized, or nonrandomized, design that included a control group. However, randomization may not be acceptable or practical in clinical settings, and alternative experimental designs may be needed for rigorous evaluation [239]. In this review, 4 studies employed pragmatic designs (e.g., interrupted time series, stepped wedge) [80,92,168,186] that may be a better fit for clinical settings [240]. There is also a need for more consistent reporting of the representativeness of patient groups included in research studies, as well as the clinical settings in which interventions are conducted, and the clinicians and healthcare staff needed to implement them [241,242]. Additionally, reporting the fidelity and/or adaptations with which interventions are implemented is critical for interpreting the effect size or lack thereof [243]. Reporting fidelity and adaptations over time will also help address gaps in understanding the sustained implementation and impact of interventions over time. The gaps in reporting maintenance in this review are consistent with reviews of efforts in a variety of healthcare service delivery sectors and settings and health behaviors and outcomes [244]. Challenges in conducting and reporting research about sustained implementation and impact may be due to malaligned research priorities and funding timelines [245,246]; clarity and consistency in defining and assessing sustainability as a dynamic, rather than static, process [244,247], and contextual factors influencing implementation and the need for adaptations (e.g., leadership, training/support, workforce turnover) [247,248]. Suggestions for more adequately addressing sustainability include: *1*) investing in research resources that allow for theory/framework-informed design, *2*) evaluation of ongoing adaptations of interventions and implementation strategies, and *3*) capacity building necessary to meet the needs of people and contextual factors in a way that continues to achieve desired health benefits/outcomes. [244,247] Finally, additional outcomes like economic impacts (e.g., cost-effectiveness, cost-benefit, return on investment) and unintended effects on patients, provider behaviors, and use of resources need to be measured and reported [242,249]. These factors can increase the potential for determining the generalizability and scalability of PSE interventions and facilitate decision making for enhancing patient care [250].

Identified strengths of the review include a rigorous protocol, broad search criteria for a variety of nutrition-focused PSE interventions, assessment of study quality, and a focus on clinical settings for tailored gaps. A limitation is that some populations that have risk factors associated with childhood obesity (e.g., gestational diabetes) or accelerated catch-up growth (e.g., very low birth weight or preterm births) were excluded, due to a variety of approaches to address high-risk population disease etiology. Additionally, classification of socially disadvantaged groups was limited to reported individual demographic variables (e.g., socioeconomic factors), rather than community or environmental factors known to influence health and risk for obesity, and thus the potential mechanism through which PSE approaches may impact health behavior. Another limitation is that the variation in reporting and measurement of outcomes limited the ability to make conclusions regarding effect and grading the certainty of the evidence. In fact, inconsistencies in primary or secondary outcomes, measurement tools, and timepoints of measurement continue to limit more conclusive statements regarding the effectiveness of interventions during this period of life [28,29,46,[251], [252], [253]]. Recently, core outcomes have been suggested for nutrition-related obesity prevention trials in pregnancy (e.g., preterm delivery, small or large for gestational age) [254] and early childhood (e.g., duration breastfeeding, timing of introduction of solid foods, BMI z-score) [[255], [256], [257]]. Consistently measuring and reporting these core outcomes in future studies will allow for more comprehensive assessment and synthesis of effectiveness.

Additionally, studies reporting only on assessments of children aged after 24 mo were excluded, which limited the comprehensive assessment of maintenance. Finally, this review was limited to nutrition-focused PSE approaches. Nutrition-sensitive interventions, interventions for which nutrition is not the primary aim but that influence underlying determinants of nutrition (e.g., paid family leave, expanded income eligibility for health insurance), may also have a significant influence on nutritional exposures and obesity prevention during the first 1000 d [258]. This presents an opportunity for future research to synthesize the impact of nutrition-sensitive interventions on obesity prevention interventions during the first 1000 d.

In conclusion, the available evidence indicates clinical settings are critical for nutrition and obesity prevention during the first 1000 d and highlights several gaps to realize the potential of PSE approaches to equitably promote healthy growth. First, interventions need to reach more diverse patient populations (e.g., rural and indigenous populations). Second, continuity of care within (e.g., primary care) and/or across care settings (e.g., prenatal and primary care or clinical-community) could support evolving nutrition-related needs. Third, clinical interventions to address food insecurity are an emerging area that requires evaluation. Finally, decision-making about whether or how to scale implementation of PSE approaches will be improved through more consistent reporting of the representativeness of patient groups and the clinical settings in which interventions are conducted, the fidelity with which they are implemented, and the economic and sustained impacts of such interventions. Continuing to develop and improve interventions for clinical settings that incorporate strategies further upstream, like PSE approaches, provides great public health potential for supporting healthy nutrition and growth for all.

## Author contributions

The authors’ responsibilities were as follows – CTL, CLK, MJF, KMJ, CR, CDN: designed research; CTL, CLK, MJF, KJM, EK, LJR, BEW, JC, KES, CDN: conducted research; CTL: analyzed data, wrote article, and primary responsibility for final content; CLK, MJF, KJM, EK, LJR, BEW, JC, KES, CR, CDN: critically reviewed and revised the manuscript; and all authors: read and approved the final manuscript.

## Data availability

Data described in the manuscript, codebook, and analytic code will be made available on request, pending application and approval.

## Funding

This review was supported by Healthy Eating Research, a national program of the Robert Wood Johnson Foundation. Additionally, Drs. Kracht and Neshteruk received support from the National Institutes of Health [grant numbers: K99HD107158, 5K12HL138030-05]. The content is solely the responsibility of the authors and does not necessarily represent official views of the National Institutes of Health.

## Conflict of interest

CTL reports financial support was provided by Robert Wood Johnson Foundation. CLK reports financial support was provided by National Institutes of Health. CDN reports financial support was provided by National Institutes of Health.
