# Supplementary material for: Upstream Interventions in Clinical Settings Focused on Nutrition to Prevent Obesity During the First 1000 Days: A Scoping Review
Source: Adv Nutr. 2025 Oct 9;16(12):100529. doi: 10.1016/j.advnut.2025.100529 (PMC12637399; doi:10.1016/j.advnut.2025.100529)
Supplement: Multimedia component 1 [file mmc1.pdf]

Supplementary Data

Upstream interventions in clinical settings focused on nutrition to prevent obesity during the first 1,000 days: A scoping review

Courtney T. Luecking, PhD, MPH, RDN,[1] Chelsea L. Kracht, PhD,[2,3] Mackenzie J. Ferrante, PhD, RDN,[4,5] Kameron J. Moding, PhD,[6] Elizabeth Kielb, MS,[6] Laura J. Rolke, PhD,[7,8] Brooke E. Wagner, PhD,[7,8] Jordan Colella,[1] Katherine E. Speirs, PhD,[9] Cayla Robinson, MSLS,[10] Cody D. Neshteruk, PhD[7,8]

Affiliations:
[1]Department of Dietetics and Human Nutrition, Martin-Gatton College of Agriculture, Food and Environment, University of Kentucky, Lexington, KY
[2]Pennington Biomedical Research Center, 6400 Perkins Road, Baton Rouge, LA, 70808
[3]University of Kansas Medical Center, 3901 Rainbow Blvd, Kansas City, KS 66160
[4]Department of Pediatrics, Jacobs School of Medicine and Biomedical Sciences, University at Buffalo, Buffalo, NY.
[5]Department of Nutritional Sciences, School of Environmental and Biological Sciences, Rutgers University, New Brunswick, NJ
[6]Department of Human Development and Family Science, Purdue University, West Lafayette, IN
[7]Department of Population Health Sciences, Duke University School of Medicine, Durham, NC
[8]Duke Center for Childhood Obesity Research, Duke University School of Medicine, Durham, NC
[9]Human Development and Family Science, John and Doris Norton School of Human Ecology, University of Arizona, Tucson, AZ
[10] Libraries, University of Kentucky, Lexington, KY

Supplemental Table 1. PubMed search string
Supplemental Table 2. Eligibility criteria
Supplemental Table 3. Exclusion reasons per report
Supplemental Table 4. Summary of RE-AIM dimensions of included studies by setting (n=126)
Supplemental Table 5. Downs and Black study quality rating checklist for included studies[a]
Supplemental Table 6. Funding sources for included articles

Supplementary Data

Supplemental Table 1. PubMed search string

This search string was subsequently translated into other strings appropriate Web of Science Core Collection and CAB Abstracts, CINAHL with Full Text, Agricola, Cochrane Database of Systematic Reviews, ProQuest's Dissertations and Theses, Embase, and Google Scholar<br><br>("1000 days"[tiab] OR "thousand days"[tiab] OR Infant[Mesh:NoExp] OR Infant*[tiab] OR Newborn*[tiab] OR "New born*"[tiab] OR Neonat*[tiab] OR Baby[tiab] OR Babies[tiab] OR "Infant, Newborn"[Mesh:NoExp] OR Toddler*[tiab] OR Fetus[Mesh:NoExp] OR Fetus*[tiab] OR Foetus*[tiab] OR Foetal*[tiab] OR Fetal*[tiab] OR "Pregnancy"[Mesh:NoExp] OR Pregnan*[tiab] OR Gestation*[tiab] OR Prenatal*[tiab] OR Perinatal*[tiab] OR Postnatal*[tiab] OR Conception*[tiab] OR Antepartum[tiab] OR "Ante partum"[tiab] OR Intrapartum[tiab] OR "intra partum"[tiab] OR postpartum[tiab] OR "post partum"[tiab] OR "24 months"[tiab] OR Mother[Mesh:NoExp] OR Mother*[tiab] OR mom[tiab] OR moms[tiab])<br><br>AND<br>(Overnutrition[Mesh] OR Overnutrition*[tiab] OR Hypernutrition*[tiab] OR Overeating[tiab] OR Overfeeding*[tiab] OR "hyper nutrition*"[tiab] OR "excessive feeding"[tiab] OR "over nutrition*"[tiab] OR BMI[tiab] OR "body mass index*"[tiab] OR "Body Fat"[tiab] OR "Overweight"[Mesh] OR Overweight*[tiab] OR Adipos*[tiab] OR Corpulenc*[tiab] OR Obesity[Mesh] OR Obes*[tiab] OR "Obesity, Maternal"[Mesh] OR "Obesity, Abdominal"[Mesh] OR "Obesity, Morbid"[Mesh] OR "Pediatric Obesity"[Mesh] OR "Weight gain*"[tiab] OR "Gestational Weight Gain"[Mesh] OR "Weight Gain"[Mesh:NoExp])<br><br>AND<br>(("Health Policy"[Mesh:NoExp] OR PSE[tiab] OR "Healthy People Programs"[Mesh] OR "healthy people*"[tiab] OR "Social Welfare"[Mesh:NoExp] OR Welfare*[tiab] OR "Community Service*"[tiab] OR "Child Welfare"[Mesh:NoExp] OR "Infant Welfare"[Mesh] OR "Maternal Welfare"[Mesh] OR "maternal child health"[tiab] OR "maternal health"[tiab] OR "Relief Work"[Mesh] OR "relief work*"[tiab] OR "Food Assistance*"[tiab] OR "Food Aid*"[tiab] OR "Food Stamp*"[tiab] OR "nutrition assistance*"[tiab] OR "government assistance"[tiab] OR "governmental assistance"[tiab] OR "SNAP*"[tiab] OR "Supplemental Nutrition Assistance Program"[tiab] OR "Head Start*"[tiab] OR "WIC"[tiab] OR "EFNEP"[tiab] OR "Expanded Food and Nutrition Education Program"[tiab] OR "CACFP"[tiab] OR "Child and Adult Care Food Program"[tiab] OR "NAPSACC"[tiab] OR "Nutrition and Physical Activity Self-Assessment for Child Care"[tiab] OR "FSNEP"[tiab] OR "Food Stamp Nutrition Education Program"[tiab] OR "SHCP"[tiab] OR "Shaping Healthy Choices Program"[tiab] OR "DHC"[tiab] OR "NEP"[tiab] OR "Nutrition Education Program"[tiab] OR "Healthy Hunger-Free Kids Act"[tiab] OR "Access to Healthy Foods"[Mesh]) OR (("Diet, Food, and Nutrition"[Mesh:NoExp] OR "Beverages"[Mesh] OR Beverage*[tiab] OR Soda*[tiab] OR Milk*[tiab] OR Tea[tiab] OR "Food"[Mesh] OR Food*[tiab] OR Bread*[tiab] OR Fruit*[tiab] OR Vegetable*[tiab] OR Dairy*[tiab] OR Eggs[tiab] OR Sugar*[tiab] OR Fat[tiab] OR Fats[tiab] OR Fiber*[tiab] OR Protein*[tiab] OR Meal*[tiab] OR Snack*[tiab] OR Meat*[tiab] OR Poultr*[tiab] OR Beef*[tiab] OR Grain*[tiab] OR Breakfast[tiab] OR Lunch[tiab] OR Dinner[tiab] OR supper[tiab] OR Legume*[tiab] OR "Eating"[Mesh:NoExp] OR Eat[tiab] OR Eating[tiab] OR Consumption*[tiab] OR "Drinking"[Mesh] OR Drink*[tiab] OR Water*[tiab] OR nutriti*[tiab] OR Appetite*[tiab] OR "Feeding Behavior"[Mesh:NoExp] OR Feed*[tiab] OR "Diet"[Mesh:NoExp] OR Diet*[tiab] OR "Diet, Healthy"[Mesh] OR "Appetite Regulation"[Mesh] OR "Bottle Feeding"[Mesh] OR Bottlefed*[tiab] OR Bottlefeed*[tiab] OR Formula*[tiab] OR "Breast Feeding"[Mesh] OR Breastfed*[tiab] OR "milk shar*"[tiab] OR Weaning[Mesh] OR Weaning[tiab] OR environmental*[tiab] OR system*[tiab]) AND (intervention*[tiab] OR campaign*[tiab] OR program*[tiab] OR polic*[tiab] OR incentive*[tiab] OR promotion*[tiab] OR assistance*[tiab] OR relief*[tiab])))

Supplementary Data

Supplemental Table 2. Eligibility criteria

Criteria	Inclusion	Exclusion
Full text	Available	Not available
Language	English	Non-English
Information source	Peer-reviewed literature<br>Protocol paper<br>Dissertations<br>Theses<br>Conference proceedings<br>Conference abstracts<br>Clinical trials information	White papers<br>Technical reports<br>Books<br>Websites<br>Other sources of grey literature not identified in the inclusion criteria
Study design	Quasi-experimental<br>Interrupted time series<br>Cohort<br>(Cluster) randomized controlled trial<br>Pilot/feasibility<br>Natural/ecological	Commentary<br>Review<br>Qualitative<br>Cross-sectional
Population	Humans	Animals
	Prenatal<br>Postpartum<br>Adult caring for child 24 months of age or younger	Child-bearing age, not pregnant<br>Fertility or prior to conception
	Infancy (0 – 12 months)<br>Toddlers (13 – 24 months)<br>Mean age of child participants 0 – 24 months	Majority or mean age of child participants >24 months
		Acute or chronic conditions
Intervention	Policy, systems, and/or environmental interventions that promote/support access to/availability of healthy foods and beverages, acceptability or consumption of healthy foods and beverages, positive feeding practices, or limit unhealthy foods and beverages	No intervention
	Implemented within context of overnutrition or dual burden of malnutrition (over- and under-nutrition)	Implemented within context of undernutrition
	Policy interventions involve new rules, procedures, laws, ordinances, resolutions, mandates, or regulations.<br>Policies may occur at federal, state, or local levels as well as within organizations.	Interventions focused on an individual, family, or household that do not include or are not the result of a policy, system, or environmental change<br>E.g., counseling, motivational interviewing, nutrition education
	Systems interventions involve changes made to rules, infrastructure, processes, or procedures within an organization or between organizations, systems, or sectors.<br>Systems change may occur within an organization, sector, or system.	Supplements or supplemental feeding; parental nutrition, formula compositions, micronutrient or macronutrient supplementation, fortified foods
	Environmental interventions change the physical (structural changes or programs or service), social (attitude or action), and/or economic factors (financial incentives or disincentives) that influence nutrition-related practices and behaviors.	Research team serves as interventionist/implementing the change<br>Recruitment without an embedded intervention (e.g., recruiting from WIC and doing a program elsewhere)
Outcome(s)	Food insecurity	Malnutrition (wasting, stunting)
	Acceptability or consumption of foods and beverages (healthy or unhealthy), diet quality, eating patterns, food groups	Nutrient content
	Breastfeeding	Food purchases
	Timing of introduction of complementary/solid foods	Anything other than what is listed in the inclusion criteria
	Feeding practices or approaches	
	Weight, adiposity, body composition, growth trajectory	

Supplementary Data

Setting	Clinical (hospital, clinics, primary care)	Non-clinical (home visiting programs, WIC, community, early care and education)

Supplementary Data

Supplemental Table 3. Exclusion reasons per report

Number	Full Citation	Exclusion Reason
1	Actrn. Weighing In Pregnancy. http://wwwwhoint/trialsearch/Trial2aspx?TrialID=ACTRN12610000331033. 2010;	No Full Text
2	Caan BJ. The effect of WIC supplemental feeding during the interpregnancy interval on the health of the mother and her infant. 1985;(8609906):214.	No Full Text
3	Pehrsson PR, Moser-Veillon P, Sims L. Iron status and weight change in nonlactating postpartum WIC participants and nonparticipants. 1995;(9622127):201.	No Full Text
4	Saadatnia S, Soltani F, Saber A, Kazemi F. The Effect of Group Counseling Based on Health Belief Model on Nutritional Behavior of Pregnant Women with Overweight: a Randomized Controlled Trial. *Avicenna Journal of Nursing and Midwifery Care*. 2021;29:102-112. doi:10.30699/ajnmc.29.2.102	Not English Language
5	Santos MMAS, de Barros DC, Baião MR, Saunders C. Nutritional care and weight gain in pregnant adolescents: A quantitative and qualitative approach. *Cien Saude Colet*. 2013;18(3):789-802. doi:10.1590/S1413-81232013000300025	Not English Language
6	Vitolo MR, Bortolini GA, Feldens CA, de Lourdes Drachler M. Impacts of the 10 Steps to Healthy Feeding in Infants: a randomized field trial. *Cad Saude Publica*. 2005;21:1448-1457. doi:10.1590/s0102-311x2005000500018	Not English Language
7	Bradford WD. Initiation and Duration of Breastfeeding among WIC Participants: Impact of State Policies. 2014.	Not Enough Information
8	Braun MLG, Giugliani ERJ, de Mattos Soares ME, Giugliani C, de Oliveira AP, Danelon CMM. Evaluation of the Impact of the Baby-Friendly Hospital Initiative on Rates of Breastfeeding. *Am J Public Health*. 2003;93:1277-1279. doi:10.2105/ajph.93.8.1277	Not Enough Information
9	Cheung KW, Tarrant A. A randomized controlled trial of an antenatal intervention to increase exclusive breastfeeding. doi:10.5353/th_b5351044	Not Enough Information
10	D'Oria R. Mommy and Me From Birth to Three: An Infant Obesity Prevention Project. *J Obstet Gynecol Neonatal Nurs*. 2011;40:S26-S27. doi:10.1111/j.1552-6909.2011.01242_36.x	Not Enough Information
11	Frank DA, Wirtz SJ, Sorenson JR, Heeren T. Commercial discharge packs and breast-feeding counseling: effects on infant-feeding practices in a randomized trial. *Pediatrics*. 1987;80:845-854.	Not Enough Information
12	Furman L, Matthews L, Davis V, Killpack S, O'Riordan MA. Breast for Success: A Community–Academic Collaboration to Increase Breastfeeding Among High-Risk Mothers in Cleveland. *Prog Community Health Partnersh*. 2016;10:339-340. doi:10.1353/cpr.2016.0040	Not Enough Information
13	Gorman KA, Lim-Miller A, Oliver L, Lenders C. Hi-5 Way: A multidisciplinary approach to secondary prevention of obesity in children 5 and under from underserved areas. *FASEB J*. 2012;26.	Not Enough Information
14	Horodynski M, Baker S, Olson B, Brophy-Herb H, Van Egeren L, Auld G. Healthy Babies through Infant-Centered Feeding. *J Nutr Educ Behav*. 2012;44:S81-S81. doi:10.1016/j.jneb.2012.03.194	Not Enough Information

15	Kyttälä P, Kautiainen S, Kronberg-Kippilä C, et al. Pilot intervention study on diet of toddlers in Finland. *Obes Rev*. 2010;11:464. doi:10.1111/j.1467-789X.2010.00763-7.x	Not Enough Information
16	Middleton P, Stuart-Butler D, Deverix J, Glover K. Mother and baby bundls: Improving aboriginal women's and infants' nutrition. *J Pediatr Child Health*. 2017;53:68. doi:10.1111/jpc.13494_200	Not Enough Information
17	Taylor SN, Barreira J, Murphy P, et al. Multidisciplinary improvements of hospital lactation support process: Impact on mothers' milk delivery to infants. *Breastfeed Med*. 2011;6:S20. doi:10.1089/bfm.2011.9985	Not Enough Information
18	Trendafilova P. Monitoring of pregnant Roma women–an effective policy intervention. *European Journal of Public Health*. 2018;28(suppl_4):cky214. 277.	Not Enough Information
19	Webber S, Butteris S, Coller R, Coller K. Promotoras de centro: A community based approach to improving health disparities in a local global population. *Pediatrics*. 2018;142(1)doi:10.1542/peds.142.1_MeetingAbstract.515	Not Enough Information
20	Werr J, Sydhoff J, Landström S, Forsberg B. Childhood Obesity Prevention program for disadvantaged minorities in Sweden highlights both challenges and successes of an innovative integrated care model. *Int J Integr Care*. 2017;17:1-2. doi:10.5334/ijic.3774	Not Enough Information
21	Abstracts from the Academy of Breastfeeding Medicine 19th Annual International Meeting. *Breastfeed Med*. 2014;9.	Wrong Information Source
22	Ahc M. Family Spirit Nurture Intervention Study Shows Benefits for the Little Ones: Coaches help problem-solve. *Case Management Advisor*. 2021;32(2):1-3.	Wrong Information Source
23	Bell LM. Can Anticipatory Guidance About Infant Diet Help Prevent Obesity. *NEJM Journal Watch.* 2012;2012	Wrong Information Source
24	Butte NF, Cobb K, Dwyer JT, Graney L, Heird WC, Rickard KA. The Start Healthy Feeding Guidelines for Infants and Toddlers. *J Am Diet Assoc*. 2004;104:442-454. doi:10.1016/j.jada.2004.01.027	Wrong Information Source
25	New Research Supports Use of a Prenatal Case Management-Style Intervention. *Hosp Case Manag*. 2022;30(1):1-3.	Wrong Information Source
26	Rasmussen KM, Latulippe ME, Yaktine AL, Board N. Promotion, Motivation, and Support of Breastfeeding with the WIC Food Packages. 2016.	Wrong Information Source
27	Zhang J, Hansen AR. Special Supplemental Nutrition Program for Women, Infants, and Children Program, a Social Experiment on Effective Strategies to Prevent Severe Obesity Among Preschool Children. *JAMA Pediatr*. 2018;172(7):704. doi:10.1001/jamapediatrics.2018.1019	Wrong Information Source
28	Abdulahi M, Fretheim A, Argaw A, Magnus JH. Breastfeeding Education and Support to Improve Early Initiation and Exclusive Breastfeeding Practices and Infant Growth: A Cluster Randomized Controlled Trial from a Rural Ethiopian Setting. *Nutrients*. 2021;13:1204-undefined. doi:10.3390/nu13041204	Wrong Intervention
29	Abdulahi M, Fretheim A, Magnus JH. Effect of breastfeeding education and support intervention (BFESI) versus routine care on timely initiation and exclusive breastfeeding in Southwest Ethiopia: study protocol for a cluster randomized controlled trial. *BMC Pediatr*. 2018;18:14. doi:10.1186/s12887-018-1278-5	Wrong Intervention

Supplementary Data

30	Aboud FE, Sadika A. A cluster-randomized evaluation of a responsive stimulation and feeding intervention in Bangladesh. *Pediatrics*. 2011;127(5):e1191-e1197. doi:10.1542/peds.2010-2160	Wrong Intervention
31	Actrn. Early intervention of multiple home visits to prevent childhood obesity among a disadvantaged population: a home-based Randomised Controlled Trial. https://trialsearchwhoint/Trial2aspx?TrialID=ACTRN12607000168459. 2007;	Wrong Intervention
32	Actrn. Learning Clubs to improve women's perinatal health and early childhood development. https://trialsearchwhoint/Trial2aspx?TrialID=ACTRN12617000442303. 2017;	Wrong Intervention
33	Actrn. Water Quality and the Microbiome Study - TUMS. https://trialsearchwhoint/Trial2aspx?TrialID=ACTRN12619000458134. 2019;	Wrong Intervention
34	Ahmed AH, Roumani AM, Szucs K, Zhang LS, King D. The Effect of Interactive Web-Based Monitoring on Breastfeeding Exclusivity, Intensity, and Duration in Healthy, Term Infants After Hospital Discharge. *J Obstet Gynecol Neonatal Nurs*. 2016;45(2):143-154. doi:10.1016/j.jogn.2015.12.001	Wrong Intervention
35	Aidam B, Pérez-Escamilla R, Lartey A. Lactation Counseling Increases Exclusive Breast-Feeding Rates in Ghana. *J Nutr*. 2005;135:1691-1695. doi:10.1093/jn/135.7.1691	Wrong Intervention
36	Ainscough K, Kennelly MA, O'Sullivan EJ, et al. 1014: Impact of a smartphone app supporting a lifestyle intervention in overweight and obese pregnancy on on maternal health and lifestyle outcomes. *Am J of Obstet Gynecol*. 2018;218:S598-S599. doi:10.1016/j.ajog.2017.11.551	Wrong Intervention
37	Ainscough KM, O'Brien EC, Lindsay KL, et al. Nutrition, Behavior Change and Physical Activity Outcomes From the PEARS RCT-An mHealth-Supported, Lifestyle Intervention Among Pregnant Women With Overweight and Obesity. *Front Endocrinol (Lausanne)*. 2019;10:938. doi:10.3389/fendo.2019.00938	Wrong Intervention
38	Aksu H, Küçük M, Düzgün G. The effect of postnatal breastfeeding education/support offered at home 3 days after delivery on breastfeeding duration and knowledge: a randomized trial. *J Matern Fetal Neonatal Med*. 2010;24:354-361. doi:10.3109/14767058.2010.497569	Wrong Intervention
39	Anderson CE, O'Malley K, Martinez CE, Ritchie LD, Whaley SE. Longer Family Participation in WIC is Associated With Lower Childhood Sugar-Sweetened Beverage Intake. *J Nutr Educ Behav*. 2022;54(3):239-248. doi:10.1016/j.jneb.2021.10.003	Wrong Intervention
40	Assis AM, Costa PR, Silva MC, et al. Effectiveness of the Brazilian Conditional Cash Transfer Program--Bolsa Alimentação--on the variation of linear and ponderal increment in children from northeast of Brazil. *Nutr Hosp*. 2014;31:689-697.	Wrong Intervention
41	Banna J, Campos M, Gibby C, et al. Multi-site trial using short mobile messages (SMS) to improve infant weight in low-income minorities: Development, implementation, lessons learned and future applications. *Contemp Clin Trials*. 2017;62:56-60. doi:10.1016/j.cct.2017.08.011	Wrong Intervention
42	Barber SL, Gertler P. The impact of Mexico's conditional cash transfer programme, Oportunidades, on birthweight. *Trop Med Int Health*. 2008;13:1405-1414. doi:10.1111/j.1365-3156.2008.02157.x	Wrong Intervention
43	Baur L. Effectiveness of a home-based early intervention on children's BMI at age two years: Randomised controlled trial. *Obes Facts*. 2012;5:34. doi:10.1159/000188329	Wrong Intervention

44	Baur LA, Askie LM, Rissel C, et al. The early prevention of obesity in children (EPOCH) collaboration-results of an individual participant data prospective meta-analysis. *Obes Facts*. 2015;8:5. doi:10.1159/000382140	Wrong Intervention
45	Berry D, Verbiest S, Hall EG, et al. A Postpartum Community-Based Weight Management Intervention Designed for Low-Income Women: Feasibility and Initial Efficacy Testing. *J Natl Black Nurses Assoc*. 2015;26(1):29-39.	Wrong Intervention
46	Bich TH, Hoa DTP, Målqvist M. Fathers as Supporters for Improved Exclusive Breastfeeding in Viet Nam. *Matern Child Health J*. 2013;18:1444-1453. doi:10.1007/s10995-013-1384-9	Wrong Intervention
47	Bick D, Taylor C, Avery A, et al. Protocol for a two-arm feasibility RCT to support postnatal maternal weight management and positive lifestyle behaviour in women from an ethnically diverse inner city population: the SWAN feasibility trial. *Pilot Feasibility Stud*. 2019;5(1)doi:10.1186/s40814-019-0497-3	Wrong Intervention
48	Bick D, Taylor C, Seed P, et al. Supporting women with postnatal weight management: The SWAN feasibility randomised controlled trial. *BJOG*. 2019;126:33. doi:10.1111/1471-0528.15633	Wrong Intervention
49	Bogaerts A, Devlieger R, Nuyts E, Witters I, Gyselaers W, Van den Bergh BRH. Effects of lifestyle intervention in obese pregnant women on gestational weight gain and mental health: a randomized controlled trial. *Int J Obes (Lond)*. 2012;37:814-821. doi:10.1038/ijo.2012.162	Wrong Intervention
50	Bonuck K, Trombley M, Freeman K, McKee D. Randomized, controlled trial of a prenatal and postnatal lactation consultant intervention on duration and intensity of breastfeeding up to 12 months. *Pediatrics*. 2005;116:1413-1426. doi:10.1542/peds.2005-0435	Wrong Intervention
51	Boonrusmee S, Cheamsanit S. Telephone-based counselling helped parents to wean children off bottles at 2 years of age. *Acta paediatr* (Oslo, Norway : 1992). 2020;110:718-719. doi:10.1111/apa.15550	Wrong Intervention
52	Bryant M, Burton W, Cundill B, et al. Effectiveness of an implementation optimisation intervention aimed at increasing parent engagement in HENRY, a childhood obesity prevention programme - the Optimising Family Engagement in HENRY (OFTEN) trial: study protocol for a randomised controlled trial. *Trials*. 2017;18(1):40. doi:10.1186/s13063-016-1732-3	Wrong Intervention
53	Bueno-Gutierrez D, Castillo EUR, Mondragón AEH. Breastfeeding counseling based on formative research at primary healthcare Services in Mexico. *Int J Equity Health*. 2021;20:173-173. doi:10.1186/s12939-021-01491-6	Wrong Intervention
54	Bull ER, Clayton H, Hendry T. Bump Start: developing and piloting a healthy living group intervention for obese pregnant women. *Br J Midwifery*. 2017;25(6):386-395. doi:10.12968/bjom.2017.25.6.386	Wrong Intervention
55	Cameron SL, Gray AR, Taylor RW, et al. Excessive growth from 6 to 24 months of age: Results from the prevention of overweight in infancy (POI) randomised controlled trial. *Arch Dis Child*. 2014;99:A109. doi:10.1136/archdischild-2014-307384.290	Wrong Intervention

56	Campbell KJ, Hesketh KD, McNaughton SA, et al. The extended Infant Feeding, Activity and Nutrition Trial (InFANT Extend) Program: a cluster-randomized controlled trial of an early intervention to prevent childhood obesity. *BMC Public Health*. 2016;16:166. doi:10.1186/s12889-016-2836-0	Wrong Intervention
57	Campos M, Pomeroy J, Mays MH, Lopez A, Palacios C. Intervention to promote physical activation and improve sleep and response feeding in infants for preventing obesity early in life, the baby-act trial: Rationale and design. *Contemp Clin Trials*. 2020;99:106185. doi:10.1016/j.cct.2020.106185	Wrong Intervention
58	Carbonneau E, Dumas A, Brodeur-Doucet A, Fontaine-Bisson B. A Community Prenatal Intervention in Social Nutrition: Evaluating the Impact on Pregnancy and Birthweight Outcomes. *Nutrients*. 2022;14(6)doi:10.3390/nu14061151	Wrong Intervention
59	Cepni AB, Taylor A, Crumbley C, et al. Feasibility and Efficacy of the "FUNPALs Playgroup" Intervention to Improve Toddler Dietary and Activity Behaviors: A Pilot Randomized Controlled Trial. *Int J Environ Res Public Health*. 2021;18(15)doi:10.3390/ijerph18157828	Wrong Intervention
60	Cilenti D, Hye-Chung K, Wells R, Whitmire JT, Goyal RK, Hillemeier MM. Changes in North Carolina Maternal Health Service Use and Outcomes Among Medicaid-Enrolled Pregnant Women During State Budget Cuts. *J Public Health Manag Pract*. 2015;21(2):208-213. doi:10.1097/PHH.0000000000000118	Wrong Intervention
61	Collin DF, Pulvera R, Hamad R. The effect of the 2009 revised U.S. guidelines for gestational weight gain on maternal and infant health: a quasi-experimental study. *BMC Pregnancy Childbirth*. 2023;23:118-undefined. doi:10.1186/s12884-023-05425-8	Wrong Intervention
62	Collins TR, DeMellier ST, Leeper JD, Milo T. Supplemental Food Program: effects on health and pregnancy outcome. *South Med J*. 1985;78(5):551-5. doi:10.1097/00007611-198505000-00014	Wrong Intervention
63	Covington DL, Peoples-Sheps M. "Baby Talk" adolescent prenatal education program: An evaluation of its impact on birth outcome. 1996;(9841021):203.	Wrong Intervention
64	Covington DL, Peoples-Sheps MD, Buescher PA, Bennett TA, Paul MV. An evaluation of an adolescent prenatal education program. *Am J Health Behav*. 1998;22(5):323-333.	Wrong Intervention
65	Cramp C, Deussen AR, Dodd JM, Louise J, Grivell RM, Moran LJ. Evaluation of a smartphone nutrition and physical activity application to provide lifestyle advice to pregnant women: The SNAPP randomised trial. *Matern Child Nutr*. 2018;14(1):n/a-1. doi:10.1111/mcn.12502	Wrong Intervention
66	Crovetto F, Crispi F, Borras R, et al. OC06.07: Mediterranean diet or stress reduction program during pregnancy for preventing SGA newborns according to prepregnancy BMI: the IMPACT-BCN trial. *Ultrasound in Obstetrics & Gynecology*. 2022;60:19-19. doi:10.1002/uog.25034	Wrong Intervention
67	Cuevas-Nasu L, Shamah-Levy T, Gaona-Pineda E, Humaran IMG, Avila-Arcos M. Malnutrition and food insecurity in beneficiary households from food assistance programs in Slp, Mexico. *FASEB J*. 2015;29(1).	Wrong Intervention
68	Daley A, Jolly K, Ives N, et al. Practice nurse-supported weight self-management delivered within the national child immunisation programme for postnatal women: a feasibility cluster RCT. *Health Technol Assess*. 2021;25:1-130. doi:10.3310/hta25490	Wrong Intervention

69	Dalrymple K, Patel N, Briley A, et al. The effects of feeding practices and appetitive traits on infant anthropometry in 6-month infants born to obese women-a secondary analysis of the UPBEAT trial. *Reproductive Sciences*. 2018;25(1):268A.	Wrong Intervention
70	Daniels L, Heath A-LM, Williams SM, et al. Baby-Led Introduction to SolidS (BLISS) study: a randomised controlled trial of a baby-led approach to complementary feeding. *BMC Pediatr*. 2015;15:1-15. doi:10.1186/s12887-015-0491-8	Wrong Intervention
71	Daniels L, Mallan K, Jansen E, Nicholson J, Magarey A. Fathers - the forgotten parent: Comparison of early feeding practices in mother-father dyads and indirect impact of the efficacious NOURISH maternal intervention on fathers' feeding practices. *Obes Facts*. 2018;11:208-209. doi:10.1159/000489691	Wrong Intervention
72	Daniels L, Mallan K, Nicholson J, Meedeniya J, Magarey A. Child behaviour and weight outcomes of NOURISH RCT. *Obes Facts*. 2013;6:16.	Wrong Intervention
73	Daniels LA, Magarey AM, Nicholson JM. The NOURISH early feeding trial: An innovative approach to child obesity prevention. *Obes Res Clin Pract*. 2011;5:S5. doi:10.1016/j.orcp.2011.08.060	Wrong Intervention
74	Daniels LA, Mallan KM, Battistutta D, et al. Child eating behavior outcomes of an early feeding intervention to reduce risk indicators for child obesity: the NOURISH RCT. *Obesity (Silver Spring)*. 2014;22(5):E104-11. doi:10.1002/oby.20693	Wrong Intervention
75	Daniels LA, Mallan KM, Battistutta D, Nicholson JM, Perry R, Magarey A. Evaluation of an intervention to promote protective infant feeding practices to prevent childhood obesity: outcomes of the NOURISH RCT at 14 months of age and 6 months post the first of two intervention modules. *Int J Obes (Lond)*. 2012;36(10):1292-8. doi:10.1038/ijo.2012.96	Wrong Intervention
76	Daniels LA, Mallan KM, Nicholson JM, Battistutta D, Magarey A. Outcomes of an early feeding practices intervention to prevent childhood obesity. *Pediatrics*. 2013;132(1):e109-18. doi:10.1542/peds.2012-2882	Wrong Intervention
77	Daniels LA. Complementary Feeding in an Obesogenic Environment: Behavioral and Dietary Quality Outcomes and Interventions. *Nestle Nutr Inst Workshop Ser*. 2017;87:167-181. doi:10.1159/000449213	Wrong Intervention
78	Denney-Wilson E, Campbell KJ, Laws R, et al. Assisting parents to prevent obesity in their young children: The COMPaRE-PHC early childhood research program. *Obes Res Clin Pract*. 2013;7:e98. doi:10.1016/j.orcp.2013.12.678	Wrong Intervention
79	Denney-Wilson E, Laws R, Taki S, et al. Growing healthy: A week-by-week, m-health intervention for parents of infants 0-9 months. *Obes Res Clin Pract*. 2014;8:25. doi:org/10.1016/j.orcp.2014.10.044	Wrong Intervention
80	Desjardins E, Hardwick D, Desjardins E, Hardwick D. How many visits by health professionals are needed to make a difference in low birthweight? A dose-response study of the Toronto Healthiest Babies Possible program. *Can J Public Health*. 1999;90(4):224-228.	Wrong Intervention
81	Dodd JM, Louise J, Cramp C, Grivell RM, Moran LJ, Deussen AR. Evaluation of a smartphone nutrition and physical activity application to provide lifestyle advice to pregnant women: The SNAPP randomised trial. *Matern Child Nutr*. 2017;14:e12502-undefined. doi:10.1111/mcn.12502	Wrong Intervention

82	Dong YW, Rivera DE, Downs DS, Savage JS, Collins LM. Theory of planned behavior with self-regulation dynamical systems model for an adaptive intervention to manage gestational weight gain. *Ann Behav Med*. 2014;47:S148-S148.	Wrong Intervention
83	Doorley E, Young C, O'shea B, Darker C, Hollywood B, O'rorke C. Is primary prevention of childhood obesity by education at 13-month immunisations feasible and acceptable? Results from a general practice based pilot study. *Ir Med J*. 2015;108(1).	Wrong Intervention
84	Edozien JC, Switzer BR, Bryan RB. Medical evaluation of the special supplemental food program for women, infants, and children. *Am J Clin Nutr*. 1979;32(3):677-92. doi:10.1093/ajcn/32.3.677	Wrong Intervention
85	El-Sayed H, Martines J, Rakha M, Zekry O, Abdel-Hak M, Abbas H. The effectiveness of the WHO training course on complementary feeding counseling in a primary care setting, Ismailia, Egypt. *J Egypt Public Health Assoc*. 2014;89(1):1-8. doi:10.1097/01.EPX.0000443990.46047.a6	Wrong Intervention
86	Ettinger de Cuba S, Mbamalu M, Bovell-Ammon A, et al. Prenatal WIC Is Associated with Increased Birth Weight of Infants Born in the United States with Immigrant Mothers. *J Acad Nutr Diet*. 2022;122:1514-1524.e4. doi:10.1016/j.jand.2022.02.005	Wrong Intervention
87	Fahey MC, Wayne Talcott G, Cox Bauer CM, et al. Moms fit 2 fight: Rationale, design, and analysis plan of a behavioral weight management intervention for pregnant and postpartum women in the U.S. military. *Contemp Clin Trials*. 2018;74:46-54. doi:10.1016/j.cct.2018.09.012	Wrong Intervention
88	Fajfrová J, Pavlík V, Vosečková A, et al. Program of body weight reduction for women after childbirth. *Obes Rev*. 2010;11:402. doi:10.1111/j.1467-789X.2010.00763-7.x	Wrong Intervention
89	Fewtrell M, Kennedy K, Lukoyanova O, et al. Short-term efficacy of two breast pumps and impact on breastfeeding outcomes at 6 months in exclusively breastfeeding mothers: A randomised trial. *Matern Child Nutr*. 2019;15:e12779-undefined. doi:10.1111/mcn.12779	Wrong Intervention
90	Flanagan EW, Altazan AD, Comardelle NR, et al. The Design of a Randomized Clinical Trial to Evaluate a Pragmatic and Scalable eHealth Intervention for the Management of Gestational Weight Gain in Low-Income Women: Protocol for the SmartMoms in WIC Trial. *JMIR Res Protoc*. 2020;9(9):e18211. doi:10.2196/18211	Wrong Intervention
91	Flynn AC, Schneeberger C, Seed PT, Barr S, Poston L, Goff LM. The Effects of the UK Pregnancies Better Eating and Activity Trial Intervention on Dietary Patterns in Obese Pregnant Women Participating in a Pilot Randomized Controlled Trial. *Nutr Metab Insights*. 2015;8(Suppl 1):79-86. doi:10.4137/nmi.S29529	Wrong Intervention
92	Frith AL, Naved RT, Persson L, Rasmussen KM, Frongillo EA. Early Participation in a Prenatal Food Supplementation Program Ameliorates the Negative Association of Food Insecurity with Quality of Maternal-Infant Interaction. *J Nutr*. 2012;142:1095-1101. doi:10.3945/jn.111.155358	Wrong Intervention
93	Frongillo EA, Nguyen PH, Sanghvi T, et al. Nutrition interventions integrated into an existing maternal, neonatal, and child health program reduce food insecurity among recently delivered and pregnant women in Bangladesh. *J Nutr*. 2019;149(1):159-166. doi:10.1093/jn/nxy249	Wrong Intervention
94	Gagnon AJ, Dougherty G, Jimenez V, Leduc N. Randomized trial of postpartum care after hospital discharge. *Pediatrics*. 2002;109:1074-1080. doi:10.1542/peds.109.6.1074	Wrong Intervention

95	Galland B, Taylor B, Gray A, et al. Early life prevention of obesity by targeting sleep, or food and activity: a randomized controlled trial. *Sleep*. 2016;39:A339-A340.	Wrong Intervention
96	Germeroth LJ, Benno MT, Kolko Conlon RP, et al. Trial design and methodology for a non-restricted sequential multiple assignment randomized trial to evaluate combinations of perinatal interventions to optimize women's health. *Contemp Clin Trials*. 2019;79:111-121. doi:10.1016/j.cct.2019.03.002	Wrong Intervention
97	Gertner G, Johannsen J, Martinez S. Effects of Nutrition Promotion on Child Growth in El Alto, Bolivia: Results from a Geographical Discontinuity Design. *Economia-Journal of the Latin American and Caribbean Economic Association*. 2016;17(1):131-165.	Wrong Intervention
98	Gesell SB, Katula JA, Strickland C, Vitolins MZ. Feasibility and Initial Efficacy Evaluation of a Community-Based Cognitive-Behavioral Lifestyle Intervention to Prevent Excessive Weight Gain During Pregnancy in Latina Women. *Matern Child Health J*. 2015;19(8):1842-1852. doi:10.1007/s10995-015-1698-x	Wrong Intervention
99	Ghodsi D, Omidvar N, Eini-Zinab H, Rashidian A, Raghfar H. Impact of the national food supplementary program for children on household food security and maternal weight status in Iran. *Int J Prev Med*. 2016;7doi:10.4103/2008-7802.190605	Wrong Intervention
100	Globus I, Latzer Y, Pshetatzki O, et al. Effects of early parent training on Mother-Infant Feeding Interactions. *J Dev Behav Pediatr*. 2019;40(2):131-138. doi:10.1097/dbp.0000000000000625	Wrong Intervention
101	Glor ED. Impacts of a prenatal program for native women. *Can J Public Health*. 1987;78(4):249-254.	Wrong Intervention
102	Guldan GS, Fan HC, Ma X, Ni ZZ, Xiang X, Tang MZ. Culturally Appropriate Nutrition Education Improves Infant Feeding and Growth in Rural Sichuan, China. *J Nutr*. 2000;130:1204-1211. doi:10.1093/jn/130.5.1204	Wrong Intervention
103	Haider R. Impact of peer counsellors on breastfeeding practices in dhaka, bangladesh. 1998;(U112345):1.	Wrong Intervention
104	Hails KA, Whipps MDM, Gross RS, et al. Breastfeeding and Responsive Parenting as Predictors of Infant Weight Change in the First Year. *J Pediatr Psychol*. 2021;46(7):768-778. doi:10.1093/jpepsy/jsab049	Wrong Intervention
105	Hannan J. APN telephone follow up to low-income first time mothers. *J Clin Nurs*. 2013;22(1-2):262-70. doi:10.1111/j.1365-2702.2011.04065.x	Wrong Intervention
	Hannan JL. Low-income first time mothers: Effects on advanced practice nurse (APN) follow up telephone calls on maternal and infant health and health care charges. 2010;(Ph.D.):148 p-148.	Wrong Intervention
106	Haque Rema S. A low cost, sustainable, locally delivered intervention to promote exclusive breastfeeding practices in rural Bangladeshi women. 2019;(27678953).	Wrong Intervention
107	Harden SM, Beauchamp MR, Pitts BH, et al. Group-based lifestyle sessions for gestational weight gain management: a mixed method approach. *Am J Health Behav*. 2014;38(4):560-9. doi:10.5993/ajhb.38.4.9	Wrong Intervention

108	Harris-Fry H, Paudel PR, Harrisson T, et al. Participatory Women's Groups with Cash Transfers Can Increase Dietary Diversity and Micronutrient Adequacy during Pregnancy, whereas Women's Groups with Food Transfers Can Increase Equity in Intrahousehold Energy Allocation. *J Nutr*. 2018;148:1472-1483. doi:10.1093/jn/nxy109	Wrong Intervention
109	Hedderson M, Ferrara A, Brown SD, Albright CL. Development and testing of an adaptive intervention among pregnant women with overweight or obesity: a pragmatic randomized controlled trial in an integrated health system. *Ann Behav Med*. 2020;54:S476-S476.	Wrong Intervention
110	Hernandez E, Lavner JA, Moore AM, et al. Sleep SAAF responsive parenting intervention improves mothers' feeding practices: a randomized controlled trial among African American mother-infant dyads. *Int J Behav Nutr Phys Act*. 2022;19(1):129. doi:10.1186/s12966-022-01366-1	Wrong Intervention
111	Herring SJ, Cruice JF, Bennett GG, Rose M, Davey A, Foster GD. Preventing excessive gestational weight gain among African American women: A randomized clinical trial. *Obesity (Silver Spring)*. 2015;24:30-36. doi:10.1002/oby.21240	Wrong Intervention
112	Horodynski MA, Stommel M. Nutrition education aimed at toddlers: an intervention study. *Pediatr Nurs*. 2005;31(5):364, 367-72.	Wrong Intervention
113	Horodynski MA. Healthy Babies through Infant Center Feeding: Impact of a Community Based Home Visitation Intervention to Reduce the Risk of Infant Obesity in Low Income Populations. *Nurs Outlook*. 2015;63(1):102-103. doi:10.1016/j.outlook.2014.12.007	Wrong Intervention
114	Huang TT, Yeh CY, Tsai YC. A diet and physical activity intervention for preventing weight retention among Taiwanese childbearing women: a randomised controlled trial. *Midwifery*. 2009;27:257-264. doi:10.1016/j.midw.2009.06.009	Wrong Intervention
115	Huang T-t, Yeh C-Y, Tsai Y-C. A diet and physical activity intervention for preventing weight retention among Taiwanese childbearing women: a randomised controlled trial. *Midwifery*. 2011;27(2):257-264. doi:10.1016/j.midw.2009.06.009	Wrong Intervention
116	Huda TM, Alam A, Tahsina T, et al. Shonjibon cash and counselling: a community-based cluster randomised controlled trial to measure the effectiveness of unconditional cash transfers and mobile behaviour change communications to reduce child undernutrition in rural Bangladesh. *BMC Public Health*. 2020;20(1):1-14. doi:10.1186/s12889-020-09780-5	Wrong Intervention
117	Hui AL, Back L, Ludwig S, et al. Lifestyle intervention on diet and exercise reduced excessive gestational weight gain in pregnant women under a randomised controlled trial. *BJOG*. 2011;119:70-77. doi:10.1111/j.1471-0528.2011.03184.x	Wrong Intervention
118	Huynh D, N TT, L TN, Berde Y, Y LL. Maternal milk supplementation as part of lactation support intervention improves breastfeeding performance, birth and growth outcomes. *J Matern Fetal Med*. 2016;29:60-. doi:10.1080/14767058.2016.1191212	Wrong Intervention
119	Hyczko AV, Ruggiero CF, Hohman EE, et al. Sex Differences in Maternal Restrictive Feeding Practices in the Intervention Nurses Start Infants Growing on Healthy Trajectories Study. *Acad Pediatr*. 2021;21(6):1070-1076. doi:10.1016/j.acap.2021.05.002	Wrong Intervention

120	Ickovics JR, Kershaw T, Westdahl C, et al. Group prenatal care and perinatal outcomes: a randomized controlled trial. *Obstet Gynecol*. 2007;110:330-339. doi:10.1097/01.aog.0000275284.24298.23	Wrong Intervention
121	Ingalls A, Rosenstock S, Foy Cuddy R, et al. Family Spirit Nurture (FSN) - a randomized controlled trial to prevent early childhood obesity in American Indian populations: trial rationale and study protocol. *BMC Obes*. 2019;6:18. doi:10.1186/s40608-019-0233-9	Wrong Intervention
122	Isrctn. A healthy future for young children in rural China: evaluating a community health worker program to improve maternal, newborn and child health. https://trialsearchwhoint/Trial2aspx?TrialID=ISRCTN16800789. 2021.	Wrong Intervention
123	Isrctn. Comprehensive care of infants: development and evaluation of an intervention strategy to enhance the promotion of health and quality of life of infants 2007 - 2010. https://trialsearchwhoint/Trial2aspx?TrialID=ISRCTN46535923. 2007.	Wrong Intervention
124	Isrctn. Healthy future: a community health worker program to improve maternal, newborn and child health in rural china. https://trialsearchwhoint/Trial2aspx?TrialID=ISRCTN98898991. 2019.	Wrong Intervention
125	Isrctn. Improving maternal and child health service utilization and dietary intake to enhance maternal and child health in rural areas of southern Terai, Nepal. https://trialsearchwhoint/Trial2aspx?TrialID=ISRCTN60684155. 2015.	Wrong Intervention
126	Isrctn. To evaluate the impact of attending The Lifestyle Course (TLC) on the health of pregnant women with a Body Mass Index (BMI) of 30kg/m2 or more and their babies. https://trialsearchwhoint/Trial2aspx?TrialID=ISRCTN09432573. 2011.	Wrong Intervention
127	Jancey JM, Monteiro SMDR, Dhaliwal SS, et al. Dietary outcomes of a community based intervention for mothers of young children: A randomised controlled trial. *Int J Behav Nutr Phys Act*. 2014;11:182-198. doi:10.1186/s12966-014-0120-1	Wrong Intervention
128	Jonsdottir OH, Fewtrell MS, Gunnlaugsson G, et al. Initiation of Complementary Feeding and Duration of Total Breastfeeding: Unlimited Access to Lactation Consultants Versus Routine Care at the Well-Baby Clinics. *Breastfeed Med*. 2014;9(4):196-202. doi:10.1089/bfm.2013.0094	Wrong Intervention
129	Kansiime E, Kabahenda MK, Bonsi EA. Improving caregivers' infant and young child-feeding practices using a three-group food guide: a randomized intervention study in central Uganda. *Afr J Food Agric Nutr Dev*. 2021;21(4):17834-17853. doi:10.18697/ajfand.99.20240	Wrong Intervention
130	Karim MR, Flora MS, Akhter S. Birthweight of the babies delivered by chronic energy deficient mothers in National Nutrition Program (NNP) intervention area. *Bangladesh Med Res Counc Bull*. 2011;37(1):17-23. doi:10.3329/bmrcb.v37i1.7794	Wrong Intervention
131	Karim MR, Flora MS, Akhter S. Targeted food supplementation through national nutrition program and pregnancy weight gain status in selected upazilas. *Bangladesh Med Res Counc Bull*. 2011;37(2):71-75. doi:10.3329/bmrcb.v37i2.8438	Wrong Intervention
132	Katzow M, Canfield C, Gross RS, et al. Maternal Depressive Symptoms and Perceived Picky Eating in a Low-Income, Primarily Hispanic Sample. *J Dev Behav Pediatr*. 2019;40(9):706-715. doi:10.1097/dbp.0000000000000715	Wrong Intervention

133	Kearney L, Kynn M, Craswell A, Reed R. The relationship between midwife-led group-based versus conventional antenatal care and mode of birth: a matched cohort study. *BMC Pregnancy Childbirth*. 2017;17:39-39. doi:10.1186/s12884-016-1216-1	Wrong Intervention
134	Kendal AP, Peterson A, Manning C, Xu F, Neville LJ, Hogue C. Improving the health of infants on Medicaid by collocating special supplemental nutrition clinics with managed care provider sites. *Am J Public Health*. 2002;92(3):399-403. doi:10.2105/ajph.92.3.399	Wrong Intervention
135	Kimani-Murage EW, Norris SA, Mutua MK, et al. Potential effectiveness of Community Health Strategy to promote exclusive breastfeeding in urban poor settings in Nairobi Kenya: a quasi-experimental study. *J Dev Orig Health Dis*. 2015;7:172-184. doi:10.1017/s2040174415007941	Wrong Intervention
136	Kramer MK, Cepak Y, Kriska A, Semler L, Venditti E. Evaluation of the group lifestyle balance program for diabetes prevention in a hispanic women infant and children (WIC) population. *Diabetes*. 2012;61:A175. doi:10.2337/db12-656-835	Wrong Intervention
137	Krummel D, Semmens E, MacBride AM, Fisher B. Lessons learned from the mothers' overweight management study in 4 West Virginia WIC offices. *J Nutr Educ Behav*. 2010;42(3 Suppl):S52-8. doi:10.1016/j.jneb.2010.02.012	Wrong Intervention
138	Kushwaha KP, Sankar J, Sankar MJ, et al. Effect of peer counselling by mother support groups on infant and young child feeding practices: the Lalitpur experience. *PLoS One*. 2014;9:e109181-undefined. doi:10.1371/journal.pone.0109181	Wrong Intervention
139	Langstaff S, Mottola M. Bringing an Evidence-Based Nutrition and Exercise Lifestyle Intervention Program (NELIP) for Obese Pregnant Women into Clinical Practice. 2015;(29243301):110.	Wrong Intervention
140	Lavner JA, Savage JS, Stansfield BK, et al. Effects of the sleep SAAF responsive parenting intervention on rapid infant weight gain: a randomized clinical trial of African American families. *Appetite*. 2022:106080-. doi:10.1016/j.appet.2022.106080	Wrong Intervention
141	Leroy JL, García-Guerra A, García R, Dominguez C, Rivera J, Neufeld LM. The Oportunidades program increases the linear growth of children enrolled at young ages in urban Mexico. *J Nutr*. 2008;138(4):793-798. doi:10.1093/jn/138.4.793	Wrong Intervention
142	Leroy JL, Olney DK, Ruel MT. PROCOMIDA, a Food-Assisted Maternal and Child Health and Nutrition Program, Contributes to Postpartum Weight Retention in Guatemala: A Cluster-Randomized Controlled Intervention Trial. *J Nutr*. 2019;149(12):2219-2227. doi:10.1093/jn/nxz175	Wrong Intervention
143	Li Ming W, Chris R, Baur LA, et al. A 3-Arm randomised controlled trial of Communicating Healthy Beginnings Advice by Telephone (CHAT) to mothers with infants to prevent childhood obesity. *BMC Public Health*. 2017;17(1):1-10. doi:10.1186/s12889-016-4005-x	Wrong Intervention
144	Lioret S, Campbell K, McNaughton S, et al. Parent focused intervention impacts obesity risk behaviours in infants: results of the melbourne infant program cluster-randomised controlled trial. *Obes Facts*. 2012;5:33-. doi:10.1159/000188329	Wrong Intervention

145	Lioret S, Campbell KJ, Crawford D, Spence AC, Hesketh K, McNaughton SA. A parent focused child obesity prevention intervention improves some mother obesity risk behaviors: the Melbourne inFANT program. *Int J Behav Nutr Phys Act*. 2012;9:100. doi:10.1186/1479-5868-9-100	Wrong Intervention
146	Lutter CK, Rodríguez A, Fuenmayor G, et al. Growth and micronutrient status in children receiving a fortified complementary food. *J Nutr*. 2008;138(2):379-388. doi:10.1093/jn/138.2.379	Wrong Intervention
147	Ly CT, Diallo A, Simondon F, Simondon KB. Early short-term infant food supplementation, maternal weight loss and duration of breast-feeding: a randomised controlled trial in rural Senegal. *Eur J Clin Nutr*. 2006;60(2):265-71. doi:10.1038/sj.ejcn.1602311	Wrong Intervention
148	Ma Y, Liang H, Jin Y. Data Analysis of Nursing Effects in Pediatric Gastroenterology Department under High Content Image Analysis Technology. *Contrast Media Mol Imaging*. 2022;2022:4302331. doi:10.1155/2022/4302331	Wrong Intervention
149	Macchi AK, Banna J, Moreira S, Campos M, Palacios C. Effect of a Short Messaging Service (SMS) intervention delivered to caregivers on energy, nutrients, and food groups intake in infant participants of the WIC program. *Front Public Health*. 2022;10:7. doi:10.3389/fpubh.2022.986330	Wrong Intervention
150	Mackerras D. Birthweight changes in the pilot phase of the Strong Women Strong Babies Strong Culture Program in the Northern Territory. *Aust N Z J Public Health*. 2001;25(1):34-40. doi:10.1111/j.1467-842x.2001.tb00547.x	Wrong Intervention
151	Mannan I, Rahman SM, Sania A, et al. Can early postpartum home visits by trained community health workers improve breastfeeding of newborns. *J Perinatol*. 2008;28:632-640. doi:10.1038/jp.2008.64	Wrong Intervention
152	Martinez JL, Perez-Escamilla R. Predictors of Exclusive Breastfeeding Behavior in Low-Income Women Attending the Special Supplemental Nutrition Program for Women, Infants, and Children. 2016;(10583269):189.	Wrong Intervention
153	McKechnie L. Family integrated care: A quality improvement project. *Arch Dis Child*. 2016;101:A253-A254. doi:10.1136/archdischild-2016-310863.418	Wrong Intervention
154	Mendelson R, Dollard D, Hall P, Zarrabi SY, Desjardin E. The impact of the Healthiest Babies Possible Program on maternal diet and pregnancy outcome in underweight and overweight clients. *J Can Diet Assoc*. 1991;52(4):229-34.	Wrong Intervention
155	Millard GL, Beerman KA, Massey L, Shulz T, Heiss C. Pregnancy and birth outcomes of high-risk women participating in a multidisciplinary intervention program. *Topics in Clinical Nutrition*. 1999;14(4):64-75.	Wrong Intervention
156	M'Liria JK. Effectiveness of mother-to-mother support groups in promoting of exclusive breastfeeding in Igembe South-Sub County, Meru County , Kenya: A Randomized Controlled Trial. 2015.	Wrong Intervention
157	Moening GA, Lovelady C. Diet quality and weight change among overweight and obese postpartum women enrolled in a behavioral intervention program. 2011;(1499601):89.	Wrong Intervention

158	Mohd Shukri NH, Eaton S, Fewtrell M, et al. Randomized controlled trial investigating the effects of a breastfeeding relaxation intervention on maternal psychological state, breast milk outcomes, and infant behavior and growth. *Am J Clin Nutr*. 2019;110(1):121-130. doi:10.1093/ajcn/nqz033	Wrong Intervention
159	Morandi A, Tommasi M, Soffiati F, et al. Correction: Prevention of obesity in toddlers (PROBIT): a randomised clinical trial of responsive feeding promotion from birth to 24 months. *Int J Obes (Lond).* 2019;43:1961-1966. doi:10.1038/s41366-019-0406-0	Wrong Intervention
160	Morrow AL, Guerrero ML, Shults J, et al. Efficacy of home-based peer counselling to promote exclusive breastfeeding: a randomised controlled trial. *Lancet*. 1999;353:1226-1231. doi:10.1016/s0140-6736(98)08037-4	Wrong Intervention
161	Mukhopadhyay DK, Sarkar AP, Aniket C, Eashin G, Sarkar GN. Can frontline workers be change agents for infant feeding and growth? - a community trial. *Al Ameen Journal of Medical Sciences*. 2017;10(1):71-77.	Wrong Intervention
162	Nct. BabyQ's: randomized Controlled Trial of Health Messaging in Pregnancy and Infancy. https://clinicaltrialsgov/show/NCT04238585. 2020.	Wrong Intervention
163	Nct. Best Start - Weight Management During Pregnancy. https://clinicaltrialsgov/show/NCT03875300. 2019.	Wrong Intervention
164	Nct. Childhood Risk Reduction Program in Hispanics. https://clinicaltrialsgov/show/NCT03903146. 2019.	Wrong Intervention
165	Nct. Community Enabled Readiness for First 1000 Days Learning Ecosystem. https://clinicaltrialsgov/show/NCT04275765. 2020.	Wrong Intervention
166	Nct. Effectiveness of an Early Nutrition Program on Promoting Breastfeeding and Optimizing Infant Growth and Diet Quality. https://clinicaltrialsgov/show/NCT03493594. 2018.	Wrong Intervention
167	Nct. Expanding Health System Intervention Through The Women, Infants and Children (WIC) Program Partnership. https://clinicaltrialsgov/show/NCT05356338. 2022.	Wrong Intervention
168	Nct. Grocery Delivery and Healthy Weight Gain Among Low-income Pregnant Young Women. https://clinicaltrialsgov/show/NCT05000645. 2021.	Wrong Intervention
169	Nct. Home Plate: a Trial to Improve Home Food Preparation Practices Among Parents of Toddlers. https://clinicaltrialsgov/show/NCT02458833. 2015.	Wrong Intervention
170	Nct. Improving Health in Low Income Women Following the Birth of a Child. https://clinicaltrialsgov/show/NCT00061386. 2003.	Wrong Intervention
171	Nct. Improving the Eating Habits of Mother and Her Infant Via Sugar Reduction. https://clinicaltrialsgov/show/NCT03141346. 2017.	Wrong Intervention
172	Nct. Increase Breastfeeding Duration Among Puerto Rican Mothers. https://clinicaltrialsgov/show/NCT02148237. 2014.	Wrong Intervention
173	Nct. Lifestyle Intervention Forever: healthy Weight for Pregnancy and Birth (Pilot Study). https://clinicaltrialsgov/show/NCT01693666. 2012.	Wrong Intervention
174	Nct. Lifestyle Intervention to Limit Excessive Weight Gain During Pregnancy in Minority Women. https://clinicaltrialsgov/show/NCT01084941. 2010.	Wrong Intervention

175	Nct. Maternal Nutrition Interventions in Uttar Pradesh, India. https://clinicaltrialsgov/show/NCT03378141. 2017.	Wrong Intervention
176	Nct. Maternal Obesity and Breastfeeding Performance. https://clinicaltrialsgov/show/NCT02756169. 2016.	Wrong Intervention
177	Nct. Mothers and Others: family-based Obesity Prevention for Infants and Toddlers. https://clinicaltrialsgov/show/NCT01938118. 2013.	Wrong Intervention
178	Nct. Office Based Intervention to Reduce Bottle Use in Toddlers: tARGet Kids! Pragmatic Randomized Trial. https://clinicaltrialsgov/show/NCT02140957. 2013.	Wrong Intervention
179	Nct. Opaque Bottle Study. https://clinicaltrialsgov/show/NCT03711370. 2018.	Wrong Intervention
	Ventura AK, Hernandez A. Effects of opaque, weighted bottles on maternal sensitivity and infant intake. *Matern Child Nutr*. 2018;15:e12737-undefined. doi:10.1111/mcn.12737	Wrong Intervention
	Ventura AK, Pollack Golen R. A pilot study comparing opaque, weighted bottles with conventional, clear bottles for infant feeding. *Appetite*. 2015;85:178-84. doi:10.1016/j.appet.2014.11.028	Wrong Intervention
180	Nct. Partnering With Media and Vaccination Program to Improve Infant and Young Child Feeding. https://clinicaltrialsgov/show/NCT01405755. 2011.	Wrong Intervention
181	Nct. Post Disaster Nutrition Intervention for Under-two-year-old Children in Lombok Indonesia. https://clinicaltrialsgov/show/NCT03895398. 2019.	Wrong Intervention
182	Nct. Preventing Childhood Obesity Through Early Guidance. https://clinicaltrialsgov/show/NCT01905072. 2013.	Wrong Intervention
183	Nct. Preventing Early Childhood Obesity, Part 1: family Spirit Nurture, 3-9 Months. https://clinicaltrialsgov/show/NCT03101943. 2017.	Wrong Intervention
184	Nct. Preventing Early Childhood Obesity, Part 2: family Spirit Nurture, Prenatal - 18 Months. https://clinicaltrialsgov/show/NCT03334266. 2017.	Wrong Intervention
185	Nct. Prevention of Overweight in Infancy. https://clinicaltrialsgov/show/NCT00892983. 2009.	Wrong Intervention
186	Nct. Reducing Obesity in Underserved Postpartum African American Women. https://clinicaltrialsgov/show/NCT02448563. 2015.	Wrong Intervention
187	Nct. Sleep-Safe: a Strong African American Families Study. https://clinicaltrialsgov/show/NCT03505203. 2018.	Wrong Intervention
188	Nct. The Baby Act Trial. https://clinicaltrialsgov/show/NCT03517891. 2018.	Wrong Intervention
189	Nct. The Healthy Lifestyles Passport Program: a Nutrition Education Program to Prevent Childhood Obesity. https://clinicaltrialsgov/show/NCT01649115. 2012.	Wrong Intervention
190	Nct. Using Digital Health Technologies to Prevent Rapid Infant Weight Gain. https://clinicaltrialsgov/show/NCT05265845. 2022.	Wrong Intervention
191	Nguyen P, Sanghvi T, Mahmud Z, et al. Integrating nutrition-focused behavior change communication and community mobilization into existing Maternal, Neonatal and child health platform improved consumption of diversified foods and micronutrients and exclusive breastfeeding practices in Bangladesh: Results of a cluster-randomized program evaluation. *FASEB J*. 2017;31(1).	Wrong Intervention

192	Nguyen PH, Kachwaha S, Tran LM, et al. Strengthening Nutrition Interventions in Antenatal Care Services Affects Dietary Intake, Micronutrient Intake, Gestational Weight Gain, and Breastfeeding in Uttar Pradesh, India: results of a Cluster-Randomized Program Evaluation. *J Nutr*. 2021;151(8):2282-2295. doi:10.1093/jn/nxab131	Wrong Intervention
193	Nichols J, Schutte NS, Brown RF, Dennis C, Price IR. The Impact of a Self-Efficacy Intervention on Short-Term Breast-Feeding Outcomes. *Health Educ Behav.* 2007;36:250-258. doi:10.1177/1090198107303362	Wrong Intervention
194	Nita B, Sarmila M, Rajiv B, Martines J, Black RE, Bhan MK. An educational intervention to promote appropriate complementary feeding practices and physical growth in infants and young children in rural Haryana, India. *J Nutr*. 2004;134(9):2342-2348.	Wrong Intervention
195	Nyström CD, Cameron AJ, Campbell KJ, Hesketh KD. Variation in outcomes of the Melbourne Infant, Feeding, Activity, and Nutrition Trial (INFANT) according to maternal education and age 2 and 3.5 years post-intervention. *Public Health Nutr*. 2021;24:1460-1468. doi:10.1017/s1368980021000045	Wrong Intervention
196	Ochola S, Labadarios D, Nduati R. Impact of counselling on exclusive breast-feeding practices in a poor urban setting in Kenya: a randomized controlled trial. *Public Health Nutr*. 2012;16:1732-1740. doi:10.1017/s1368980012004405	Wrong Intervention
197	Oken E, Guthrie LB, Bloomingdale A, et al. A pilot randomized controlled trial to promote healthful fish consumption during pregnancy: The Food for Thought Study. *Nutr J*. 2013;12:11. doi:10.1186/1475-2891-12-33	Wrong Intervention
198	Ordway MR, Sadler LS, Holland ML, Slade A, Close N, Mayes LC. A home visiting parenting program and child obesity: a randomized trial. *Pediatrics*. 2018;141(2):peds.2017-1076.	Wrong Intervention
199	Øverby NC, Hillesund ER, Helland SH, et al. Evaluating the effectiveness and implementation of evidence-based early-life nutrition interventions in a community setting a hybrid type 1 non-randomized trial - the Nutrition Now project protocol. *Front Endocrinol (Lausanne)*. 2023;13:1071489-undefined. doi:10.3389/fendo.2022.1071489	Wrong Intervention
200	Owais A, Schwartz B, Kleinbaum DG, et al. A Nutrition Education Program in Rural Bangladesh Was Associated with Improved Feeding Practices but Not with Child Growth. *J Nutr*. 2017;147(5):948-954. doi:10.3945/jn.116.243956	Wrong Intervention
201	Pactr. Effect of promoting fruits and vegetables consumption on maternal nutritional status and birth weight at Robe and Goba Towns, South East Ethiopia: a cluster randomized controlled trial. https://trialsearchwhoint/Trial2aspx?TrialID=PACTR202201731802989. 2021.	Wrong Intervention
202	Pactr. Randomized controlled trial to study the effect of nutritional intervention from farm to feeding on gestational weight gain, anemia and child nutritio. https://trialsearchwhoint/Trial2aspx?TrialID=PACTR201804003012418. 2018.	Wrong Intervention
203	Patel N, Godfrey KM, Pasupathy D, et al. Infant adiposity following a randomised controlled trial of a behavioural intervention in obese pregnancy. *Int J Obes (Lond)*. 2017;41(7):1018-1026. doi:10.1038/ijo.2017.44	Wrong Intervention
204	Paul IM, Savage JS, Anzman SL, et al. Preventing obesity during infancy: a pilot study. *Obes*. 2011;19(2):353-361. doi:10.1038/oby.2010.182	Wrong Intervention

205	Paul IM, Savage JS, Anzman SL, et al. Preventing Obesity during Infancy: A Pilot Study. *Obesity (Silver Spring)*. 2010;19:353-361. doi:10.1038/oby.2010.182	Wrong Intervention
206	Paul IM, Savage JS, Anzman-Frasca S, et al. Effect of a Responsive Parenting Educational Intervention on Childhood Weight Outcomes at 3 Years of Age: The INSIGHT Randomized Clinical Trial. *JAMA*. 2018;320(5):461-468. doi:10.1001/jama.2018.9432	Wrong Intervention
207	Peacock L, Seed PT, Dalrymple KV, White SL, Poston L, Flynn AC. The UK Pregnancies Better Eating and Activity Trial (UPBEAT); Pregnancy Outcomes and Health Behaviours by Obesity Class. *Int J Environ Res Public Health*. 2020;17(13)doi:10.3390/ijerph17134712	Wrong Intervention
208	Penny ME, Creed-Kanashiro HM, Robert RC, Narro MR, Caulfield LE, Black RE. Effectiveness of an educational intervention delivered through the health services to improve nutrition in young children: a cluster-randomised controlled trial. *Lancet*. 2005;365(9474):1863-72. doi:10.1016/s0140-6736(05)66426-4	Wrong Intervention
209	Phelan S, Hagobian T, Brannen A, et al. Effect of an Internet-Based Program on Weight Loss for Low-Income Postpartum Women: A Randomized Clinical Trial. *JAMA*. 2017;317(23):2381-2391. doi:10.1001/jama.2017.7119	Wrong Intervention
210	Phelan S, Phipps MG, Abrams B, et al. Does behavioral intervention in pregnancy reduce postpartum weight retention? Twelve-month outcomes of the Fit for Delivery randomized trial. *Am J Clin Nutr*. 2013;99:302-311. doi:10.3945/ajcn.113.070151	Wrong Intervention
211	Pisacane A, Continisio GI, Aldinucci M, D'Amora S, Continisio P. A controlled trial of the father's role in breastfeeding promotion. *Pediatrics*. 2005;116:e494-8. doi:10.1542/peds.2005-0479	Wrong Intervention
212	Prado B, McMahan S, Mouttapa M, Salazar I, Love G, Norwood S. An educational telenovela (soap opera) approach to promote breastfeeding among U.S. Latinas. *Calif J Health Promot*. 2012;10(Special issue 1):67-73.	Wrong Intervention
213	Puharić D, Malički M, Borovac JA, et al. The effect of a combined intervention on exclusive breastfeeding in primiparas: A randomised controlled trial. *Matern Child Nutr*. 2020;16(3):1-12. doi:10.1111/mcn.12948	Wrong Intervention
214	Qureshi AM, Oche OM, Sadiq UA, Kabiru S. Using Community Volunteers to Promote Exclusive Breastfeeding in Sokoto State, Nigeria. *Pan Afr Med J*. 2011;10:8-8. doi:10.4314/pamj.v10i0.72215	Wrong Intervention
215	Reifsnider E, McCormick DP, Cullen KW, et al. A randomized controlled trial to prevent childhood obesity through early childhood feeding and parenting guidance: rationale and design of study. *BMC Public Health*. 2013;13:880. doi:10.1186/1471-2458-13-880	Wrong Intervention
216	Ricketts S, Tolliver R, Schwalberg R. Short-lived success: assessment of an intervention to improve pregnancy weight gain in Colorado. *Matern Child Health J*. 2014;18(4):772-7. doi:10.1007/s10995-013-1336-4	Wrong Intervention
217	Ronnberg A, Hanson U, Östlund I, Nilsson K. Effects on postpartum weight retention after antenatal lifestyle intervention - a secondary analysis of a randomized controlled trial. *Acta Obstetricia et Gynecologica Scandinavica*. 2016;95:999-1007. doi:10.1111/aogs.12910	Wrong Intervention

218	Rosenstock S, Ingalls A, Foy Cuddy R, et al. Effect of a Home-Visiting Intervention to Reduce Early Childhood Obesity Among Native American Children: a Randomized Clinical Trial .JAMA Pediatr. 2021;175(2):133-142. doi:10.1001/jamapediatrics.2020.3557	Wrong Intervention
219	Rotheram-Borus MJ, Tomlinson M, le Roux IM, et al. A cluster randomised controlled effectiveness trial evaluating perinatal home visiting among South African mothers/infants. *PLoS One*. 2014;9:e105934-undefined. doi:10.1371/journal.pone.0105934	Wrong Intervention
220	Rotheram-Fuller E, Swendeman D, Becker K, et al. Adapting Current Strategies to Implement Evidence-Based Prevention Programs for Paraprofessional Home Visiting. *Prev Sci*. 2017;18(5):590-599. doi:10.1007/s11121-017-0787-z	Wrong Intervention
221	Rush D, Sloan NL, Leighton J, et al. V. Longitudinal study of pregnant women. *Am J Clin Nutr*. 1988;48(2 SUPPL.):439-483. doi:10.1093/ajcn/48.2.439	Wrong Intervention
222	Rush D. Nutritional services during pregnancy and birthweight: a retrospective matched pair analysis. *Can Med Assoc J*. 1981;125(6):567-76.	Wrong Intervention
223	Sagedal LR, Sanda B, Øverby NC, et al. The effect of prenatal lifestyle intervention on weight retention 12 months postpartum: results of the Norwegian Fit for Delivery randomised controlled trial. *BJOG*. 2016;124:111-121. doi:10.1111/1471-0528.13863	Wrong Intervention
224	Sandborg J, Henriksson P, Söderström E, et al. The effects of a lifestyle intervention (the HealthyMoms app) during pregnancy on infant body composition: Secondary outcome analysis from a randomized controlled trial. *Pediatr Obes*. 2022;17(6):e12894. doi:10.1111/ijpo.12894	Wrong Intervention
225	Sanghvi T, Haque R, Roy S, et al. Achieving behaviour change at scale: Alive & Thrive's infant and young child feeding programme in Bangladesh. *Matern Child Nutr*. 2016;12:141-154. doi:10.1111/mcn.12277	Wrong Intervention
226	Santos I, Victora CG, Martines J, et al. Nutrition counseling increases weight gain among Brazilian children. *J Nutr*. 2001;131(11):2866-73. doi:10.1093/jn/131.11.2866	Wrong Intervention
227	Santos KD, Patrício PT, Lima TSV, Barros DC, Saunders C. A pilot intervention to reduce postpartum weight retention at primary health care in Brazil. *Nutr Hosp*. 2019;36(4):854-861. doi:10.20960/nh.02508	Wrong Intervention
228	Savage JS, Birch LL, Hohman EE, Marini ME, Paul IM, Shelly A. INSIGHT responsive parenting intervention and infant feeding practices: randomized clinical trial. *Int J Behav Nutr Phys Act*. 2018;15(1):64-64. doi:10.1186/s12966-018-0700-6	Wrong Intervention
229	Savage JS, Birch LL, Marini M, Anzman-Frasca S, Paul IM. Effect of the INSIGHT Responsive Parenting Intervention on Rapid Infant Weight Gain and Overweight Status at Age 1 Year: A Randomized Clinical Trial. *JAMA Pediatr*. 2016;170(8):742-9. doi:10.1001/jamapediatrics.2016.0445	Wrong Intervention
230	Shim JE, Kim J, Heiniger JB. Breastfeeding Duration in Relation to Child Care Arrangement and Participation in the Special Supplemental Nutrition Program for Women, Infants, and Children. *J Hum Lact*. 2012;28(1):28-35. doi:10.1177/0890334411424728	Wrong Intervention

231	Singh JK, Kadel R, Acharya D, Lombard D, Khanal S, Singh SP. 'MATRI-SUMAN' a capacity building and text messaging intervention to enhance maternal and child health service utilization among pregnant women from rural Nepal: study protocol for a cluster randomised controlled trial. *BMC Health Serv Res*. 2018;18(1):447. doi:10.1186/s12913-018-3223-6	Wrong Intervention
232	Singh V, Ahmed S, Dreyfuss ML, et al. An integrated nutrition and health program package on IYCN improves breastfeeding but not complementary feeding and nutritional status in rural northern India: a quasi-experimental randomized longitudinal study. *PLoS One*. 2017;12(9):e0185030. doi:10.1371/journal.pone.0185030	Wrong Intervention
233	Smithers LG, Lynch J, Hedges J, Jamieson LM. Diet and anthropometry at 2 years of age following an oral health promotion programme for Australian Aboriginal children and their carers: a randomised controlled trial. *Br J Nutr*. 2017;118(12):1061-1069. doi:10.1017/s000711451700318x	Wrong Intervention
234	Sobko T, Svensson V, Ek A, et al. A randomised controlled trial for overweight and obese parents to prevent childhood obesity--Early STOPP (STockholm Obesity Prevention Program). *BMC Public Health*. 2011;11:336. doi:10.1186/1471-2458-11-336	Wrong Intervention
235	Soliman AZM, Hassan A, Fahmy HH, Abdelsalam AE, Salem MAA. Maternal and fetal outcomes of pregnant females after a nutritional health education program. An interventional study. *Prog Nutr*. 2019;21(4):1063-1070. doi:10.23751/pn.v21i4.8934	Wrong Intervention
236	Soliman AZM, Hassan AS, Fahmy HH, Abdelsalam AE, Salem MAA. Obstetrics outcome after a nutritional health education program for pregnant females at zagazig university hospitals; an interventional study. *Open Public Health Journal*. 2019;12(1):496-503. doi:10.2174/1874944501912010496	Wrong Intervention
237	Sontag-Padilla L, Burns RM, Shih RA, et al. The Urban Child Institute CANDLE Study. 2015.	Wrong Intervention
238	Spence AC, Campbell KJ, Crawford DA, McNaughton SA, Hesketh KD. Mediators of improved child diet quality following a health promotion intervention: the Melbourne InFANT Program. *Int J Behav Nutr Phys Act*. 2014;11:137. doi:10.1186/s12966-014-0137-5	Wrong Intervention
239	Spence AC, McNaughton SA, Lioret S, Hesketh KD, Crawford DA, Campbell KJ. A health promotion intervention can affect diet quality in early childhood. *J Nutr*. 2013;143(10):1672-8. doi:10.3945/jn.113.177931	Wrong Intervention
240	Spencer B, Thomas H, Morris J. A randomized controlled trial of the provision of a social support service during pregnancy: the South Manchester Family Worker Project. *Br J Obstet Gynaecol*. 1989;96(3):281-8. doi:10.1111/j.1471-0528.1989.tb02387.x	Wrong Intervention
241	Sreeparna Ghosh M, Nagma Nigar S, Pia S. Ensuring pregnancy weight gain: an integrated community-based approach to tackle maternal nutrition in India. *Field Exch*. 2019;(61):14-17.	Wrong Intervention
242	Srinivas GL, Benson M, Worley S, Schulte E. A clinic-based breastfeeding peer counselor intervention in an urban, low-income population: interaction with breastfeeding attitude. *J Hum Lact*. 2014;31:120-128. doi:10.1177/0890334414548860	Wrong Intervention
243	Strully KW, Rehkopf DH, Xuan Z. Effects of Prenatal Poverty on Infant Health: State Earned Income Tax Credits and Birth Weight. *Am Sociol Rev*. 2010;75:534-562. doi:10.1177/0003122410374086	Wrong Intervention

244	Sudarmi, Bertalina, Berawi KN. Assistance intervention in the form of distribution of local food (serwit) with the implementation of interprofessional education-collaborative practices on nutritional status of pregnant women. *Eur J Mol Clin Med*. 2020;7(9):192-204.	Wrong Intervention
245	Tahir NM, Al-Sadat N. Does telephone lactation counselling improve breastfeeding practices? A randomised controlled trial. *Int J Nurs Stud*. 2012;50:16-25. doi:10.1016/j.ijnurstu.2012.09.006	Wrong Intervention
246	Taki S, Li Ming W, Shaw M, Caffrey P, Gordon P. Integrating an effective obesity prevention program into existing home visiting services: The Healthy Beginnings Program. *Int J Integr Care*. 2018;18:1-2. doi:10.5334/ijic.s1164	Wrong Intervention
247	Taylor B, Taylor R, Gray A, et al. The prevention of obesity in infancy by targeting sleep or food and activity: RCT outcomes at 5 years. *Obes Facts*. 2017;10:23. doi:10.1159/000468958	Wrong Intervention
248	Taylor BJ, Heath A-LM, Galland BC, et al. Prevention of Overweight in Infancy (POI.nz) study: a randomised controlled trial of sleep, food and activity interventions for preventing overweight from birth. BMC Public Health. 2011;11(1):942-942. doi:10.1186/1471-2458-11-942	Wrong Intervention
249	Taylor R. Providing additional guidance and support to parents about sleep, diet and physical activity from birth to 2 years of age: The Prevention of Overweight in Infancy study. *Obes Res Clin Pract*. 2014;8:102-103. doi:org/10.1016/j.orcp.2014.10.187	Wrong Intervention
250	Tommasone G, Bazzani M, Solinas V, et al. Midwifery E-Health: from design to validation of "Mammastyle - Gravidanza Fisiologica". 18th IEEE International Conference on e-Health Networking, Applications and Services (Healthcom). 2016:454-459.	Wrong Intervention
251	Tylleskär T, Jackson D, Meda N, et al. Exclusive breastfeeding promotion by peer counsellors in sub-Saharan Africa (PROMISE-EBF) : a cluster-randomised trial. *Lancet*. 2011;378:420-427. doi:10.1016/s0140-6736(11)60738-1	Wrong Intervention
252	van der Veek SMC, de Graaf C, de Vries JHM, et al. BMC Pediatrics - Baby's first bites: a randomized controlled trial to assess the effects of vegetable-exposure and sensitive feeding on vegetable acceptance, eating behavior and weight gain in infants and toddlers. *BMC Pediatr*. 2019;19:266-266. doi:10.1186/s12887-019-1627-z	Wrong Intervention
253	Varea A, Malpeli A, Disalvo L, et al. Evaluation of the Impact of a Food Program on the Micronutrient Nutritional Status of Argentinean Lactating Mothers. *Biol Trace Elem Res*. 2012;150(1-3):103-108. doi:10.1007/s12011-012-9512-8	Wrong Intervention
254	Verbestel V, De Bourdeaudhuij I, De Coen V, Huybrechts I, Maes L, Van Winckel M. Prevention of overweight in children younger than 2 years old: a pilot cluster-randomized controlled trial. *Public Health Nutr*. 2014;17(6):1384-1392. doi:10.1017/S1368980013001353	Wrong Intervention
255	Verbestel V, De Coen V, Van Winckel M, Huybrechts I, Maes L, De Bourdeaudhuij I. Prevention of overweight in children younger than 2 years old: a pilot cluster-randomized controlled trial. *Public Health Nutr*. 2013;17:1384-1392. doi:10.1017/s1368980013001353	Wrong Intervention
256	Vieten C, Laraia BA, Kristeller J, et al. The mindful moms training: development of a mindfulness-based intervention to reduce stress and overeating during pregnancy. *BMC Pregnancy Childbirth*. 2018;18(1):201. doi:10.1186/s12884-018-1757-6	Wrong Intervention

257	Vogl TS. Urban land rights and child nutritional status in Peru, 2004. *Econ Hum Biol*. 2007;5(2):302-21. doi:10.1016/j.ehb.2007.01.001	Wrong Intervention
258	Walker LO, Sterling BS, Latimer L, Kim SH, Garcia AA, Fowles ER. Ethnic-Specific Weight-Loss Interventions for Low-Income Postpartum Women: Findings and Lessons. *W J Nurs Res*. 2012;34(5):654-676. doi:10.1177/0193945911403775	Wrong Intervention
259	Walshaw CA, Owens JM, Scally AJ, Walshaw MJ. Does breastfeeding method influence infant weight gain? Arch Dis Child. 2008;93(4):292-6. doi:10.1136/adc.2006.107102	Wrong Intervention
260	Wambach K, Aaronson LS, Breedlove G, Domian EW, Rojjanasrirat W, Yeh H. A randomized controlled trial of breastfeeding support and education for adolescent mothers. *W J Nurs Res.* 2010;33:486-505. doi:10.1177/0193945910380408	Wrong Intervention
261	Wasser H, Bentley M, Thompson A. Home-based Intervention Among Non-Hispanic Black Families Finds No Significant Difference in Growth at 15 Months: Results from the 'Mothers & Others' Randomized Trial (P04-187-19). *Curr Dev Nutr*. 2019;3(Supplement_1)doi:10.1093/cdn/nzz051.P04-187-19	Wrong Intervention
262	Wasser HM, Thompson AL, Suchindran CM, et al. Family-based obesity prevention for infants: Design of the "Mothers & Others" randomized trial. *Contemp Clin Trials*. 2017;60:24-33. doi:10.1016/j.cct.2017.06.002	Wrong Intervention
263	Wasser HM, Thompson AL, Suchindran CM, et al. Home-based intervention for non-Hispanic black families finds no significant difference in infant size or growth: results from the Mothers & Others randomized controlled trial. *BMC Pediatr*. 2020;20(1):385. doi:10.1186/s12887-020-02273-9	Wrong Intervention
264	Wen L, Baur LA, Rissel C, Wardle K, Alperstein G, Simpson JM. Early intervention of multiple home visits to prevent childhood obesity in a disadvantaged population: a home-based randomised controlled trial (Healthy Beginnings Trial). *BMC Public Health*. 2007;7(76):(10 May 2007). doi:10.1186/1471-2458-7-76	Wrong Intervention
265	Wen L, Rissel C, Xu H, Taki S, Baur L. Obesity prevention in the first years of life: Stories beyond publications from Healthy Beginnings research and translation. *Obes Rev*. 2020;21(SUPPL 1)doi:10.1111/obr.13115	Wrong Intervention
266	Wen LM, Baur LA, Rissel C, Simpson JM. A randomized controlled trial of an early intervention on childhood obesity: Results from the first 12 months. *Obes*. 2011;19:S67. doi:10.1038/oby.2011.222	Wrong Intervention
267	Wen LM, Baur LA, Simpson JM, Rissel C, Flood VM. Effectiveness of an Early Intervention on Infant Feeding Practices and "Tummy Time" A Randomized Controlled Trial. *Arch Pediatr Adolesc Med*. 2011;165(8):701-707. doi:10.1001/archpediatrics.2011.115	Wrong Intervention
268	Wen LM, Baur LA, Simpson JM, Rissel C, Wardle K, Flood VM. Effectiveness of home based early intervention on children's BMI at age 2: randomised controlled trial. *BMJ*. 2012;344:e3732. doi:10.1136/bmj.e3732	Wrong Intervention
269	Wen LM, Baur LA, Simpson JM, Rissel C, Wardle K, Flood VM. Healthy beginnings trial: The journey from the beginning. *Obes Res Clin Pract*. 2013;7:e2. doi:10.1016/j.orcp.2013.12.502	Wrong Intervention

270	Wen LM, Xu H, Taki S, et al. Effects of telephone support or short message service on body mass index, eating and screen time behaviours of children age 2 years: A 3-arm randomized controlled trial. *Pediatr Obes*. 2021;17:e12875-undefined. doi:10.1111/ijpo.12875	Wrong Intervention
271	Whooten RC, Kwete GM, Farrar-Muir H, et al. Engaging fathers in the first 1000 days to improve perinatal outcomes and prevent obesity: Rationale and design of the First Heroes randomized trial. *Contemp Clin Trials*. 2020;101:106253-undefined. doi:10.1016/j.cct.2020.106253	Wrong Intervention
272	Whyte K, Johnson J, Kelly K, et al. No sustained effects of an intervention to prevent excessive GWG on offspring fat and lean mass at 54 weeks: Yet a greater head circumference persists. *Pediatr Obes*. 2021;16(7):1-8. doi:10.1111/ijpo.12767	Wrong Intervention
273	Widodo T, Sumarmi S. The influence of monitoring activities on maternal weight gain among pregnant women. *J Public Health Res*. 2020;9(2):1845. doi:10.4081/jphr.2020.1845	Wrong Intervention
274	Wilkinson SA, van der Pligt P, Gibbons K, McIntyre HD. Trial for Reducing Weight Retention in New Mums: a randomised controlled trial evaluating a low intensity, postpartum weight management programme. *J Hum Nutr Diet*. 2013;28:15-28. doi:10.1111/jhn.12193	Wrong Intervention
275	Williams C, Cprek S, Asaolu I, et al. Kentucky Health Access Nurturing Development Services Home Visiting Program Improves Maternal and Child Health. *Matern Child Health J*. 2017;21(5):1166-1174. doi:10.1007/s10995-016-2215-6	Wrong Intervention
276	Woo Baidal JA, Nichols K, Charles N, et al. Text messages to curb sugar-sweetened beverage consumption among pregnant women and mothers: a mobile health randomized controlled trial. *Nutrients*. 2021;13(12)doi:10.3390/nu13124367	Wrong Intervention
277	Yan J. The Impact of Medicaid Managed Care on Obstetrical Care and Birth Outcomes: A Case Study. *J Womens Health (Larchmt)*. 2020;29(2):167-176. doi:10.1089/jwh.2019.7792	Wrong Intervention
278	Zakarija-Grković I, Puharić D, Malički M, Hoddinott P. Breastfeeding booklet and proactive phone calls for increasing exclusive breastfeeding rates: RCT protocol. *Matern Child Nutr*. 2016;13doi:10.1111/mcn.12249	Wrong Intervention
279	Zhang J, Xu P, Liu F. One-child policy and childhood obesity. *China Econ Rev*. 2020;59:19. doi:10.1016/j.chieco.2016.05.003	Wrong Intervention
280	Zywicka B, Fihosy S, Kolotourou M. New mum, new you: Preliminary results of a post-natal healthy lifestyle and weight management intervention. *Acta Paediatr*. 2017;106:37.	Wrong Intervention
281	Anderson CE, Whaley SE, Crespi CM, Wang MC, Chaparro MP. Mixed Infant Feeding Is Not Associated With Increased Risk of Decelerated Growth Among WIC-Participating Children in Southern California. *Front Nutr*. 2021;8:9. doi:10.3389/fnut.2021.723501	Wrong Outcome(s)
282	Appel JM, Fullerton K, Hennessy E, et al. Design and methods of Shape Up Under 5: Integration of systems science and community-engaged research to prevent early childhood obesity. *PLoS One*. 2019;14(8):e0220169. doi:10.1371/journal.pone.0220169	Wrong Outcome(s)
283	Benjamin Neelon S, Taveras E, Østbye T, Gillman M. Preventing Obesity in Infants and Toddlers in Child Care: Results from a Pilot Randomized Controlled Trial. *Matern Child Health J*. 2014;18(5):1246-1257. doi:10.1007/s10995-013-1359-x	Wrong Outcome(s)

284	Biediger-Friedman L, Friedman B, Crixell SH. Best Food for Families, Infants and Toddlers Child-Care Center Intervention to Prevent Childhood Obesity...Insights to Action. Academy of Nutrition and Dietetics. Food & Nutrition Conference & Expo, Houston TX, October 19-22, 2013. *J Acad Nutr Diet*. 2013;113:A75-A75. doi:10.1016/j.jand.2013.06.257	Wrong Outcome(s)
285	Bonvecchio A, Reyes H, Neufeld L, et al. Promoting healthy growth in the context of a nutrition transition: Design of a social marketing model to improve primary care practices. *FASEB J*. 2014;28(1)	Wrong Outcome(s)
286	Boyd NR, Windsor R. A formative evaluation in maternal and child health practice: the Partners for Life Nutrition Education Program for pregnant women. *Matern Child Health J*. 2003;7:137-143. doi:10.1023/a:1023873112024	Wrong Outcome(s)
287	Carter EB, Barbier K, Sarabia R, Macones GA, Cahill AG, Tuuli MG. Group versus traditional prenatal care in low-risk women delivering at term: a retrospective cohort study. *J Perinatol*. 2017;37:769-771. doi:10.1038/jp.2017.33	Wrong Outcome(s)
288	Claesson IM, Brynhildsen J, Cedergren M, Jeppsson A, Sydsjo A, Josefsson A. Weight gain restriction during pregnancy is safe for both the mother and neonate. *Acta Obstetricia et Gynecologica Scandinavica*. 2009;88(10):1158-1162. doi:10.1080/00016340903214916	Wrong Outcome(s)
289	Crockett AH, Chen L, Heberlein EC, et al. Group vs traditional prenatal care for improving racial equity in preterm birth and low birthweight: the Centering and Racial Disparities randomized clinical trial study. *Am J of Obstet Gynecol*. 2022;227:893.e1-893.e15. doi:10.1016/j.ajog.2022.06.066	Wrong Outcome(s)
290	Ctri. Prevention of Gestational Diabetes by multi-pronged intervention - A cluster RCT. https://trialsearchwhoint/Trial2aspx?TrialID=CTRI/2022/07/043989. 2022.	Wrong Outcome(s)
291	Fealy S, Hure A, Browne G, Prince C. Developing a clinical care pathway for obese pregnant women: A quality improvement project. *Women Birth*. 2014;27(4):e67-71. doi:10.1016/j.wombi.2014.09.001	Wrong Outcome(s)
292	García-de-León-González R, Oliver-Roig A, Hernández-Martínez M, et al. Becoming baby-friendly in Spain: a quality-improvement process. *Acta paediatr (Oslo, Norway : 1992)*. 2010;100:445-450. doi:10.1111/j.1651-2227.2010.02061.x	Wrong Outcome(s)
293	Griffith R, von Hinke S, Smith S. Getting a healthy start: The effectiveness of targeted benefits for improving dietary choices. *Am J Health Econ*. 2018;58:176-187. doi:10.1016/j.jhealeco.2018.02.009	Wrong Outcome(s)
294	Hanks AS, Gunther C, Lillard DR, Scharff RL. From paper to plastic: Understanding the impact of eWIC on WIC recipient behavior. *Food Policy*. 2019;83:83-91. doi:10.1016/j.foodpol.2018.12.002	Wrong Outcome(s)
295	Havas S, Anliker J, Damron D, Langenberg P, Ballesteros MF, Feldman R. Final results of the Maryland WIC 5-A-Day Promotion Program. *Am J Public Health*. 1998;88:1161-1167. doi:10.2105/ajph.88.8.1161	Wrong Outcome(s)
296	Heins Jr HC, Webster Nance N, McCarthy BJ, Melvin Efird C. A randomized trial of nurse-midwifery prenatal care to reduce low birth weight. *Obstet Gynecol*. 1990;75(3 I):341-345.	Wrong Outcome(s)

297	Henderson JL. Breastfeeding Initiation and Maintenance Among African Americans and Blacks Enrolled in a Nurse Home Visitation Program: An Outcomes Focused Program Evaluation. 2013;doi:10.21236/ad1012834	Wrong Outcome(s)
298	Hillier A, McLaughlin J, Cannuscio CC, Chilton M, Krasny S, Karpyn A. The Impact of WIC Food Package Changes on Access to Healthful Food in 2 Low-Income Urban Neighborhoods. *J Nutr Educ Behav*. 2012;44:210-216. doi:10.1016/j.jneb.2011.08.004	Wrong Outcome(s)
299	Kennedy E, Gershoff SN, Reed R, Austin JE. Evaluation of the effect of WIC supplemental feeding on birth weight. *J Am Diet Assoc*. 1982;80:220-227. doi:10.1016/s0002-8223(21)08452-2	Wrong Outcome(s)
300	Kennedy ET, Kotelchuck M. The effect of WIC supplemental feeding on birth weight: a case-control analysis. *Am J Clin Nutr*. 1984;40:579-585. doi:10.1093/ajcn/40.3.579	Wrong Outcome(s)
301	Khan M, Akram DS. Effects of baby-friendly hospital initiative on breast-feeding practices in sindh. *J Pak Med Assoc (1951)*. 2013;63:756-759.	Wrong Outcome(s)
302	Lan Y, Hanks A. Three Essays on Participation in and Effects of US Food Assistance Programs. 2021;(28890225):164.	Wrong Outcome(s)
303	Lewis JB, Cunningham SD, Shabanova V, et al. Group prenatal care and improved birth outcomes: Results from a type 1 hybrid effectiveness-implementation study. *Prev Med*. 2021;153:106853-undefined. doi:10.1016/j.ypmed.2021.106853	Wrong Outcome(s)
304	Lin WT, Hsieh CC, Chang FC, Wang CL, Lin CI, Chuang HY. Effects of workplaces receiving "accreditation of health workplaces" on breastfeeding promotion, parental leave, and gender equality. *J Occup Health*. 2020;62(1):10. doi:10.1002/1348-9585.12140	Wrong Outcome(s)
305	Lindberg S, Anderson C. Improving Gestational Weight Gain Counseling Through Meaningful Use of an Electronic Medical Record. *Matern Child Health J*. 2014;18(9):2188-2194. doi:10.1007/s10995-014-1467-2	Wrong Outcome(s)
306	Louis-Jacques AF, Vereen S, Hernandez I, et al. Impact of Doula-Led Lactation Education on Breastfeeding Outcomes in Low-Income, Minoritized Mothers. *J Perinat Educ*. 2021;30:203-undefined. doi:10.1891/j-pe-d-20-00022	Wrong Outcome(s)
307	McInnes RJ, Stone DH. The process of implementing a community-based peer breast-feeding support programme: the Glasgow experience. *Midwifery*. 2001;17:65-73. doi:10.1054/midw.2000.0236	Wrong Outcome(s)
308	Namirembe G, Shrestha R, Mezzano J, et al. Effective nutrition governance is correlated with better nutrition outcomes in Nepal. *BMC Pediatr*. 2021;21(1):434. doi:10.1186/s12887-021-02898-4	Wrong Outcome(s)
309	Natale R, Camejo ST, Sanders LM. Communities Putting Prevention to Work: Results of an Obesity Prevention Initiative in Child Care Facilities. *J Res Child Educ*. 2016;30:306-319. doi:10.1080/02568543.2016.1178672	Wrong Outcome(s)
310	Nct. Impact of Home Weight Tele-monitoring on the Number of Office Visits in the First Six Weeks of Life in Infants. https://clinicaltrialsgov/show/NCT04985227. 2021.	Wrong Outcome(s)
311	Nct. The Maternal Well-Being Study. https://clinicaltrialsgov/show/NCT05484999. 2022.	Wrong Outcome(s)

312	Pellerin A. The nutritional impact of an integrated rural development project in Haiti: A quasi-experimental study. 1991;(9113322):761.	Wrong Outcome(s)
313	Rankins J, Maloney MA, Rainford K, Hopkins M. A comparative report on rehabilitation interventions and undernutrition outcomes in 12-35 month olds in Jamaica. J Nutr Educ. 1990;22(1):39-46.	Wrong Outcome(s)
314	Rozga MR. Breastfeeding practices, program efficacy, and reasons for breastfeeding discontinuation for low-income women enrolled in a peer counseling breastfeeding support program. 2014.	Wrong Outcome(s)
315	Santos MMAdS, Cavalcante de Barros D, Lima Nogueira J, Ribeiro Baiao M, Saunders C. Impact of an intervention nutrition program during prenatal on the weight of newborns from teenage mothers. *Nutr Hosp*. 2013;28(6):1943-1950. doi:10.3305/nutr hosp.v28in06.6860	Wrong Outcome(s)
316	Scharff DP, Elliott M, Rechtenwald A, Allen J, Strand G. Evidence of Effectiveness of a Home Visitation Program on Infant Weight Gain and Breastfeeding. *Matern Child Health J*. 2021;25(4):676-683. doi:10.1007/s10995-020-03072-5	Wrong Outcome(s)
317	Shrader K. Utilizing Group Prenatal Care to Support Underserved Pregnant Women. 2021.	Wrong Outcome(s)
318	Spivey CBB, Klerman LV. The influence of counseling and education on behavior and pregnancy outcome in African American Medicaid -eligible women. 2002;(3082023):96.	Wrong Outcome(s)
319	Srinivas SK, Durnwald C, Line L, et al. 295: “Safe Start”: A community health worker program that improves perinatal outcomes in high risk women. *Am J of Obstet Gynecol*. 2019;220(1):S208. doi:10.1016/j.ajog.2018.11.316	Wrong Outcome(s)
320	Taylor N, Sahota P, Sargent J, Barber SE, Loach J, Wright J. Development of HAPPY (Healthy and Active Parenting Programme for the early Years) an intervention to prevent childhood obesity: A theory-based approach. *Obes Facts*. 2013;6:159.	Wrong Outcome(s)
321	Thornton HEB, Amanda MR, Julia AVB, Sylvia HC. Differences in Energy and Micronutrient Intakes Among Central Texas WIC Infants and Toddlers After the Package Change. *J Nutr Educ Behav*. 2014;46(3):S79-S86. doi:10.1016/j.jneb.2014.02.005	Wrong Outcome(s)
322	Tohotoa J, Maycock B, Hauck Y, Howat P, Burns S, Binns CW. Supporting mothers to breastfeed: the development and process evaluation of a father inclusive perinatal education support program in Perth, Western Australia. *Health Promot Int*. 2010;26:351-361. doi:10.1093/heapro/daq077	Wrong Outcome(s)
323	Tussing-Humphreys L, Thomson JL, Hemphill NO, Goodman MH, Landry AS. Maternal weight in the postpartum: results from the Delta healthy sprouts trial. *Matern Health Neonatol Perinatol*. 2017;3:20-20. doi:10.1186/s40748-017-0058-9	Wrong Outcome(s)
324	Watkins EL, Larson K, Harlan C, Young S. A model program for providing health services for migrant farmworker mothers and children. *Public Health Rep*. 1990;105(6):567-75.	Wrong Outcome(s)
325	Yan J. Is WIC effective in improving pregnancy-related outcomes? An empirical reassessment. *Econ Hum Biol*. 2022;47:101197-101197. doi:10.1016/j.ehb.2022.101197	Wrong Outcome(s)
326	Actrn. Confident and understanding parents: child nutrition and active play intervention among highly disadvantaged families attending Supported Playgroups. http://wwwwhoint/trialsearch/Trial2aspx?TrialID=ACTRN12615000969561. 2015;	Wrong Population

327	Arvidsson L, Bogl L, Eiben G, et al. Fat, sugar and water intakes among families from the IDEFICS intervention and control groups: First observations from I.Family. *Obes Facts*. 2016;9:127. doi:10.1159/000446744	Wrong Population
328	Arvidsson L, Bogl LH, Eiben G, et al. Fat, sugar and water intakes among families from the IDEFICS intervention and control groups: first observations from I.Family. *Obes Rev*. 2015;16 Suppl 2:127-37. doi:10.1111/obr.12325	Wrong Population
329	Asfaw A. Do government food price policies affect the prevalence of obesity? Empirical evidence from Egypt. *World Development*. 2007;35(4):687-701. doi:10.1016/j.worlddev.2006.05.005	Wrong Population
330	Assis AM, Costa PR, da Silva Ma, et al. Effectiveness of the Brazilian Conditional Cash Transfer Program--Bolsa Alimentação--on the variation of linear and ponderal increment in children from northeast of Brazil. *Nutr Hosp*. 2015;31(6):2786-2794.	Wrong Population
331	Au LE, Lorrene DR, Martha M, Nila JR, Shannon W. Online and In-Person Nutrition Education Improves Breakfast Knowledge, Attitudes, and Behaviors: A Randomized Trial of Participants in the Special Supplemental Nutrition Program for Women, Infants, and Children. *J Acad Nutr Diet*. 2016;116(3):490-500. doi:10.1016/j.jand.2015.10.012	Wrong Population
332	Au LE, Whaley SE, Rosen NJ, Meza M, Ritchie LD. Online and In-Person Nutrition Education Improves Breakfast Knowledge, Attitudes, and Behaviors: A Randomized Trial of Participants in the Special Supplemental Nutrition Program for Women, Infants, and Children. *J Acad Nutr Diet*. 2015;116:490-500. doi:10.1016/j.jand.2015.10.012	Wrong Population
333	Bera A, Ghosh J, Singh AK, Hazra A, Mukherjee S, Mukherjee R. Effect of kangaroo mother care on growth and development of low birthweight babies up to 12 months of age: a controlled clinical trial. *Acta Paediatr*. 2014;103(6):643-50. doi:10.1111/apa.12618	Wrong Population
334	Bevier WC, Fischer R, Jovanovic L. Treatment of women with an abnormal glucose challenge test (but a normal oral glucose tolerance test) decreases the prevalence of macrosomia. *Am J Perinatol*. 1999;16(6):269-275. doi:10.1055/s-2007-993871	Wrong Population
335	Bohn CM, Haskins DD, Loo RK, Ahrendt LJ. Evaluation of the South Dakota fitCare child care provider training program targeting nutrition and physical activity. *S D Med*. 2014;67(8):305-13.	Wrong Population
336	Borys JM, Le Bodo Y, Jebb SA, et al. EPODE approach for childhood obesity prevention: methods, progress and international development. *Obes Rev*. 2012;13(4):299-315. doi:10.1111/j.1467-789X.2011.00950.x	Wrong Population
337	Caan B, Horgen DM, Margen S. Benefits associated with WIC supplemental feeding during the interpregnancy interval. *Am J Clin Nutr*. 1987;45(1):29-41. doi:10.1093/ajcn/45.1.29	Wrong Population
338	Carriere C, Thibault H, Barat P, et al. Short-term and long-term positive outcomes of the multidisciplinary care implemented by the French health networks for the prevention and care of paediatric overweight and obesity. *Pediatr Obes*. 2019;14(8):e12522. doi:10.1111/ijpo.12522	Wrong Population
339	Carrillo-Larco RM, Jaime Miranda J, Bernabé-Ortiz A. Impact of Food Assistance Programs on Obesity in Mothers and Children: A Prospective Cohort Study in Peru. *Am J Public Health*. 2016;106(7):1301-1307. doi:10.2105/AJPH.2016.303191	Wrong Population

340	Carson V, Zhang Z, Kuzik N, et al. The impact of new government childcare accreditation standards on children's in-care physical activity and sedentary time. *BMC Public Health*. 2022;22(616)doi:10.1186/s12889-022-12888-5	Wrong Population
341	Chang M, Brown R, Nitzke S. A community-based intervention program's effects on dietary intake behaviors. *Obes*. 2017;25(12):2055-2061. doi:10.1002/oby.21862	Wrong Population
342	Chang M-W, Nitzke S, Brown R, Resnicow K. A community based prevention of weight gain intervention (Mothers In Motion) among young low-income overweight and obese mothers: design and rationale. *BMC Public Health*. 2014;14(1):280-280. doi:10.1186/1471-2458-14-280	Wrong Population
343	Chaparro MP, Whaley SE, Anderson CE, Wang MC, Crespi CM. The role of income and neighbourhood poverty in the association between the 2009 Special Supplemental Nutrition Program for Women, Infants and Children (WIC) food package change and child obesity among WIC-participating children in Los Angeles County, 2003–2016. *Public Health Nutr*. 2021;24(13):4212-4219. doi:10.1017/S1368980020005200	Wrong Population
344	Chiasson M, Scheinmann R, Hartel D, et al. Predictors of Obesity in a Cohort of Children Enrolled in WIC as Infants and Retained to 3 Years of Age. *J. Community Health*. 2016;41(1):127-133. doi:10.1007/s10900-015-0077-2	Wrong Population
345	ChiCtr. SCHeLTI (Sino-Canadian Healthy Life Trajectory Initiative). http://wwwwhoint/trialsearch/Trial2aspx?TrialID=ChiCTR1800017773. 2018.	Wrong Population
346	Chorniy A, Currie J, Sonchak L. Does Prenatal WIC Participation Improve Child Outcomes? 2018;doi:10.3386/w24691	Wrong Population
347	Cotwright CJ, Bales DW, Jung Sun L, Parrott K, Celestin N, Olubajo B. Like Peas and Carrots: Combining Wellness Policy Implementation With Classroom Education for Obesity Prevention in the Childcare Setting. *Public Health Rep*. 2017;132:74S-80S. doi:10.1177/0033354917719706	Wrong Population
348	Ctri. Comparison of earlier and late onset kangaroo mother care in inmproving the breastfeeding. https://trialsearchwhoint/Trial2aspx?TrialID=CTRI/2013/12/004222. 2013.	Wrong Population
349	Ctri. Comparison of supplementation of preterm versus term donor pooled human milk on growth of very low birth weight neonates. https://trialsearchwhoint/Trial2aspx?TrialID=CTRI/2020/02/023569. 2020.	Wrong Population
350	Dehkhoda N, Valizadeh S, Jodeiry B, Hosseini MB. The effects of an educational and supportive relactation program on weight gain of preterm infants. *J Caring Sci*. 2013;2(2):97-103. doi:10.5681/jcs.2013.012	Wrong Population
351	Dennis CL, Marini F, Dick JA, et al. Protocol for a randomised trial evaluating a preconception-early childhood telephone-based intervention with tailored e-health resources for women and their partners to optimise growth and development among children in Canada: a Healthy Life Trajectory Initiative (HeLTI Canada). *BMJ Open*. 2021;11(2)doi:10.1136/bmjopen-2020-046311	Wrong Population
352	Dev DA, Williams N, Iruka I, et al. Improving the nutrition and screen time environment through self-assessment in family childcare homes in Nebraska. *Public Health Nutr*. 2018;21(13):2351-2359. doi:10.1017/s1368980018001416	Wrong Population

353	Dor F, Wright K, Dutton F, Willoughby R, Bird C. Tackling child inequality in a UK emergency department: a pilot early intervention service on the shop floor. *Arch Dis Child*. 2022;107:A22. doi:10.1136/archdischild-2022-rcpch.38	Wrong Population
354	Enö Persson J, Bohman B, Tynelius P, Rasmussen F, Ghaderi A. Prevention of Childhood Obesity in Child Health Services: Follow-Up of the PRIMROSE Trial. *Child Obes*. 2018;14(2):99-105. doi:10.1089/chi.2017.0117	Wrong Population
355	Farrell L, Lloyd B, Matthews R, Bravo A, Wiggers J, Rissel C. Applying a performance monitoring framework to increase reach and adoption of children's healthy eating and physical activity programs. *Public Health Res Pract*. 2014;25(1)doi:10.17061/phrp2511408	Wrong Population
356	Fernald LC, Gertler PJ, Neufeld LM. Role of cash in conditional cash transfer programmes for child health, growth, and development: an analysis of Mexico's Oportunidades. *Lancet*. 2008;371(9615):828-37. doi:10.1016/s0140-6736(08)60382-7	Wrong Population
357	Fernandez A, Lozano A, Lee T, Messiah SE, Prado G. A healthy lifestyle intervention for Hispanic families: moderating effects of education, income, nativity. *J Nutr Educ Behav*. 2022;54(2):125-134. doi:10.1016/j.jneb.2021.05.001	Wrong Population
358	Funkquist EL, Tuvemo T, Jonsson B, Serenius F, Nyqvist KH. Influence of test weighing before/after nursing on breastfeeding in preterm infants. *Adv Neonatal Care*. 2010;10(1):33-9. doi:10.1097/ANC.0b013e3181cbf910	Wrong Population
359	Funkquist EL, Tuvemo T, Jonsson B, Serenius F, Nyqvist KH. Milk for small infants. *Acta Paediatr*. 2007;96(4):596-9. doi:10.1111/j.1651-2227.2007.00222.x	Wrong Population
360	Garratt J, Norman S, Wong HL, et al. An audit to assess the efficacy of changes to nutritional practice on nutrition and growth outcomes in an australian tertiary neonatal unit. *J Pediatr Child Health*. 2015;51:15. doi:10.1111/jpc.12884	Wrong Population
361	Garvin TM, Chiappone A, Weissenburger-Moser Boyd L, Shuell J, Plumlee C, Yaroch AL. Effectiveness in adapting the implementation of the Early Care and Education Learning Collaboratives Project (ECELC) using real-world conditions. *Transl Behav Med*. 2021;11(1):56-63. doi:10.1093/tbm/ibz152	Wrong Population
362	Ginovart G, Gich I, Gutiérrez A, Verd S. A Fortified Donor Milk Policy is Associated With Improved In-Hospital Head Growth and Weight Gain in Very Low-Birth-Weight Infants. *Adv Neonatal Care*. 2017;17(4):250-257. doi:10.1097/anc.0000000000000387	Wrong Population
363	Gross SM, Augustyn M, Henderson JL, et al. Integrating Obstetrical Care and WIC Nutritional Services to Address Maternal Obesity and Postpartum Weight Retention. *Matern Child Health J*. 2018;22(6):794-802. doi:10.1007/s10995-018-2449-6	Wrong Population
364	Guan A, Batra A, Hamad R. Effects of the revised WIC food package on women's and children's health: a quasi-experimental study. *BMC Pregnancy Childbirth*. 2022;22(1):806. doi:10.1186/s12884-022-05116-w	Wrong Population
365	Gunn TR, Thompson JMD, Jackson H, McKnight S, Buckthought G, Gunn AJ. Does early hospital discharge with home support of families with preterm infants affect breastfeeding success? A randomized trial. *Acta Paediatr*. 2000;89(11):1358-1363. doi:10.1080/080352500300002570	Wrong Population

366	Haines J, Rifas-Shiman SL, Gross D, McDonald J, Kleinman K, Gillman MW. Randomized trial of a prevention intervention that embeds weight-related messages within a general parenting program. *Obes*. 2016;24(1):191-199. doi:10.1002/oby.21314	Wrong Population
367	Haire-Joshu D, Schwarz CD, Steger-May K, et al. A Randomized Trial of Weight Change in a National Home Visiting Program. *Am J Prev Med*. 2018;54(3):341-351. doi:10.1016/j.amepre.2017.12.012	Wrong Population
368	Haire-Joshu DL, Schwarz CD, Peskoe SB, Budd EL, Brownson RC, Joshu CE. A group randomized controlled trial integrating obesity prevention and control for postpartum adolescents in a home visiting program. *Int J Behav Nutr Phys Act*. 2015;12:88. doi:10.1186/s12966-015-0247-8	Wrong Population
369	Hamad R, Collin DF, Rehkopf DH. Estimating the Short-Term Effects of the Earned Income Tax Credit on Child Health. *Am J Epidemiol*. 2018;187:2633-2641. doi:10.1093/aje/kwy179	Wrong Population
370	Hargreaves MB, Honeycutt T, Orfield C, et al. The Healthy Weight Collaborative: using learning collaboratives to enhance community-based prevention initiatives addressing childhood obesity. *J Health Care Poor Underserved*. 2013;24:103-115. doi:10.1353/hpu.2013.0095	Wrong Population
371	Havas S, Damron D, Treiman K, et al. The Maryland WIC 5 a day promotion program pilot study: Rationale, results, and lessons learned. *J Nutr Educ*. 1997;29:343-350. doi:10.1016/s0022-3182(97)70249-8	Wrong Population
372	Helland SH, Bere E, Øverby NC. Study protocol for a multi-component kindergarten-based intervention to promote healthy diets in toddlers: a cluster randomized trial. *BMC Public Health*. 2016;16:273. doi:10.1186/s12889-016-2952-x	Wrong Population
373	Herring SJ, Bersani VM, Santoro C, McNeil SJ, Kilby LM, Bailer B. Feasibility of using a peer coach to deliver a behavioral intervention for promoting postpartum weight loss in Black and Latina mothers. *Transl Behav Med*. 2021;11(6):1226-1234. doi:10.1093/tbm/ibaa096	Wrong Population
374	Horton LA, Ayala GX, Slymen DJ, et al. A Mediation Analysis of Mothers' Dietary Intake: The Entre Familia: Reflejos de Salud Randomized Controlled Trial. *Health Educ Behav*. 2018;45(4):501-510. doi:10.1177/1090198117742439	Wrong Population
375	Hudak KM, Racine EF. Do additional SNAP benefits matter for child weight?: Evidence from the 2009 benefit increase. *Econ Hum Biol*. 2021;41:100966. doi:10.1016/j.ehb.2020.100966	Wrong Population
376	Hughes SO, Adair L, AnaMaria Diaz M, et al. Strategies for Effective Eating Development—SEEDS: Design of an Obesity Prevention Program to Promote Healthy Food Preferences and Eating Self-Regulation in Children From Low-Income Families. *J Nutr Educ Behav*. 2016;48(6):405. doi:10.1016/j.jneb.2016.04.388	Wrong Population
377	Irct2015122325668N. Effect of theory-based training on weight and knowledge of women about overweight before pregnancy. https://trialsearchwhoint/Trial2aspx?TrialID=IRCT2015122325668N1. 2016.	Wrong Population
378	Isrctn. A web-based program to reduce food-related fears and promote healthy dietary habits in toddlers. https://trialsearchwhoint/Trial2aspx?TrialID=ISRCTN98064772. 2017.	Wrong Population

379	Isrctn. Feasibility of a brief weight loss intervention for women following childbirth, delivered within the national child immunisation programme. https://trialsearchwhoint/Trial2aspx?TrialID=ISRCTN10460064. 2015.	Wrong Population
380	Isrctn. Preconception-early childhood telephone-based intervention to optimize growth and development among children in Canada: a Healthy Life Trajectory Initiative (HeLTI-Canada). https://trialsearchwhoint/Trial2aspx?TrialID=ISRCTN13308752. 2019.	Wrong Population
381	James KS, Connelly CD, Gracia L, Mareno N, Baietto J. Ways to Enhance Children's Activity and Nutrition (WE CAN) - a pilot project with Latina mothers. *J Spec Pediatr Nurs*. 2010;15(4):292-300. doi:10.1111/j.1744-6155.2010.00252.x	Wrong Population
382	Japakasetr S, Sirikulchayanonta C, Suthutvoravut U, Chindavijak B, Kagawa M, Nokdee S. Implementation of a Nutrition Program Reduced Post-Discharge Growth Restriction in Thai Very Low Birth Weight Preterm Infants. *Nutrients*. 2016;8(12)doi:10.3390/nu8120820	Wrong Population
383	Jo Y, Wang Q. The impact of maternal employment on children's adiposity: Evidence from China's labor policy reform. *Health Econ*. 2017;26(12):e236-e255. doi:10.1002/hec.3498	Wrong Population
384	Johnson MJ, Leaf AA, Pearson F, et al. Successfully implementing and embedding guidelines to improve the nutrition and growth of preterm infants in neonatal intensive care: a prospective interventional study. *BMJ Open*. 2017;7(12):e017727. doi:10.1136/bmjopen-2017-017727	Wrong Population
385	Jones LJ, VanWassenhove-Paetzold J, Thomas K, et al. Impact of a fruit and vegetable prescription program on health outcomes and behaviors in young navajo children. *Curr Dev Nutr*. 2020;4(8)doi:10.1093/cdn/nzaa109	Wrong Population
386	Kain J, Vio F, Albala C. Childhood nutrition in Chile: From deficit to excess. *Nutr Res*. 1998;18(11):1825-1837. doi:10.1016/S0271-5317(98)00152-3	Wrong Population
387	Keller C, Ainsworth B, Records K, et al. A comparison of a social support physical activity intervention in weight management among post-partum Latinas. *BMC Public Health*. 2014;14:971. doi:10.1186/1471-2458-14-971	Wrong Population
388	Khanal S, Welsby D, Lloyd B, Innes-Hughes C, Lukeis S, Rissel C. Effectiveness of a once per week delivery of a family-based childhood obesity intervention: a cluster randomised controlled trial. *Pediatr Obes*. 2016;11(6):475-483. doi:10.1111/ijpo.12089	Wrong Population
389	Kong A, Odoms-Young AM, Schiffer LA, et al. The 18-month impact of special supplemental nutrition program for women, infants, and children food package revisions on diets of recipient families. *Am J Prev Med*. 2014;46(6):543-551. doi:10.1016/j.amepre.2014.01.021	Wrong Population
390	Kumaran K, Krishnaveni GV, Suryanarayana KG, et al. Protocol for a cluster randomised trial evaluating a multifaceted intervention starting preconceptionally-Early Interventions to Support Trajectories for Healthy Life in India (EINSTEIN): a Healthy Life Trajectories Initiative (HeLTI) Study. *BMJ Open*. 2021;11(2)doi:10.1136/bmjopen-2020-045862	Wrong Population
391	Lakin B, Grime C, Guratsky V, Brearey S. Retrospective neonatal nutrition audit- Calorie counting counts. *Intensive Care Medicine*. 2011;37:S392. doi:10.1007/s00134-011-2387-x	Wrong Population
392	Lebihan L, Takongmo COM. Unconditional cash transfers and parental obesity. *Social Science & Medicine*. 2019;224:116-126.	Wrong Population

393	Levene I, McCormick K. Improved growth of extremely and very preterm babies: Evaluation of a quality-of-care initiative. *J Paediatr Child Health*. 2020;56(3):444-449. doi:10.1111/jpc.14662	Wrong Population
394	Liu ST, Graffagino CL, Leser KA, Trombetta AL, Pirie PL. Obesity Prevention Practices and Policies in Child Care Settings Enrolled and Not Enrolled in the Child and Adult Care Food Program. *Matern Child Health J*. 2016;20(9):1933-9. doi:10.1007/s10995-016-2007-z	Wrong Population
395	Lizarazo-Medina JP, Ospina-Diaz JM, Ariza-Riaño NE. The Kangaroo Mothers' programme: A simple and cost-effective alternative for protecting the premature newborn or low-birth-weight babies. *Revista de Salud Publica*. 2013;14(2 SUPPL.):32-45.	Wrong Population
396	Markides BR, Courtney T, Lesli B-F, Sylvia Hurd C. Staff Workshop Improves Child Care Center Menus in South Central Texas: A Best Food for Families, Infants, and Toddlers (Best Food FITS) Intervention. *J Nutr Educ Behav*. 2017;49(5):435. doi:10.1016/j.jneb.2017.02.002	Wrong Population
397	Mayo C. Parent Satisfaction with Feeding Competence in Preterm Infants: Infant-Driven Feeding Compared with Standard Scheduled Feeding. *Ochsner J*. 2019;19(1):e12. doi:10.31486/toj.19.5007	Wrong Population
398	Mei-Wei C, Brown R, Nitzke S, Chang M-W. Results and lessons learned from a prevention of weight gain program for low-income overweight and obese young mothers: Mothers In Motion. *BMC Public Health*. 2017;17(1):1-12. doi:10.1186/s12889-017-4109-y	Wrong Population
399	Moore LH, Gott K, Ahrens L, Garrod A, List R, Froh D. A pilot telehealth program for remote home weight and nutrition monitoring for infants with cystic fibrosis. *Pediatric Pulmonology*. 2020;55(SUPPL 2):127.	Wrong Population
400	Murray N. Effects of implememtation of infant driven feeding (IDF) protocols which included pre and post breast feeding weights in 35 week gestational age infants in the neonatal intensive care unit (NICU). *J Pediatr Gastroenterol Nutr*. 2017;65:S344. doi:10.1097/MPG.0000000000001805	Wrong Population
401	Nct. A Pilot Study of Diabetes Risk Reduction Program With WIC Mothers(The Special Supplemental Nutrition Program for Women, Infants, and Children) Overweight WIC Mothers. https://clinicaltrialsgov/show/NCT00944060. 2009;	Wrong Population
402	Nct. Achieving Energy Balance in Post Partum Teens. https://clinicaltrialsgov/show/NCT01617486. 2012.	Wrong Population
403	Nct. An Ecological Approach to Addressing Childhood Obesity Issues in Imperial County. https://clinicaltrialsgov/show/NCT02197390. 2014.	Wrong Population
404	Nct. Educational Intervention in the First 18 Months of Life to Prevent/Manage Obesity of Children After Gestational Diabetes. https://clinicaltrialsgov/show/NCT04493177. 2020.	Wrong Population
405	Nct. Healthy Eating and Active Living Taught at Home (HEALTH) Dissemination & Implementation (D&I). https://clinicaltrialsgov/show/NCT03758638. 2018.	Wrong Population
406	Nct. Helping Moms to be Healthy After Baby. https://clinicaltrialsgov/show/NCT03257657. 2017.	Wrong Population
407	Nct. Mothers in Motion Program to Prevent Weight Gain in WIC Mothers (MIM). https://clinicaltrialsgov/show/NCT01839708. 2013.	Wrong Population
408	Nct. Online Videos and New Feeding Content to Enhance a Current EFNEP Program. https://clinicaltrialsgov/show/NCT03170700. 2017.	Wrong Population

409	Nct. PALS-CATCH Intervention for Obesity Prevention Among At-risk Toddlers. https://clinicaltrialsgov/show/NCT02739334. 2016.	Wrong Population
410	Nct. Postpartum Weight Retention. https://clinicaltrialsgov/show/NCT02867631. 2015.	Wrong Population
411	Nct. Promoting Healthy Development With the Recipe 4 Success Intervention. https://clinicaltrialsgov/show/NCT03976089. 2019.	Wrong Population
412	Nct. REACH-OUT: Chicago Children's Diabetes Prevention Program. https://clinicaltrialsgov/show/NCT00723853. 2008.	Wrong Population
413	Nct. Third Ward Wellness Study for Families. https://clinicaltrialsgov/show/NCT03590301. 2018.	Wrong Population
414	Nicklas TA, O'Neil CE, Fulgoni VL. Evaluating the Impact of the Revised Special Supplemental Nutrition Program for Women, Infants, and Children Fruit Juice Allotment on Fruit Intake, Dietary Quality, and Energy/Nutrient Intakes among Children 1-4 Years of Age. *Int J Child Health Nutr*. 2018;7(4):146-156. doi:10.6000/1929-4247.2018.07.04.3	Wrong Population
415	Ntr. Computer-tailored health information for parents with young children combined with personal counselling in well-child care. https://trialsearchwhoint/Trial2aspx?TrialID=NTR616. 2006.	Wrong Population
416	Oberle MM, Freese R, Shults J, Stallings VA, Virudachalam S. Impact of the 2009 WIC Food Package Changes on Maternal Dietary Quality. *J Hunger Environ Nutr*. 2020;15:739-752. doi:10.1080/19320248.2020.1724227	Wrong Population
417	O'Brien K, Robson K, Bracht M, et al. Effectiveness of Family Integrated Care in neonatal intensive care units on infant and parent outcomes: a multicentre, multinational, cluster-randomised controlled trial. *Lancet Child Adolesc Health*. 2018;2(4):245-254. doi:10.1016/s2352-4642(18)30039-7	Wrong Population
418	Olaya G, Buitrago MF, Fewtrell M. Randomised trial testing new complementary feeding guidelines: Effects on food consumption and growth at 6 years of age. *J Pediatr Gastroenterol Nutr*. 2018;66:1160.	Wrong Population
419	Oltmann L, Bauer L, Gauthier K, Savage R, May KL. KOALA (Kangaroo Oriented Ad Lib Advancement of Breastfeeding) Blocks: Creating a bridge to breastfeeding. *JPEN J Parenter Enteral Nutr*. 2021;45(SUPPL 1):S211-S213. doi:10.1002/jpen.2095	Wrong Population
420	One-Year Efficacy Testing of Enabling Mothers to Prevent Pediatric Obesity Through Web-Based Education and Reciprocal Determinism (EMPOWER) Randomized Control Trial. *Health Educ Behav*. 43 (1) (pp 94-106), 2016 Date of publication: 01 feb 2016. 2016;doi:10.1177/1090198115596737	Wrong Population
421	Ong T, Nay L, Casey SC, et al. Grams to grow quality initiative: Systemlevel improvements to approach CF infant nutrition in a single center. *Pediatric Pulmonology*. 2018;53:363. doi:10.1002/ppul.24152	Wrong Population
422	Østbye T, Mann CM, Vaughn AE, et al. The keys to healthy family child care homes intervention: study design and rationale. *Contemp Clin Trials.* 2015;40:81-9. doi:10.1016/j.cct.2014.11.003	Wrong Population

423	Palencia D, Mendoza CJ, Torres J, Echandía CA. Kangaroo mother program: Physical growth and morbidity in a cohort of children, followed from 40 weeks of postconceptional age until first year. *Colomb Med (Cali)*. 2009;40(3):292-299.	Wrong Population
424	Paratmanitya Y, Helmyati S, Nurdiati DS, Lewis EC, Gittelsohn J, Hadi H. The effect of a maternal mentoring program on the timing of first antenatal care visit among pregnant women in Bantul, Indonesia: Results of a cluster randomized trial. *Health Promot Perspect*. 2021;11(3):307-315. doi:10.34172/hpp.2021.39	Wrong Population
425	Parretti HM, Ives NJ, Tearne S, et al. Protocol for the feasibility and acceptability of a brief routine weight management intervention for postnatal women embedded within the national child immunisation programme: randomised controlled cluster feasibility trial with nested qualitative study (PIMMS-WL). *BMJ Open*. 2020;10(2):e033027. doi:10.1136/bmjopen-2019-033027	Wrong Population
426	Perkins S, Daley A, Yerxa K, Therrien M. The Effectiveness of the Expanded Food and Nutrition Education Program (EFNEP) on Diet Quality as Measured by the Healthy Eating Index. *Am J Lifestyle Med*. 2020;14(3):316-325. doi:10.1177/1559827619872733	Wrong Population
427	Persson JE, Bohman B, Tynelius P, Rasmussen F, Ghaderi A. Prevention of Childhood Obesity in Child Health Services: Follow-Up of the PRIMROSE Trial. *Child Obes*. 2017;14:99-105. doi:10.1089/chi.2017.0117	Wrong Population
428	Peters P, Gold A, Abbott A, et al. A quasi-experimental study to mobilize rural low-income communities to assess and improve the ecological environment to prevent childhood obesity. *BMC Public Health*. 2016;16:376. doi:10.1186/s12889-016-3047-4	Wrong Population
429	Peterson KE, Sorensen G, Pearson M, Hebert JR, Gottlieb BR, McCormick MC. Design of an intervention addressing multiple levels of influence on dietary and activity patterns of low-income, postpartum women. *Health Educ Res*. 2002;17(5):531-40. doi:10.1093/her/17.5.531	Wrong Population
430	Powell C, Baker-Henningham H, Walker S, Gernay J, Grantham-McGregor S. Feasibility of integrating early stimulation into primary care for undernourished Jamaican children: cluster randomised controlled trial. *BMJ*. 2004;329(7457):89-91. doi:10.1136/bmj.38132.503472.7C	Wrong Population
431	Powell JM, Hersh AR, Clark GV, Valent AM. Perinatal outcomes among women with gestational diabetes receiving WIC benefits. *Am J of Obstet Gynecol*. 2022;226(1):S458. doi:10.1016/j.ajog.2021.11.760	Wrong Population
432	Puma JE, Thompson D, Baer K, et al. Enhancing Periconceptional Health by Targeting Postpartum Mothers at Rural WIC Clinics. *Health Promot Pract*. 2018;19(3):390-399. doi:10.1177/1524839917699553	Wrong Population
433	Rao PNS, Rekha U, Ruchi N. Kangaroo mother care for low birth weight infants: a randomized controlled trial. *Indian Pediatr*. 2008;45(1):17-23.	Wrong Population
434	Ricketts SA, Murray EK, Schwalberg R. Reducing low birthweight by resolving risks: results from Colorado's prenatal plus program. *Am J Public Health*. 2005;95(11):1952-7. doi:10.2105/ajph.2004.047068	Wrong Population
435	Saha C, Nambiar VS. Improving Food and Nutrition Security by Public-Private Partnership Rural Households. 2019;(28151862):334.	Wrong Population

436	Sajn TM, Andresen PA, Mitchell JW. 5-4-3-2-1 Go! Standardizing Nutrition and Physical Activity-Related Counseling. *J Nurse Pract*. 2020;16(10):e173-e175. doi:10.1016/j.nurpra.2020.06.026	Wrong Population
437	Salzedo S, Perez Y, Brust L, Akong K, Lim M. A quality improvement project to improve the growth trends of young childen (0-24 months old) and preschool chldren (2-5.9 years old) with cystic fibrosis. *Pediatric Pulmonology*. 2017;52:428. doi:10.1002/ppul.23840	Wrong Population
438	Samburu BM, Young SL, Wekesah FM, et al. Effectiveness of the baby-friendly community initiative in promoting exclusive breastfeeding among HIV negative and positive mothers: a randomized controlled trial in Koibatek Sub-County, Baringo, Kenya. *Int Breastfeed J*. 2020;15(1):13. doi:10.1186/s13006-020-00299-4	Wrong Population
439	Samra NM, El Taweel A, Cadwell K. Effect of Intermittent Kangaroo Mother Care on Weight Gain of Low Birth Weight Neonates With Delayed Weight Gain. *J Perinat Educ*. 2013;22(4):194-200. doi:10.1891/1058-1243.22.4.194	Wrong Population
440	Sayyari AA, Abdollahi Z, Ziaodini H, et al. Methodology of the comprehensive program on prevention and control of overweight and obesity in iranian children and adolescents: The Iran-ending childhood obesity (Iran-ECHO) program. *Int J Prev Med*. 2017;8doi:10.4103/ijpvm.IJPVM_426_17	Wrong Population
441	Sekhobo JP, Edmunds LS, Dalenius K, et al. Neighborhood disparities in prevalence of childhood obesity among low-income children before and after implementation of New York City child care regulations. *Prev Chronic Dis*. 2014;11:E181. doi:10.5888/pcd11.140152	Wrong Population
442	Shah NB, Fenick AM, Rosenthal MS. A Healthy Weight for Toddlers? Two-Year Follow-up of a Randomized Controlled Trial of Group Well-Child Care. *Clin Pediatr*. 2016;55:1354-1357. doi:10.1177/0009922815623230	Wrong Population
443	Shilts MK, Rios LKD, Panarella KH, et al. Feasibility of colocating a nutrition education program into a medical clinic setting to facilitate pediatric obesity prevention. *J Prim Care Community Health*. 2021;12doi:10.1177/21501327211009695	Wrong Population
444	Smith TM, Blaser C, Geno-Rasmussen C, et al. Improving Nutrition and Physical Activity Policies and Practices in Early Care and Education in Three States, 2014-2016. *Prev Chronic Dis*. 2017;14:E73. doi:10.5888/pcd14.160513	Wrong Population
445	Tabak RG, Schwarz CD, Kemner A, et al. Disseminating and implementing a lifestyle-based healthy weight program for mothers in a national organization: a study protocol for a cluster randomized trial. *Implement Sci*. 2019;14(1):68. doi:10.1186/s13012-019-0916-0	Wrong Population
446	Tomayko EJ, Prince RJ, Cronin KA, et al. Healthy Children, Strong Families 2: A randomized controlled trial of a healthy lifestyle intervention for American Indian families designed using community-based approaches. *Clin Trials*. 2017;14:152-161. doi:10.1177/1740774516685699	Wrong Population
447	Towey M, Harrell R, Lee B. Evaluation of "One Body, One Life": A Community-Based Family Intervention for the Prevention of Obesity in Children. *J Obes*. 2011;2011:1-14. doi:10.1155/2011/619643	Wrong Population

448	van Grieken A, Vlasblom E, Lu W, et al. Personalized Web-Based Advice in Combination With Well-Child Visits to Prevent Overweight in Young Children: Cluster Randomized Controlled Trial. *J Med Internet Res*. 2017;19(7):1-15. doi:10.2196/jmir.7115	Wrong Population
449	Ward DS, Benjamin SE, Ammerman AS, Ball SC, Neelon BH, Bangdiwala SI. Nutrition and physical activity in child care: results from an environmental intervention. *Am J Prev Med*. 2008;35(4):352-6. doi:10.1016/j.amepre.2008.06.030	Wrong Population
450	Wen LM, Baur LA, Simpson JM, et al. Sustainability of Effects of an Early Childhood Obesity Prevention Trial Over Time: A Further 3-Year Follow-up of the Healthy Beginnings Trial. *JAMA Pediatr*. 2015;169(6):543-51. doi:10.1001/jamapediatrics.2015.0258	Wrong Population
451	White-Traut RC, Rankin KM, Yoder JC, et al. Influence of H-HOPE intervention for premature infants on growth, feeding progression and length of stay during initial hospitalization. *J Perinatol*. 2015;35(8):636-41. doi:10.1038/jp.2015.11	Wrong Population
452	Wilken LR, Novotny R, Fialkowski MK, et al. Children's Healthy Living (CHL) Program for remote underserved minority populations in the Pacific region: rationale and design of a community randomized trial to prevent early childhood obesity. *BMC Public Health*. 2013;13:944. doi:10.1186/1471-2458-13-944	Wrong Population
453	Woo Baidal JA, Meyer D, Partida I, et al. Feasibility of Food FARMacia: Mobile Food Pantry to Reduce Household Food Insecurity in Pediatric Primary Care. *Nutrients*. 2022;14(5)doi:10.3390/nu14051059	Wrong Population
454	Wu Y, Marc I, Bouchard L, et al. Study protocol for the Sino-Canadian Healthy Life Trajectories Initiative (SCHeLTI): a multicentre, cluster-randomised, parallel-group, superiority trial of a multifaceted community-family-mother-child intervention to prevent childhood overweight and obesity. *BMJ Open*. 2021;11(4):e045192. doi:10.1136/bmjopen-2020-045192	Wrong Population
455	Xu T, Lai X, He K, Ma L, Fang H. Subsidy programme for gestational diabetes mellitus screening and lifestyle management in rural areas of western China: a study protocol for a multicentre randomised controlled trial. *BMJ Open*. 2021;11(7):e045503. doi:10.1136/bmjopen-2020-045503	Wrong Population
456	Yu J, Wei Z, Wells J, Fewtrell M. Effects of relaxation therapy on maternal psychological status and infant growth following late pre-term and early term delivery: Data from a randomised controlled trial. *J Pediatr Gastroenterol Nutr*. 2021;72(SUPPL 1):1216. doi:10.1097/MPG.0000000000003177	Wrong Population
457	Yuan K, Wang H, Chen Y, et al. A 12-hour comprehensive nutrition care benefits blood glucose level and weight gain and improves outcomes in pregnant women with gestational diabetes mellitus. *Ann Palliat Med*. 2020;9(3):661-670. doi:10.21037/apm.2020.03.16	Wrong Population
458	Anderson CE, Whaley SE, Crespi CM, Wang MC, Chaparro MP. Every month matters: longitudinal associations between exclusive breastfeeding duration, child growth and obesity among WIC-participating children. *J Epidemiol Community Health*. 2020;74(10):785-791. doi:10.1136/jech-2019-213574	Wrong Setting
459	Au LE, Paolicelli C, Gurzo K, et al. Contribution of WIC-Eligible Foods to the Overall Diet of 13- and 24-Month-Old Toddlers in the WIC Infant and Toddler Feeding Practices Study-2. *J Acad Nutr Diet*. 2019;119:435-448. doi:10.1016/j.jand.2018.11.001	Wrong Setting

460	Ball R, Vaschak R, Bailey A, et al. Study Protocol of the Parents in Child Nutrition Informing Community (PICNIC) Peer Education Cohort Study to Improve Child Feeding and Dietary Intake of Children Aged Six Months to Three Years Old. Children-Basel. 2020;7(1):14. doi:10.3390/children7010003	Wrong Setting
461	Bonuck KA, Huang V, Fletcher J. Inappropriate bottle use: an early risk for overweight? Literature review and pilot data for a bottle-weaning trial. *Matern Child Nutr*. 2010;6(1):38-52. doi:10.1111/j.1740-8709.2009.00186.x	Wrong Setting
	Bonuck K, Avraham SB, Richard Kahn Yl, Hyden C. Bottle-weaning intervention and toddler overweight. *J Pediatr*. 2014;164(2):306-312.e2. doi:10.1016/j.jpeds.2013.09.029	Wrong Setting
	Nct. Feeding Young Children Study: bottle Weaning Intervention. https://clinicaltrialsgov/show/NCT00756626. 2008.	Wrong Setting
462	Boulvain M, Perneger TV, Othenin-Girard V, Petrou S, Berner M, Irion O. Home-based versus hospital-based postnatal care: a randomised trial. *BJOG*. 2004;111:807-813. doi:10.1111/j.1471-0528.2004.00227.x	Wrong Setting
463	Bradley PJ. *The Impact of Care Coordination Provided to Low-Income Pregnant Women in an Inner City Neighborhood*. [Doctoral dissertation]. Bloomington, IN: Indiana University School of Nursing; 1993.	Wrong Setting
464	Brownell M, Chartier M, Nickel NC, et al. Unconditional Prenatal Income Supplement and Birth Outcomes. *Pediatrics*. 2016;137doi:10.1542/peds.2015-2992	Wrong Setting
	Brownell M, Nickel NC, Chartier M, et al. An Unconditional Prenatal Income Supplement Reduces Population Inequities In Birth Outcomes. *Health Aff*. 2018;37(3):447-455. doi:10.1377/hlthaff.2017.1290	Wrong Setting
465	Bullinger LR, Gurley-Calvez T. WIC participation and maternal behavior: breastfeeding and work life. *Contemp Econ Policy*. 2016;34(1):158-172. doi:10.1111/coep.12123	Wrong Setting
466	Cahill AG, Haire-Joshu D, Cade WT, Stein RI, Woolfolk CL, Klein S. Randomized controlled trial of home-based weight management therapy in pregnant socioeconomically disadvantaged (SED) women with overweight/obesity. *Am J Obstet Gynecol*. 2017;216(1):S56-S57.	Wrong Setting
	Cahill AG, Haire-Joshu D, Cade WT, et al. Weight control program and gestational weight gain in disadvantaged women with overweight or obesity: a randomized clinical trial. *Obesity*. 2018;26(3):485-491. doi:10.1002/oby.22070	Wrong Setting
	Haire-Joshu D, Cahill AG, Stein RI, et al. Randomized controlled trial of home-based lifestyle therapy on postpartum weight in underserved women with overweight or obesity. *Obesity*. 2019;27(4):535-541. doi:10.1002/oby.22413	Wrong Setting
	Lewkowitz AK, Rhaodes JS, López JD, et al. Does a home-based lifestyle intervention improve breastfeeding rates in socioeconomically disadvantaged (SED) obese women? *Am J Obstet Gynecol*. 2018;218(1):S530.	Wrong Setting

467	Chaparro MP, Anderson CE, Crespi CM, Whaley SE, Wang MC. The effect of the 2009 WIC food package change on childhood obesity varies by gender and initial weight status in Los Angeles County. *Pediatr Obes*. 2019;14(9):e12526. doi:10.1111/ijpo.12526	Wrong Setting
	Chaparro MP, Wang MC, Anderson CE, Crespi CM, Whaley SE. The Association between the 2009 WIC Food Package Change and Early Childhood Obesity Risk Varies by Type of Infant Package Received. *J Acad Nutr Diet*. 2020;120(3):371-385. doi:10.1016/j.jand.2019.09.014	Wrong Setting
	Chaparro MP, Anderson CE, Crespi CM, Wang MC, Whaley SE. The new child food package is associated with reduced obesity risk among formula fed infants participating in the Special Supplemental Nutrition Program for Women, Infants and Children (WIC) in Los Angeles County, California, 2003–2016. *Int J Behav Nutr Phys Act*. 2020;17(1):18-18. doi:10.1186/s12966-020-0921-3	Wrong Setting
	Chaparro MP, Crespi CM, Anderson CE, Wang MC, Whaley SE. The 2009 Special Supplemental Nutrition Program for Women, Infants, and Children (WIC) food package change and children's growth trajectories and obesity in Los Angeles County. *Am J Clin Nutr*. 2019;109(5):1414-1421. doi:10.1093/ajcn/nqy347	Wrong Setting
468	Pia Chaparro M, Crespi CM, Anderson CE, Wang MC, Whaley SE. The 2009 special supplemental nutrition program for women, infants, and children (WIC) food package change and children's growth trajectories and obesity in Los Angeles County. *Am J Clin Nutr*. 2019;109(5):1414-1421. doi:10.1093/ajcn/nqy347	Wrong Setting
469	Chiasson MA, Findley SE, Sekhobo JP, et al. Changing WIC changes what children eat. *Obesity (Silver Spring)*. 2013;21(7):1423-1429. doi:10.1002/oby.20295	Wrong Setting
470	Cloutier MM, Wiley JF, Kuo CL, Cornelius T, Wang Z, Gorin AA. Outcomes of an early childhood obesity prevention program in a low-income community: a pilot, randomized trial. *Pediatr Obes*. 2018;13(11):677-685. doi:10.1111/ijpo.12458	Wrong Setting
	Cloutier MM, Wiley J, Wang Z, Grant A, Gorin AA. The Early Childhood Obesity Prevention Program (ECHO): an ecologically-based intervention delivered by home visitors for newborns and their mothers. *BMC Public Health*. 2015;15:584. doi:10.1186/s12889-015-1897-9	Wrong Setting
471	Condie R, Murano PS. *Assessing the Revised Special Supplemental Nutrition Program for Women, Infants, And Children (WIC) Food Packages by Surveying Native American WIC Participants and Administering a Metabolic, Dietary Study of the Revised and Original WIC Food Packages*. [Doctoral dissertation]. College Station, TX: Texas A&M; 2011.	Wrong Setting
472	Davis AM, Gallagher K, Taylor M, et al. An in-home intervention to improve nutrition, physical activity, and knowledge among low-income teen mothers and their children: results from a pilot study. *J Dev Behav Pediatr*. 2013;34(8):609-615. doi:10.1097/DBP.0b013e3182a509df	Wrong Setting
473	de la Haye K, Bell BM, Salvy SJ. The role of maternal social networks on the outcomes of a home-based childhood obesity prevention pilot intervention. *J Soc Struct*. 2019;20(3):7-28. doi:10.21307/joss-2019-004	Wrong Setting
	de la Haye K, Fluke M, Laney PC, et al. In-home obesity prevention in low-income infants through maternal and social transmission. *Contemp Clin Trials*. 2019;77:61-69. doi:10.1016/j.cct.2018.12.010	Wrong Setting
	Nct. In-home Obesity Prevention to Reach Low-income Infants. https://clinicaltrialsgov/show/NCT03529695. 2018.	Wrong Setting

Supplementary Data

474	Edmunds LS, Sekhobo JP, Dennison BA, Chiasson MA, Stratton HH, Davison KK. Association of prenatal participation in a public health nutrition program with healthy infant weight gain. *Am J Public Health*. 2014;104 Suppl 1(Suppl 1):S35-42. doi:10.2105/ajph.2013.301793	Wrong Setting
	Edmunds LS, Davison KK, Stratton HH. *Prenatal Participation in WIC: Impact on Breastfeeding Initiation and Duration and on Infant Weight Gain*. [Doctoral dissertation]. Albany, NY: University at Albany; 2012.	Wrong Setting
475	Edwards RC, Thullen MJ, Korfmacher J, Lantos JD, Henson LG, Hans SL. Breastfeeding and Complementary Food: Randomized Trial of Community Doula Home Visiting. *Pediatrics*. 2013;132:S160-S166. doi:10.1542/peds.2013-1021P	Wrong Setting
476	Enns J, Nickel NC, Chartier M, et al. An unconditional prenatal income supplement is associated with improved birth and early childhood outcomes among First Nations children in Manitoba, Canada: a population-based cohort study. *BMC Pregnancy Childbirth*. 2021;21:312-312. doi:10.1186/s12884-021-03782-w	Wrong Setting
477	Goodman MH, Tussing-Humphreys LM, Thomson JL. StartSmart: A randomized intervention to promote maternal weight control and reduce childhood obesity in the Mississippi Delta. *FASEB J*. 2013;27.	Wrong Setting
	Nct. Delta Healthy Sprouts: intervention to Promote Maternal Weight Control and Reduce Childhood Obesity in the MS Delta. https://clinicaltrialsgov/show/NCT01746394. 2012.	Wrong Setting
	Thomson JL, Goodman MH, Tussing-Humphreys LM, Landry AS. Infant growth outcomes from birth to 12 months of age: findings from the Delta Healthy Sprouts randomized comparative impact trial. *Obes Sci Pract*. 2018;4(4):299-307. doi:10.1002/osp4.272	Wrong Setting
	Thomson JL, Tussing-Humphreys LM, Landry AS, Goodman MH. No Improvements in Postnatal Dietary Outcomes Were Observed in a Two-Arm, Randomized, Controlled, Comparative Impact Trial among Rural, Southern, African-American Women. *J Acad Nutr Diet*. 2018;118(7):1196-1207. doi:10.1016/j.jand.2017.11.010	Wrong Setting
	Thomson J, Tussing-Humphreys LM, Goodman MH, Landry AS. Delta Healthy sprouts: Participants' postnatal diet quality and food habits. *FASEB J*. 2017;31(1)	Wrong Setting
	Thomson JL, Goodman MH, Olender SE, Tussing-Humphreys LM. Gestational Weight Gain: Results from the Delta Healthy Sprouts Comparative Impact Trial. *J Pregnancy*. 2016;2016:1-12. doi:10.1155/2016/5703607 https://handle.nal.usda.gov/10113/5425115	Wrong Setting
	Thomson JL, Goodman MH, Tussing-Humphreys LM. Delta Healthy Sprouts: A randomized comparative effectiveness trial to promote maternal weight control and reduce childhood obesity in the Mississippi Delta. *Contemp Clin Trials*. 2014;38(1):82-91. doi:10.1016/j.cct.2014.03.004 https://handle.nal.usda.gov/10113/61920	Wrong Setting
	Tussing-Humphreys L, Thomson JL, Goodman MH, Olender S. Maternal diet quality and nutrient intake in the gestational period: results from the delta healthy sprouts comparative impact trial. *Matern Health Neonatol Perinatol*. 2016;2:8-8. doi:10.1186/s40748-016-0036-7	Wrong Setting
	Tussing-Humphreys L, Thomson JL, Goodman M, Landry A. Enhanced vs Standard Parents as Teacher Curriculum on Factors Related to Infant Feeding among African American Women. *South Med J*. 2019;112(10):512-519. doi:10.14423/smj.0000000000001024	Wrong Setting

478	Gross SM, Resnik AK, Cross-Barnet C, Nanda J, Augustyn M, Paige DM. The Differential Impact of WIC Peer Counseling Programs on Breastfeeding Initiation across the State of Maryland. *J Hum Lact*. 2009;25:435-443. doi:10.1177/0890334409342070	Wrong Setting
479	Guillory C. Honey child new beginnings: A faith-based initiative increasing breastfeeding in African American women. *Pediatrics*. 2019;144(2)doi:10.1542/peds.144.2-MeetingAbstract.261	Wrong Setting
	Guillory C, Livingston E. Honey child new beginnings: Combating the trickle down effects of maternal. *Pediatrics*. 2021;147(3):190-191. doi:10.1542/peds.147.3_MeetingAbstract.190	Wrong Setting
480	Guthrie JF, Anater A, Hampton JC, et al. The Special Supplemental Nutrition Program for Women, Infants, and Children is Associated with Several Changes in Nutrient Intakes and Food Consumption Patterns of Participating Infants and Young Children, 2008 Compared with 2016. *J Nutr*. 2020;150:2985-2993. doi:10.1093/jn/nxaa265	Wrong Setting
481	Haider R, Ashworth A, Kabir I, Huttly S. Effect of community-based peer counsellors on exclusive breastfeeding practices in Dhaka, Bangladesh: a randomised controlled trial. *Lancet*. 2000;356:1643-1647. doi:10.1016/s0140-6736(00)03159-7	Wrong Setting
482	Hamad R, Batra A, Karasek D, et al. The Impact of the Revised WIC Food Package on Maternal Nutrition During Pregnancy and Postpartum. *Am. J. Epidemiol*. 2019;188(8):1493-1502. doi:10.1093/aje/kwz098	Wrong Setting
483	Hamad R, Collin DF, Baer RJ, Jelliffe-Pawlowski LL. Association of Revised WIC Food Package with Perinatal and Birth Outcomes: A Quasi-Experimental Study. *Obstet Gynecol Surv*. 2020;75(2):77-78. doi:10.1097/OGX.0000000000000773	Wrong Setting
	Hamad R, Collin DF, Baer RJ, Jelliffe-Pawlowski LL. Association of Revised WIC Food Package With Perinatal and Birth Outcomes: A Quasi-Experimental Study. *JAMA Pediatr*. 2019;173(9):845-852. doi:10.1001/jamapediatrics.2019.1706	Wrong Setting
484	Hans SL, Edwards RC, Zhang Y. Randomized Controlled Trial of Doula-Home-Visiting Services: Impact on Maternal and Infant Health. *Matern Child Health J*. 2018;22:105-113. doi:10.1007/s10995-018-2537-7	Wrong Setting
485	Harvey-Berino J, Rourke J. Obesity prevention in preschool native-american children: a pilot study using home visiting. *Obes Res*. 2003;11(5):606-11. doi:10.1038/oby.2003.87	Wrong Setting
486	Herman D, Harrison GG, Afifi AA, Jenks E. Effect of a targeted subsidy on intake of fruits and vegetables among low-income women in the Special Supplemental Nutrition Program for Women, Infants, and Children. *Am J Public Health*. 2007;98:98-105. doi:10.2105/ajph.2005.079418	Wrong Setting
487	Jacknowitz A, Novillo D, Tiehen L. Special Supplemental Nutrition Program for Women, Infants, and Children and infant feeding practices. *Pediatrics*. 2007;119:281-289. doi:10.1542/peds.2006-1486	Wrong Setting
488	Joyce T, Racine A, Yunzal-Butler C. Reassessing the WIC effect: evidence from the Pregnancy Nutrition Surveillance System. *J Policy Anal Manage*. 2008;27(2):277-303. doi:10.1002/pam.20325	Wrong Setting

489	Kapinos KA, Bullinger LR, Gurley-Calvez T. Lactation Support Services and Breastfeeding Initiation: Evidence from the Affordable Care Act. Health Services Research. 2016;52:2175-2196. doi:10.1111/1475-6773.12598	Wrong Setting
490	Karanja N, Aickin M, Lutz T, et al. A community-based intervention to prevent obesity beginning at birth among American Indian children: study design and rationale for the PTOTS study. *J Prim Prev*. 2012;33(4):161-174. doi:10.1007/s10935-012-0278-8	Wrong Setting
	Karanja N, Lutz T, Ritenbaugh C, et al. The TOTS community intervention to prevent overweight in American Indian toddlers beginning at birth: a feasibility and efficacy study. *J Community Health*. 2010;35(6):667-75. doi:10.1007/s10900-010-9270-5	Wrong Setting
491	Karasz A, Bonuck K. Reducing pediatric caries and obesity risk in South Asian immigrants: randomized controlled trial of common health/risk factor approach. *BMC Public Health*. 2018;18(1):680. doi:10.1186/s12889-018-5317-9	Wrong Setting
	Nct. Obesity and Caries in Young South Asian Children: a Common Risk Factor Approach. https://clinicaltrialsgov/show/NCT03077425. 2017.	Wrong Setting
492	Kemp L, Grace R, Comino E, et al. The effectiveness of a sustained nurse home visiting intervention for Aboriginal infants compared with non-Aboriginal infants and with Aboriginal infants receiving usual child health care: a quasi-experimental trial - the Bulundidi Gudaga study. *BMC Health Serv Res*. 2018;18(599):(3 August 2018). doi:10.1186/s12913-018-3394-1	Wrong Setting
493	Kieffer EC, Welmerink DB, Sinco BR, et al. Dietary Outcomes in a Spanish-Language Randomized Controlled Diabetes Prevention Trial With Pregnant Latinas. *Am J Public Health*. 2014;104(3):526-533. doi:10.2105/ajph.2012.301122	Wrong Setting
494	Kronborg H, Vaeth M, Olsen J, Iversen L, Harder I. Effect of early postnatal breastfeeding support: a cluster-randomized community based trial. *Acta Paediatr*. 2007;96:1064-1070. doi:10.1111/j.1651-2227.2007.00341.x	Wrong Setting
495	Langellier BA, Chaparro MP, Wang MC, Koleilat M, Whaley SE. The new food package and breastfeeding outcomes among women, infants, and children participants in Los Angeles County. *Am J Public Health*. 2013;104:112-118. doi:10.2105/ajph.2013.301330	Wrong Setting
496	Lazariu-Bauer V, Stratton HH, Pruzek RM, Woelfel ML. A comparative analysis of effects of early versus late prenatal WIC participation on birth weight: NYS, 1995. *Matern Child Health J*. 2004;8:77-86. doi:10.1023/b:maci.0000025730.02966.62	Wrong Setting
497	Martinez-Brockman JL, Harari N, Perez-Escamilla R. Lactation advice through texting can help (LATCH): An analysis of intensity of engagement via two-way text messaging. *FASEB J*. 2017;31(1)	Wrong Setting
	Martinez-Brockman JL, Harari N, Segura-Pérez S, Goeschel L, Bozzi V, Pérez-Escamilla R. Impact of the Lactation Advice Through Texting Can Help (LATCH) Trial on Time to First Contact and Exclusive Breastfeeding among WIC Participants. *J Nutr Educ Behav*. 2018;50(1):33-42.e1. doi:10.1016/j.jneb.2017.09.001	Wrong Setting
	Martinez-Brockman JL, Harari N, Segura-Perez S, Goeschel L, Perez-Escamilla R. Impact of the lactation advice through texting can help (LATCH) randomized controlled trial. *FASEB J*. 2017;31(1)	Wrong Setting

498	McEachan RRC, Santorelli G, Bryant M, et al. The HAPPY (Healthy and Active Parenting Programmme for early Years) feasibility randomised control trial: acceptability and feasibility of an intervention to reduce infant obesity. *BMC Public Health*. 2016;16:211. doi:10.1186/s12889-016-2861-z	Wrong Setting
499	Metallinos-Katsaras ES, Brown L, Colchamiro R. Maternal WIC participation improves breastfeeding rates: A statewide analysis of WIC participants. *FASEB J*. 2013;27	Wrong Setting
	Metallinos-Katsaras E, Brown L, Colchamiro R. Maternal WIC participation improves breastfeeding rates: a statewide analysis of WIC participants. *Matern Child Health J*. 2015;19(1):136-43. doi:10.1007/s10995-014-1504-1	Wrong Setting
500	Navarro JI, Sigulem DM, Ferraro AA, Polanco JJ, Barros AJ, Barros AJD. The double task of preventing malnutrition and overweight: a quasi-experimental community-based trial. *BMC Public Health*. 2013;13(1):212-212. doi:10.1186/1471-2458-13-212	Wrong Setting
501	Nct. Promoting Healthy Weight Across the Pregnancy and Postpartum Period. https://clinicaltrialsgov/show/NCT04253977. 2020.	Wrong Setting
502	Nct. Healthy Eating for My Infant (HEMI). https://clinicaltrialsgov/show/NCT04977947. 2021.	Wrong Setting
503	Nct. Baby Nutrition and Physical Activity Self-Assessment for Child Care (NAP SACC) Intervention Study. https://clinicaltrialsgov/show/NCT01890681. 2013.	Wrong Setting
504	Nct. Partnering With WIC to Prevent Excessive Weight Gain in Pregnancy. https://clinicaltrialsgov/show/NCT03707834. 2018.	Wrong Setting
505	Olson CM, Baker IR, Demment MM, et al. The healthy start partnership: an approach to obesity prevention in young families. *Fam Community Health*. 2014;37(1):74-85. doi:10.1097/fch.0000000000000016	Wrong Setting
506	Pulvera R, Collin DF, Hamad R. The effect of the 2009 WIC revision on maternal and child health: A quasi-experimental study. *Paediatr Perinat Epidemiol*. 2022;36(6):851-860. doi:10.1111/ppe.12898	Wrong Setting
507	Ridberg RA, Levi R, Marpadga S, Akers M, Tancredi DJ, Seligman HK. Additional Fruit and Vegetable Vouchers for Pregnant WIC Clients: An Equity-Focused Strategy to Improve Food Security and Diet Quality. *Nutrients*. 2022;14:2328-2328. doi:10.3390/nu14112328	Wrong Setting
	Ridberg RA, Marpadga S, Akers M, Bell JF, Seligman HK. Fruit and Vegetable Vouchers in Pregnancy: Preliminary Impact on Diet & Food Security. *J Hunger Environ Nutr*. 2020;16:149-163. doi:10.1080/19320248.2020.1778593	Wrong Setting
508	Rotheram-Fuller E, Swendeman D, Becker K, et al. Replicating Evidence-Based Practices with Flexibility for Perinatal Home Visiting by Paraprofessionals. *Matern Child Health J*. 2017;21(12):2209-2218. doi:10.1007/s10995-017-2342-8	Wrong Setting

509	Sandy JM, Anisfeld E, Ramirez E. Effects of a prenatal intervention on breastfeeding initiation rates in a Latina immigrant sample. *J Hum Lact*. 2009;25:404-411. doi:10.1177/0890334409337308	Wrong Setting
510	Santana JM, Pereira M, Lisboa CS, Santos DB, Oliveira AM. Influence of conditional cash transfer program on prenatal care and nutrition during pregnancy: NISAMI cohort study. *Sao Paulo Med J*. 2022;140(4):595-603. doi:10.1590/1516-3180.2021.0449.R1.23112021	Wrong Setting
511	Silva Junior AEd, Macena MdL, Vasconcelos LGL, et al. Trend of the nutritional status of pregnant adolescent beneficiaries of the Brazilian Bolsa Familia conditional cash transfer program in the 2008-2018 period. *Cienc Saude Colet*. 2021;26(7):2613-2624. doi:10.1590/1413-81232021267.08172021	Wrong Setting
512	Skovgaard AM, Bakermans-Kranenburg M, Pontoppidan M, et al. The Infant Health Study - Promoting mental health and healthy weight through sensitive parenting to infants with cognitive, emotional, and regulatory vulnerabilities: protocol for a stepped-wedge cluster-randomized trial and a process evaluation within municipality settings. *BMC Public Health*. 2022;22(1):194-. doi:10.1186/s12889-022-12551-z	Wrong Setting
513	Sonchak L. The impact of WIC on breastfeeding initiation and gestational weight gain: Case study of South Carolina Medicaid mothers. *Child Youth Serv Rev*. 2017;79:115-125. doi:10.1016/j.childyouth.2017.05.024	Wrong Setting
514	Struck S, Enns J, Sanguins J, et al. An unconditional prenatal cash benefit is associated with improved birth and early childhood outcomes for Metis families in Manitoba, Canada. *Child Youth Serv Rev*. 2021;121:105853-undefined. doi:10.1016/j.childyouth.2020.105853	Wrong Setting
515	Topolyan I, Xu X. Differential effects of mother's and child's postnatal WIC participation on breastfeeding. *Appl Econ*. 2017;49(23):2216-2225. doi:10.1080/00036846.2016.1234702	Wrong Setting
516	Varea A, Malpeli A, Etchegoyen G, et al. Short-term evaluation of the impact of a food program on the micronutrient nutritional status of Argentinean children under the age of six. *Biol Trace Elem Res*. 2011;143(3):1337-48. doi:10.1007/s12011-011-8978-0	Wrong Setting
517	Vari P, Camburn J, Henly SJ. Professionally mediated peer support and early breastfeeding success. The *J Perinat Educ*. 2000;9:22-30. doi:10.1624/105812400x87473	Wrong Setting
518	Ventura AK, Silva Garcia K, Meza M, Rodriguez E, Martinez CE, Whaley SE. Promoting Responsive Bottle-Feeding Within WIC: Evaluation of a Policy, Systems, and Environmental Change Approach. *J Acad Nutr Diet*. 2022;122(1):99-109.e2. doi:10.1016/j.jand.2021.05.003	Wrong Setting
519	Wang G, Seligman H, Levi R, Hamad R. Impact of fruit and vegetable benefits on pregnancy outcomes among WIC participants: a natural experiment. *Transl Behav Med*. 2022;doi:10.1093/tbm/ibac063	Wrong Setting
520	Watt RG, Tull KI, Hardy R, et al. Effectiveness of a social support intervention on infant feeding practices: randomised controlled trial. *J Epidemiol Community Health*. 2009;63(2):156-62. doi:10.1136/jech.2008.077115	Wrong Setting
521	Whaley SE, Koleilat M, Whaley M, Gomez J, Meehan K, Saluja K. Impact of policy changes on infant feeding decisions among low-income women participating in the Special Supplemental Nutrition Program for Women, Infants, and Children. *Am J Public Health*. 2012;102:2269-2273. doi:10.2105/ajph.2012.300770	Wrong Setting

522	Whaley SE, Samar Mcgregor LJ, Gomez J, Harrison G, Jenks E. A WIC-based intervention to prevent early childhood overweight. *J Nutr Educ Behav*. 2010;42(3 suppl. 1):S47-S51. doi:10.1016/j.jneb.2010.02.010	Wrong Setting
523	Wilde P, Wolf A, Fernandes M, Collins AM. Food-package assignments and breastfeeding initiation before and after a change in the Special Supplemental Nutrition Program for Women, Infants, and Children. *Am J Clin Nutr*. 2012;96:560-566. doi:10.3945/ajcn.112.037622	Wrong Setting
524	Williams CB, LaCoursiere DY, Talavera GA, Gahagan S. A Feasibility Study to Promote Optimal Weight in First Time Pregnant Mothers and Their Babies: Lessons Learned in a US-Mexico Border Community. *Matern Child Health J*. 2019;23(5):578-584. doi:10.1007/s10995-018-2685-9	Wrong Setting
525	Yun S, Liu Q, Mertzlufft K, et al. Evaluation of the Missouri WIC (Special Supplemental Nutrition Program for Women, Infants, and Children) breast-feeding peer counselling programme. *Public Health Nutr*. 2009;13:229-237. doi:10.1017/s1368980009990668	Wrong Setting
526	Actrn. The Early Prevention of Obesity in Children (EPOCH) Collaboration. http://wwwwhoint/trialsearch/Trial2aspx?TrialID=ACTRN12610000789066. 2010;	Wrong Study Design
527	Adeli K. Closing the gaps in pediatric reference intervals: An Update on the CALIPER Project. *Clinical Biochemistry*. 2014;47(9):737-739. doi:10.1016/j.clinbiochem.2014.05.037	Wrong Study Design
528	Alemán-Díaz AY, Backhaus S, Siebers LL, et al. Child and adolescent health in Europe: monitoring implementation of policies and provision of services. *Lancet Child Adolesc Health*. 2018;2(12):891-904. doi:10.1016/s2352-4642(18)30286-4	Wrong Study Design
529	Al-Jawaldeh A, Abul-Fadl A. Assessment of the Baby Friendly Hospital Initiative Implementation in the Eastern Mediterranean Region. *Children (Basel)*. 2018;5(3)doi:10.3390/children5030041	Wrong Study Design
530	Ammerman AS, Ward DS, Benjamin SE, et al. An intervention to promote healthy weight: Nutrition and Physical Activity Self-Assessment for Child Care (NAP SACC) theory and design. *Prev Chronic Dis*. 2007;4(3):A67.	Wrong Study Design
531	Black MM, Cutts DB, Frank DA, et al. Special Supplemental Nutrition Program for Women, Infants, and Children participation and infants' growth and health: a multisite surveillance study. *Pediatrics*. 2004;114(1):169-76. doi:10.1542/peds.114.1.169	Wrong Study Design
532	Bottorff JL, Huisken A, Hopkins M, Nesmith C. A RE-AIM evaluation of Healthy Together: a family-centred program to support children's healthy weights. *BMC Public Health*. 2020;20(1):1754. doi:10.1186/s12889-020-09737-8	Wrong Study Design
533	Bruner LD, Glover SH. The Association of Hospital Practices to Breastfeeding Behaviors in South Carolina: Analysis of 2013-2015 Pregnancy Risk Assessment Monitoring System (PRAMS) Data. 2018;(10980620):144.	Wrong Study Design
534	Bruney TL, Campbell D, Iyare A. Achieving Baby-Friendly Designation at a Large Metropolitan Center. *Am J Perinatol*. 2019;37:430-435. doi:10.1055/s-0039-1681056	Wrong Study Design
535	Buckler AG. The Military Health System and TRICARE: breastfeeding promotion. *Breastfeed Med*. 2011;6:295-7. doi:10.1089/bfm.2011.0065	Wrong Study Design

536	Colchamiro R, Edwards RA, Nordstrom C, et al. Mobilizing Community Resources to Enhance Postdischarge Support for Breastfeeding in Massachusetts (USA): Results of a Catalyst Grant Approach. *J Hum Lact*. 2015;31(4):631-40. doi:10.1177/0890334415597680	Wrong Study Design
537	Dave DM, Kaestner R, Wehby GL. Does public insurance coverage for pregnant women affect prenatal health behaviors? *J Popul Econ*. 2019;32(2):419-453. doi:10.1007/s00148-018-0714-z	Wrong Study Design
538	Design of lifestyle intervention trials to prevent excessive gestational weight gain in women with overweight or obesity. *Obes*. 2016;24(2):305-313.	Wrong Study Design
539	Dharod JM, Drewette-Card R, Crawford D. Development of the Oxford Hills Healthy Moms Project Using a Social Marketing Process: A Community-Based Physical Activity and Nutrition Intervention for Low-Socioeconomic-Status Mothers in a Rural Area in Maine. *Health Promot Pract*. 2011;12(2):312-321. doi:10.1177/1524839909355521	Wrong Study Design
540	Dulon M, Kersting M, Bender R. Breastfeeding promotion in non-UNICEF-certified hospitals and long-term breastfeeding success in Germany. *Acta paediatr (Oslo, Norway : 1992)*. 2007;92:653-658. doi:10.1111/j.1651-2227.2003.tb00594.x	Wrong Study Design
541	Figlio DN, Hamersma S, Roth J. Does prenatal WIC participation improve birth outcomes? New evidence from Florida. *J Public Econ*. 2009;93:235-245. doi:10.1016/j.jpubeco.2008.08.003	Wrong Study Design
542	Forrester-Knauss C, Merten S, Weiss C, Ackermann-Liebrich U, Stutz EZ. The baby-friendly hospital initiative in Switzerland: trends over a 9-year period. *J Hum Lact*. 2013;29:510-516. doi:10.1177/0890334413483923	Wrong Study Design
543	Francescon J, Haile ZT, Kling D, Chertok IRA. Association Between WIC Enrollment and Exclusive Breastfeeding at 3 Months Postpartum Among Low-Income Mothers. *J Am Osteopath Assoc*. 2016;116:770-779. doi:10.7556/jaoa.2016.152	Wrong Study Design
544	Gleason S, Wilkin M, Sallack L, Whaley SE, Martinez CE, Paolicelli C. Breastfeeding Duration Is Associated With WIC Site-Level Breastfeeding Support Practices. *J Nutr Educ Behav*. 2020;52:680-687. doi:10.1016/j.jneb.2020.01.014	Wrong Study Design
545	Gottesman MM. Turn up the heat: the Healthy Eating and Activity Together initiative. *Adv Nurse Pract*. 2006;14(3):47-8, 50, 52.	Wrong Study Design
546	Huang X, Yang B, Liu Q, Zhang R, Tang S, Story M. Improving maternal and child nutrition in China: an analysis of nutrition policies and programs initiated during the 2000-2015 Millennium Development Goals era and implications for achieving the Sustainable Development Goals. *J Health Popul Nutr*. 2020;39(1):12. doi:10.1186/s41043-020-00221-y	Wrong Study Design
547	Israel G. Sisters in Birth: Improving Birth Outcomes in Mississippi. *J Perinat Educ*. 2020;29(4):168-180. doi:10.1891/j-pe-d-20-00044	Wrong Study Design
548	Jung S, Nobari TZ, Whaley SE. Breastfeeding Outcomes Among WIC-Participating Infants and Their Relationships to Baby-Friendly Hospital Practices. *Breastfeed Med*. 2019;14:424-431. doi:10.1089/bfm.2019.0004	Wrong Study Design
549	Kavle JA, Ahoya B, Kiige L, et al. Baby-Friendly Community Initiative-From national guidelines to implementation: A multisectoral platform for improving infant and young child feeding practices and integrated health services. *Matern Child Nutr*. 2019;15:e12747-undefined. doi:10.1111/mcn.12747	Wrong Study Design

550	Kawsar J, Leschewski A. Food Assistance Programs in the U.S. and Their Impact on Childhood Obesity. 2019;(22618225):64.	Wrong Study Design
551	Kristjansson E, Francis D, Liberato SC, et al. Early childhood feeding interventions to improve the physical and psychosocial health of disadvantaged children aged 3 months to 5 years. 2012.	Wrong Study Design
552	Lakshmanan A, Song AY, Flores-Fenlon N, et al. Association of WIC Participation and Growth and Developmental Outcomes in High-Risk Infants. *Clin Pediatr*. 2019;59:53-61. doi:10.1177/0009922819884583	Wrong Study Design
553	Li Ming W. A bottle-weaning counselling intervention for parents of 12-month-old infants reduces bottle use at age 24 months but has no effect on child weight. *Evid Based Nurs*. 2015;18(1):14-14. doi:10.1136/eb-2014-101825	Wrong Study Design
554	Love P, Laws R, Hesketh KD, Campbell KJ. Lessons on early childhood obesity prevention interventions from the Victorian Infant Program. *Public Health Res Pract*. 2019;29(1)doi:10.17061/phrp2911904	Wrong Study Design
555	Ma X, Liu J, Smith MG. WIC Participation and Breastfeeding in South Carolina: Updates from PRAMS 2009–2010. *Matern Child Health J*. 2013;18:1271-1279. doi:10.1007/s10995-013-1362-2	Wrong Study Design
556	Marshall S, Xu H, Taki S, et al. Engagement, satisfaction, retention and behavioural outcomes of linguistically diverse mothers and infants participating in an Australian early obesity prevention trial. *Health Promot J Austr*. 2021;33(2):350-360. doi:10.1002/hpja.521	Wrong Study Design
557	Mayer K, Cheadle A. Describing Hunger-related Outcomes in a Community Kitchen located in the Pacific Northwest, United States. 2014;(1573704):59.	Wrong Study Design
558	McGinnis S, Lee E, Kirkland K, Miranda-Julian C, Greene R. Let's Talk About Breastfeeding: The Importance of Delivering a Message in a Home Visiting Program. *Am J Health Promot*. 2017;32:989-996. doi:10.1177/0890117117723802	Wrong Study Design
559	Mikiel-Kostyra K, Mazur J. Hospital policies and their influence on newborn body weight. *Acta Paediatr*. 1999;88(1):72-75. doi:10.1080/080352599750029637	Wrong Study Design
560	Nagata JM, Djafari JT, Chamberlain LJ. The option of replacing the special supplemental nutrition program for women, infants, and children fruit juice supplements with fresh fruits and vegetables. *JAMA Pediatr*. 2016;170(9):823-824. doi:10.1001/jamapediatrics.2016.1178	Wrong Study Design
561	Pérez-Escamilla R, Lutter CK, Rabadan-Diehl C, et al. Prevention of childhood obesity and food policies in Latin America: from research to practice. *Obes Rev*. 2017;18:28-38.	Wrong Study Design
562	Phelan S, Clifton RG, Haire-Joshu D, et al. One-year postpartum anthropometric outcomes in mothers and children in the LIFE-Moms lifestyle intervention clinical trials. *Int J Obes (Lond)*. 2020;44(1):57-68. doi:10.1038/s41366-019-0410-4	Wrong Study Design
563	Reat AM, Crixell S, Friedman B, Von Bank J. Comparison of Food Intake Among Infants and Toddlers Participating in a South Central Texas WIC Program Reveals Some Improvements After WIC Package Changes. *Matern Child Health J*. 2015;19:1834-1841. doi:10.1007/s10995-015-1697-y	Wrong Study Design
564	Redwood D, Mitchell-Box K, Peterson E, Provost E. Improving the Health of Alaska Native People Through Use of a Policy Change Model and Capacity Building. *Prev Chronic Dis*. 2019;16:E64. doi:10.5888/pcd16.190077	Wrong Study Design

565	Rushton FE, Byrne WW, Darden PM, McLeigh J. Enhancing child safety and well-being through pediatric group well-child care and home visitation: The Well Baby Plus Program. *Child Abuse Negl*. 2015;41:182-9. doi:10.1016/j.chiabu.2015.01.008	Wrong Study Design
566	Schoenfelder SL, Wych S, Willows CA, Harrington J, Christoffel KK, Becker AB. Engaging Chicago hospitals in the baby-friendly hospital initiative. *Matern Child Health J*. 2013;17(9):1712-7. doi:10.1007/s10995-012-1144-2	Wrong Study Design
567	Stopka TJ, Segura-Pérez S, Chapman DJ, Damio G, Pérez-Escamilla R. An innovative community-based approach to encourage breastfeeding among Hispanic/Latino women. *J Am Diet Assoc*. 2002;102:766-767. doi:10.1016/s0002-8223(02)90170-7	Wrong Study Design
568	Strang L, Broeks M. Maternity Leave Policies: Trade-Offs Between Labour Market Demands and Health Benefits for Children. *Rand Health Q*. 2017;6(4):9.	Wrong Study Design
569	Struthers A, Metge CJ, Charette C, et al. Understanding the Particularities of an Unconditional Prenatal Cash Benefit for Low-Income Women: A Case Study Approach. *Inquiry*. 2019;56:46958019870967-undefined. doi:10.1177/0046958019870967	Wrong Study Design
570	Truiett-Theodorson R, Tuck S, Bowie JV, Summers AC, Kelber-Kaye J. Building effective partnerships to improve birth outcomes by reducing obesity: The B'more Fit for healthy babies coalition of Baltimore. *Eval Program Plann*. 2015;51:53-8. doi:10.1016/j.evalprogplan.2014.12.007	Wrong Study Design
571	Vanderlinden K, Buffel V, Van de Putte B, Van de Velde S. Motherhood in Europe: An Examination of Parental Leave Regulations and Breastfeeding Policy Influences on Breastfeeding Initiation and Duration. *Social Sciences*. 2020;9:222-undefined. doi:10.3390/socsci9120222	Wrong Study Design
572	Venancio SI, Saldiva S, Escuder MML, Giugliani ERJ. The Baby-Friendly Hospital Initiative shows positive effects on breastfeeding indicators in Brazil. *J Epidemiol Community Health*. 2011;66:914-918. doi:10.1136/jech-2011-200332	Wrong Study Design
573	Whaley S, Koleilat M, Whaley M. Breastfeeding & obesity: WIC policy changes increase breastfeeding rates and reduce obesity at age four. *FASEB J*. 2012;26.	Wrong Study Design
574	Whaley SE, Ritchie LD, Spector P, Gomez J. Revised WIC Food Package Improves Diets of WIC Families. *J Nutr Educ Behav*. 2012;44:204-209. doi:10.1016/j.jneb.2011.09.011	Wrong Study Design
575	Zhang Q, Chen C, Xue H, Park K, Wang Y. Revisiting the Relationship between WIC Participation and Breastfeeding among Low-Income Children in the U.S. after the 2009 WIC Food Package Revision. *Food Policy*. 2021;101:102089-undefined. doi:10.1016/j.foodpol.2021.102089	Wrong Study Design
576	Ziol-Guest KM, Hernandez DC. First- and second-trimester WIC participation is associated with lower rates of breastfeeding and early introduction of cow's milk during infancy. *J Am Diet Assoc.* 2010;110:702-709. doi:10.1016/j.jada.2010.02.013	Wrong Study Design

Supplementary Data

Supplemental Table 4. Summary of RE-AIM dimensions of included studies by setting (n=126)

**Study Identifiers**	**Study Elements**	**Reach**	**Effectiveness**	**Adoption**	**Implementation**	**Maintenance**
Author, year<br><br>*Intervention Name*<br><br>Location	Design:<br><br>Comparator:<br><br>Theory:	Sample:<br><br>Priority groups:<br><br>Analytic sample:<br><br>Attrition:	Outcomes:<br><br>Subgroup analysis:<br><br>Reported unintended consequences:	Setting:<br><br>Serve priority groups:<br><br>Analytic sample:<br><br>Interventionist:	Length of intervention:<br><br>Policy:<br><br>System:<br><br>Environmental:<br><br>Fidelity:<br><br>Cost:	Intervention sustained beyond study:<br><br>Reported sustainability of impact:
**Clinical + Community (n=19)**						
Beck, 2014(123)<br><br>*Pediatric primary care-community partnership*<br><br>USA	Design: Cohort<br><br>Comparator: No intervention<br><br>Theory: Not reported	Sample: Children with birthdays between June 1, 2010, and May 31, 2012<br><br>Priority groups: Low income, race and ethnicity<br><br>Analytic sample: 5,071 infants<br><br>Attrition: 0%	Outcomes:<br>Child weight: θ<br><br>Subgroup analysis: No<br><br>Reported unintended consequences: No	Setting: Partnership between Pediatric Primary Care Center with the Cincinnati Children's Hospital Medical Center and the Freestore Foodbank<br><br>Serve priority groups: Yes<br><br>Analytic sample: 1 primary care pediatric practice<br><br>Interventionist: Physicians	Length of intervention: 12 months<br><br>Policy:<br><br>System: Addition of food insecurity and social risk screening procedures to well infant care visits; co-developed process to link families with supplementary infant formula, educational materials, clinic and community resources<br><br>Environmental: Provision of formula<br><br>Fidelity: No<br><br>Cost: No	Intervention sustained beyond study: Yes<br><br>Reported sustainability of impact: No
Budge, 2023(141)<br><br>*Group well-child care with WIC*<br><br>USA	Design: Non-randomized control trial<br><br>Comparator: Historical control<br><br>Theory: Not reported	Sample: Infants and mothers seen for well-child care visits during the study period<br><br>Priority groups: Low income, race and ethnicity<br><br>Analytic sample: 6,413 infants<br><br>Attrition: 2%	Outcomes:<br>Child weight: θ<br><br>Subgroup analysis: No<br><br>Reported unintended consequences: No	Setting: Yale Pediatric Primary Care Center<br><br>Serve priority groups: Yes<br><br>Analytic sample: 1 primary care pediatric practice<br><br>Interventionist: Clinical team - nurse, pediatric resident, attending pediatrician, social	Length of intervention: 12 months<br><br>Policy:<br><br>System: Change from individual to group well-child care<br><br>Environmental: Peer groups<br><br>Fidelity: Yes	Intervention sustained beyond study: Unsure<br><br>Reported sustainability of impact: No

Supplementary Data

Study Identifiers	Study Elements	Reach	Effectiveness	Adoption	Implementation	Maintenance
				worker, child-life specialist	Cost: No	
Feldman-Winter, 2010(176)<br><br>*Residency curriculum for breastfeeding care*<br><br>USA	Design: Repeated cross-sectional<br><br>Comparator: Standard of care<br><br>Theory: Not reported	Sample: Charts dated after completion of the intervention<br><br>Priority groups: Unsure<br><br>Analytic sample: 850 medical records<br><br>Attrition: 30%	Outcomes:<br>Breastfeeding: +*<br>Formula feeding: +*<br><br>Subgroup analysis: No<br><br>Reported unintended consequences: No	Setting: Pediatrics, family medicine, and obstetrics and gynecology residency programs<br><br>Serve priority groups: Yes<br><br>Analytic sample: 12 sites (resident programs)<br><br>Interventionist: Faculty physicians	Length of intervention: Not reported<br><br>Policy:<br><br>System: Training residents in breastfeeding curriculum<br><br>Environmental:<br><br>Fidelity: No<br><br>Cost: No	Intervention sustained beyond study: Unsure<br><br>Reported sustainability of impact: No
Grant, 2018(184)<br><br>*CenteringPregnancy®*<br><br>USA	Design: Retrospective cohort study<br><br>Comparator: No control<br><br>Theory: Not reported	Sample: Low-income women in the Southwest Public Health District in Georgia<br><br>Priority groups: Low income, race and ethnicity<br><br>Analytic sample: 338 patients<br><br>Attrition: n/a	Outcomes:<br>Breastfeeding: Not reported<br>Child weight: +<br><br>Subgroup analysis: No<br><br>Reported unintended consequences: No	Setting: Doherty County Health Department and Ellington Farm Worker Clinic<br><br>Serve priority groups: Yes<br><br>Analytic sample: 2 primary prenatal care sites<br><br>Interventionist: Health department nurse practitioners, nurses, and staff	Length of intervention: 6 months<br><br>Policy:<br><br>System: Change from individual to group model of prenatal care and training for clinical and administrative staff; transportation assistance; telehealth services; social service linkages<br><br>Environmental: Peer support<br><br>Fidelity: No<br><br>Cost: No	Intervention sustained beyond study: Yes<br><br>Reported sustainability of impact: No
Holmes, 2012 (185)<br>Holmes, 2009(1)(*Suppl. Ref*)<br><br>*Physician breastfeeding education*<br><br>USA	Design: Non-randomized control trial<br><br>Comparator: No intervention<br><br>Theory: Not reported	Sample: All infants with at least one well-child visit<br><br>Priority groups: Rural, medically indigent<br><br>Analytic sample: 176 infants<br><br>Attrition: 2%	Outcomes:<br>Breastfeeding: +*<br><br>Subgroup analysis: Faculty-resident, low-high dose of training<br><br>Reported unintended consequences: No	Setting: Family medicine residency programs<br><br>Serve priority groups: Yes<br><br>Analytic sample: 2 residency programs<br><br>Interventionist: Medical faculty and residents	Length of intervention: 9 months<br><br>Policy:<br><br>System: Modifications of standard training opportunities for medical residency program<br><br>Environmental:<br><br>Fidelity: Yes	Intervention sustained beyond study: Unsure<br><br>Reported sustainability of impact: No

Supplementary Data

Study Identifiers	Study Elements	Reach	Effectiveness	Adoption	Implementation	Maintenance
					Cost: No	
Kim (NCT) , 2019(2) (*Suppl. Ref*)<br><br>*Integrating maternal nutrition intervention services into antenatal care services*<br><br>Ethiopia	Design: Randomized control trial<br><br>Comparator: Standard of care<br><br>Theory: Not reported	Sample: Women registered at the government health center as currently pregnant and or have a child under 6 months of age at the time of the survey and have attended at least 1 antenatal care visit at the government health center/post<br><br>Priority groups: Low income, ethnicity, rural<br><br>Analytic sample: Not reported<br><br>Attrition: n/a	Outcomes:<br>Ongoing study (breastfeeding, maternal diet)<br><br>Subgroup analysis: No<br><br>Reported unintended consequences: Yes	Setting: Districts in Southern Nations Nationalities and People's Region and Somali region<br><br>Serve priority groups: Yes<br><br>Analytic sample: 30 health center catchment areas<br><br>Interventionist: Nurse-midwife, health worker/extension worker	Length of intervention: 6 months<br><br>Policy:<br><br>System: Integrated package of maternal nutrition interventions into existing antenatal care services<br>Trainings for key actors and supportive supervision of maternal nutrition activities<br><br>Environmental: Health Extension Workers and community volunteers (peers) to provide services, including supplements<br>Community meetings and mother support groups<br>Encouragement of husband participation<br><br>Fidelity: Yes<br><br>Cost: No	Intervention sustained beyond study: Unsure<br><br>Reported sustainability of impact: No
Kistin, 1994(137)<br><br>*Peer counseling for breastfeeding*<br><br>USA	Design: Non-randomized control trial<br><br>Comparator: Standard of care<br><br>Theory: Empowerment Education	Sample: English or Spanish speaking women who were primigravida or reported previous breastfeeding difficult, planned to breastfeed, and had requested a peer counselor<br><br>Priority groups: Low income, race and ethnicity<br><br>Analytic sample: 102 women<br><br>Attrition: 0%	Outcomes:<br>Breastfeeding: +*<br><br>Subgroup analysis: No<br><br>Reported unintended consequences: Yes	Setting: Partnership between Chicago Breastfeeding Task Force and Cook County Hospital<br><br>Serve priority groups: Yes<br><br>Analytic sample: 1 community-hospital partnership<br><br>Interventionist: Peer counselors	Length of intervention: 12 weeks<br><br>Policy:<br><br>System: Establishment of breastfeeding peer counselor program and referrals to community resources<br><br>Environmental: Peer counselors<br><br>Fidelity: No<br><br>Cost: No	Intervention sustained beyond study: Unsure<br><br>Reported sustainability of impact: No

Supplementary Data

Study Identifiers	Study Elements	Reach	Effectiveness	Adoption	Implementation	Maintenance
Kramer, 2001(149)<br><br>*Promotion of Breastfeeding Intervention Trial (PROBIT)*<br><br>Belarus	Design: Cluster randomized control trial<br><br>Comparator: Standard of care<br><br>Theory: Not reported	Sample: Mothers of singletons ≥ 37 weeks' gestation who upon admission to postpartum ward expressed intention to breastfeed and did not have contraindications for breastfeeding<br><br>Priority groups: Unsure<br><br>Analytic sample: 16,442 mother infant pairs<br><br>Attrition: 3%	Outcomes:<br>Breastfeeding: +*<br><br>Subgroup analysis: No<br><br>Reported unintended consequences: No	Setting: Maternity hospitals and polyclinics in the Republic of Belarus<br><br>Serve priority groups: Unsure<br><br>Analytic sample: 31 maternal hospitals and associated polyclinics<br><br>Interventionist: Chief obstetrician and chief pediatrician, midwives, nurses, physicians, pediatricians	Length of intervention:<br>Policy: n/a<br><br>Policy: Written breastfeeding policy<br><br>System: Lactation management training course for health professionals<br><br>Environmental:<br><br>Fidelity: No<br><br>Cost: No	Intervention sustained beyond study: Unsure<br><br>Reported sustainability of impact: No
Maingi, 2018(96)<br><br>*Baby Friendly Community Initiative*<br><br>Kenya	Design: Randomized control trial<br><br>Comparator: Standard of care<br><br>Theory: Not reported	Sample: Mothers with children 6 - 23 months residing in community units where initially recruited<br><br>Priority groups: Rural<br><br>Analytic sample: 270 mother-child pairs<br><br>Attrition: 0%	Outcomes:<br>Child diet: +*<br>Complementary feeding: +*<br><br>Subgroup analysis: No<br><br>Reported unintended consequences: No	Setting: Community units in Koibatek Sub-County, in Baringo County<br><br>Serve priority groups: Yes<br><br>Analytic sample: 6 community units<br><br>Interventionist: Healthcare providers and community health volunteers	Length of intervention:<br>Policy: n/a<br><br>Policy: Written Maternal and Infant and Young Child Nutrition policy summary statement<br><br>System: Baby-Friendly Community Initiative expands Baby-Friendly Hospital Initiative to support breastfeeding after leaving health facility<br><br>Environmental: Provide environments supportive of breastfeeding families Support groups<br><br>Fidelity: No<br><br>Cost: No	Intervention sustained beyond study: Unsure<br><br>Reported sustainability of impact: No
Metwally, 2022(140)<br><br>*Communication for behavioral development approach*<br><br>Egypt	Design: Non-randomized control trial<br><br>Comparator: No intervention<br><br>Theory: Not reported	Sample: Mothers of children ≤ 24 months<br><br>Priority groups: Unsure<br><br>Analytic sample: 400 mother-infant pairs	Outcomes:<br>Child diet: +*<br>Complementary feeding: +*<br>Child weight: +*<br><br>Subgroup analysis: By sex	Setting: Villages in the El Mahala district, in El Gharbyia governorate<br><br>Serve priority groups: Unsure	Length of intervention:<br>3 years<br><br>Policy:<br><br>System:	Intervention sustained beyond study: Unsure<br><br>Reported sustainability of impact: No

Supplementary Data

Study Identifiers	Study Elements	Reach	Effectiveness	Adoption	Implementation	Maintenance
		Attrition: 32%	Reported unintended consequences: No	Analytic sample: 2 villages<br><br>Interventionist: Social workers	Environmental: A model nutrition kitchen<br>Peer support and communication campaigns to build awareness and change norms<br><br>Fidelity: No<br><br>Cost: No	
Nommsen-Rivers, 2009(98)<br><br>*Doula care program*<br><br>USA	Design: Cohort<br><br>Comparator: Standard of care<br><br>Theory: Not reported	Sample: Primiparous low-income patient who speaks English or Spanish without contraindications for breastfeeding<br><br>Priority groups: Low income, race and ethnicity, low education, patients with obesity<br><br>Analytic sample: 141 women<br><br>Attrition: 17%	Outcomes:<br>Breastfeeding: +<br><br>Subgroup analysis: Maternal age, Hispanic ethnicity, maternal education, obesity status feeding plans<br><br>Reported unintended consequences: No	Setting: Regional hospital in Sacramento, California<br><br>Serve priority groups: Yes<br><br>Analytic sample: 1 hospital<br><br>Interventionist: Doulas	Length of intervention: 2 weeks<br><br>Policy:<br><br>System: Provision of in-hospital and home visit doula care<br><br>Environmental: Emotional, informational, and physical support from doulas<br><br>Fidelity: Yes<br><br>Cost: No	Intervention sustained beyond study: Unsure<br><br>Reported sustainability of impact: No
Paul, 2012(139)<br><br>*Nurses for Infants through Teaching and Assessment after the Nursery (NITTANY)*<br><br>USA	Design: Randomized control<br><br>Comparator: Standard of care<br><br>Theory: Not reported	Sample: Singletons and twins born after 34 weeks' gestation to English speaking mothers attempting to breastfeed<br><br>Priority groups: No<br><br>Analytic sample: 1,154 mothers<br><br>Attrition: 0%	Outcomes:<br>Breastfeeding: +*<br><br>Subgroup analysis: Parity, insurance status, timing of newborn discharge<br><br>Reported unintended consequences: No	Setting: Academic hospital coordinating with community-based, private home health agency<br><br>Serve priority groups: No<br><br>Analytic sample: 1 academic hospital<br><br>Interventionist: Maternal Child Health nurses	Length of intervention: 1 day<br><br>Policy:<br><br>System: Home nursing visits within 48 hours of discharge; scheduling healthy newborn visit for approximately one week after home visit before hospital discharge<br><br>Environmental:<br><br>Fidelity: No<br><br>Cost: No	Intervention sustained beyond study: No<br><br>Reported sustainability of impact: Yes

Supplementary Data

Study Identifiers	Study Elements	Reach	Effectiveness	Adoption	Implementation	Maintenance
Pugh, 2002(138)<br><br>*Enhanced lactation support*<br><br>USA	Design: Randomized control trial<br><br>Comparator: Standard of care<br><br>Theory: Not reported	Sample: Low-income postpartum women<br><br>Priority groups: Low income, race and ethnicity<br><br>Analytic sample: 41 women<br><br>Attrition: 0%	Outcomes:<br>Breastfeeding: +<br>Formula feeding: +*<br><br>Subgroup analysis: No<br><br>Reported unintended consequences: No	Setting: Large academic medical center in the mid-Atlantic region<br><br>Serve priority groups: Yes<br><br>Analytic sample: 1 academic medical center<br><br>Interventionist: Community health nurse/peer counselor team	Length of intervention: 6 months<br><br>Policy:<br><br>System: Supplementary visits from community health nurse/peer counselor team in hospital and at home<br><br>Environmental: Peer counselors<br><br>Fidelity: No<br><br>Cost: Yes	Intervention sustained beyond study: No<br><br>Reported sustainability of impact: No
Redsell, 2017(102)<br><br>*Digital technology to facilitate Proactive Assessment of Obesity Risk during Infancy (ProAsk)*<br><br>United Kingdom	Design: Single group, pre-post design, feasibility study<br><br>Comparator: No control<br><br>Theory: Not reported	Sample: Parents of infants aged 6 - 8 weeks who understood English and did not screen for postnatal depression<br><br>Priority groups: Low income<br><br>Analytic sample: 53 parent-infant dyads<br><br>Attrition: 20%	Outcomes: Not reported (feeding practices, child diet, child weight)<br><br>Subgroup analysis: No<br><br>Reported unintended consequences: No	Setting: Urban and rural localities<br><br>Serve priority groups: Yes<br><br>Analytic sample: 3 sites in 2 localities<br><br>Interventionist: Health visitors	Length of intervention: 3 months<br><br>Policy:<br><br>System: Incorporation of obesity screening and therapeutic wheel messaging to health visitor visits<br><br>Environmental:<br><br>Fidelity: Yes<br><br>Cost: No	Intervention sustained beyond study: No<br><br>Reported sustainability of impact: Yes
Savage, 2022(108)<br>Savage, 2018(3) (*Suppl. Ref*)<br>Savage (NCT), 2018(4) (*Suppl. Ref*)<br><br>*Patient-centered, coordinated care approach delivered by community and pediatric primary care providers*<br><br>USA	Design: Randomized control trial<br><br>Comparator: Standard of care<br><br>Theory: Not reported	Sample: Mother-singleton infant dyads who speak English and are eligible for or enrolled in the WIC program<br><br>Priority groups: Low income, race and ethnicity<br><br>Analytic sample: 288 mother-infant dyads<br><br>Attrition: 17%	Outcomes:<br>Feeding practices: +*<br>Child weight: θ<br><br>Subgroup analysis: Multiparous, partnership status<br><br>Reported unintended consequences: No	Setting: Pediatric clinics in Luzerne County within Geisinger (health system) of northeastern Pennsylvania<br><br>Serve priority groups: Yes<br><br>Analytic sample: Not reported<br><br>Interventionist: Pediatric primary care providers and WIC nutritionists	Length of intervention: 6 months<br><br>Policy:<br><br>System: Incorporation of curriculum into well-child visits and regularly scheduled WIC visits, early healthy lifestyles assessment tool, and data integration for the coordination of care between clinical and WIC settings	Intervention sustained beyond study: Unsure<br><br>Reported sustainability of impact: No

Supplementary Data

Study Identifiers	Study Elements	Reach	Effectiveness	Adoption	Implementation	Maintenance
					Environmental:<br><br>Fidelity: No<br><br>Cost: No	
Sharma, 2018(85)<br>Rinehart, 2019(5)<br>(*Suppl. Ref*)<br><br>*HEAL*<br><br>USA	Design: Single group, pre-post design, pilot study<br><br>Comparator: No control<br><br>Theory: Social Cognitive Theory, Theory of Planned Behavior	Sample: Women pregnant <28 weeks and eligible for or participant of Medicaid<br><br>Priority groups: Low income, race and ethnicity, overweight or obesity<br><br>Analytic sample: 210 pregnant women<br>328 mother-infant dyads<br><br>Attrition: 36%	Outcomes:<br>Food security: +<br>Maternal diet: +*<br>Breastfeeding: +<br><br>Subgroup analysis: Income<br><br>Reported unintended consequences: No	Setting: Collaboration between University of Texas Physicians, the clinical practice arm of the UT Medical School at Houston, University of Texas School of Public Health, and community-based organizations<br><br>Serve priority groups: Yes<br><br>Analytic sample: 1 clinic-community partnership<br><br>Interventionist: Registered dietitians and certified community health workers	Length of intervention: 2 months<br><br>Policy:<br><br>System: Integration of programming into healthcare system and offered as standard of care; electronic medical record system changes for recruitment, tracking, and communication; clinic-community linkages and systems-level supports for sustainability of program components<br><br>Environmental: Provision of fresh, donated produce Community Health Workers provide social support and navigation for assistance programs<br><br>Fidelity: Yes<br><br>Cost: No	Intervention sustained beyond study: Yes<br><br>Reported sustainability of impact: No
Simpson, 2021(86)<br>John, 2014(6) (*Suppl. Ref*)<br>Jewell, 2014(7) (*Suppl. Ref*)<br>Gallagher, 2018(8)<br>(*Suppl. Ref*)<br><br>*Healthy eating and lifestyle in pregnancy (HELP)*<br><br>United Kingdom	Design: Cluster randomized control trial<br><br>Comparator: Standard of care<br><br>Theory: Control Theory, Social Cognitive Theory, Transactional Analysis, Motivational Interviewing, Compassionate Mind Theory	Sample: Pregnant women with BMI ≥ 30 kg/m^2 between 12 - 20 weeks' gestation<br><br>Priority groups: Obesity<br><br>Analytic sample: 464 postpartum women<br><br>Attrition: 22%	Outcomes:<br>Breastfeeding: Not reported<br>Child weight: θ<br>Maternal diet: +*<br>Maternal weight: θ<br><br>Subgroup analysis: Parity, social class, ethnicity and smoking status<br><br>Reported unintended consequences: Yes	Setting: Maternity units and community sites<br><br>Serve priority groups: Yes<br><br>Analytic sample: 20 maternity units<br><br>Interventionist: Midwife and consultants from Slimming World, a commercial weight	Length of intervention: 8 months<br><br>Policy:<br><br>System: Collaboration between National Health Service antenatal clinics and Slimming World to offer weight management groups<br><br>Environmental: Social support	Intervention sustained beyond study: Unsure<br><br>Reported sustainability of impact: Yes

Supplementary Data

Study Identifiers	Study Elements	Reach	Effectiveness	Adoption	Implementation	Maintenance
				management organisation	Voucher for a free Slimming World session at a 'regular' community group<br><br>Fidelity: Yes<br><br>Cost: Yes	
Su, 2007(106)<br><br>*Antenatal education and postnatal support strategies*<br><br>Singapore	Design: Randomized control trial<br><br>Comparator: Standard of care<br><br>Theory: Not reported	Sample: Mothers of singletons > 34 weeks' gestation at delivery who expressed intention to breastfeed and lacked contraindications for breastfeeding<br><br>Priority groups: Race and ethnicity<br><br>Analytic sample: 450 women<br><br>Attrition: 0%	Outcomes:<br>Breastfeeding: +*<br><br>Subgroup analysis: No<br><br>Reported unintended consequences: Yes	Setting: Tertiary hospital associated outpatient clinics<br><br>Serve priority groups: Unsure<br><br>Analytic sample: 1 hospital and associated outpatient clinic<br><br>Interventionist: Lactation counselor	Length of intervention: 30-60 minutes<br><br>Policy:<br><br>System: Addition of antenatal breastfeeding education and access to lactation counselor or postnatal lactation support program with hospital visit from a lactation consultant<br><br>Environmental:<br><br>Fidelity: Yes<br><br>Cost: No	Intervention sustained beyond study: Unsure<br><br>Reported sustainability of impact: Yes
Taveras, 2021(80)<br>Simione, 2021(9) (*Suppl. Ref*)<br>Blake-Lamb, 2020(10) (*Suppl. Ref*)<br>Blake-Lamb, 2018(11) (*Suppl. Ref*)<br>Taveras (NCT), 2017(12) (*Suppl. Ref*)<br><br>*First 1,000 Days Program*<br><br>USA	Design: Time series<br><br>Comparator: Standard of care<br><br>Theory: Collective Impact Model	Sample: All expectant mothers and families with singleton 0–2-year-olds<br><br>Priority groups: Low income, race and ethnicity<br><br>Analytic sample:<br>1,645 infants<br>1,530 mothers<br><br>Attrition: 26%	Outcomes:<br>Child weight: +*<br>Maternal diet: +*<br>Maternal weight: +*<br><br>Subgroup analysis: By intervention site<br><br>Reported unintended consequences: No	Setting: Community health centers affiliated with Mass General Brigham, an integrated health system in Massachusetts<br><br>Serve priority groups: Yes<br><br>Analytic sample: 3 community health centers<br><br>Interventionist: Providers, staff, patient navigators, health coaches	Length of intervention: 3 years<br><br>Policy:<br><br>System: Staff and provider training to standardize obesity prevention efforts, newly implemented clinical decision supports in electronic health record, universal screening, standardized educational materials and text messages, and patient navigation<br><br>Environmental:<br><br>Fidelity: Yes	Intervention sustained beyond study: Unsure<br><br>Reported sustainability of impact: No

Supplementary Data

Study Identifiers	Study Elements	Reach	Effectiveness	Adoption	Implementation	Maintenance
					Cost: No	
**Clinical, not specified (n=1)**						
Fan, 2022(13) (*Suppl. Ref*)<br><br>*Lactation consultant led telephone calls*<br><br>Location not reported	Design: Randomized control trial<br><br>Comparator: Standard of care<br><br>Theory: Not reported	Sample: Mothers, not specified<br><br>Priority groups: Unsure<br><br>Analytic sample: Not reported<br><br>Attrition: n/a	Outcomes: Breastfeeding: +*<br><br>Subgroup analysis: Neonatal Unit; mothers with obesity; mothers with depression<br><br>Reported unintended consequences: No	Setting: Not reported<br><br>Serve priority groups: Unsure<br><br>Analytic sample:1 centre<br><br>Interventionist: Lactation consultant	Length of intervention: 1 month<br><br>Policy:<br><br>System: Enhanced standard care with lactation consultant<br><br>Environmental:<br><br>Fidelity: No<br><br>Cost: No	Intervention sustained beyond study: Unsure<br><br>Reported sustainability of impact: No
**Hospital (n=28)**						
Abrahams, 2009(186)<br><br>*Baby-Friendly Hospital Initiative*<br><br>Bolivia, Brazil, Columbia, Dominican Republic, Egypt, Ghana, Indonesia, Jordan, Kenya, Mali, Niger, Peru, Uganda, Zimbabwe	Design: Time series<br><br>Comparator: Historical control<br><br>Theory: Not reported	Sample: Nationally representative samples of children <2 months and <6 months of age who were exclusively breastfed in 72 developing countries<br><br>Priority groups: Unsure<br><br>Analytic sample: Not reported - country-level data<br><br>Attrition: Unable to calculate	Outcomes: Breastfeeding: +<br><br>Subgroup analysis: No<br><br>Reported unintended consequences: No	Setting: Hospitals in developing countries<br><br>Serve priority groups: Yes<br><br>Analytic sample: 14 developing countries<br><br>Interventionist: Not reported	Length of intervention: Policy: n/a<br><br>Policy: Written breastfeeding policy<br><br>System: Organizational practices to incorporate the Ten Steps to Successful Breastfeeding<br><br>Environmental: Rooming-in during hospital stay<br>Support groups<br><br>Fidelity: No<br><br>Cost: No	Intervention sustained beyond study: Unsure<br><br>Reported sustainability of impact: Yes
Anderson, 2005(155)<br><br>*Peer counseling for breastfeeding*<br><br>USA	Design: Randomized control trial<br><br>Comparator: Standard of care<br><br>Theory: Education	Sample: Healthy pregnant women ≤ 32 weeks' gestation without contraindications for breastfeeding<br><br>Priority groups: Low income, race and ethnicity, low education<br><br>Analytic sample: 135 women	Outcomes: Breastfeeding: +*<br><br>Subgroup analysis: No<br><br>Reported unintended consequences: No	Setting: Greater Hartford area, collaboration between the University of Connecticut, the Hispanic Health Council, and Hartford Hospital<br><br>Serve priority groups: Yes<br><br>Analytic sample: 1 hospital	Length of intervention: 4 months<br><br>Policy:<br><br>System: Peer counselor plus conventional support<br><br>Environmental: Peer counselors<br><br>Fidelity: Yes	Intervention sustained beyond study: Unsure<br><br>Reported sustainability of impact: No

Supplementary Data

Study Identifiers	Study Elements	Reach	Effectiveness	Adoption	Implementation	Maintenance
		Attrition: 17%		Interventionist: Peer counselors	Cost: No	
Brodribb, 2013(132)<br><br>*Baby-Friendly Hospital Initiative*<br><br>Australia	Design: Retrospective cohort study<br><br>Comparator: Standard of care<br><br>Theory: Not reported	Sample: All women who had a live single or multiple birth in Queensland from February 1 to May 31, 2010 and an accurate mailing address<br><br>Priority groups: Low income, rural<br><br>Analytic sample: 6,752 women<br><br>Attrition: 6%	Outcomes:<br>Breastfeeding: -*<br><br>Subgroup analysis: Dose-response<br><br>Reported unintended consequences: No	Setting: Hospitals<br><br>Serve priority groups: Yes<br><br>Analytic sample: Not reported<br><br>Interventionist: Hospital staff	Length of intervention: Policy: n/a<br><br>Policy: Written breastfeeding policy<br><br>System: Organizational practices to incorporate the Ten Steps to Successful Breastfeeding<br><br>Environmental: Rooming-in during hospital stay<br>Support groups<br><br>Fidelity: Yes<br><br>Cost: No	Intervention sustained beyond study: Yes<br><br>Reported sustainability of impact: Yes
Chapman, 2013(121)<br>Chapman, 2010(14)<br>(*Suppl. Ref*)<br><br>*Breastfeeding peer counseling*<br><br>USA	Design: Retrospective cohort<br><br>Comparator: No intervention<br><br>Theory: Not reported	Sample: All mother-newborn pairs in Manitoba<br><br>Priority groups: Low income<br><br>Analytic sample: 76,049 mother-newborn dyads<br><br>Attrition: 28%	Outcomes:<br>Breastfeeding: +*<br>Child weight: +*<br><br>Subgroup analysis: Rural-urban<br><br>Reported unintended consequences: No	Setting: Manitoba<br><br>Serve priority groups: Yes<br><br>Analytic sample: Not reported<br><br>Interventionist: Not reported	Length of intervention: 6 months<br><br>Policy: Population/province-level intervention<br><br>System:<br><br>Environmental: Unconditional income supplement during second and third trimester<br><br>Fidelity: No<br><br>Cost: No	Intervention sustained beyond study: Unsure<br><br>Reported sustainability of impact: No
Chapman, 2004(120)<br><br>*Breastfeeding peer counseling*<br><br>USA	Design: Randomized control trial<br><br>Comparator: Standard of care<br><br>Theory: Not reported	Sample: Women aged 18 years or older at ≤ 26 weeks' gestation who are considering breastfeeding, have low income, reside in greater Hartford area, are available for telephone follow-up, and not yet enrolled in the peer	Outcomes:<br>Breastfeeding: +*<br><br>Subgroup analysis: No<br><br>Reported unintended consequences: No	Setting: Hartford Hospital<br><br>Serve priority groups: Yes<br><br>Analytic sample: 1 hospital	Length of intervention: 6 months<br><br>Policy:<br><br>System: Collaborative effort between Hartford Hospital, the Hispanic Health Council, and the University of	Intervention sustained beyond study: Yes<br><br>Reported sustainability of impact: No

Supplementary Data

Study Identifiers	Study Elements	Reach	Effectiveness	Adoption	Implementation	Maintenance
		counseling program and then healthy, full-term singleton<br><br>Priority groups: Low income, race and ethnicity, low education<br><br>Analytic sample: 165 women<br><br>Attrition: 25%		Interventionist: Peer counselors	Connecticut Family Nutrition Program to provide home-and hospital-based peer counseling<br><br>Environmental: Electric breast pumps as needed Peer counselors<br><br>Fidelity: Yes<br><br>Cost: No	
Chiurco, 2015(103)<br><br>*International Board Certified Lactation Consultant*<br><br>Italy	Design: Repeated cross-sectional<br><br>Comparator: Standard of care<br><br>Theory: Not reported	Sample: Mothers of healthy term newborns capable of understanding and speaking Italian<br><br>Priority groups: Unsure<br><br>Analytic sample: 391 mother-child pairs<br><br>Attrition: 3%	Outcomes:<br>Breastfeeding: θ<br><br>Subgroup analysis: No<br><br>Reported unintended consequences: No	Setting: Maternity ward of Institute for Maternal and Child Health, IRCCS "Burlo Garofolo" in Trieste (Friuli Venezia Giulia region)<br><br>Serve priority groups: Unsure<br><br>Analytic sample: 1 hospital<br><br>Interventionist: International Board-Certified Lactation Consultant	Length of intervention: 18 months<br><br>Policy:<br><br>System: Addition of International Board Certified Lactation Consultant to staff; links between hospital and community facilities<br><br>Environmental:<br><br>Fidelity: Yes<br><br>Cost: No	Intervention sustained beyond study: Unsure<br><br>Reported sustainability of impact: No
Donkoh, 2013(179)<br><br>*Baby-Friendly Hospital Initiative*<br><br>Ghana	Design: Non-randomized control trial<br><br>Comparator: Standard of care<br><br>Theory: Not reported	Sample: Healthy infant delivered at ≥ 37 weeks' gestation and willing to attend child welfare clinic services in facility where delivered<br><br>Priority groups: Low income<br><br>Analytic sample: 100 mother-infant pairs<br><br>Attrition: 0%	Outcomes:<br>Breastfeeding: +<br>Child weight: θ<br><br>Subgroup analysis: Age, education, specific Baby-Friendly Hospital Initiative steps<br><br>Reported unintended consequences: No	Setting: Hospitals in Tema and Ashaiman, 2 out of the 15 administrative areas of the Ghana Health Service in the Greater Accra Region<br><br>Serve priority groups: Yes<br><br>Analytic sample: 6 hospitals<br><br>Interventionist: Hospital staff	Length of intervention: Policy: n/a<br><br>Policy: Written breastfeeding policy<br><br>System: Organizational practices to incorporate the Ten Steps to Successful Breastfeeding<br><br>Environmental: Rooming-in during hospital stay<br>Support groups<br><br>Fidelity: Yes<br><br>Cost: No	Intervention sustained beyond study: Yes<br><br>Reported sustainability of impact: Yes

Supplementary Data

Study Identifiers	Study Elements	Reach	Effectiveness	Adoption	Implementation	Maintenance
Gagnon, 1997(147)<br><br>*Early postpartum discharge with nurse visitations*<br><br>Canada	Design: Randomized control trial<br><br>Comparator: Standard of care<br><br>Theory: Not reported	Sample: Parity 0 to 4, normal pregnancy, ability to speak English, French, or Spanish, telephone availability, and residence within 30 minutes of the hospital<br><br>Priority groups: Ethnicity, immigrants<br><br>Analytic sample: 180 women<br><br>Attrition: 50%	Outcomes:<br>Breastfeeding: θ<br>Child weight: +<br><br>Subgroup analysis: Vulnerable subgroups: primiparous, recent immigrants, unmarried, < 11 completed years of education<br><br>Reported unintended consequences: Yes	Setting: Sir Mortimer B. Davis-Jewish General Hospital<br><br>Serve priority groups: Yes<br><br>Analytic sample: 1 hospital<br><br>Interventionist: Nurses	Length of intervention: 1 month<br><br>Policy:<br><br>System: Early postpartum discharge program including nursing care by telephone at standard intervals postpartum and home visits late prenatal and early postpartum<br><br>Environmental:<br><br>Fidelity: No<br><br>Cost: No	Intervention sustained beyond study: No<br><br>Reported sustainability of impact: No
Griffin (NCT), 2020(65)<br><br>*Breastfeeding educational video*<br><br>USA	Design: Randomized control trial, pilot study<br><br>Comparator: Standard of care<br><br>Theory: Not reported	Sample: Nulliparous women gestational age 37 - 42 weeks fluent in English or Spanish who intends to exclusively breastfeed<br><br>Priority groups: Unsure<br><br>Analytic sample: Not reported - clinical trial posting<br><br>Attrition: n/a	Outcomes:<br>Ongoing study (breastfeeding, feeding practices)<br><br>Subgroup analysis: No<br><br>Reported unintended consequences: No	Setting: Women and Infants Hospital in Providence, Rhode Island<br><br>Serve priority groups: Unsure<br><br>Analytic sample: 1 hospital<br><br>Interventionist: Not reported	Length of intervention: 15 minutes<br><br>Policy:<br><br>System: Revise admission procedures to include video about breastfeeding<br><br>Environmental:<br><br>Fidelity: No<br><br>Cost: No	Intervention sustained beyond study: Unsure<br><br>Reported sustainability of impact: No
Gross, 2022(165)<br><br>*Baby-Friendly Hospital Initiative*<br><br>USA	Design: Repeated cross sectional<br><br>Comparator: No intervention<br><br>Theory: Not reported	Sample: Infants born > 37 weeks' gestation without contraindications for breastfeeding participating in WIC<br><br>Priority groups: Low income<br><br>Analytic sample: 22,543 mother-infant dyads<br><br>Attrition: 9%	Outcomes:<br>Breastfeeding: θ<br><br>Subgroup analysis: No<br><br>Reported unintended consequences: No	Setting: Birthing hospitals under Maryland Department of Health<br><br>Serve priority groups: Yes<br><br>Analytic sample: Not reported<br><br>Interventionist: Hospital staff	Length of intervention: Policy: n/a<br><br>Policy: State-level voluntary policy for breastfeeding policies in hospitals<br><br>System: Organizational practices to incorporate the Ten Steps to Successful Breastfeeding	Intervention sustained beyond study: Yes<br><br>Reported sustainability of impact: Yes

Supplementary Data

Study Identifiers	Study Elements	Reach	Effectiveness	Adoption	Implementation	Maintenance
					Environmental:<br>Rooming-in during hospital stay<br>Support groups<br><br>Fidelity: No<br><br>Cost: No	
Grossman, 2009(172)<br><br>*Hospital Education in Lactation Practices (Project HELP)*<br><br>USA	Design: Repeated cross-sectional<br><br>Comparator: Historical control<br><br>Theory: Not reported	Sample: Patients at five hospitals in Massachusetts that have low breastfeeding initiation ratings<br><br>Priority groups: Low income, immigrant<br><br>Analytic sample: 1,180 medical records<br><br>Attrition: 23%	Outcomes:<br>Breastfeeding: +*<br><br>Subgroup analysis: No<br><br>Reported unintended consequences: No	Setting: Community hospitals with low breastfeeding initiation rates<br><br>Serve priority groups: Yes<br><br>Analytic sample: 4 community hospitals<br><br>Interventionist: Public health professionals, perinatal clinicians including a pediatrician and board-certified lactation consultant	Length of intervention: Not reported<br><br>Policy:<br><br>System: Professional development for practitioners<br><br>Environmental:<br><br>Fidelity: No<br><br>Cost: No	Intervention sustained beyond study: Unsure<br><br>Reported sustainability of impact: No
Hannula, 2014(113)<br><br>*Enhanced lactation support*<br><br>Finland	Design: Non-randomized control trial<br><br>Comparator: Standard of care<br><br>Theory: Not reported	Sample: Singleton pregnancy and ability to speak Finnish<br><br>Priority groups: Unsure<br><br>Analytic sample: 705 mothers<br><br>Attrition: 50%	Outcomes:<br>Breastfeeding: +*<br><br>Subgroup analysis: No<br><br>Reported unintended consequences: No	Setting: Public maternity hospitals in the Helsinki Metropolitan area<br><br>Serve priority groups: Unsure<br><br>Analytic sample: 3 public maternity hospitals<br><br>Interventionist: Midwives and clinic staff	Length of intervention: 16 months<br><br>Policy:<br><br>System: Training for clinical staff and use LATCH screening tool with follow-up<br><br>Environmental: Establishment of outpatient breastfeeding clinic<br><br>Fidelity: Yes<br><br>Cost: No	Intervention sustained beyond study: Unsure<br><br>Reported sustainability of impact: No
Hawkins, 2015(166)<br><br>*Baby-Friendly Hospital Initiative*<br><br>USA	Design: Repeated cross-sectional<br><br>Comparator: Standard of care	Sample: Mothers 2 - 6 months postpartum who gave birth in a hospital and for whom breastfeeding initiation information available	Outcomes:<br>Breastfeeding: θ<br><br>Subgroup analysis: Mother's level of education	Setting: Hospitals in 5 states<br><br>Serve priority groups: Unsure	Length of intervention: Policy: n/a<br><br>Policy: Written breastfeeding policy	Intervention sustained beyond study: Yes<br><br>Reported sustainability of impact: Yes

Supplementary Data

Study Identifiers	Study Elements	Reach	Effectiveness	Adoption	Implementation	Maintenance
	Theory: Not reported	through Pregnancy Risk Assessment Monitoring System (PRAMS) data sets<br><br>Priority groups: Low education<br><br>Analytic sample: 25,327 mothers<br><br>Attrition: 0%	Reported unintended consequences: No	Analytic sample: 32 healthcare facilities<br><br>Interventionist: Not reported	System: Organizational practices to incorporate the Ten Steps to Successful Breastfeeding and the International Code of Breastmilk Substitutes<br><br>Environmental: Rooming-in during hospital stay<br>Support groups<br><br>Fidelity: No<br><br>Cost: No	
Hopkinson, 2009(114)<br><br>*Hospital-based breastfeeding clinic*<br><br>USA	Design: randomized control trial<br><br>Comparator: Standard of care<br><br>Theory: Not reported	Sample: Mothers who had low-risk infants, mixed feeding (both breast and formula) in the hospital, had telephones and access to transportation<br><br>Priority groups: Race and ethnicity, immigrants<br><br>Analytic sample: 467 women<br><br>Attrition: 11%	Outcomes:<br>Breastfeeding: +*<br>Complementary feeding: -*<br><br>Subgroup analysis: Treatment received<br><br>Reported unintended consequences: No	Setting: Large community hospital in Houston, Texas<br><br>Serve priority groups: Yes<br><br>Analytic sample: 1 community hospital<br><br>Interventionist: Breastfeeding counselors	Length of intervention: 1 week<br><br>Policy:<br><br>System: Addition of hospital-based breastfeeding clinic visit at 3-7 days postpartum<br><br>Environmental: Creation of breastfeeding spaces in newborn follow-up clinic that included furniture and breast pumps to support breastfeeding<br><br>Fidelity: Yes<br><br>Cost: No	Intervention sustained beyond study: No<br><br>Reported sustainability of impact: Yes
Kair, 2013(133)<br><br>*Baby-Friendly Hospital Initiative*<br><br>USA	Design: Repeated cross-sectional<br><br>Comparator: Historical control<br><br>Theory: Not reported	Sample: Infants born before and after the month of policy implementation<br><br>Priority groups: Low income, race and ethnicity<br><br>Analytic sample: 2,075 infants	Outcomes:<br>Breastfeeding: -*<br>Formula feeding: +*<br><br>Subgroup analysis: No<br><br>Reported unintended consequences: No	Setting: Oregon Health and Science University<br><br>Serve priority groups: Yes<br><br>Analytic sample: 1 mother and baby unit<br><br>Interventionist: Physicians and nurses	Length of intervention: Policy: n/a<br><br>Policy: Policy to cease routine offering of pacifiers to breastfeeding newborns<br><br>System: Organizational practices to deter provision of pacifiers	Intervention sustained beyond study: Unsure<br><br>Reported sustainability of impact: No

Supplementary Data

Study Identifiers	Study Elements	Reach	Effectiveness	Adoption	Implementation	Maintenance
		Attrition: 0%			Environmental: Bedside reminders discouraging pacifier use in the hospital<br><br>Fidelity: No<br><br>Cost: No	
Kellams, 2014(15) (*Suppl. Ref*)<br><br>*Physician performance improvement project*<br><br>USA	Design: Single group, pre-post design<br><br>Comparator: No control<br><br>Theory: Not reported	Sample: Archived charts<br><br>Priority groups: Unsure<br><br>Analytic sample: 5,827 charts<br><br>Attrition: 0%	Outcomes: Breastfeeding: +*<br><br>Subgroup analysis: No<br><br>Reported unintended consequences: No	Setting: American Board of Pediatrics approved activity - Breastfeeding Friendly Performance Improvement Project<br><br>Serve priority groups: Unsure<br><br>Analytic sample: Not reported<br><br>Interventionist: Physicians	Length of intervention: Not reported<br><br>Policy:<br><br>System: Breastfeeding Friendly Performance Improvement Project and professional development<br><br>Environmental:<br><br>Fidelity: Yes<br><br>Cost: No	Intervention sustained beyond study: Unsure<br><br>Reported sustainability of impact: Yes
Kola, 2016(16) (*Suppl. Ref*)<br><br>*Baby-Friendly Hospital Initiative*<br><br>USA	Design: Repeated cross sectional<br><br>Comparator: National data set<br><br>Theory: Not reported	Sample: Not reported<br><br>Priority groups: Unsure<br><br>Analytic sample: Not reported<br><br>Attrition: n/a	Outcomes: Breastfeeding: +*<br><br>Subgroup analysis: No<br><br>Reported unintended consequences: No	Setting: Hospital<br><br>Serve priority groups: Unsure<br><br>Analytic sample: 1 hospital<br><br>Interventionist: Lactation consultant, nursing staff, and physicians	Length of intervention: Policy: n/a<br><br>Policy: Written breastfeeding policy<br><br>System: Organizational practices to support successful breastfeeding<br><br>Environmental: Rooming in<br><br>Fidelity: No<br><br>Cost: No	Intervention sustained beyond study: Unsure<br><br>Reported sustainability of impact: No
Merewood, 2019(129)<br>Bumham, 2022(17) (*Suppl. Ref*)<br>Bugg, 2017(18)(*Suppl. Ref*)<br>*Baby-Friendly Hospital Initiative*<br><br>USA	Design: Cohort<br><br>Comparator: No control<br><br>Theory: Not reported	Sample: All infants not scoring on the Joint Commission Perinatal Core Measure 5 Exclusion Criteria<br><br>Priority groups: Race and ethnicity	Outcomes: Breastfeeding: +*<br><br>Subgroup analysis: Race<br><br>Reported unintended consequences: Yes	Setting: Hospitals in Mississippi, Tennessee, Texas, and greater New Orleans area<br><br>Serve priority groups: Yes	Length of intervention: Policy: n/a<br><br>Policy: Hospital policy about breastfeeding<br><br>System: Collaborations with agencies that serve women and children and	Intervention sustained beyond study: Yes<br><br>Reported sustainability of impact: Yes

Supplementary Data

Study Identifiers	Study Elements	Reach	Effectiveness	Adoption	Implementation	Maintenance
		Analytic sample: 39,272 infants<br><br>Attrition: n/a		Analytic sample: 31 hospitals<br><br>Interventionist: Physician lead, local consultants, and key nursing leaders from around the state	communities through a national organization to promote breastfeeding in communities of color<br><br>Training and coaching to prepare for charting and data collection, BabyFriendly USA assessment, skills to implement policy<br><br>Organizational practices to incorporate the Ten Steps to Successful Breastfeeding<br><br>Environmental: Reaching Our Sisters Everwhere provided support for community changes in breastfeeding practices<br><br>Fidelity: Yes<br><br>Cost: No	
Merten, 2005(161)<br><br>*Baby-Friendly Hospital Initiative*<br><br>Switzerland	Design: Retrospective cohort study<br><br>Comparator: National data set<br><br>Theory: Not reported	Sample: Mothers who had given birth within the past 9 months in their communes<br><br>Priority groups: Unsure<br><br>Analytic sample: 2,861 infants<br><br>Attrition: 7%	Outcomes:<br>Breastfeeding: +<br><br>Subgroup analysis: Baby-friendly hospital compliance<br><br>Reported unintended consequences: No	Setting: Regional, community based, mother-child health services and hospitals<br><br>Serve priority groups: Unsure<br><br>Analytic sample: 146 health facilities<br><br>Interventionist: Not reported	Length of intervention: Policy: n/a<br><br>Policy: Hospital policy about breastfeeding<br><br>System: Organizational practices to support successful breastfeeding<br><br>Environmental: Rooming-in during hospital stay<br>Support groups<br><br>Fidelity: Yes<br><br>Cost: No	Intervention sustained beyond study: Yes<br><br>Reported sustainability of impact: Yes
Milinco, 2020(104)<br><br>*Enhanced lactation support*	Design: Randomized control trial	Sample: Women with a singleton pregnancy who expressed intention to breastfeed and without	Outcomes:<br>Breastfeeding: ϑ<br><br>Subgroup analysis: No	Setting: Maternity ward of IRCCS Burlo Garofolo, a research institute and third level	Length of intervention: 10 months<br><br>Policy:	Intervention sustained beyond study: Unsure

Supplementary Data

Study Identifiers	Study Elements	Reach	Effectiveness	Adoption	Implementation	Maintenance
Italy	Comparator: Standard of care<br><br>Theory: Not reported	contraindications for breastfeeding<br><br>Priority groups: Unsure<br><br>Analytic sample: 188 mother-newborn pairs<br><br>Attrition: 10%	Reported unintended consequences: No	hospital located in Trieste<br><br>Serve priority groups: Unsure<br><br>Analytic sample: 1 maternity ward<br><br>Interventionist: Midwives and nurses	System: Clinical practice to provide breastfeeding support in the maternity ward<br><br>Environmental:<br><br>Fidelity: No<br><br>Cost: No	Reported sustainability of impact: Yes
Mottl-Santiago, 2007(97)<br>Mottl-Santiago (NCT), 2015(19) (*Suppl. Ref*)<br><br>*Best Beginnings for Babies Birth Sister Program*<br><br>USA	Design: Retrospective cohort study<br><br>Comparator: Standard of care<br><br>Theory: Not reported	Sample: Women giving birth to singleton, live infants ≥ 37 weeks<br><br>Priority groups: Race and ethnicity, low income<br><br>Analytic sample: 11,471 mothers<br><br>Attrition: 0.2%	Outcomes:<br>Breastfeeding: +*<br><br>Subgroup analysis: Primiparous/multiparous<br><br>Reported unintended consequences: No	Setting: Boston Medical Center's Birth Sister doula program<br><br>Serve priority groups: Yes<br><br>Analytic sample: 1 hospital doula program<br><br>Interventionist: Lay doulas	Length of intervention: 3 months<br><br>Policy:<br><br>System: Referral processes<br><br>Environmental: Peer support from doulas<br><br>Fidelity: No<br><br>Cost: Yes	Intervention sustained beyond study: Yes<br><br>Reported sustainability of impact: No
Nickel, 2011(183)<br><br>*Baby-Friendly Hospital Initiative*<br><br>USA	Design: Non-randomized control trial<br><br>Comparator: Delayed<br><br>Theory: Organizational Readiness to Change	Sample: Not reported<br><br>Priority groups: Low income, race and ethnicity<br><br>Analytic sample: Not reported<br><br>Attrition: n/a	Outcomes:<br>Breastfeeding: +<br><br>Subgroup analysis: No<br><br>Reported unintended consequences: No	Setting: Hospitals in North Carolina<br><br>Serve priority groups: Yes<br><br>Analytic sample: 6 hospitals<br><br>Interventionist: Hospital administration and staff	Length of intervention: Policy: n/a<br><br>Policy: Written breastfeeding policy<br><br>System: Organizational practices to incorporate the Ten Steps to Successful Breastfeeding<br><br>Environmental: Rooming-in during hospital stay<br>Support groups<br><br>Fidelity: Yes<br><br>Cost: No	Intervention sustained beyond study: Unsure<br><br>Reported sustainability of impact: No
Patel, 2018(127)<br><br>*Cell phone counselling calls and text messages*	Design: Randomized control trial, pilot study	Sample: Pregnant women in third trimester who do not have complications that could affect breastfeeding	Outcomes:<br>Breastfeeding: +*<br>Complementary feeding: +<br>Child weight: +*	Setting: Urban, public, maternity hospitals in Nagpur	Length of intervention: 9 months<br><br>Policy:	Intervention sustained beyond study: Unsure<br><br>Reported sustainability of impact: No

Supplementary Data

Study Identifiers	Study Elements	Reach	Effectiveness	Adoption	Implementation	Maintenance
India	Comparator: Baby Friendly Hospital Initiative re-training<br><br>Theory: Not reported	Priority groups: Low income<br><br>Analytic sample: 951 women<br><br>Attrition: 8%	Subgroup analysis: No<br><br>Reported unintended consequences: No	Serve priority groups: Yes<br><br>Analytic sample: 4 hospitals<br><br>Interventionist: Auxiliary nurse midwives	System: Baby Friendly Hospital Initiative re-training and incorporation of cell phone counselling<br><br>Environmental: Provision of cell phone, free recharge vouchers and subsidized prepaid calling card for cell phone counselling<br><br>Fidelity: No<br><br>Cost: Yes	
Rasmussen, 2011(119)<br><br>*Bassett Improving Breastfeeding*<br><br>USA	Design: Randomized control trial<br><br>Comparator: Standard of care<br><br>Theory: Not reported	Sample: Singleton pregnancy 35 weeks of gestational age with pre-pregnancy BMI >29 kg/m^2, aged >19 years and intentions to breastfeed, with no history of breast surgery<br><br>Priority groups: Rural, obesity<br><br>Analytic sample:<br>BIBS 1: 40 women<br>BIBS 2: 34 women<br><br>Attrition:<br>BIBS 1: 20%<br>BIBS 2: 13%	Outcomes:<br>Breastfeeding: -*<br><br>Subgroup analysis: BIBS 1: Those who received prescribed calls<br><br>Reported unintended consequences: No	Setting: Hospital in rural New York state<br><br>Serve priority groups: Yes<br><br>Analytic sample: 1 hospital<br><br>Interventionist: Hospital nurses and lactation consultants	Length of intervention: 5 days<br><br>Policy:<br><br>System: BIBS 1: Rooming in and Mother-Baby Assessment tool during breastfeeding sessions during each 8-hour shift and additional support from lactation consultant; time limits for visitors<br><br>Environmental: BIBS 2: breast pump<br><br>Fidelity: Yes<br><br>Cost: No	Intervention sustained beyond study: No<br><br>Reported sustainability of impact: No
Rosen, 2008(174)<br><br>*Prenatal breastfeeding education*<br><br>USA	Design: Retrospective cohort study<br><br>Comparator: No intervention<br><br>Theory: Not reported	Sample: Patients at least 6 months postpartum who attended a lactation education class or expressed a desire to breastfeed and had full-term, singleton pregnancy<br><br>Priority groups: Race and Ethnicity	Outcomes:<br>Breastfeeding: +*<br><br>Subgroup analysis: No<br><br>Reported unintended consequences: No	Setting: Army medical center<br><br>Serve priority groups: Yes<br><br>Analytic sample: 1 Army medical center	Length of intervention: 2 hours<br><br>Policy:<br><br>System: Offer two education models - one-on-one with a lactation consultant and pediatrician and 2-hour	Intervention sustained beyond study: Yes<br><br>Reported sustainability of impact: No

Supplementary Data

Study Identifiers	Study Elements	Reach	Effectiveness	Adoption	Implementation	Maintenance
		Analytic sample: 194 participants<br><br>Attrition: n/a		Interventionist: Lactation consultant and pediatrician	class with structured curriculum<br><br>Environmental: Support group<br><br>Fidelity: No<br><br>Cost: No	
Tarrant, 2011(175)<br><br>*Baby-Friendly Hospital Initiative*<br><br>Hong Kong	Design: Cohort<br><br>Comparator: No control<br><br>Theory: Not reported	Sample: Singleton pregnancies with intention to breastfeed who lived in Hong Kong for more than one year, spoke Cantonese, and did not have serious medical or obstetrical complications<br><br>Priority groups: Unsure<br><br>Analytic sample: 1,242 mother infant pairs<br><br>Attrition: 12%	Outcomes:<br>Breastfeeding: +<br><br>Subgroup analysis: No<br><br>Reported unintended consequences: No	Setting: Public hospitals<br><br>Serve priority groups: Unsure<br><br>Analytic sample: 4 public hospitals<br><br>Interventionist: Not reported	Length of intervention: Policy: n/a<br><br>Policy: Written breastfeeding policy<br><br>System: Organizational practices to support successful breastfeeding<br><br>Environmental: Rooming-in during hospital stay<br><br>Fidelity: Yes<br><br>Cost: No	Intervention sustained beyond study: Yes<br><br>Reported sustainability of impact: No
Tarrant, 2015(162)<br>Tarrant, 2016(20)<br>(*Suppl. Ref*)<br><br>*Hospital formula purchase policy*<br><br>Hong Kong	Design: Cohort<br><br>Comparator: No intervention<br><br>Theory: Not reported	Sample: Singleton pregnancies with intention to breastfeed who lived in Hong Kong for more than one year, spoke Cantonese, and did not have serious medical or obstetrical complications<br><br>Priority groups: Unsure<br><br>Analytic sample: 2,560 mother infant pairs<br><br>Attrition: 5%	Outcomes:<br>Breastfeeding: +*<br>Formula feeding: +*<br><br>Subgroup analysis: No<br><br>Reported unintended consequences: No	Setting: Public hospitals<br><br>Serve priority groups: Unsure<br><br>Analytic sample: 4 public hospitals<br><br>Interventionist: Not reported	Length of intervention: Policy: n/a<br><br>Policy: Policy to purchase infant formula at market price<br><br>System:<br><br>Environmental:<br><br>Fidelity: No<br><br>Cost: No	Intervention sustained beyond study: Yes<br><br>Reported sustainability of impact: Yes
Winterburn, 2000(158)<br><br>*Duration of postnatal stay*<br><br>United Kingdom	Design: Randomized control trial<br><br>Comparator: Other<br><br>Theory: Not reported	Sample: Nulliparous women in the third trimester of pregnancy willing to attempt breastfeeding<br><br>Priority groups: Unsure	Outcomes:<br>Breastfeeding: θ<br><br>Subgroup analysis: Normal delivery	Setting: Large teaching hospital<br><br>Serve priority groups: Unsure	Length of intervention: 1 week<br><br>Policy:<br><br>System: Short (6 – 48 hours) or long (>48	Intervention sustained beyond study: No<br><br>Reported sustainability of impact: No

Supplementary Data

Study Identifiers	Study Elements	Reach	Effectiveness	Adoption	Implementation	Maintenance
		Analytic sample: 248 women<br><br>Attrition: 3%	Reported unintended consequences: No	Analytic sample: 1 teaching hospital<br><br>Interventionist: Not reported	hours) postpartum hospital stay with midwife support for breastfeeding in community or hospital, respectively<br><br>Environmental:<br><br>Fidelity: No<br><br>Cost: No	
**Pediatric primary care (n=26)**						
Chwah, 2016(171)<br><br>*Midwifery-led intervention group*<br><br>Australia	Design: Retrospective cohort<br><br>Comparator: Standard of care<br><br>Theory: Not reported	Sample: All live born singleton pregnancies with a documented maternal BMI > 30 at booking<br><br>Priority groups: Obesity<br><br>Analytic sample: 213 women<br><br>Attrition: n/a	Outcomes:<br>Breastfeeding: +*<br>Child weight: θ<br><br>Subgroup analysis: No<br><br>Reported unintended consequences: No	Setting: St. George or Sutherland hospitals<br><br>Serve priority groups: Unsure<br><br>Analytic sample: 2 hospitals<br><br>Interventionist: Midwives	Length of intervention: 6 months<br><br>Policy:<br><br>System: Change and standardize clinical care for pregnant women with BMI >35<br><br>Environmental:<br><br>Fidelity: Yes<br><br>Cost: No	Intervention sustained beyond study: Yes<br><br>Reported sustainability of impact: No
Corriveau, 2013(95)<br><br>*Office protocol for breastfeeding*<br><br>USA	Design: Repeated cross-sectional<br><br>Comparator: Standard of care<br><br>Theory: Not reported	Sample: Healthy, singleton infants ≥ 37 weeks' gestation at birth<br><br>Priority groups: Low income, rural<br><br>Analytic sample: Pre-intervention: 376 mother-infant dyads<br>Post-intervention: 381 mother-infant dyads<br><br>Attrition: n/a	Outcomes:<br>Breastfeeding: +*<br><br>Subgroup analysis: Primiparous/multiparous<br><br>Reported unintended consequences: No	Setting: Pediatric primary care practices in northern Virginia<br><br>Serve priority groups: Yes<br><br>Analytic sample: 2 pediatric primary care settings<br><br>Interventionist: International Board Certified Lactation Consultant and clinic staff	Length of intervention: 10 months<br><br>Policy: Office policy manual<br><br>System: Training staff in breastfeeding curriculum; provision of on-site lactation consultant support during initial newborn appointments<br><br>Environmental: Clinical space to include furniture and supplies to provide in-office lactation support<br>Support group	Intervention sustained beyond study: Unsure<br><br>Reported sustainability of impact: No

Supplementary Data

Study Identifiers	Study Elements	Reach	Effectiveness	Adoption	Implementation	Maintenance
					Fidelity: Yes<br><br>Cost: No	
Döring, 2016(82)<br>Döring, 2016(21) (*Suppl. Ref*)<br>Döring, 2014(22) (*Suppl. Ref*)<br><br>*Motivational interviewing in child health services (PRIMROSE)*<br><br>Sweden	Design: Cluster randomized control trial<br><br>Comparator: Standard of care<br><br>Theory: Social Cognitive Theory, Learning Theory, Cognitive Behavior Therapy	Sample: First time parents who speak Swedish who have 5 - 6-month-old infants<br><br>Priority groups: Unsure<br><br>Analytic sample:<br>1,148 children<br>1,050 mothers<br><br>Attrition:<br>Children: 16%<br>Mothers: 23%	Outcomes:<br>Child diet: +*<br>Child weight: θ<br>Maternal diet: θ<br>Maternal weight: θ<br><br>Subgroup analysis: Completers of all motivational interview sessions; delivered by nurses who completed training<br><br>Reported unintended consequences: No	Setting: Child Health Care Centers<br><br>Serve priority groups: Unsure<br><br>Analytic sample: 59 Child Healthcare Centers<br><br>Interventionist: Nurses	Length of intervention: 39 months<br><br>Policy:<br><br>System: Intervention embedded in Swedish child health services; training for nurses<br><br>Environmental:<br><br>Fidelity: Yes<br><br>Cost: Yes	Intervention sustained beyond study: Unsure<br><br>Reported sustainability of impact: No
French, 2012(146)<br>Groner, 2009(23) (*Suppl. Ref*)<br><br>*Mother-centered anticipatory guidance (MOMS Project)*<br><br>USA	Design: Cluster randomized control trial<br><br>Comparator: Standard of care<br><br>Theory: Not reported	Sample: Biological mothers of healthy full-term infants ≤ 2 months of age<br><br>Priority groups: Low income, race and ethnicity<br><br>Analytic sample: 191 mother-child dyads<br><br>Attrition: 38%	Outcomes:<br>Complementary feeding: +<br>Child weight: θ<br>Maternal diet: θ<br><br>Subgroup analysis: No<br><br>Reported unintended consequences: No	Setting: Pediatric primary care clinics in Nationwide Children's Hospital Primary Care Network in Columbus, Ohio<br><br>Serve priority groups: Yes<br><br>Analytic sample: 3 clinics<br><br>Interventionist: Clinic physicians, nurses, and medical assistants	Length of intervention: 12 months<br><br>Policy:<br><br>System: Intervention delivered at standard well-child visits and embedded brief reminders for clinicians in anticipatory guidance checklists in infant charts<br><br>Environmental:<br><br>Fidelity: Yes<br><br>Cost: No	Intervention sustained beyond study: No<br><br>Reported sustainability of impact: No
Hale, 2023(128)<br><br>*Raising Infants to Be Smart Eaters (RAISE)*<br><br>Canada	Design: Non-randomized control trial, pilot study<br><br>Comparator: Standard of care<br><br>Theory: Satter Division of Responsibility in Feeding	Sample: Women who delivered in two communities between March 2018 and January 2019<br><br>Priority groups: No<br><br>Analytic sample: 17 mothers<br><br>Attrition: 37%	Outcomes:<br>Child diet: θ<br>Feeding style: -<br><br>Subgroup analysis: No<br><br>Reported unintended consequences: No	Setting: Communities<br><br>Serve priority groups: Unsure<br><br>Analytic sample: 2 communities<br><br>Interventionist: Public health nurses	Length of intervention: 18 months<br><br>Policy:<br><br>System: Incorporation of brief discussion to well-baby visits<br><br>Environmental:<br><br>Fidelity: Yes	Intervention sustained beyond study: No<br><br>Reported sustainability of impact: No

Supplementary Data

Study Identifiers	Study Elements	Reach	Effectiveness	Adoption	Implementation	Maintenance
					Cost: No	
Heinig (NCT), 2015(24) (*Suppl. Ref*)<br><br>*Supporting Baby Behavior Through Pediatric Offices*<br><br>USA	Design: Randomized control trial<br><br>Comparator: Standard of care<br><br>Theory: Not reported	Sample: Women with primiparous, singleton pregnancy currently enrolled in WIC who speaks and reads English or Spanish, has access to telephone and internet, and did not smoke during pregnancy<br><br>Priority groups: Low income, ethnicity<br><br>Analytic sample: Not reported<br><br>Attrition: n/a	Outcomes:<br>Ongoing study (breastfeeding, feeding practices, child diet, child weight)<br><br>Subgroup analysis: No<br><br>Reported unintended consequences: No	Setting: Pediatric offices<br><br>Serve priority groups: Yes<br><br>Analytic sample: Not reported<br><br>Interventionist: Healthcare providers and medical staff	Length of intervention: 6 months<br><br>Policy:<br><br>System: Training for health care providers to align health care messaging with Baby Behavior education for WIC participants<br><br>Environmental:<br><br>Fidelity: No<br><br>Cost: No	Intervention sustained beyond study: Unsure<br><br>Reported sustainability of impact: No
Howard, 2018(25) (*Suppl. Ref*)<br><br>*Primary care obesity prevention*<br><br>Not reported	Design: Non-randomized control trial<br><br>Comparator: No detail provided<br><br>Theory: Not reported	Sample: Newborn infants presenting for well-child care<br><br>Priority groups: Unsure<br><br>Analytic sample: 85 families<br><br>Attrition: 58%	Outcomes:<br>Breastfeeding: +<br>Feeding style: +*<br>Child weight: Not reported<br><br>Subgroup analysis: No<br><br>Reported unintended consequences: No	Setting: Primary care - private practice offices<br><br>Serve priority groups: Unsure<br><br>Analytic sample: 4 practices<br><br>Interventionist: Physicians	Length of intervention: Not reported<br><br>Policy:<br><br>System: Physician training<br>Incorporation of obesity-prevention handouts and small group visits to routine care<br><br>Environmental:<br><br>Fidelity: No<br><br>Cost: No	Intervention sustained beyond study: Unsure<br><br>Reported sustainability of impact: No
Laws, 2021(26) (*Suppl. Ref*)<br><br>*INFANT*<br><br>Australia	Design: Non-randomized control, hybrid effectiveness-implementation trial (Type II)<br><br>Comparator: Standard of care<br><br>Theory: Not reported	Sample: First time parents of an infant aged 0 - 3 months who communicate in English and own a phone that accesses the internet<br><br>Priority groups: Unsure<br><br>Analytic sample:<br>Goal: 2,000 infants<br><br>Attrition:<br>Estimated: 33%	Outcomes:<br>Ongoing study (breastfeeding, child diet, child weight)<br><br>Subgroup analysis: No<br><br>Reported unintended consequences: No	Setting: Maternal and child, population, and Aboriginal health services in Victoria<br><br>Serve priority groups: Unsure<br><br>Analytic sample: Not reported<br><br>Interventionist: Maternal Child Health nurses, dietitians, community	Length of intervention: 18 months<br><br>Policy:<br><br>System: Promoted as flagship state-wide intervention<br>Group-based intervention delivered via established first time parents' groups by universal Maternal and Child Health services	Intervention sustained beyond study: Unsure<br><br>Reported sustainability of impact: No

Supplementary Data

Study Identifiers	Study Elements	Reach	Effectiveness	Adoption	Implementation	Maintenance
				health workers or nominated personnel	Environmental:<br><br>Fidelity: Yes<br><br>Cost: Yes	
Machuca, 2016(88)<br><br>*Well baby group care*<br><br>USA	Design: Non-randomized control trial<br><br>Comparator: Standard of care<br><br>Theory: Transtheoretical Model, Social Learning Theory, Freirian Framework	Sample: Infants with a visit by 2 months and again at least the 24- or 30-month well-child care visit<br><br>Priority groups: Low income, race and ethnicity<br><br>Analytic sample: 187 mother-infant dyads<br><br>Attrition: 0%	Outcomes: Child weight: +*<br><br>Subgroup analysis: No<br><br>Reported unintended consequences: No	Setting: Federally Qualified Health Center in the South Bronx, New York<br><br>Serve priority groups: Yes<br><br>Analytic sample: 1 Federally Qualified Health Center<br><br>Interventionist: Pediatrician and Registered Dietician	Length of intervention: 17 months<br><br>Policy:<br><br>System: Well Baby Group is a model of group care for routine well-child care that adds three additional visits<br><br>Environmental: Peer support<br><br>Fidelity: No<br><br>Cost: No	Intervention sustained beyond study: Unsure<br><br>Reported sustainability of impact: No
Martin-Iglesias, 2011(27) (*Suppl. Ref*)<br><br>*Healthcare staff training*<br><br>Spain	Design: Cluster randomized control trial<br><br>Comparator: Standard of care<br><br>Theory: Not reported	Sample: Mothers and infants without contraindications for breastfeeding<br><br>Priority groups: Unsure<br><br>Analytic sample: Not reported<br><br>Attrition: n/a	Outcomes: Ongoing study (breastfeeding)<br><br>Subgroup analysis: No<br><br>Reported unintended consequences: No	Setting: Primary Healthcare Centres in Leganés, Madrid<br><br>Serve priority groups: Unsure<br><br>Analytic sample: Not reported<br><br>Interventionist: Doctors and nurses	Length of intervention: 12 months<br><br>Policy:<br><br>System: Training for health professionals; appointment of a person responsible for promoting breastfeeding<br><br>Environmental:<br><br>Fidelity: No<br><br>Cost: No	Intervention sustained beyond study: Unsure<br><br>Reported sustainability of impact: No
Matvienko-Sikar, 2019(67)<br>Matvienko-Sikar, 2019(28)(*Suppl. Ref*)<br><br>*Choosing Healthy Eating for Infant Health (CHErIsH)*<br><br>Ireland	Design: Single group, pre-post design, feasibility study<br><br>Comparator: No control<br><br>Theory: Behaviour Change Wheel, Behaviour Change Technique Taxonomy,	Sample: Parent of an infant ≤6 weeks who intends to attend a the primary care centre for child's vaccination visits<br><br>Priority groups: Unsure<br><br>Analytic sample: Not reported	Outcomes: Ongoing study (breastfeeding, complementary feeding, child diet, child weight)<br><br>Subgroup analysis: No<br><br>Reported unintended consequences: No	Setting: Mallow Primary Healthcare Centre in Cork<br><br>Serve priority groups: Unsure<br><br>Analytic sample: 1 primary healthcare center	Length of intervention: 11 months<br><br>Policy:<br><br>System: Incorporation of intervention to routine vaccination visits<br>Local opinion leader, incentives, resources,	Intervention sustained beyond study: Unsure<br><br>Reported sustainability of impact: No

Supplementary Data

Study Identifiers	Study Elements	Reach	Effectiveness	Adoption	Implementation	Maintenance
	Theory of Planned Behaviour, Responsive Feeding, Social Cognitive Theory, Family Systems Theory	Attrition: n/a		Interventionist: Practice nurses or general practitioners	and educational materials to support training Electronic prompts for healthcare providers Local technical assistance<br><br>Environmental:<br><br>Fidelity: Yes<br><br>Cost: Yes	
Minkovitz, 2001(167)<br><br>*Healthy Steps for Young Children program*<br><br>USA	Design: Randomized control and quasi experimental<br><br>Comparator: Standard of care<br><br>Theory: Not reported	Sample: Children of parents who spoke English or Spanish fluently<br><br>Priority groups: Low income, race and ethnicity, adolescent mothers<br><br>Analytic sample: 4,896 families<br><br>Attrition: 12%	Outcomes: Breastfeeding: + Complementary feeding: +<br><br>Subgroup analysis: No<br><br>Reported unintended consequences: No	Setting: Group practices, hospital-based clinics, and pediatric practices in health maintenance organizations<br><br>Serve priority groups: Yes<br><br>Analytic sample: 15 sites<br><br>Interventionist: Pediatrician, family physician, nurse practitioners, and early childhood development specialists	Length of intervention: 3 years<br><br>Policy:<br><br>System: Enhanced well-child care visits with home visits; child development telephone intervention line; links to community resources<br><br>Environmental: Peer groups<br><br>Fidelity: Yes<br><br>Cost: No	Intervention sustained beyond study: Unsure<br><br>Reported sustainability of impact: No
Palacios (NCT), 2021(29)(*Suppl. Ref*)<br><br>*Baby Feed*<br><br>USA	Design: Randomized control trial<br><br>Comparator: Standard of care<br><br>Theory: Not reported	Sample: Parent or guardian of an infant age 4 to 12 months, who is healthy and with any weight status<br><br>Priority groups: Unsure<br><br>Analytic sample: Not reported<br><br>Attrition: n/a	Outcomes: Ongoing study (child diet, child weight)<br><br>Subgroup analysis: No<br><br>Reported unintended consequences: No	Setting: Borinquen Health Care Center in Miami<br><br>Serve priority groups: Unsure<br><br>Analytic sample: 1 health care center<br><br>Interventionist: Clinicians	Length of intervention: 3 months<br><br>Policy:<br><br>System: Baby Feed platform to evaluate infant diets online and provide immediate results to health care providers and recommendations for providers and parents<br><br>Environmental:<br><br>Fidelity: No<br><br>Cost: No	Intervention sustained beyond study: Unsure<br><br>Reported sustainability of impact: No

Supplementary Data

Study Identifiers	Study Elements	Reach	Effectiveness	Adoption	Implementation	Maintenance
Paul (NCT), 2005(30) (*Suppl. Ref*)<br><br>*Healthy Sleeping and Feeding During Infancy*<br><br>USA	Design: Randomized control trial<br><br>Comparator: Standard of care<br><br>Theory: Not reported	Sample: Primiparous mother, singleton pregnancy<br><br>Priority groups: Unsure<br><br>Analytic sample: Not reported<br><br>Attrition: n/a	Outcomes:<br>Ongoing study (child weight)<br><br>Subgroup analysis: No<br><br>Reported unintended consequences: No	Setting: Primary care setting<br><br>Serve priority groups: Unsure<br><br>Analytic sample: Not reported<br><br>Interventionist: Not reported	Length of intervention: 4 months<br><br>Policy:<br><br>System: Incorporation of educational program into regular primary care visits<br><br>Environmental:<br><br>Fidelity: No<br><br>Cost: No	Intervention sustained beyond study: Unsure<br><br>Reported sustainability of impact: No
Rybak, 2023(143) Children's Hospital Medical Center (NCT), 2021(31)(*Suppl. Ref*)<br><br>*Teaching Healthy Responsive Parenting During Infancy to Promote Vital Growth and dEvelopment (THRIVE)*<br><br>USA	Design: Randomized control trial, pilot study<br><br>Comparator: Attention control<br><br>Theory: ORBIT model of intervention development	Sample: Mothers of singleton infants who speak English<br><br>Priority groups: Low income, race<br><br>Analytic sample: 49 mother-infant dyads<br><br>Attrition: 11%	Outcomes:<br>Feeding practice: not reported<br>Child weight: +*<br><br>Subgroup analysis: No<br><br>Reported unintended consequences: Yes	Setting: Urban pediatric primary care practice<br><br>Serve priority groups: Yes<br><br>Analytic sample: 3 clinics<br><br>Interventionist: Postdoctoral psychology fellow with training in Integrated Behavioral Health with infants	Length of intervention: 6 months<br><br>Policy:<br><br>System: Intervention sessions with Integrated Behavioral Health Specialist integrated into routine well-child care<br><br>Environmental:<br><br>Fidelity: No<br><br>Cost: No	Intervention sustained beyond study: Unsure<br><br>Reported sustainability of impact: Yes
Sabo, 2018(32)(*Suppl. Ref*)<br><br>*Healthcare staff training*<br><br>USA	Design: Repeated cross-sectional<br><br>Comparator: No control<br><br>Theory: Not reported	Sample: Patient charts each of newborn and 1 and 6-month olds<br><br>Priority groups: Unsure<br><br>Analytic sample: 30 patient charts<br><br>Attrition: n/a	Outcomes:<br>Breastfeeding: +<br><br>Subgroup analysis: No<br><br>Reported unintended consequences: No	Setting: Outpatient practice<br><br>Serve priority groups: Unsure<br><br>Analytic sample: Not reported<br><br>Interventionist: Medical residents	Length of intervention: 9 months<br><br>Policy:<br><br>System: Addition of monthly lactation reviews for medical residents and nursing and medical assistant education<br><br>Environmental:<br><br>Fidelity: Yes<br><br>Cost: No	Intervention sustained beyond study: Yes<br><br>Reported sustainability of impact: No

Supplementary Data

Study Identifiers	Study Elements	Reach	Effectiveness	Adoption	Implementation	Maintenance
Sanders, 2021(153)<br>Sanders, 2014(33)<br>(*Suppl. Ref*)<br>Heerman, 2022(68)<br>Heerman, 2019(34)<br>(*Suppl. Ref*)<br><br>*Greenlight (Plus)*<br><br>USA	Design: Cluster randomized control trial<br><br>Comparator: Attention control<br><br>Theory: Social Cognitive Theory, Health literacy principles	Sample: Child presenting for a 2-month well child visit, born ≥ 34 weeks, whose parents speak Spanish or English<br><br>Priority groups: Low income, race and ethnicity<br><br>Analytic sample: 802 parent-child dyads<br><br>Attrition: 7%	Outcomes:<br>Feeding practices: Not reported<br>Child diet: Not reported<br>Child weight: +<br><br>Subgroup analysis: No<br><br>Reported unintended consequences: No	Setting: Academic-medical-center-based pediatric residency clinics<br><br>Serve priority groups: Yes<br><br>Analytic sample: 4 clinics<br><br>Interventionist: Resident physicians	Length of intervention: 2 years<br><br>Policy:<br><br>System: Health care provider training in health communication and incorporation of intervention and assessment during regular preventive care visits<br><br>Environmental:<br><br>Fidelity: Yes<br><br>Cost: No	Intervention sustained beyond study: Unsure<br><br>Reported sustainability of impact: Yes
Sangalli, 2021(116)<br><br>*Healthcare staff training*<br><br>Brazil	Design: Cluster randomized control trial<br><br>Comparator: Standard of care<br><br>Theory: Not reported	Sample: Pregnant women attending participating centers<br><br>Priority groups: Low income<br><br>Analytic sample: 545 mother-child dyads<br><br>Attrition: 76%	Outcomes:<br>Breastfeeding: Not reported<br>Child diet: +*<br>Child weight: θ<br><br>Subgroup analysis: No<br><br>Reported unintended consequences: No	Setting: Healthcare centers providing prenatal, infant, and other primary care services in Porto Alegre<br><br>Serve priority groups: Yes<br><br>Analytic sample: 20 healthcare centers<br><br>Interventionist: Physicians, nurses, and administrative staff	Length of intervention: 6 years<br><br>Policy:<br><br>System: Training and provision of a pocket guide for physicians, nurses, and administrative staff Incorporation of strategies to support Ten Steps to Successful Breastfeeding<br><br>Environmental: Posters added to waiting rooms<br><br>Fidelity: No<br><br>Cost: No	Intervention sustained beyond study: Unsure<br><br>Reported sustainability of impact: Yes
Schroeder, 2015(157)<br>Schroeder, 2012(35)<br>(*Suppl. Ref*)<br><br>*Primary care-based obesity prevention*<br><br>USA	Design: Cluster randomized control trial<br><br>Comparator: Standard of care<br><br>Theory: Not reported	Sample: All healthy newborns with ≥2000 g body weight and who were discharged home within 5 days after birth<br><br>Priority groups: Low income, race and ethnicity	Outcomes:<br>Feeding practices: +*<br>Child diet: +*<br>Child weight: θ<br><br>Subgroup analysis: Urban-suburban	Setting: Health centers from the Johns Hopkins Community Physicians network in Maryland<br><br>Serve priority groups: Unsure	Length of intervention: 5 years<br><br>Policy:<br><br>System: Incorporation of educational discussions between patient and pediatrician at visits	Intervention sustained beyond study: Unsure<br><br>Reported sustainability of impact: No

Supplementary Data

Study Identifiers	Study Elements	Reach	Effectiveness	Adoption	Implementation	Maintenance
		Analytic sample: 232 infants<br><br>Attrition: 11%	Reported unintended consequences: No	Analytic sample: 4 health centers<br><br>Interventionist: Pediatricians, nurse practitioners, and clinic staff	Environmental:<br><br>Fidelity: Yes<br><br>Cost: No	
Scott, 2015(105)<br><br>*Enhanced lactation support in a clinic setting*<br><br>USA	Design: Retrospective cohort<br><br>Comparator: Standard of care<br><br>Theory: Not reported	Sample: All breastfeeding mother–baby dyads seen at an ambulatory care pediatric clinic by the 7th day of age who spoke English or Spanish<br><br>Priority groups: Ethnicity, low education<br><br>Analytic sample: 35 mother-infant dyads<br><br>Attrition: 19%	Outcomes:<br>Breastfeeding: -<br><br>Subgroup analysis: No<br><br>Reported unintended consequences: No	Setting: Ambulatory care pediatric clinic<br><br>Serve priority groups: Unsure<br><br>Analytic sample: 1 clinic<br><br>Interventionist: Advanced practicing nurse, pediatric nurse practitioner, lactation consultant	Length of intervention: 2 months<br><br>Policy:<br><br>System: Enhanced well-baby visits with phone call support from pediatric nurse practitioner/International Board Certified Lactation Consultant<br><br>Environmental:<br><br>Fidelity: No<br><br>Cost: No	Intervention sustained beyond study: Unsure<br><br>Reported sustainability of impact: No
Taveras, 2011(178)<br><br>*First steps for mommy and me*<br><br>USA	Design: Non-randomized control trial<br><br>Comparator: Standard of care<br><br>Theory: Not reported	Sample: All infants aged 0–1 month old with a mother who could respond to interviews and questionnaires in English<br><br>Priority groups: No<br><br>Analytic sample: 80 mother-infant dyads<br><br>Attrition: 5%	Outcomes:<br>Breastfeeding: θ<br>Complementary feeding: +*<br>Child weight: +<br>Maternal diet: θ<br><br>Subgroup analysis: No<br><br>Reported unintended consequences: No	Setting: Pediatric primary care practices in the Boston, Massachusetts area part of Harvard Vanguard Medical Associates<br><br>Serve priority groups: Yes<br><br>Analytic sample: 3 practices<br><br>Interventionist: Pediatric providers and health educator	Length of intervention: 6 months<br><br>Policy:<br><br>System: Incorporation of brief negotiation to well child care visits and addition of monthly parenting skills training sessions<br><br>Environmental: Monthly parenting sessions promote peer support and allow for social networking<br><br>Fidelity: No<br><br>Cost: No	Intervention sustained beyond study: Unsure<br><br>Reported sustainability of impact: No
Virtanen (NCT), 2010(36)(*Suppl. Ref*)	Design: Randomized control trial, pilot study	Sample: All families who speak Finnish at their child's standard 6-	Outcomes:<br>Ongoing study<br>(child diet, maternal diet)	Setting: Child welfare health clinics	Length of intervention: 8 months	Intervention sustained beyond study: Unsure

Supplementary Data

Study Identifiers	Study Elements	Reach	Effectiveness	Adoption	Implementation	Maintenance
Lifestyle Intervention for Toddlers<br><br>Finland	Comparator: Standard of care<br><br>Theory: Not reported	month child welfare clinic visit<br><br>Priority groups: Unsure<br><br>Analytic sample: Not reported<br><br>Attrition: n/a	Subgroup analysis: No<br><br>Reported unintended consequences: No	Serve priority groups: Unsure<br><br>Analytic sample: Not reported<br><br>Interventionist: Public health nurses	Policy:<br><br>System: Intensified dietary counseling at child wellfare clinics<br><br>Environmental:<br><br>Fidelity: No<br><br>Cost: No	Reported sustainability of impact: No
Vlasblom, 2020(135) Eline, 2016(37)(*Suppl. Ref*)<br><br>*Parenting support to prevent overweight during regular well-child visits (BBOFT plus program)*<br><br>Netherlands	Design: Cluster randomized control trial<br><br>Comparator: Standard of care<br><br>Theory: Social Learning Theory	Sample: Parents with a child born between January 2009 and September 2010 who could read Dutch<br><br>Priority groups: Unsure<br><br>Analytic sample: 2,318 parents<br><br>Attrition: 23%	Outcomes:<br>Breastfeeding: +*<br>Feeding style: -*<br>Child diet: +*<br>Child weight: θ<br><br>Subgroup analysis: No<br><br>Reported unintended consequences: No	Setting: Youth Health Care services<br><br>Serve priority groups: Unsure<br><br>Analytic sample: 10 Youth Health Care services<br>51 Youth Health Care clinic teams<br><br>Interventionist: Community physicians and nurses	Length of intervention: 3 years<br><br>Policy:<br><br>System: Addition of education and guidance program during well-child visits<br><br>Environmental:<br><br>Fidelity: No<br><br>Cost: No	Intervention sustained beyond study: Unsure<br><br>Reported sustainability of impact: No
Wang, 2019(151)<br><br>*Different growth standards*<br><br>China	Design: Cluster randomized control trial<br><br>Comparator: Standard of care<br><br>Theory: Not reported	Sample: Single fetus with gestational age 37 - 42 weeks without congenital or genetic metabolic diseases, birth weight 2500 - 4500 g, willing to come for long-term follow up<br><br>Priority groups: Unsure<br><br>Analytic sample: 15,019 infants<br><br>Attrition: 0%	Outcomes:<br>Child weight: +*<br><br>Subgroup analysis: No<br><br>Reported unintended consequences: No	Setting: Community health service centers in Shanghai<br><br>Serve priority groups: Unsure<br><br>Analytic sample: 19 community health service centers<br><br>Interventionist: Pediatricians, nurse practitioners	Length of intervention: 12 months<br><br>Policy:<br><br>System: Application of different growth standards to generate a growth curve for every child and provide nutritional advice to caregivers based on growth tendency<br><br>Environmental:<br><br>Fidelity: No<br><br>Cost: No	Intervention sustained beyond study: Unsure<br><br>Reported sustainability of impact: No
Witt, 2012(94)<br><br>*Integrating routine lactation consultant*	Design: Repeated cross-sectional	Sample: Charts of consecutive newborns to the practice before and	Outcomes:<br>Breastfeeding: +*<br><br>Subgroup analysis: No	Setting: Suburban pediatric practice in Cleveland, Ohio	Length of intervention:<br>Policy: n/a	Intervention sustained beyond study: Yes

Supplementary Data

Study Identifiers	Study Elements	Reach	Effectiveness	Adoption	Implementation	Maintenance
support into a pediatric practice<br><br>USA	Comparator: Historical control<br><br>Theory: Not reported	after program implementation<br><br>Priority groups: No<br><br>Analytic sample: 350 patients<br><br>Attrition: 0%	Reported unintended consequences: No	Serve priority groups: No<br><br>Analytic sample: 1 pediatric practice<br><br>Interventionist: International Board-Certified Lactation Consultant and physician	Policy: Policy to see all healthy term breastfeeding infants in office by 3-5 days of life<br><br>System: Protocol that healthy term breastfeeding infants seen in office by 3-5 days of life by lactation consultant and physician. Change staff position to ensure regular coverage by lactation consultant<br><br>Environmental:<br><br>Fidelity: No<br><br>Cost: No	Reported sustainability of impact: No
Witt, 2021(107)<br><br>*Team-based breastfeeding support*<br><br>USA	Design: Repeated cross-sectional<br><br>Comparator: Historical control<br><br>Theory: Not reported	Sample: Charts of consecutive newborns to the practice before and after program implementation<br><br>Priority groups: Low income, race and ethnicity, immigrants<br><br>Analytic sample: 442 newborns<br><br>Attrition: Unable to calculate	Outcomes:<br>Breastfeeding: Ø<br><br>Subgroup analysis: NICU admission, prenatal care with midwife, lactation consultant support, initiation of breastfeeding<br><br>Reported unintended consequences: No	Setting: Federally Qualified Health Center in Cleveland, Ohio<br><br>Serve priority groups: Yes<br><br>Analytic sample: 1 Federally Qualified Health Center<br><br>Interventionist: Advanced Practice Registered Nurse/ International Board-Certified Lactation Consultant<br>and Registered Nurse/Certified Lactation Consultant	Length of intervention: 40 minutes<br><br>Policy:<br><br>System: Implementation of routine lactation consultant and primary care physician visits for families at first newborn visit<br><br>Environmental:<br><br>Fidelity: Yes<br><br>Cost: No	Intervention sustained beyond study: Yes<br><br>Reported sustainability of impact: No
**Prenatal care (n=44)**						
Alayli, 2020(75)<br><br>*Computer-assisted multiprofessional intervention to address lifestyle-related risk factors*	Design: Cluster randomized hybrid effectiveness-implementation trial (Type II)	Sample: Pregnant women <12 weeks' gestation proficient in German who have health insurance from a program partner	Outcomes:<br>Ongoing study (breastfeeding, feeding practices, child diet, child weight, maternal diet, maternal weight)	Setting: Urban and rural areas within Baden-Wuerttemberg<br><br>Serve priority groups: Yes	Length of intervention: 18 months<br><br>Policy:<br><br>System: Incorporation of lifestyle intervention into	Intervention sustained beyond study: Unsure<br><br>Reported sustainability of impact: No

Supplementary Data

Study Identifiers	Study Elements	Reach	Effectiveness	Adoption	Implementation	Maintenance
Germany	Comparator: Standard of care<br><br>Theory: RE-AIM	Priority groups: Unsure<br><br>Analytic sample: Goal: 1,240 mother-infant dyads<br><br>Attrition: n/a	Subgroup analysis: No<br><br>Reported unintended consequences: No	Analytic sample: 8 regions<br><br>Interventionist: Gynecologists, midwives, and pediatricians	routine prenatal visits and infant check-ups. Facilitate cooperation between gynecologists, pediatricians, and midwives through telehealth platform.<br><br>Environmental: Development of telehealth platform<br><br>Fidelity: Yes<br><br>Cost: Yes	
Alberdi, 2018(115) O'Sullivan, 2018(38)(*Suppl. Ref*)<br><br>*Multidimensional breastfeeding support intervention*<br><br>Ireland	Design: Single group, pre-post design, feasibility study<br><br>Comparator: No control<br><br>Theory: Not reported	Sample: Primiparous women between 32–38 weeks' gestation with singleton pregnancies and easy access to the internet<br><br>Priority groups: Rural<br><br>Analytic sample: 60 women<br><br>Attrition: 53%	Outcomes: Breastfeeding: +<br><br>Subgroup analysis: Urban-rural<br><br>Reported unintended consequences: No	Setting: National Maternity Hospital in Dublin and Wexford General Hospital in Wexford<br><br>Serve priority groups: Yes<br><br>Analytic sample: 2 hospitals<br><br>Interventionist: International Board-Certified Lactation Consultants	Length of intervention: 7 months<br><br>Policy:<br><br>System: Enhanced standard of care through antenatal classes and lactation specialist consultation<br><br>Environmental: Direct helpline and clinic opportunities with lactation consultant Compulsory support person attendance at a class<br><br>Fidelity: Yes<br><br>Cost: No	Intervention sustained beyond study: Unsure<br><br>Reported sustainability of impact: No
Ardalan, 2009(85)<br><br>*Comprehensive Perinatal Services Program*<br><br>USA	Design: Retrospective cohort<br><br>Comparator: No control<br><br>Theory: Not reported	Sample: Women with singleton pregnancy and BMI ≥ 25.0 kg/m^2<br><br>Priority groups: Low income, overweight or obesity<br><br>Analytic sample: 101 prenatal charts<br><br>Attrition: n/a	Outcomes: Maternal weight: θ<br><br>Subgroup analysis: No<br><br>Reported unintended consequences: No	Setting: Harbor-UCLA Faculty Perinatal Practice<br><br>Serve priority groups: Unsure<br><br>Analytic sample: 1 practice<br><br>Interventionist: Not reported	Length of intervention: Not reported<br><br>Policy:<br><br>System: New system to evaluate, educate and assess pregnant patients in nutrition, health education and psychology; nutritionist	Intervention sustained beyond study: Unsure<br><br>Reported sustainability of impact: No

Supplementary Data

Study Identifiers	Study Elements	Reach	Effectiveness	Adoption	Implementation	Maintenance
					referral based upon screening weight<br><br><u>Environmental</u>:<br><br><u>Fidelity</u>: Yes<br><br><u>Cost</u>: No	
Bennett, 2022(39)<br><br>*Healthy for Two/Healthy for You*<br><br>USA	<u>Design</u>: Randomized control trial<br><br><u>Comparator</u>: Standard of care<br><br><u>Theory</u>: Not reported	<u>Sample</u>: Women who speak English, currently have a singleton pregnancy ≤15 weeks' gestation with any parity status and BMI ≥ 25.0 kg/m^2<br><br><u>Priority groups</u>: Low income, race, overweight or obesity<br><br><u>Analytic sample</u>: Goal: 304 mother-infant dyads<br><br><u>Attrition</u>: n/a	<u>Outcomes</u>: Ongoing study (breastfeeding, child weight, maternal diet, maternal weight)<br><br><u>Subgroup analysis</u>: No<br><br><u>Reported unintended consequences</u>: No	<u>Setting</u>: Academic and community-based prenatal care clinics in and around Baltimore, Maryland<br><br><u>Serve priority groups</u>: Yes<br><br><u>Analytic sample</u>: 6 clinics<br><br><u>Interventionist</u>: Nurses, medical assistants, dietitians, midwives	<u>Length of intervention</u>: 10 months<br><br><u>Policy</u>:<br><br><u>System</u>: Coaching program integrated into prenatal care. Electronic health record interface that enables direct referrals and progress reports for providers<br><br><u>Environmental</u>:<br><br><u>Fidelity</u>: Yes<br><br><u>Cost</u>: Yes	<u>Intervention sustained beyond study</u>: Unsure<br><br><u>Reported sustainability of impact</u>: No
Bonuck, 2014(118)<br>Bonuck (NCT), 2008(40) (*Suppl. Ref*)<br>Bonuck (NCT), 2008(41) (*Suppl. Ref*)<br><br>*Primary care intervention on breastfeeding (BINGO and PAIRINGS)*<br><br>USA	<u>Design</u>: Randomized control trial<br><br><u>Comparator</u>: Standard of care<br><br><u>Theory</u>: Not reported	<u>Sample</u>: English- or Spanish-speaking women in the first or second trimester of a singleton pregnancy, without risk factors for premature birth, or contraindications of breastfeeding<br><br><u>Priority groups</u>: Low income, race and ethnicity<br><br><u>Analytic sample</u>:<br>BINGO: 628 women<br>PAIRINGS: 262 women<br><br><u>Attrition</u>:<br>BINGO: 6%<br>PAIRINGS: 5%	<u>Outcomes</u>: Breastfeeding: +*<br><br><u>Subgroup analysis</u>: Breastfeeding intensity<br><br><u>Reported unintended consequences</u>: No	<u>Setting</u>: Urban medical center and affiliated prenatal care clinics in the Bronx, New York<br><br><u>Serve priority groups</u>: Yes<br><br><u>Analytic sample</u>: Not reported<br><br><u>Interventionist</u>: BINGO: Resident and attending obstetrician or gynecologists and certified nurse-midwives.<br>PAIRINGS: obstetrician or gynecologist faculty<br>Lactation consultants	<u>Length of intervention</u>: 9 months<br><br><u>Policy</u>:<br><br><u>System</u>: Electronic medical record prompts<br>Routine presence of and contact with lactation consultant<br><br><u>Environmental</u>: Routine presence of lactation consultants at prenatal sites and hospitals<br>Provision of nursing bras and breast pumps<br><br><u>Fidelity</u>: Yes<br><br><u>Cost</u>: No	<u>Intervention sustained beyond study</u>: Unsure<br><br><u>Reported sustainability of impact</u>: No

Supplementary Data

Study Identifiers	Study Elements	Reach	Effectiveness	Adoption	Implementation	Maintenance
Brownfoot, 2016(99)<br>Brownfoot, 2015(42)<br>(*Suppl. Ref*)<br><br>*Weighing in Pregnancy*<br><br>Australia	Design: Randomized control trial<br><br>Comparator: Standard of care<br><br>Theory: Not reported	Sample: Women < 21 weeks' gestation with singleton pregnancy<br><br>Priority groups: Unsure<br><br>Analytic sample: 741 women<br><br>Attrition: 5%	Outcomes:<br>Maternal weight: θ<br>Breastfeeding: θ<br><br>Subgroup analysis: BMI categories, age, parity, number of times weighed<br><br>Reported unintended consequences: No	Setting: Antenatal clinics tertiary obstetric hospital in Melbourne<br><br>Serve priority groups: Unsure<br><br>Analytic sample: 1 antenatal clinic in tertiary obstetric hospital<br><br>Interventionist: Clinicians	Length of intervention: 6 months<br><br>Policy:<br><br>System: Adding weight assessment at antenatal visits and discussion about weight gain<br><br>Environmental:<br><br>Fidelity: Yes<br><br>Cost: No	Intervention sustained beyond study: Unsure<br><br>Reported sustainability of impact: No
Brumley, 2016(182)<br><br>*CenteringPregnancy®*<br><br>USA	Design: Retrospective cohort study<br><br>Comparator: Standard of care<br><br>Theory: Not reported	Sample: All women eligible for midwifery care through 20 weeks' gestation<br><br>Priority groups: Race and ethnicity<br><br>Analytic sample: 195 women<br><br>Attrition: n/a	Outcomes:<br>Breastfeeding: +*<br>Maternal weight: θ<br>Child weight: θ<br><br>Subgroup analysis: No<br><br>Reported unintended consequences: No	Setting: Academic practice in southeast<br><br>Serve priority groups: Unsure<br><br>Analytic sample: 1 academic practice<br><br>Interventionist: Not reported	Length of intervention: 6 months<br><br>Policy:<br><br>System: Change from individual to group model of prenatal care<br><br>Environmental: Group support<br><br>Fidelity: No<br><br>Cost: No	Intervention sustained beyond study: Unsure<br><br>Reported sustainability of impact: No
Chae, 2017(163)<br><br>*CenteringPregnancy®*<br><br>USA	Design: Cohort<br><br>Comparator: Standard of care<br><br>Theory: Not reported	Sample: Women with gestational age ≤ 24 weeks who speak and read English and able to attend groups<br><br>Priority groups: Low income, race and ethnicity, immigrants<br><br>Analytic sample: 341 women<br><br>Attrition: 21%	Outcomes:<br>Breastfeeding: +*<br><br>Subgroup analysis: No<br><br>Reported unintended consequences: No	Setting: Residency-based Family Medicine outpatient site<br><br>Serve priority groups: Yes<br><br>Analytic sample: 1 residency-based Family Medicine outpatient site<br><br>Interventionist: Attending family medicine physician, faculty psychologist and family medicine resident physician	Length of intervention: 28 weeks<br><br>Policy:<br><br>System: Change from individual to group model of prenatal care<br><br>Environmental: Peer support<br><br>Fidelity: No<br><br>Cost: No	Intervention sustained beyond study: Unsure<br><br>Reported sustainability of impact: No
Chen, 2017(115)	Design: Randomized control trial	Sample: Women aged 14–45 years who enter	Outcomes:<br>Ongoing study	Setting: Greenville Health System Obstetrics	Length of intervention: 6 months	Intervention sustained beyond study: Unsure

Supplementary Data

Study Identifiers	Study Elements	Reach	Effectiveness	Adoption	Implementation	Maintenance
CenteringPregnancy®<br><br>USA	Comparator: Standard of care<br><br>Theory: Not reported	prenatal care before their 20th week of gestation, low risk pregnancy<br><br>Priority groups: Race and ethnicity<br><br>Analytic sample: Not reported<br><br>Attrition: n/a	(breastfeeding, food insecurity, maternal diet, maternal weight, child weight)<br><br>Subgroup analysis: Self-report race and ethnicity; socially assigned race and ethnicity<br><br>Reported unintended consequences: Yes	Center and ultrasound unit in Greenville, South Carolina<br><br>Serve priority groups: Yes<br><br>Analytic sample: 1 primary care clinic<br><br>Interventionist: Nurse practitioner, nurse midwife, or physician and a co-facilitator	Policy:<br><br>System: Change from individual to group model of prenatal care<br><br>Environmental: Group sessions for socialization and to build relationships<br><br>Fidelity: Yes<br><br>Cost: No	Reported sustainability of impact: No
Clements, 2016(43) (*Suppl. Ref*)<br><br>*Get Healthy in Pregnancy*<br><br>Australia	Design: Cluster randomized control trial<br><br>Comparator: Minimal intervention<br><br>Theory: Not reported	Sample: Women who have a singleton pregnancy gestation of ≤ 18 weeks and speak English.<br><br>Priority groups: Overweight or obesity<br><br>Analytic sample: n/a<br><br>Attrition: n/a	Outcomes: Ongoing study (maternal diet, maternal weight)<br><br>Subgroup analysis: No<br><br>Reported unintended consequences: No	Setting: Antenatal clinics of hospitals in New South Wales<br><br>Serve priority groups: Unsure<br><br>Analytic sample: 5 antenatal clinics<br><br>Interventionist: Dietitians and exercise physiologists (health coaches)	Length of intervention: 9 months<br><br>Policy:<br><br>System: Integrating telephone-based health coaching with standard maternity care and setting weight-gain range target at first antenatal visit with midwife<br><br>Environmental:<br><br>Fidelity: No<br><br>Cost: No	Intervention sustained beyond study: Unsure<br><br>Reported sustainability of impact: Yes
Daley, 2015(100)<br>Daley, 2014(44) (*Suppl. Ref*)<br><br>*Regular weighing in pregnancy*<br><br>United Kingdom	Design: Randomized control trial<br><br>Comparator: Standard of care<br><br>Theory: Not reported	Sample: Low risk pregnant women receiving community midwife led care within BMI ranges 18–29.9 kg/m^2 at their first antenatal appointment<br><br>Priority groups: Highest quartiles of multiple deprivation index, overweight<br><br>Analytic sample: 68 women<br><br>Attrition: 11%	Outcomes: Maternal weight: +<br><br>Subgroup analysis: No<br><br>Reported unintended consequences: Yes	Setting: Maternity center<br><br>Serve priority groups: Unsure<br><br>Analytic sample: 1 maternity center<br><br>Interventionist: Community midwife	Length of intervention: 6 months<br><br>Policy:<br><br>System: Addition of routine weighing and documentation to antenatal visits<br><br>Environmental:<br><br>Fidelity: Yes<br><br>Cost: No	Intervention sustained beyond study: Unsure<br><br>Reported sustainability of impact: Yes

Supplementary Data

Study Identifiers	Study Elements	Reach	Effectiveness	Adoption	Implementation	Maintenance
de Jersey, 2022(89)<br><br>*Changes to routine antenatal care*<br><br>Australia	Design: Cohort<br><br>Comparator: No control<br><br>Theory: PRECEDE PROCEED	Sample: Women <20 weeks' gestation with English language skills<br><br>Priority groups: Low income<br><br>Analytic sample: 693 women<br><br>Attrition: 42%	Outcomes:<br>Maternal weight: θ<br><br>Subgroup analysis: No<br><br>Reported unintended consequences: No	Setting: Metropolitan birthing facility<br><br>Serve priority groups: Yes<br><br>Analytic sample: 1 birthing facility<br><br>Interventionist: Midwives and dietetic lead	Length of intervention: 6 months<br><br>Policy:<br><br>System: Added pregnancy weight gain charts and brief intervention advice framework during routine consultations; training for health care providers<br><br>Environmental:<br><br>Fidelity: Yes<br><br>Cost: No	Intervention sustained beyond study: Unsure<br><br>Reported sustainability of impact: No
Garmendia, 2020(145)<br>Garmendia, 2015(45) (*Suppl. Ref*)<br>Garmendia (NCT), 2013(46) (*Suppl. Ref*)<br><br>*Chilean maternal and infant cohort study (ChiMINCs)*<br><br>Chile	Design: Cluster randomized control trial<br><br>Comparator: Standard of care<br><br>Theory: Not reported	Sample: Women <15 weeks' gestation<br><br>Priority groups: Overweight or obesity<br><br>Analytic sample: 4,631 women<br>3763 infants<br><br>Attrition: n/a	Outcomes:<br>Child weight: θ<br>Maternal weight: +*<br><br>Subgroup analysis: Excluding those diagnosed with gestational diabetes and women for whom weight was obtained at delivery<br><br>Reported unintended consequences: Yes	Setting: Primary health care centers from two of the largest urban counties of the Southeast Health Area of Santiago<br><br>Serve priority groups: Unsure<br><br>Analytic sample: 12 primary health care centers<br><br>Interventionist: Midwives, dietitians, physical activity trainers	Length of intervention: 6 months<br><br>Policy:<br><br>System: Training on weight monitoring and delivery of messages for health professionals; installation of a computer-assisted system for maternal weight monitoring; incorporation of weight gain assessment to visits; referral to dietitian; physical activity program<br><br>Environmental:<br><br>Fidelity: Yes<br><br>Cost: No	Intervention sustained beyond study: Unsure<br><br>Reported sustainability of impact: No
Gomes, 2019(87)<br><br>*Educational intervention with health professionals*<br><br>Brazil	Design: Non-randomized control trial<br><br>Comparator: Standard of care	Sample: Pregnant women < 14 weeks' gestation<br><br>Priority groups: Low income, overweight or obesity, low education	Outcomes:<br>Maternal diet: +*<br><br>Subgroup analysis: No<br><br>Reported unintended consequences: No	Setting: Prenatal care clinics in public primary health units in Botucatu<br><br>Serve priority groups: Yes	Length of intervention: 6 months<br><br>Policy:<br><br>System: Training for health professionals to	Intervention sustained beyond study: Unsure<br><br>Reported sustainability of impact: No

Supplementary Data

Study Identifiers	Study Elements	Reach	Effectiveness	Adoption	Implementation	Maintenance
	Theory: Transtheoretical Model, Motivational Interviewing	Analytic sample: 267 pregnant women<br><br>Attrition: 26%		Analytic sample: 15 clinics<br><br>Interventionist: General physicians, nurses, nursing technicians, and community health agents	incorporate guidance into prenatal care appointments<br><br>Environmental:<br><br>Fidelity: No<br><br>Cost: No	
Graça, 2011(180)<br><br>*Nursing intervention in primary healthcare*<br><br>Portugal	Design: Non-randomized control trial<br><br>Comparator: Standard of care<br><br>Theory: Not reported	Sample: Primipara women between 18 and 38 years of age<br><br>Priority groups: Unsure<br><br>Analytic sample: 151 primiparae<br><br>Attrition: 11%	Outcomes:<br>Breastfeeding: +<br><br>Subgroup analysis:No<br><br>Reported unintended consequences: Not reported	Setting: Health Centers in the district of Viana do Castelo<br><br>Serve priority groups: Unsure<br><br>Analytic sample: Not reported<br><br>Interventionist: Specialist nurses in maternal health and obstetrics	Length of intervention: 14 weeks<br><br>Policy:<br><br>System: Incorporation of consult and education at prepartum visits and either a support group or postpartum visit<br><br>Environmental: Peer support<br><br>Fidelity: No<br><br>Cost: No	Intervention sustained beyond study: Unsure<br><br>Reported sustainability of impact: No
Graziano (NCT), 2013(47) (*Suppl. Ref*)<br><br>*Gestational Weight Gain and the Electronic Medical Record (WATE)*<br><br>USA	Design: Randomized control trial<br><br>Comparator: Standard of care<br><br>Theory: Not reported	Sample: Women <14 weeks' gestation with a singleton<br><br>Priority groups: Unsure<br><br>Analytic sample: Not reported<br><br>Attrition: n/a	Outcomes:<br>Ongoing study (maternal weight)<br><br>Subgroup analysis: No<br><br>Reported unintended consequences: No	Setting: Prenatal care system<br><br>Serve priority groups: Unsure<br><br>Analytic sample: Not reported<br><br>Interventionist: Providers	Length of intervention: Not reported<br><br>Policy:<br><br>System: Alerts added to electronic medical reporting system to remind providers to counsel patients on recommendations for gestational weight gain<br><br>Environmental:<br><br>Fidelity: No<br><br>Cost: No	Intervention sustained beyond study: Unsure<br><br>Reported sustainability of impact: No
Gregory, 2016(109)<br><br>*Gestational weight management intervention*	Design: Retrospective cohort<br><br>Comparator: Historical control	Sample: Women with Medicaid insurance who have a pre-pregnancy BMI of 30 kg/m^2 or greater	Outcomes:<br>Child weight: θ<br>Maternal weight: +	Setting: Nutrition in Pregnancy clinic at Johns Hopkins Hospital, Baltimore, Maryland	Length of intervention: 6 months<br><br>Policy:	Intervention sustained beyond study: Unsure<br><br>Reported sustainability of impact: Yes

Supplementary Data

Study Identifiers	Study Elements	Reach	Effectiveness	Adoption	Implementation	Maintenance
USA	Theory: Not reported	Priority groups: Low income, race, obesity<br><br>Analytic sample: 154 infants<br><br>Attrition: 25%	Subgroup analysis: Number of visits and gestational weight gain or infant weight status at 1 year<br><br>Reported unintended consequences: No	Serve priority groups: Yes<br><br>Analytic sample: 1 obstetrical clinic<br><br>Interventionist: Obstetrician, nurse practitioner, nutritionist, and social worker, obstetrical residents	System: Integration of nutritional care with obstetrical care<br><br>Environmental: Creation of Nutrition in Pregnancy clinic<br><br>Fidelity: No<br><br>Cost: No	
Haby, 2015(124)<br><br>*Mighty Mums*<br><br>Sweden	Design: Non-randomized control trial, pilot study<br><br>Comparator: Standard of care<br><br>Theory: Not reported	Sample: BMI 30 – 40 kg/m^2 at the first antenatal health care visit<br><br>Priority groups: Obesity<br><br>Analytic sample: 100 women<br><br>Attrition: 67%	Outcomes:<br>Maternal weight: +*<br><br>Subgroup analysis: Use of interpreter<br><br>Reported unintended consequences: No	Setting: Antenatal Health Clinic in Göteborg<br><br>Serve priority groups: Unsure<br><br>Analytic sample: Not reported<br><br>Interventionist: Midwives	Length of intervention: 6 months<br><br>Policy:<br><br>System: Additional time with midwife and lifestyle follow-up during regular appointments; routine checking of weight Network formed with doulas, health care providers, and surrounding community<br><br>Environmental: Price reductions at municipal sports and recreation facilities<br><br>Fidelity: Yes<br><br>Cost: No	Intervention sustained beyond study: Yes<br><br>Reported sustainability of impact: Yes
Heberlein, 2016(130)<br><br>*CenteringPregnancy®*<br><br>USA	Design: Prospective cohort<br><br>Comparator: Standard of care<br><br>Theory: Not reported	Sample: Medically low risk pregnant women entering care before 17 weeks' gestation who speak English<br><br>Priority groups: Low income, race<br><br>Analytic sample: 209 mothers<br><br>Attrition: 16%	Outcomes:<br>Food security: +*<br><br>Subgroup analysis: Parity, dose of intervention<br><br>Reported unintended consequences: No	Setting: Large outpatient hospital-affiliated obstetrics practice in the Southeast<br><br>Serve priority groups: Yes<br><br>Analytic sample: 1 obstetrics practice<br><br>Interventionist: Not reported	Length of intervention: 8 months<br><br>Policy:<br><br>System: Change from individual to group model of prenatal care<br><br>Environmental: Group sessions for socialization and to build relationships<br><br>Fidelity: Yes	Intervention sustained beyond study: Yes<br><br>Reported sustainability of impact: No

Supplementary Data

**Study Identifiers**	**Study Elements**	**Reach**	**Effectiveness**	**Adoption**	**Implementation**	**Maintenance**
					Cost: No	
Hoch, 2023(90)<br><br>*Enhanced implementation of routine antenatal care practices*<br><br>Location not reported	Design: Repeated cross-sectional<br><br>Comparator: Historical control<br><br>Theory: PRECEDE-PROCEED	Sample: Not reported<br><br>Priority groups: Unsure<br><br>Analytic sample: 771 women<br><br>Attrition: 35%	Outcomes:<br>Maternal weight: +*<br><br>Subgroup analysis: Number of weights plotted (>3)<br><br>Reported unintended consequences: No	Setting: Large metropolitan birthing facility<br><br>Serve priority groups: Unsure<br><br>Analytic sample: 1 birthing facility<br><br>Interventionist: Antenatal staff	Length of intervention: 9 months<br><br>Policy: Mandatory training about expectations and documentation of weight-related care<br><br>System: Incorporation of routine weighing to all antenatal appointments and use of pregnancy weight gain charts<br><br>Environmental: Provision of scales<br><br>Fidelity: Yes<br><br>Cost: No	Intervention sustained beyond study: Yes<br><br>Reported sustainability of impact: Yes
Hoffman, 2021(148)<br>Kunath, 2019(48) (*Suppl. Ref*)<br>Hoffmann, 2019(49) (*Suppl. Ref*)<br>Rauh, 2014(50) (*Suppl. Ref*)<br>Hoffman, 2020(51) (*Suppl. Ref*)<br>Hauner (NCT), 2013(52) (*Suppl. Ref*)<br>Rauh, 2013(53) (*Suppl. Ref*)<br><br>*Healthy Living in Pregnancy (GeliS)*<br><br>Germany	Design: Cluster randomized control trial<br><br>Comparator: Standard of care<br><br>Theory: Not reported	Sample: Women with a singleton pregnancy <13 weeks' gestation with pre-pregnancy BMI 18.5 - 40.0 kg/m^2 and German language skills<br><br>Priority groups: Unsure<br><br>Analytic sample: 1,998 mother-infant dyads<br><br>Attrition: 13%	Outcomes:<br>Breastfeeding: +*<br>Formula feeding: θ<br>Complementary feeding: +*<br>Child weight: θ<br>Maternal weight: θ<br><br>Subgroup analysis: Maternal pre-pregnancy BMI<br><br>Reported unintended consequences: No	Setting: Primary care practices in administrative regions of Bavaria<br><br>Serve priority groups: Unsure<br><br>Analytic sample: 71 practices<br><br>Interventionist: Midwives, medical personnel, or gynaecologists	Length of intervention: 9 months<br><br>Policy:<br><br>System: Incorporation of counseling sessions into routine care visits<br><br>Environmental:<br><br>Fidelity: Yes<br><br>Cost: No	Intervention sustained beyond study: Unsure<br><br>Reported sustainability of impact: Yes
Ickovics, 2016(150)<br>Magriples, 2015(54) (*Suppl. Ref*)<br><br>*CenteringPregnancy®*<br><br>USA	Design: Cluster randomized control trial<br><br>Comparator: Delayed<br><br>Theory: Not reported	Sample: Adolescents aged 14 - 21 years at < 24 weeks' gestation with low-risk pregnancy who spoke English or Spanish and willing to participate in group care	Outcomes:<br>Maternal diet: Not reported<br>Maternal weight: +*<br>Breastfeeding: θ<br>Child weight: +	Setting: Community health centers and hospitals in New York City<br><br>Serve priority groups: Yes	Length of intervention: 6 months<br><br>Policy:<br><br>System: Change from individual to group model of prenatal care	Intervention sustained beyond study: No<br><br>Reported sustainability of impact: No

Supplementary Data

Study Identifiers	Study Elements	Reach	Effectiveness	Adoption	Implementation	Maintenance
		Priority groups: Low income, race and ethnicity, people with overweight or obesity, adolescent mothers<br><br>Analytic sample: 1,148 women<br><br>Attrition: 7%	Subgroup analysis: Minimal dose of intervention, pre-pregnancy BMI group<br><br>Reported unintended consequences: No	Analytic sample: 13 clinical sites<br><br>Interventionist: Obstetrician or midwife and nurse or medical assistant	and training for clinical and administrative staff<br><br>Environmental: Peer support<br><br>Fidelity: Yes<br><br>Cost: No	
Kettrey, 2020(136)<br><br>*CenteringPregnancy®*<br><br>USA	Design: Non-randomized control trial, pilot study<br><br>Comparator: Standard of care<br><br>Theory: Not reported	Sample: Prenatal care patients over age 18<br><br>Priority groups: Low income, ethnicity, rural<br><br>Analytic sample: 1,524 records<br><br>Attrition: 22%	Outcomes:<br>Breastfeeding: +*<br>Child weight: -*<br><br>Subgroup analysis: Dose-response<br><br>Reported unintended consequences: No	Setting: Diverse prenatal care sites across the state of Tennessee<br><br>Serve priority groups: Yes<br><br>Analytic sample: 6 prenatal care sites<br><br>Interventionist: Licensed obstetric provider	Length of intervention: 28 weeks<br><br>Policy:<br><br>System: Change from individual to group model of prenatal care<br><br>Environmental: Peer support<br><br>Fidelity: No<br><br>Cost: No	Intervention sustained beyond study: Unsure<br><br>Reported sustainability of impact: No
Kingsland, 2021(76)<br><br>*Implementation intervention for routine provision of antenatal care addressing gestational weight gain*<br><br>Australia	Design: Stepped wedge<br><br>Comparator: Standard of care<br><br>Theory: Theoretical Domains Framework, Capacity, Opportunity, Motivation- Behaviours (COM-B) model, Behaviour Change Wheel behaviour change techniques	Sample: Pregnant at > 12 weeks' gestation and < 37 weeks' gestation with English language proficiency and mentally and physically capable of completing the survey<br><br>Priority groups: Aboriginal people<br><br>Analytic sample: Not reported<br><br>Attrition: n/a	Outcomes: Ongoing study (maternal diet, maternal weight)<br><br>Subgroup analysis: No<br><br>Reported unintended consequences: No	Setting: Maternity services in three health sectors within the Hunter New England Local Health District, New South Wales<br><br>Serve priority groups: Yes<br><br>Analytic sample: Not reported<br><br>Interventionist: Midwives, staff specialists in obstetrics, fellows, registrars, resident medical officers, general practice obstetricians, Aboriginal Health Practitioners, Aboriginal Health Workers, and students	Length of intervention: 4 months<br><br>Policy:<br><br>System: Implementing best practice care pathway, opportunity to be weighed at each visit, referral to services for additional support, including free statewide, government-funded health coaching service<br><br>Environmental:<br><br>Fidelity: Yes<br><br>Cost: Yes	Intervention sustained beyond study: Unsure<br><br>Reported sustainability of impact: Yes

Supplementary Data

Study Identifiers	Study Elements	Reach	Effectiveness	Adoption	Implementation	Maintenance
Klima, 2009(181)<br><br>*CenteringPregnancy®*<br><br>USA	Design: Retrospective cohort<br><br>Comparator: Standard of care<br><br>Theory: Not reported	Sample: <18 weeks' gestation upon entry into care and plan to continue care at the site<br><br>Priority groups: Low income, race and ethnicity<br><br>Analytic sample: 268 women<br><br>Attrition: 1%	Outcomes:<br>Breastfeeding: +<br>Child weight: θ<br>Maternal weight: +<br><br>Subgroup analysis: Premature births<br><br>Reported unintended consequences: No	Setting: Urban public health clinic in the Midwest<br><br>Serve priority groups: Yes<br><br>Analytic sample: 1 public health clinic<br><br>Interventionist: Providers and staff	Length of intervention: 6 months<br><br>Policy:<br><br>System: Change from individual to group model of prenatal care<br><br>Environmental: Group sessions for socialization and to build relationships<br><br>Fidelity: No<br><br>Cost: No	Intervention sustained beyond study: Unsure<br><br>Reported sustainability of impact: No
Malta, 2021(160)<br>Malta, 2017(55) (*Suppl. Ref*)<br><br>*Lifestyle intervention in primary health care*<br><br>Brazil	Design: Non-randomized control trial<br><br>Comparator: Standard of care<br><br>Theory: Ecological Model	Sample: All pregnant women in first gestational trimester enrolled in the antenatal public health units in the urban area of Botucatu<br><br>Priority groups: Unsure<br><br>Analytic sample: 267 pregnant women<br><br>Attrition: 24%	Outcomes:<br>Maternal diet: +<br><br>Subgroup analysis: No<br><br>Reported unintended consequences: No	Setting: Antenatal public health units in the urban area of Botucatu, São Paulo State<br><br>Serve priority groups: Unsure<br><br>Analytic sample: 17 public health units<br><br>Interventionist: Physicians and nurses	Length of intervention: 15 months<br><br>Policy:<br><br>System: Provider training and systematic promotion of diet and physical activity during antenatal visits<br><br>Environmental:<br><br>Fidelity: No<br><br>Cost: No	Intervention sustained beyond study: Unsure<br><br>Reported sustainability of impact: No
McGiveron, 2015(110)<br><br>*Screening and referral for enhanced prenatal care*<br><br>United Kingdom	Design: Non-randomized control trial<br><br>Comparator: No intervention<br><br>Theory: Not reported	Sample: All pregnant women attending first dating antenatal ultrasound clinics at Lincoln Hospital with a BMI of ≥35 kg/m^2<br><br>Priority groups: Obesity<br><br>Analytic sample: 178 pregnant women<br><br>Attrition: 8%	Outcomes:<br>Breastfeeding: +*<br>Child weight: θ<br>Maternal weight: +<br><br>Subgroup analysis: No<br><br>Reported unintended consequences: Yes	Setting: Hospital antenatal clinics or local community 'health shops'<br><br>Serve priority groups: Yes<br><br>Analytic sample: Not reported<br><br>Interventionist: Specialist healthy lifestyle midwife and healthy lifestyle advisors	Length of intervention: 7 months<br><br>Policy:<br><br>System: Screening and referral process for advanced care<br><br>Environmental: New hospital-based program to support behavior change and healthy weight gain<br><br>Fidelity: Yes	Intervention sustained beyond study: Yes<br><br>Reported sustainability of impact: No

Supplementary Data

Study Identifiers	Study Elements	Reach	Effectiveness	Adoption	Implementation	Maintenance
					Cost: No	
Middleton, 2019(110)<br><br>*Aboriginal family and baby bundles (ABFABB)*<br><br>Australia	Design: Repeated cross-sectional<br><br>Comparator: No control<br><br>Theory: Not reported	Sample: Aboriginal and Torres Strait Islander families accessing the Aboriginal family and baby bundles at the Adelaide Women's and Children's Hospital<br><br>Priority groups: Aboriginal people<br><br>Analytic sample: Not reported<br><br>Attrition: n/a	Outcomes:<br>Maternal diet: +<br><br>Subgroup analysis: No<br><br>Reported unintended consequences: No	Setting: Aboriginal Family Birthing Program at Adelaide Women's and Children's Hospital<br><br>Serve priority groups: Yes<br><br>Analytic sample: 1 birthing program<br><br>Interventionist: Not reported	Length of intervention: 18 months<br><br>Policy:<br><br>System: Novel package of culturally appropriate and intensive nutrition strategies embedded in the SA Aboriginal Family Birthing Program<br><br>Environmental: Provision of baby boxes that include healthy food, vouchers, and equipment to optimize lifestyle<br><br>Fidelity: No<br><br>Cost: No	Intervention sustained beyond study: Unsure<br><br>Reported sustainability of impact: No
Mustila, 2013(79)<br>Mustila, 2012(56)<br>(*Suppl. Ref*)<br>Mustil (NCT)a, 2009(57)<br>(*Suppl. Ref*)<br><br>*Vaasa Childhood Obesity Primary Prevention (VACOPP)*<br><br>Finland	Design: Non-randomized control trial<br><br>Comparator: Standard of care<br><br>Theory: Not reported	Sample: Mothers who speak Finnish and have BMI ≥ 25 kg/m^2, macrosomic newborn in any previous pregnancy, immediate family history of diabetes, and/or age ≥ 40 years<br><br>Priority groups: Overweight or obesity<br><br>Analytic sample: 185 women-infant dyads<br><br>Attrition: 14%	Outcomes:<br>Breastfeeding: θ<br>Child weight: θ<br>Maternal weight: θ<br><br>Subgroup analysis: No<br><br>Reported unintended consequences: Yes	Setting: Maternity clinics in Vaasa<br><br>Serve priority groups: Unsure<br><br>Analytic sample: 8 maternity clinics<br><br>Interventionist: Public health nurses, physiotherapist, and dietitian	Length of intervention: 6 years<br><br>Policy:<br><br>System: Incorporation of lifestyle program in standard care in maternity and child health care clinics<br><br>Environmental:<br><br>Fidelity: Yes<br><br>Cost: No	Intervention sustained beyond study: Unsure<br><br>Reported sustainability of impact: No
Nabulsi, 2014(58)<br>(*Suppl. Ref*)<br><br>*Complex breastfeeding promotion and support intervention*<br><br>Lebanon	Design: Randomized control trial<br><br>Comparator: Standard of care<br><br>Theory: Social Network Theory, Social Support Theory	Sample: Healthy pregnant women in the first or second trimester who intend to breastfeed after delivery<br><br>Priority groups: Unsure<br><br>Analytic sample: Not reported	Outcomes:<br>Ongoing study (breastfeeding)<br><br>Subgroup analysis: No<br><br>Reported unintended consequences: No	Setting: The Women's Health Center of the American University of Beirut Medical Center and the Obstetrics Clinics of Sahel General Hospital<br><br>Serve priority groups: Unsure	Length of intervention: 14 months<br><br>Policy:<br><br>System: Addition of breastfeeding education and counseling from professional lactation in hospital and establishing	Intervention sustained beyond study: Unsure<br><br>Reported sustainability of impact: Yes

Supplementary Data

**Study Identifiers**	**Study Elements**	**Reach**	**Effectiveness**	**Adoption**	**Implementation**	**Maintenance**
		Attrition: n/a		Analytic sample: Not reported<br><br>Interventionist: Nurses, lactation consultant, support mothers	a mother-to-mother tree of support<br><br>Environmental: Mother-to-mother tree of lay support to build social support and enhance social capital<br><br>Fidelity: Yes<br><br>Cost: Yes	
Nagle, 2011(59) (*Suppl. Ref*)<br><br>*Continuity of care*<br><br>Australia	Design: Randomized control trial<br><br>Comparator: Standard of care<br><br>Theory: Not reported	Sample: Primigravid women who have a BMI≥30 and are <17 weeks' gestation<br><br>Priority groups: Unsure<br><br>Analytic sample: Not reported<br><br>Attrition: n/a	Outcomes: Ongoing study (Maternal weight)<br><br>Subgroup analysis: No<br><br>Reported unintended consequences: No	Setting: Maternity services<br><br>Serve priority groups: Unsure<br><br>Analytic sample: Not reported<br><br>Interventionist: Midwives	Length of intervention: 9 months<br><br>Policy:<br><br>System: Continuity of care model<br><br>Environmental:<br><br>Fidelity: No<br><br>Cost: No	Intervention sustained beyond study: Unsure<br><br>Reported sustainability of impact: No
O'Reilly, 2021(60) (*Suppl. Ref*)<br>O'Brien, 2019(61) (*Suppl. Ref*)<br>O'Brien, 2019(62) (*Suppl. Ref*)<br>McAuliffe, 2019(63) (*Suppl. Ref*)<br><br>*LatchOn*<br><br>Ireland	Design: Randomized control trial<br><br>Comparator: Standard of care<br><br>Theory: COM-B Behaviour Change Framework	Sample: Primiparous women, singleton pregnancy, BMI ≥ 25 at booking visit, good understanding of English, have a support partner available and willing to participate<br><br>Priority groups: Overweight or obesity<br><br>Analytic sample: Not reported<br><br>Attrition: n/a	Outcomes: Ongoing study (breastfeeding)<br><br>Subgroup analysis: No<br><br>Reported unintended consequences: No	Setting: National Maternity Hospital in Dublin, Wexford General Hospital in Wexford, St Luke's General Hospital in Kilkenny, and Midlands Regional Hospital in Mullingar<br><br>Serve priority groups: Unsure<br><br>Analytic sample: 4 hospitals<br><br>Interventionist: International Board-Certified Lactation Consultants	Length of intervention: 5 months<br><br>Policy:<br><br>System: Changes to support for prospective mothers and their support partners through group education, individual consultation, phone call from IBCLC, and breastfeeding support group clinic<br><br>Environmental: Breastfeeding support clinics<br>Inclusion of support partners in education<br><br>Fidelity: No<br><br>Cost: No	Intervention sustained beyond study: Unsure<br><br>Reported sustainability of impact: Yes

Supplementary Data

Study Identifiers	Study Elements	Reach	Effectiveness	Adoption	Implementation	Maintenance
Olayiwola, 2013(111)<br><br>*Living Smart, Living Fit*<br><br>USA	Design: Single group, pre-post design, pilot study<br><br>Comparator: No control<br><br>Theory: Not reported	Sample: Pregnant women with a PHQ-9 score ≥10 or documented mood disorder or BMI >25 kg/m^2<br><br>Priority groups: Low income, race and ethnicity, overweight or obesity<br><br>Analytic sample: 68 women<br><br>Attrition: 36%	Outcomes:<br>Child weight: θ<br>Maternal weight: θ<br><br>Subgroup analysis: Engagement with services<br><br>Reported unintended consequences: No	Setting: Community Health Center, Inc in Connecticut<br><br>Serve priority groups: Yes<br><br>Analytic sample: Not reported<br><br>Interventionist: Intake staff and clinical care coordinators	Length of intervention: 8 months<br><br>Policy:<br><br>System: Link patients to clinical care coordinators trained in motivational interviewing who promoted participation in a portfolio of mental and physical wellness activities<br><br>Environmental: Initiation of Living Smart, Living Fit program<br><br>Fidelity: Yes<br><br>Cost: No	Intervention sustained beyond study: Unsure<br><br>Reported sustainability of impact: No
Parat, 2019(144)<br><br>*Prenatal education for pregnant women with overweight or obesity (ETOIG)*<br><br>France	Design: Randomized control trial<br><br>Comparator: No intervention<br><br>Theory: Not reported	Sample: Pregnant women ≤ 21 weeks' gestation with a pre-pregnancy BMI > 25 kg/m^2 having a singleton pregnancy, able to understand French and covered by French medical insurance system<br><br>Priority groups: Pregnant people with overweight or obesity<br><br>Analytic sample: 268 mother-infant pairs<br><br>Attrition: 3%	Outcomes:<br>Breastfeeding: θ<br>Complementary feeding: θ<br>Child weight: θ<br><br>Subgroup analysis: Per protocol, available data<br><br>Reported unintended consequences: Yes	Setting: University hospitals<br><br>Serve priority groups: No<br><br>Analytic sample: 4 university hospitals<br><br>Interventionist: Physician (pediatrician or endocrinologist), dietician, or a midwife	Length of intervention: 9 months<br><br>Policy:<br><br>System: Change format of service delivery to include more dietitian visits and group education sessions<br><br>Environmental:<br><br>Fidelity: No<br><br>Cost: No	Intervention sustained beyond study: Unsure<br><br>Reported sustainability of impact: Yes
Robertson, 2009(173)<br><br>*CenteringPregnancy®*<br><br>USA	Design: Non-randomized control trial<br><br>Comparator: Standard of care<br><br>Theory: Not reported	Sample: Women who self-identify Hispanic ethnicity and able to read and speak English or Spanish who attended at least four prenatal visits<br><br>Priority groups: Low income, ethnicity	Outcomes:<br>Breastfeeding: θ<br><br>Subgroup analysis: No<br><br>Reported unintended consequences: No	Setting: Hospital-based clinic<br><br>Serve priority groups: Yes<br><br>Analytic sample: 1 hospital-based clinic	Length of intervention: 16 weeks<br><br>Policy:<br><br>System: Change from individual to group model of prenatal care	Intervention sustained beyond study: Yes<br><br>Reported sustainability of impact: No

Supplementary Data

Study Identifiers	Study Elements	Reach	Effectiveness	Adoption	Implementation	Maintenance
		Analytic sample: 33 women<br><br>Attrition: 33%		Interventionist: Healthcare provider	Environmental: Support group; invitation for spouses and support people to participate<br><br>Fidelity: No<br><br>Cost: No	
Savitri, 2016(106)<br><br>*BReastfeeding Attitude and Volume Optimization (BRAVO)*<br><br>Indonesia	Design: Cluster randomized control trial<br><br>Comparator: Standard of care<br><br>Theory: Not reported	Sample: Pregnant women under primary or referred healthcare who intend to breastfeed newborn shortly or not at all<br><br>Priority groups: Unsure<br><br>Analytic sample: Not reported<br><br>Attrition: n/a	Outcomes: Ongoing study (breastfeeding, child weight)<br><br>Subgroup analysis: No<br><br>Reported unintended consequences: No	Setting: Budi Kemuliaan Hospital in Jakarta<br><br>Serve priority groups: Yes<br><br>Analytic sample: Not reported<br><br>Interventionist: Lactation manager, midwives, or primary care physicians	Length of intervention: 12 months<br><br>Policy:<br><br>System: Coordination by lactation manager to provide breast pump and written advocacy to employers for accommodations; additional lactation support visit through 6 months postpartum; centrally managed short messages service; breastfeeding hotline<br><br>Environmental: Coordination to provide breast pump and written advocacy to employers to provide lactation room and time to express milk; peer support<br><br>Fidelity: No<br><br>Cost: No	Intervention sustained beyond study: No<br><br>Reported sustainability of impact: Yes
Tanner-Smith, 2013(159)<br>Tanner-Smith, 2014(64) (*Suppl. Ref*)<br><br>*CenteringPregnancy®*<br><br>USA	Design: Retrospective cohort study<br><br>Comparator: Standard of care<br><br>Theory: Not reported	Sample: Women who received Centering Pregnancy or traditional prenatal care<br><br>Priority groups: Low income, race and ethnicity, rural<br><br>Analytic sample: 794 prenatal care recipients	Outcomes:<br>Breastfeeding: +<br>Maternal weight: +*<br>Child weight: θ<br><br>Subgroup analysis: No<br><br>Reported unintended consequences: Yes	Setting: Prenatal care sites in Tennessee<br><br>Serve priority groups: Yes<br><br>Analytic sample: 4 prenatal care sites<br><br>Interventionist: Certified nurse-midwives, physicians, licensed	Length of intervention: 6 months<br><br>Policy:<br><br>System: Change from individual to group model of prenatal care<br><br>Environmental: Peer support	Intervention sustained beyond study: Yes<br><br>Reported sustainability of impact: No

Supplementary Data

Study Identifiers	Study Elements	Reach	Effectiveness	Adoption	Implementation	Maintenance
		Sub-study: 393 prenatal recipients<br><br>Attrition:<br>Breastfeeding: 50%<br>Gestational Weight Gain: 31%		practical nurses, advanced practice nurses, midwives, and doulas	Fidelity: No<br><br>Cost: No	
Trotman, 2015(169)<br><br>*CenteringPregnancy®*<br><br>USA	Design: Retrospective cohort<br><br>Comparator: Standard of care<br><br>Theory: Not reported	Sample: Pregnant adolescents, aged 11-21 years<br><br>Priority groups: Low income, race and ethnicity, adolescent mothers<br><br>Analytic sample: 150 charts<br><br>Attrition: n/a	Outcomes:<br>Breastfeeding: +*<br>Maternal weight: +<br><br>Subgroup analysis: No<br><br>Reported unintended consequences: No	Setting: MedStar Washington, DC Hospital Center Obstetrics and Gynecology clinic - teaching hospital in an urban community<br><br>Serve priority groups: Yes<br><br>Analytic sample: 1 clinic<br><br>Interventionist: certified nurse midwives or an attending obstetrician	Length of intervention: 8 months<br><br>Policy:<br><br>System: Change from individual to group model of prenatal care<br><br>Environmental: Group sessions for socialization and to build relationships<br><br>Fidelity: Yes<br><br>Cost: No	Intervention sustained beyond study: Unsure<br><br>Reported sustainability of impact: No
Trudnak, 2011(134)<br>Trudnak, 2013(65)<br>(*Suppl. Ref*)<br><br>*CenteringPregnancy®*<br><br>USA	Design: Retrospective cohort<br><br>Comparator: Standard of care<br><br>Theory: Not reported	Sample: Women self-identifying as Spanish-speaking and Hispanic who entered into prenatal care<br><br>Priority groups: Ethnicity<br><br>Analytic sample: 487 patient charts<br><br>Attrition: n/a	Outcomes:<br>Breastfeeding: -*<br>Child weight: θ<br>Maternal weight: -*<br><br>Subgroup analysis: No<br><br>Reported unintended consequences: No	Setting: Pinellas County Health Department-Clearwater clinic, Florida<br><br>Serve priority groups: Yes<br><br>Analytic sample: 1 county health department<br><br>Interventionist: Health educator, physician, obstetricians	Length of intervention: 8 months<br><br>Policy:<br><br>System: Change from individual to group model of prenatal care<br><br>Environmental: Group sessions for socialization and to build relationships<br><br>Fidelity: Yes<br><br>Cost: No	Intervention sustained beyond study: Unsure<br><br>Reported sustainability of impact: No
Tubay, 2019(154)<br><br>*CenteringPregnancy®*<br><br>USA	Design: Randomized control trial<br><br>Comparator: Standard of care<br><br>Theory: Not reported	Sample: Pregnant with a gestational age < 19 weeks and Department of Defense active duty or dependent beneficiary<br><br>Priority groups: Race and ethnicity, low education	Outcomes:<br>Breastfeeding: θ<br>Child weight: +*<br>Maternal weight: +<br><br>Subgroup analysis: Participants that had gestational diabetes	Setting: David Grant USAF Medical Center, Travis Air Force Base, California<br><br>Serve priority groups: Unsure	Length of intervention: 6 months<br><br>Policy:<br><br>System: Change from individual to group model of prenatal care	Intervention sustained beyond study: Unsure<br><br>Reported sustainability of impact: No

Supplementary Data

Study Identifiers	Study Elements	Reach	Effectiveness	Adoption	Implementation	Maintenance
		Analytic sample: 129 prenatal patients<br><br>Attrition: 28%	Reported unintended consequences: No	Analytic sample: 1 medical treatment facility<br><br>Interventionist: Certified nurse midwives and family medicine residents and faculty	Environmental: Group sessions for socialization and to build relationships<br><br>Fidelity: No<br><br>Cost: No	
Walton, 2015(170)<br><br>*CenteringPregnancy®*<br><br>USA	Design: Retrospective cohort study<br><br>Comparator: Standard of care<br><br>Theory: Not reported	Sample: Dependent wives of active duty service members or active duty members<br><br>Priority groups: No<br><br>Analytic sample: 404 patients<br><br>Attrition: n/a	Outcomes:<br>Breastfeeding: θ<br>Maternal weight: θ<br>Child weight: θ<br><br>Subgroup analysis: No<br><br>Reported unintended consequences: Yes	Setting: Naval Medical Center San Diego<br><br>Serve priority groups: No<br><br>Analytic sample: 1 tertiary care teaching hospital with outpatient clinics<br><br>Interventionist: Certified nurse midwives	Length of intervention: 8 months<br><br>Policy:<br><br>System: Change from individual to group model of prenatal care<br><br>Environmental: Peer support<br><br>Fidelity: No<br><br>Cost: No	Intervention sustained beyond study: Unsure<br><br>Reported sustainability of impact: No
Watt, 2015(125)<br><br>*Primary-care based early childhood nutrition intervention*<br><br>USA	Design: Non-randomized control trial, pilot study<br><br>Comparator: No intervention<br><br>Theory: Not reported	Sample: Pregnant women attending a primary care clinic that serves Hispanic women with low income<br><br>Priority groups: Low income, ethnicity<br><br>Analytic sample: 43 women<br><br>Attrition: 30%	Outcomes:<br>Breastfeeding: +<br>Child weight: θ<br>Maternal diet: +*<br>Maternal weight: -<br><br>Subgroup analysis: No<br><br>Reported unintended consequences: No	Setting: Primary care clinic in the Southwest<br><br>Serve priority groups: Yes<br><br>Analytic sample: 1 primary care clinic<br><br>Interventionist: Clinic health educator and lactations consultant and non-profit community partner facilitator	Length of intervention: 15 months<br><br>Policy:<br><br>System: Incorporation of classes and lactation counseling to regular prenatal and well child care visits<br><br>Environmental: Group classes to develop a cohort of women for support and encouragement Farmers' market vouchers for fruit and vegetables<br><br>Fidelity: Yes<br><br>Cost: No	Intervention sustained beyond study: Unsure<br><br>Reported sustainability of impact: No
Wilkinson, 2018(112)<br>Wilkinson, 2013(66)<br>(*Suppl. Ref*)	Design: Cohort	Sample: Women receiving postnatal care	Outcomes:<br>Breastfeeding: θ<br>Maternal diet: θ	Setting: South-East Queensland tertiary maternity hospital	Length of intervention: 10 years	Intervention sustained beyond study: Unsure

Supplementary Data

Study Identifiers	Study Elements	Reach	Effectiveness	Adoption	Implementation	Maintenance
Tertiary maternity hospital nutrition service iterative redesign<br><br>Australia	Comparator: No intervention<br><br>Theory: Knowledge-to-Action Framework	in the Maternal Health Hospital<br><br>Priority groups: Unsure<br><br>Analytic sample: 2014-2017: 421 postnatal surveys<br>2008: 101 postnatal surveys<br><br>2011: 494 antenatal and postnatal surveys<br>2008: 405 antenatal and postnatal surveys<br><br>Attrition: n/a	Maternal weight: +*<br><br>Subgroup analysis: Pre-pregnancy BMI<br><br>Reported unintended consequences: No	Serve priority groups: Unsure<br><br>Analytic sample: 1 hospital<br><br>Interventionist: Nutrition and dietetics maternity service	Policy:<br><br>System: Improved promotion of dietetic services and education to hospital staff; translation of nutrition practice guidelines and integration of dietitian into antenatal clinic<br><br>Environmental: Creation of pre- and postnatal outpatient nutrition programs<br><br>Fidelity: No<br><br>Cost: No	Reported sustainability of impact: No
Zielinski, 2014(93)<br>Gogel, 2013(67) (*Suppl. Ref*)<br><br>*CenteringPregnancy®*<br><br>USA	Design: Retrospective cohort study<br><br>Comparator: Standard of care<br><br>Theory: Not reported	Sample: CenteringPregnancy and traditional care participants matched on race, age, and insurance status<br><br>Priority groups: Low income, race, overweight or obesity<br><br>Analytic sample: 343 prenatal patients<br><br>Attrition: 1%	Outcomes:<br>Breastfeeding: +*<br>Maternal weight: ϴ<br><br>Subgroup analysis: No<br><br>Reported unintended consequences: No	Setting: Prenatal clinic sites in southwest Michigan in a certified nurse-midwifery, hospital-based practice<br><br>Serve priority groups: Yes<br><br>Analytic sample: 2 prenatal clinics<br><br>Interventionist: Health care providers including certified nurse midwives	Length of intervention: 6 months<br><br>Policy: Health center policy to offer CenteringPregnancy to all women who qualify for midwifery care<br><br>System: Change from individual to group model of prenatal care<br><br>Environmental: Group sessions for socialization and to build relationships<br><br>Fidelity: No<br><br>Cost: No	Intervention sustained beyond study: Unsure<br><br>Reported sustainability of impact: No
**Prenatal and pediatric primary care (n=8)**						
Diaz-Rodriguez, 2020(61)<br><br>*Multifactorial intervention*<br><br>Spain	Design: Non-randomized control trial<br><br>Comparator: Standard of care<br><br>Theory: Not reported	Sample: Two-year-old infants whose pregnancy began in the 14 months following the start of the intervention, and whose mothers' completed follow-up of their pregnancy	Outcomes:<br>Ongoing study (child weight, maternal weight)<br><br>Subgroup analysis: No<br><br>Reported unintended consequences: No	Setting: Clinical Management Units of Puerto Real<br><br>Serve priority groups: Unsure<br><br>Analytic sample: Not reported	Length of intervention: 30 months<br><br>Policy:<br><br>System: Integration of intervention into routine primary care health programs	Intervention sustained beyond study: Unsure<br><br>Reported sustainability of impact: No

Supplementary Data

Study Identifiers	Study Elements	Reach	Effectiveness	Adoption	Implementation	Maintenance
		and began the monitoring of child health<br><br>Priority groups: Unsure<br><br>Analytic sample: Not reported<br><br>Attrition: n/a		Interventionist: Nurse midwife	Environmental:<br><br>Fidelity: No<br><br>Cost: No	
Ekström, 2012(156)<br><br>*Breastfeeding training program for healthcare professionals*<br><br>Sweden	Design: Cluster randomized control trial<br><br>Comparator: Standard of care<br><br>Theory: Not reported	Sample: Swedish-speaking, healthy, first-time mothers who gave birth to single, healthy, full-term babies<br><br>Priority groups: Unsure<br><br>Analytic sample: 187 mothers<br><br>Attrition: 39%	Outcomes:<br>Breastfeeding: +<br>Complementary feeding: +*<br><br>Subgroup analysis: No<br><br>Reported unintended consequences: No	Setting: Prenatal and child health centers in largest municipalities<br><br>Serve priority groups: Yes<br><br>Analytic sample: 10 municipalities<br><br>Interventionist: Midwives and postnatal nurses	Length of intervention: Not reported<br><br>Policy:<br><br>System: Process-oriented training program for midwives and postnatal nurses<br><br>Environmental: Social support<br><br>Fidelity: No<br><br>Cost: No	Intervention sustained beyond study: Unsure<br><br>Reported sustainability of impact: No
Ekström, 2014(152)<br><br>*Use of nipple shields*<br><br>Sweden	Design: Randomized control trial<br><br>Comparator: Standard of care<br><br>Theory: Not reported	Sample: Swedish-speaking, healthy, first-time mothers who gave birth to single, healthy, full-term babies<br><br>Priority groups: Unsure<br><br>Analytic sample: 403 mothers<br><br>Attrition: 16%	Outcomes:<br>Breastfeeding: +*<br>Child weight: Not reported<br><br>Subgroup analysis: Use of nipple shield<br><br>Reported unintended consequences: No	Setting: Largest municipalities with antenatal and child health centers<br><br>Serve priority groups: Unsure<br><br>Analytic sample: 10 municipalities<br><br>Interventionist: Midwives and child health nurses	Length of intervention: 9 months<br><br>Policy:<br><br>System: Process-oriented training program for midwives and postnatal nurses<br><br>Environmental:<br><br>Fidelity: No<br><br>Cost: No	Intervention sustained beyond study: Unsure<br><br>Reported sustainability of impact: No
Ferreira, 2018(117)<br><br>*Primary health care intervention for infant feeding practices*<br><br>Brazil	Design: Cluster randomized control trial<br><br>Comparator: Standard of care<br><br>Theory: Not reported	Sample: Pregnant women in the last trimester<br><br>Priority groups: low income, low education<br><br>Analytic sample:	Outcomes:<br>Child diet: +*<br>Breastfeeding: θ<br>Bottle feeding and Complementary feeding: +*	Setting: Health centres part of the National Health Care System<br><br>Serve priority groups: Yes	Length of intervention: Not reported<br><br>Policy:<br><br>System: Promote adoption of Ten Steps to Successful	Intervention sustained beyond study: Yes<br><br>Reported sustainability of impact: No

Supplementary Data

Study Identifiers	Study Elements	Reach	Effectiveness	Adoption	Implementation	Maintenance
		6 months: 617 mother-child pairs<br>12 months: 516 mother-child pairs<br><br>Attrition:<br>6 months: 14%<br>12 months: 28%	Subgroup analysis: Recommended number of clinic visits<br><br>Reported unintended consequences: Yes	Analytic sample: 20 health clinics<br><br>Interventionist: Physicians, nurses, and administrative staff	Breastfeeding; provision of printed materials and a pocket guide for health staff to use during appointments and waiting room sessions<br><br>Environmental: Posters to promote breastfeeding added to waiting rooms; provision of pocket guides for health staff to use during appointments and waiting room sessions<br><br>Fidelity: No<br><br>Cost: No	
Flax, 2022(164)<br><br>*Breastfeeding interpersonal communication, mobile phone support, and mass media messaging*<br><br>Nigeria	Design: Cohort<br><br>Comparator: No intervention<br><br>Theory: Not reported	Sample: Women in their third trimester of pregnancy, and current clients of a private health facility. Continued eligibility if infant alive and no contraindications for breastfeeding.<br><br>Priority groups: No<br><br>Analytic sample: 1,106 postpartum women<br><br>Attrition: 8%	Outcomes:<br>Breastfeeding: +*<br><br>Subgroup analysis: No<br><br>Reported unintended consequences: No	Setting: Private health facilities that provided maternity and pediatric services and registered with the Association of General and Private Medical Practitioners of Nigeria and the Health Facility Monitoring and Accreditation Agency<br><br>Serve priority groups: Unsure<br><br>Analytic sample: 20 private health facilities<br><br>Interventionist: Facility managers and healthcare providers	Length of intervention: 9 months<br><br>Policy:<br><br>System: Promote adoption of Ten Steps to Successful Breastfeeding; incorporate interpersonal communication and counseling at antenatal care; distribute materials at all points of clinical care; broadcast mass media messages; deliver mobile phone messaging<br><br>Environmental: Peer support; text inclusion of influential family members in text messages; mass media to influence knowledge and norms around breastfeeding<br><br>Fidelity: Yes<br><br>Cost: No	Intervention sustained beyond study: Unsure<br><br>Reported sustainability of impact: No

Supplementary Data

Study Identifiers	Study Elements	Reach	Effectiveness	Adoption	Implementation	Maintenance
Gross, 2016(78)<br>Messito, 2020(68)<br>(*Suppl. Ref*)<br>Messito, 2020(69)<br>(*Suppl. Ref*)<br>Messito, 2019(70)<br>(*Suppl. Ref*)<br>Messito, 2018(71)<br>(*Suppl. Ref*)<br>Clinical trials, 2012(72)<br>(*Suppl. Ref*)<br><br>*Starting Early Obesity Prevention Program*<br><br>USA	Design: Randomized control trial<br><br>Comparator: Standard of care<br><br>Theory: Health Belief Model, Social Cognitive Theory	Sample: Women with a singleton uncomplicated pregnancy who self-identified as Latina or Hispanic and spoke English or Spanish and could provide phone numbers<br><br>Priority groups: Low income and ethnicity<br><br>Analytic sample: 412 mother-infant dyads<br><br>Attrition: 27%	Outcomes:<br>Breastfeeding: +*<br>Complementary feeding: +*<br>Feeding style: +*<br>Child weight: +*<br><br>Subgroup analysis: Dose-response<br><br>Reported unintended consequences: Yes	Setting: Primary care prenatal and pediatric clinics and the postpartum ward of a large urban public hospital and an affiliated satellite neighborhood health center<br><br>Serve priority groups: Yes<br><br>Analytic sample: Not reported<br><br>Interventionist: Bilingual English- and Spanish-speaking registered dietitians who are certified lactation counselor	Length of intervention: 3 years<br><br>Policy:<br><br>System: Enhanced standard care with nutrition counseling during prenatal visits and nutrition and parenting support groups coordinated with well-child visits<br><br>Environmental: Support groups encouraged peer interaction and social support<br><br>Fidelity: Yes<br><br>Cost: No	Intervention sustained beyond study: Unsure<br><br>Reported sustainability of impact: No
Kinnunen, 2007(177)<br>Kinnunen, 2007(73)<br>(*Suppl. Ref*)<br>Mustila, 2012(74)<br>(*Suppl. Ref*)<br>Mustila, 2012(75)<br>(*Suppl. Ref*)<br><br>*Lifestyle counseling in primary health care*<br><br>Finland	Design: Non-randomized control trial, pilot study<br><br>Comparator: Standard of care<br><br>Theory: Not reported	Sample: Pregnant mothers with no previous deliveries<br><br>Priority groups: Unsure<br><br>Analytic sample: 72 pregnant mothers<br><br>Attrition: 34%	Outcomes:<br>Child weight: θ<br>Maternal diet: +<br>Maternal weight: θ<br><br>Subgroup analysis: No<br><br>Reported unintended consequences: No	Setting: Primary care maternity and child health clinics in the cities of Tampere and Hämeenlinna<br><br>Serve priority groups: Unsure<br><br>Analytic sample: 6 maternity clinics<br><br>Interventionist: Public health nurses	Length of intervention: 15 months<br><br>Policy:<br><br>System: Incorporation of individual counseling to routine visits with maternity health care nurse<br><br>Environmental:<br><br>Fidelity: Yes<br><br>Cost: No	Intervention sustained beyond study: Unsure<br><br>Reported sustainability of impact: Yes
Rosen-Carole, 2016(168)<br><br>*Baby-Friendly Hospital Initiative*<br><br>USA	Design: Time series<br><br>Comparator: No control<br><br>Theory: Not reported	Sample: Infant charts from well child visits<br><br>Priority groups: Unsure<br><br>Analytic sample: 1,948 infant charts<br><br>Attrition: Unable to calculate	Outcomes:<br>Breastfeeding: +*<br><br>Subgroup analysis: No<br><br>Reported unintended consequences: No	Setting: Institute for Family Health<br><br>Serve priority groups: Yes<br><br>Analytic sample: 31 health centers	Length of intervention: Policy: n/a<br><br>Policy: Breastfeeding policy adopted<br><br>System: Increased breastfeeding training for residents; creation of breastfeeding education coordinator position to	Intervention sustained beyond study: Yes<br><br>Reported sustainability of impact: No

Supplementary Data

Study Identifiers	Study Elements	Reach	Effectiveness	Adoption	Implementation	Maintenance
				Interventionist: Coordinator and healthcare staff	meet with every breastfeeding mother-infant dyad at every visit and to conduct follow-up outreach; identification of community resources and creation of local referral lists added to electronic medical record<br><br>Environmental: Removal of formula samples; appropriate facilities for employees to express milk at work; presentations and events to build institutional and community support for breastfeeding<br><br>Fidelity: No<br><br>Cost: No	

Supplementary Data

Supplemental Table 5. Downs and Black study quality rating checklist for included studies[a]

Author, Year	1. Objective	2. Outcomes	3. Participants	4. Interventions	5. Confounders	6. Findings	7. Variability	8. Adverse Events	9. Lost to Follow Up	10. Probability Values	11. Representative - Recruitment	12. Representative - Enrollment	13. Representative - Setting	14. Blinding - Participants	15. Blinding - Assessors	16. Data Dredging	17. Length of Follow Up	18. Statistical Analysis	19. Intervention Compliance	20. Outcome Measures	21. Recruitment - Same Population	22. Recruitment - Time Period	23. Randomization	24. Randomization Assignment	25. Analysis - Confounding	26. Analysis - Loss to Follow Up	27. Power Analysis
Abrahams, 2009 (186)	Y	Y	N	Y	N	Y	N	N	N	Y	U	U	U	U	N	Y	Y	Y	U	Y	Y	N	N	N	U	U	N
Alberdi, 2018(115)	Y	N	Y	Y	N	Y	N	N	Y	N	U	U	U	N	N	Y	Y	Y	Y	U	U	U	N	N	N	Y	N
Anderson, 2005 (155)	Y	Y	Y	Y	Y	Y	Y	N	N	N	U	U	Y	N	N	Y	Y	Y	Y	Y	Y	Y	Y	U	Y	U	N
Beck, 2014(123)	Y	Y	Y	Y	Y	Y	N	N	Y	Y	Y	Y	Y	N	U	Y	U	Y	Y	Y	Y	Y	N	N	N	Y	N
Bonuck, 2014(118)	Y	Y	Y	Y	Y	Y	Y	N	Y	Y	U	U	U	N	N	Y	Y	Y	Y	Y	Y	Y	Y	N	Y	Y	Y
Brodribb, 2013(132)	Y	Y	Y	Y	Y	Y	N	N	N	Y	Y	N	Y	N	N	Y	Y	Y	Y	Y	Y	Y	N	N	N	N	U
Brownfoot, 2016(99)	Y	Y	Y	Y	Y	Y	Y	N	Y	Y	U	U	U	U	N	Y	Y	Y	Y	Y	Y	Y	Y	Y	U	Y	Y
Brumley, 2016 (182)	Y	Y	Y	Y	N	Y	Y	N	Y	Y	U	U	Y	N	N	N	Y	Y	U	Y	Y	Y	N	N	N	N	N
Budge, 2023(141)	Y	Y	Y	Y	Y	Y	Y	N	N	Y	Y	U	Y	N	N	Y	Y	Y	U	Y	Y	N	N	N	Y	Y	N
Chae, 2017(163)	Y	Y	Y	Y	Y	Y	Y	N	N	Y	U	U	Y	N	N	Y	Y	Y	Y	Y	Y	Y	N	N	Y	N	Y
Chapman, 2013(121)	Y	Y	Y	Y	Y	Y	Y	N	N	Y	U	U	N	N	Y	Y	Y	Y	Y	Y	Y	Y	Y	Y	Y	Y	Y
Chapman, 2004(120)	Y	Y	Y	Y	N	Y	Y	N	Y	Y	U	U	Y	N	Y	Y	Y	Y	N	U	Y	Y	Y	U	Y	Y	N
Chiurco, 2015(103)	Y	Y	Y	Y	N	Y	Y	N	Y	Y	U	U	U	U	N	Y	Y	Y	U	U	Y	N	N	N	U	Y	N
Chwah, 2016(171)	Y	Y	Y	Y	Y	Y	Y	N	Y	Y	Y	U	N	N	N	Y	Y	Y	U	Y	Y	Y	N	N	N	Y	N
Corriveau, 2013(95)	Y	Y	Y	Y	Y	Y	N	N	Y	N	Y	U	Y	N	Y	Y	Y	Y	Y	Y	Y	N	N	N	Y	Y	Y
Daley, 2015(100)	Y	Y	Y	Y	Y	Y	Y	Y	Y	N	Y	U	Y	N	N	Y	Y	Y	Y	Y	Y	Y	Y	Y	N	N	Y
de Jersey, 2022(89)	Y	Y	Y	Y	Y	Y	Y	N	Y	N	Y	Y	U	U	Y	Y	Y	Y	Y	Y	Y	N	N	N	Y	Y	N
Donkoh, 2013(179)	Y	Y	Y	Y	N	Y	Y	N	Y	Y	U	U	Y	U	N	Y	Y	Y	N	Y	N	Y	N	N	N	Y	Y
Döring, 2016(82)	Y	Y	Y	Y	Y	Y	Y	N	Y	Y	N	N	Y	N	N	Y	Y	Y	Y	Y	Y	Y	Y	N	Y	Y	Y
Ekström, 2012(156)	Y	Y	Y	N	Y	Y	Y	N	Y	Y	U	U	Y	Y	U	Y	Y	Y	U	Y	N	Y	Y	U	Y	N	N
Ekström, 2014(152)	Y	Y	Y	Y	Y	Y	Y	N	Y	Y	U	U	U	Y	U	Y	Y	Y	U	Y	N	U	Y	N	Y	Y	Y
Feldman-Winter, 2010 (176)	Y	Y	Y	Y	Y	Y	Y	N	Y	Y	U	U	U	N	N	Y	Y	Y	U	Y	N	Y	N	N	U	Y	Y

Supplementary Data

Author, Year	1. Objective	2. Outcomes	3. Participants	4. Interventions	5. Confounders	6. Findings	7. Variability	8. Adverse Events	9. Lost to Follow Up	10. Probability Values	11. Representative - Recruitment	12. Representative - Enrollment	13. Representative - Setting	14. Blinding - Participants	15. Blinding - Assessors	16. Data Dredging	17. Length of Follow Up	18. Statistical Analysis	19. Intervention Compliance	20. Outcome Measures	21. Recruitment - Same Population	22. Recruitment - Time Period	23. Randomization	24. Randomization Assignment	25. Analysis - Confounding	26. Analysis - Loss to Follow Up	27. Power Analysis
Ferreira, 2018(117)	Y	Y	Y	Y	Y	Y	Y	Y	Y	Y	U	U	Y	N	Y	Y	Y	Y	U	Y	N	Y	Y	N	U	Y	Y
Flax, 2022(164)	Y	Y	Y	Y	Y	Y	Y	N	Y	Y	U	U	Y	N	N	Y	Y	Y	N	Y	N	Y	N	N	Y	Y	Y
French, 2012(146)	Y	Y	Y	Y	Y	Y	Y	N	Y	Y	Y	U	U	U	N	Y	Y	Y	Y	Y	Y	Y	Y	N	Y	Y	Y
Gagnon, 1997(147)	Y	Y	Y	Y	Y	Y	Y	Y	Y	N	U	U	U	N	Y	Y	Y	Y	U	Y	Y	Y	Y	N	Y	Y	Y
Garmendia, 2020(145)	Y	Y	Y	Y	Y	Y	Y	Y	Y	Y	U	U	Y	N	U	Y	Y	Y	Y	Y	Y	Y	Y	N	Y	Y	Y
Gomes, 2019(87)	Y	Y	Y	Y	Y	Y	Y	N	N	Y	Y	Y	Y	N	N	Y	Y	Y	Y	Y	Y	Y	N	N	Y	N	N
Graça, 2011(180)	Y	Y	Y	Y	Y	Y	Y	N	N	Y	Y	U	Y	Y	N	Y	N	N	U	Y	U	Y	U	N	N	N	N
Grant, 2018(184)	Y	Y	Y	Y	N	Y	N	N	N	N	U	U	U	N	N	Y	Y	Y	U	Y	Y	Y	N	N	N	Y	N
Gregory, 2016(109)	Y	Y	Y	Y	Y	Y	Y	N	N	Y	U	U	U	N	N	Y	Y	Y	U	Y	Y	N	N	N	U	Y	N
Gross, 2016(78)	Y	Y	Y	Y	Y	Y	Y	Y	Y	Y	U	U	U	U	Y	Y	Y	Y	Y	Y	Y	Y	Y	N	Y	Y	Y
Gross, 2022(165)	Y	Y	Y	Y	Y	Y	N	N	Y	Y	Y	U	Y	U	U	Y	Y	Y	U	Y	Y	Y	N	N	Y	Y	N
Grossman, 2009(172)	Y	Y	Y	Y	Y	Y	N	N	Y	Y	Y	Y	Y	N	N	Y	N	Y	U	Y	Y	N	N	N	Y	N	Y
Haby, 2015(124)	Y	Y	Y	Y	Y	Y	Y	N	Y	Y	U	U	Y	U	U	Y	Y	Y	Y	Y	Y	Y	N	N	Y	Y	N
Hale, 2023(128)	Y	Y	N	Y	N	Y	N	N	N	N	U	U	U	U	N	Y	Y	Y	Y	Y	N	Y	N	N	N	N	N
Hannula, 2014(113)	Y	Y	Y	Y	Y	Y	Y	N	N	Y	U	U	Y	U	U	Y	Y	Y	U	Y	N	Y	N	N	Y	N	U
Hawkins, 2015(166)	Y	Y	Y	Y	Y	Y	Y	N	Y	Y	U	U	Y	U	U	Y	Y	Y	Y	Y	N	Y	N	N	Y	Y	N
Heberlein, 2016(130)	Y	Y	Y	Y	Y	Y	Y	N	Y	Y	U	U	Y	N	N	Y	Y	Y	Y	Y	Y	Y	N	N	Y	Y	N
Hoch, 2023(90)	Y	Y	N	Y	N	Y	Y	N	N	Y	U	U	Y	U	N	Y	Y	Y	U	Y	Y	N	N	N	U	U	Y
Hoffman, 2021(148)	Y	Y	Y	Y	Y	Y	Y	N	Y	Y	U	U	Y	U	U	Y	Y	Y	Y	Y	N	Y	Y	N	Y	Y	Y
Holmes, 2012(185)	Y	Y	N	Y	N	Y	N	N	N	Y	U	U	U	N	N	Y	Y	Y	Y	Y	N	Y	N	N	N	Y	N
Hopkinson, 2009(114)	Y	Y	Y	Y	Y	Y	Y	N	Y	Y	U	U	Y	N	Y	Y	Y	Y	Y	Y	Y	Y	Y	Y	Y	Y	Y
Ickovics, 2016(150)	Y	Y	Y	Y	Y	Y	Y	N	Y	Y	U	U	U	N	U	Y	Y	Y	U	Y	N	Y	Y	Y	Y	Y	Y
Kair, 2013(133)	Y	Y	Y	Y	Y	Y	N	N	Y	N	Y	Y	Y	N	U	Y	Y	Y	Y	Y	Y	N	N	N	N	Y	N

Supplementary Data

Author, Year	1. Objective	2. Outcomes	3. Participants	4. Interventions	5. Confounders	6. Findings	7. Variability	8. Adverse Events	9. Lost to Follow Up	10. Probability Values	11. Representative - Recruitment	12. Representative - Enrollment	13. Representative - Setting	14. Blinding - Participants	15. Blinding - Assessors	16. Data Dredging	17. Length of Follow Up	18. Statistical Analysis	19. Intervention Compliance	20. Outcome Measures	21. Recruitment - Same Population	22. Recruitment - Time Period	23. Randomization	24. Randomization Assignment	25. Analysis - Confounding	26. Analysis - Loss to Follow Up	27. Power Analysis
Kettrey, 2020(136)	Y	Y	Y	Y	Y	Y	Y	N	Y	N	Y	Y	Y	N	U	Y	Y	Y	Y	Y	Y	Y	N	N	Y	U	Y
Kinnunen, 2007(177)	Y	Y	Y	Y	P	Y	Y	N	Y	Y	U	U	Y	N	N	Y	Y	Y	Y	Y	N	Y	N	N	Y	N	N
Kistin, 1994(137)	Y	Y	Y	Y	Y	Y	N	Y	Y	N	U	U	Y	N	N	Y	Y	Y	U	Y	Y	Y	N	N	U	Y	N
Klima, 2009(181)	Y	Y	Y	Y	P	Y	Y	N	Y	N	U	U	U	N	U	Y	Y	Y	U	Y	Y	Y	N	N	U	Y	N
Kramer, 2001(149)	Y	Y	Y	Y	Y	Y	Y	N	Y	Y	U	U	Y	U	N	Y	Y	Y	U	Y	N	Y	Y	Y	Y	Y	Y
Machuca, 2016(88)	Y	Y	Y	Y	Y	Y	Y	N	Y	Y	Y	U	Y	N	N	Y	Y	Y	Y	Y	Y	Y	N	N	Y	Y	N
Maingi, 2018(96)	Y	Y	Y	Y	Y	Y	Y	N	Y	Y	U	U	Y	N	N	Y	Y	Y	Y	Y	N	Y	Y	N	Y	Y	Y
Malta, 2021(160)	Y	Y	Y	Y	Y	Y	Y	N	Y	Y	U	U	Y	U	Y	Y	Y	Y	U	Y	Y	Y	N	N	Y	N	Y
McGiveron, 2015(110)	Y	Y	Y	Y	N	Y	Y	Y	N	Y	U	U	U	N	N	N	Y	Y	Y	Y	Y	Y	N	N	N	Y	N
Merewood, 2019(129)	Y	Y	Y	Y	Y	Y	Y	Y	Y	N	Y	Y	U	U	N	Y	Y	Y	Y	U	Y	Y	N	N	N	Y	N
Merten, 2005(161)	Y	Y	Y	N	Y	Y	Y	N	Y	Y	Y	N	Y	N	N	Y	Y	Y	Y	Y	Y	Y	N	N	Y	Y	N
Metwally, 2022(140)	Y	Y	Y	Y	N	Y	Y	N	N	Y	U	U	Y	U	U	Y	U	Y	Y	Y	N	U	N	N	N	U	N
Milinco, 2020(104)	Y	Y	Y	Y	Y	Y	Y	N	Y	N	U	U	Y	N	Y	Y	Y	Y	Y	Y	Y	Y	Y	Y	Y	Y	Y
Minkovitz, 2001(167)	Y	Y	Y	Y	Y	Y	N	N	Y	N	U	U	Y	U	N	Y	Y	Y	Y	Y	Y	Y	Y	U	Y	Y	N
Mottl-Santiago, 2007(97)	Y	Y	Y	Y	Y	Y	Y	N	Y	Y	U	U	Y	N	U	Y	Y	Y	Y	Y	Y	Y	N	N	Y	Y	Y
Mustila, 2013(79)	Y	Y	Y	Y	Y	Y	Y	Y	Y	Y	U	U	Y	N	N	Y	Y	Y	Y	Y	N	N	N	N	Y	Y	N
Nickel, 2011(183)	Y	Y	N	Y	N	Y	Y	N	N	Y	U	U	Y	N	N	Y	Y	Y	Y	Y	N	Y	N	N	N	U	U
Nommsen-Rivers, 2009(98)	Y	Y	Y	Y	Y	Y	Y	N	Y	Y	U	U	Y	N	Y	Y	Y	Y	U	Y	Y	Y	N	N	Y	Y	Y
Olayiwola, 2013(111)	Y	Y	Y	Y	N	Y	Y	N	Y	Y	U	U	Y	N	N	Y	Y	Y	Y	Y	Y	Y	N	N	N	N	N
Parat, 2019(144)	Y	Y	Y	Y	Y	Y	Y	Y	Y	Y	U	U	Y	Y	N	Y	Y	Y	Y	Y	Y	Y	Y	N	Y	Y	Y
Patel, 2018(127)	Y	Y	Y	Y	Y	Y	N	N	Y	Y	U	U	U	N	N	Y	Y	Y	Y	Y	Y	Y	Y	N	Y	Y	Y
Paul, 2012(139)	Y	Y	Y	N	Y	Y	Y	N	Y	Y	U	U	Y	N	Y	Y	Y	Y	U	Y	Y	Y	Y	Y	Y	Y	Y

Supplementary Data

Author, Year	1. Objective	2. Outcomes	3. Participants	4. Interventions	5. Confounders	6. Findings	7. Variability	8. Adverse Events	9. Lost to Follow Up	10. Probability Values	11. Representative - Recruitment	12. Representative - Enrollment	13. Representative - Setting	14. Blinding - Participants	15. Blinding - Assessors	16. Data Dredging	17. Length of Follow Up	18. Statistical Analysis	19. Intervention Compliance	20. Outcome Measures	21. Recruitment - Same Population	22. Recruitment - Time Period	23. Randomization	24. Randomization Assignment	25. Analysis - Confounding	26. Analysis - Loss to Follow Up	27. Power Analysis
Pugh, 2002(138)	Y	Y	N	Y	Y	Y	N	N	Y	Y	U	U	U	N	N	Y	Y	Y	U	Y	Y	Y	Y	U	N	Y	N
Rasmussen 2011(119)	Y	Y	Y	Y	Y	Y	Y	N	Y	N	U	U	Y	N	Y	Y	Y	N	N	Y	Y	Y	Y	U	Y	Y	N
Redsell, 2017(102)	Y	N	Y	Y	Y	N	N	N	N	N	U	U	Y	N	N	Y	Y	Y	N	U	Y	Y	N	N	U	Y	N
Robertson, 2009(173)	Y	Y	Y	Y	Y	Y	Y	N	Y	Y	U	U	Y	N	N	Y	Y	Y	Y	Y	Y	Y	N	N	U	N	N
Rosen, 2008(174)	Y	Y	Y	Y	P	Y	N	N	Y	Y	U	U	Y	N	N	Y	Y	Y	Y	Y	Y	Y	N	N	U	Y	Y
Rosen-Carole, 2016(168)	Y	Y	Y	Y	Y	Y	Y	N	Y	Y	Y	U	Y	N	N	Y	Y	Y	Y	Y	Y	N	N	N	U	Y	N
Rybak, 2023(143)	Y	Y	Y	Y	Y	Y	Y	Y	Y	Y	Y	Y	Y	U	Y	Y	Y	Y	Y	Y	Y	Y	Y	U	Y	Y	N
Sanders, 2021(153)	Y	Y	Y	Y	Y	Y	Y	N	N	Y	U	U	U	N	N	Y	Y	Y	Y	Y	N	Y	Y	N	Y	Y	Y
Sangalli, 2021(116)	Y	Y	Y	Y	N	Y	Y	N	Y	Y	Y	U	Y	N	Y	Y	Y	Y	U	Y	Y	Y	Y	N	U	Y	Y
Savage, 2022(108)	Y	Y	Y	Y	Y	Y	Y	N	Y	Y	U	U	Y	U	U	Y	Y	Y	U	Y	Y	Y	Y	U	Y	Y	Y
Schroeder, 2015(157)	Y	Y	Y	Y	P	Y	Y	N	N	Y	U	U	Y	U	U	N	Y	Y	Y	Y	Y	U	Y	U	Y	Y	N
Scott, 2015(105)	Y	Y	Y	Y	N	Y	N	N	Y	N	U	U	Y	N	N	Y	Y	U	Y	Y	Y	Y	N	N	N	N	N
Sharma, 2018(85)	Y	Y	Y	Y	Y	Y	Y	N	Y	Y	U	U	U	N	N	Y	Y	Y	Y	Y	Y	Y	N	N	Y	Y	N
Simpson, 2021(86)	Y	Y	Y	Y	Y	Y	Y	Y	Y	Y	Y	N	Y	N	N	Y	Y	Y	Y	Y	N	Y	Y	N	Y	Y	Y
Su, 2007(106)	Y	Y	Y	Y	Y	Y	Y	Y	Y	Y	U	U	Y	N	Y	Y	Y	Y	Y	Y	Y	Y	Y	Y	U	Y	Y
Tanner-Smith, 2013(159)	Y	Y	Y	Y	Y	Y	Y	N	Y	Y	Y	Y	Y	N	N	Y	Y	Y	Y	Y	Y	Y	N	N	Y	Y	N
Tarrant, 2011(175)	Y	Y	Y	Y	Y	Y	Y	N	Y	N	U	U	Y	N	N	Y	Y	Y	N	Y	Y	Y	N	N	Y	Y	N
Tarrant, 2015(162)	Y	Y	Y	Y	Y	Y	Y	N	Y	Y	U	U	Y	N	N	Y	Y	Y	Y	Y	Y	N	N	N	Y	Y	Y
Taveras, 2011(178)	Y	Y	Y	Y	Y	Y	Y	N	N	Y	U	U	Y	N	N	Y	Y	Y	U	Y	N	Y	N	N	Y	Y	N
Taveras, 2021(80)	Y	Y	Y	Y	Y	Y	Y	Y	Y	Y	U	U	Y	N	N	Y	Y	Y	Y	Y	N	Y	N	N	Y	Y	Y
Trotman, 2015(169)	Y	Y	Y	Y	Y	Y	Y	N	Y	Y	U	U	U	N	U	Y	Y	Y	Y	Y	Y	Y	N	N	Y	Y	N
Trudnak, 2011(134)	Y	Y	Y	Y	Y	Y	Y	N	N	Y	Y	N	U	N	N	Y	Y	Y	Y	Y	Y	Y	N	N	Y	Y	N

Supplementary Data

Author, Year	1. Objective	2. Outcomes	3. Participants	4. Interventions	5. Confounders	6. Findings	7. Variability	8. Adverse Events	9. Lost to Follow Up	10. Probability Values	11. Representative - Recruitment	12. Representative - Enrollment	13. Representative - Setting	14. Blinding - Participants	15. Blinding - Assessors	16. Data Dredging	17. Length of Follow Up	18. Statistical Analysis	19. Intervention Compliance	20. Outcome Measures	21. Recruitment - Same Population	22. Recruitment - Time Period	23. Randomization	24. Randomization Assignment	25. Analysis - Confounding	26. Analysis - Loss to Follow Up	27. Power Analysis
Tubay, 2019(154)	Y	Y	Y	Y	Y	Y	N	N	Y	Y	U	U	U	N	N	U	Y	Y	Y	Y	Y	Y	Y	U	Y	Y	Y
Vlasblom, 2020(135)	Y	Y	Y	Y	Y	Y	Y	N	N	Y	U	U	Y	N	N	Y	Y	Y	U	Y	Y	Y	Y	N	Y	Y	Y
Walton, 2015(170)	Y	Y	Y	Y	Y	Y	Y	Y	Y	Y	U	U	N	N	N	Y	Y	Y	Y	Y	Y	N	N	N	U	Y	Y
Wang, 2019(151)	Y	Y	Y	Y	N	Y	Y	N	Y	Y	Y	Y	Y	N	N	Y	Y	Y	Y	Y	N	Y	Y	U	U	Y	Y
Watt, 2015(125)	Y	Y	Y	Y	Y	Y	N	N	N	Y	U	U	U	N	Y	N	Y	Y	Y	Y	Y	Y	N	N	Y	Y	Y
Wilkinson, 2018(112)	Y	Y	Y	Y	Y	Y	Y	N	Y	Y	U	U	Y	N	N	Y	Y	Y	Y	Y	Y	N	N	N	Y	Y	N
Winterburn, 2000(158)	Y	Y	N	Y	N	Y	N	N	Y	Y	U	U	U	N	U	Y	Y	Y	N	Y	Y	Y	Y	N	N	Y	Y
Witt, 2021(107)	Y	Y	Y	Y	Y	Y	N	N	Y	Y	U	U	Y	N	N	Y	Y	Y	Y	Y	Y	N	N	N	Y	Y	N
Witt, 2012(94)	Y	Y	Y	Y	P	Y	N	N	Y	Y	U	U	Y	N	N	Y	Y	Y	Y	Y	Y	N	N	N	N	U	Y
Zielinski, 2014(93)	Y	Y	Y	Y	P	Y	Y	N	Y	Y	U	U	Y	N	N	Y	Y	Y	Y	Y	Y	Y	N	N	Y	Y	U

[a] Y (green color): yes; P (yellow color): partial; U (yellow color): unable to determine; N (orange color): no

Supplementary Data

Supplemental Table 6. Funding sources for included studies

Author, Year	Funding Source(s); Country	Authors' Provided Description of Funding Source(s)
Abrahams, 2009(186)	Carolina Global Breastfeeding Institute with partial support from UNICEF/NYHQ; Bolivia, Brazil, Columbia, Dominican Republic, Egypt, Ghana, Indonesia, Jordan, Kenya, Mali, Niger, Peru, Uganda, Zimbabwe	Carolina Global Breastfeeding Institute at the University of North Carolina at Chapel Hill, with partial support from UNICEF/NYHQ
Alayli, 2020(75)	Federal Joint Committee; Germany	This study is funded by the Innovation Fund of the Federal Joint Committee (G-BA), Module 3: Improving communication with patients and promoting health literacy (Project no. 01NVF17014).
Alberdi, 2018(115)	the National Maternity Hospital in Dublin; Ireland	This study was generously funded by the Nursing and Midwifery Innovation Initiative funding by the National Maternity Hospital in Dublin.
Anderson, 2005(155)	CDC; USA	This study was supported by the Centers for Disease Control and Prevention (Atlanta, Ga) through a subcontract by the Association of Teachers of Preventive Medicine.
Ardalan, 2009(85)	Not reported; USA	Not reported
Beck, 2014(123)	Cincinnati Children's Hospital Medical Center, Procter & Gamble; USA	Dr Beck received funding through the Cincinnati Children's Hospital Medical Center Procter Scholar Award.Funding for the Keeping Infants Nourished and Developing program came from a grant from Procter & Gamble's Live, Learn, and Thrive Initiative.
Bennet, 2022(39) (*Suppl. Ref*)	NIH-NIDDK, partial support by Johns Hopkins Institute for Clinical and Translational Research; USA	This study is funded by a grant from the NIH-NIDDK (1R18DK122416, PI: Wendy L. Bennett). We acknowledge assistance for clinical data coordination and retrieval from the Core for Clinical Research Data Acquisition, supported in part by the Johns Hopkins Institute for Clinical and Translational Research (UL1TR001079).
Bonuck, 2014(118)	NICHD, NIMHD; USA	This work was supported by the National Institute of Child Health and Human Development (NICHD; grant R01 HD04976301A2), and by the National Institute on Minority Health and Health Disparities (NIMHD; grant 1P60 MD 000516-05).
Brodribb, 2013(132)	Queensland Health; Australia	Supported with funding from Queensland Health.
Brownfoot, 2016(99)	The Victorian Managed Insurance Agency; Australia	The Victorian Managed Insurance Agency.
Brumley, 2016(182)	Not reported; USA	Not reported
Budge, 2023(141)	Child Health and Development Institute of Connecticut, Inc; USA	The preparation of this manuscript was financed under an agreement with the Child Health and Development Institute of Connecticut, Inc (CHDI).
Chae, 2017(163)	March of Dimes, Johnson and Johnson Foundations; USA	SungY.Chae MD was the principal project contact for clinical grants by the March of Dimes and the Johnson and Johnson Foundations for the implementation and maintenance of the CenteringPregnancy® program at the JFK Family Medicine Center. Mark H. Chae PhD received a stipend from both of these clinical grants for consultation on the analysis and evaluation of the data included in this manuscript.
Chapman, 2013(121)	USDA, Hartford Hospital, CDC; USA	Funding for the program is provided by the University of Connecticut Family Nutrition Program, through a grant from the US Department of Agriculture Food Stamp Family Nutrition Program, and by Hartford Hospital. Funding for this study was received by Dr Pe´rez-Escamilla from the Centers for Disease Control and Prevention, through a subcontract with the Association of Teachers of Preventive Medicine; Connecticut Family Nutrition Program for Infants, Toddlers, and Children; and the Hartford Hospital Research Foundation.
Chapman, 2004(120)	NIH, partial support Patrick and Catherine Weldon Donaghue Medical Research Foundation , partial support National Center on Minority Health and Health Disparities; USA	Partially supported by the Patrick and Catherine Weldon Donaghue Medical Research Foundation and by the National Center on Minority Health and Health Disparities, National Institutes of Health EXPORT grant P20 MD001765. Funded by the National Institutes of Health (NIH).
Chen, 2017(115)	NICHD, NIH; USA	This study is supported by National Institute of Child Health & Human Development (NICHD), National Institutes of Health (NIH) through a five-year grant (R01HD082311).
Chiurco, 2015(103)	Institute for Maternal and Child Health – IRCCS "Burlo Garofolo"; Italy	This study was approved by the Technical Scientific Committee of the Institute for Maternal and Child Health – IRCCS "Burlo Garofolo", Trieste, Italy, and has been made possible by the research grant RC 14/2006 of the same Institute.
Chwah, 2016(171)	No financial support; Australia	The author(s) received no financial support for the research, authorship, and/or publication of this article.
Clements, 2016(43) (*Suppl. Ref*)	NSW Office of Preventive Health; Australia	The study was internally funded by the NSW Office of Preventive Health.
Corriveau, 2013(95)	Loudoun Pediatric Associates, University of Virginia; USA	This work was supported by Loudoun Pediatric Associates, a pediatric primary care clinic that is part of Loudoun Medical Group located in Leesburg, Virginia, and by the University of Virginia, Charlottesville, Virginia.

Daley, 2015(100)	National Institute for Health Research; United Kingdom	This study was funded by the National Institute for Health Research School for Primary Care Research. AD was supported by a National Institute for Health Research Senior Research Fellowship during the time of this research. SK and KJ are part funded by the NIHR through the Collaborations for Leadership in Applied Health Research and Care for West Midlands (CLAHRC-WM) programme.
de Jersey, 2022(89)	Metro North Hospital and Health Service, Royal Brisbane and Women's Hospital Foundation (RBWH), RBWH Research Advisory Committee, National Health and Medical Research Council, Queensland University of Technology, Advance Queensland Women's Academic Fund, University of Queensland; Australia	Dr de Jersey is supported by a Metro North Hospital and Health Service Clinician Research Fellowship. Project funding was provided by the Royal Brisbane and Women's Hospital (RBWH) Foundation for the New Beginnings Healthy Mothers and Babies Study; RBWH Research Advisory Committee and National Health and Medical Research Council (NHMRC) provided PhD Scholarship funding (SdeJ 1,017,169). TG was supported by a vacation research scholarship from Queensland University of Technology during 2015 that supported part of this work. Funding from the Advance Queensland Women's Academic Fund (Maternity Leave SdeJ) and a University of Queensland New Staff Research Start-up grant was used to support research assistant time for data collection, entry and analysis of the Healthy Pregnancy Healthy Baby study.
Diaz- Rodríguez, 2020(61)	Health Department of the Andalusian Government, FEDER operational program of Andalusia; Spain	This study was supported by public funds the ITI call (integrated territorial investment), developed by the Health Department of the Andalusian Government. The project has been 80% co-financed by funds from the FEDER operational program of Andalusia 2014–2020.
Donkoh, 2013(179)	Not reported; Ghana	Not reported
Döring, 2016(82)	Swedish Research Council, Research and Development Committee, Stockholm County Council, Regional Research Council of the Uppsala and Örebro Health Care Region, Uppsala County Council, Sörmland County Council, Public Health Committee of Stockholm County Council, Vårdal Foundation, AFA Insurance, Foundation of the Swedish Diabetes Society, Karolinska Health Care Sciences Postgraduate School, Karolinska Institutet; Sweden	The PRIMROSE trial has been supported by grants from the following agencies: the Swedish Research Council for Health, Working Life and Welfare (2006-0226 and 2011-0413), the Swedish Research Council (K2006-27X-20069-01-3 and K2012-69X-22058-01-3), the Research and Development Committee, Stockholm County Council (2006-0324), the Regional Research Council of the Uppsala and Örebro Health Care Region (RFR-12404), Uppsala County Council, Sörmland County Council, the Public Health Committee of Stockholm County Council (0803-377), the Vårdal Foundation (B2007-006), AFA Insurance (H- 06: 05/070001), the Foundation of the Swedish Diabetes Society (TMA2006-004), and the Karolinska Health Care Sciences Postgraduate School (2008). The trial has also received faculty funds from the Karolinska Institutet for PhD student grants (2012 and 2013).
Ekström, 2012(156)	University of Sko¨vde, Central Hospital, Swedish Research Council; Sweden	This study was supported by the Skaraborg Institute for Research and Development, School of Life Sciences of the University of Sko¨vde, Sweden; the Primary Care Unit in Skaraborg and the Science Committee, Central Hospital, Sko¨vde, Sweden; and the Board of Research for Health and Caring Sciences, Swedish Research Council, with grant numbers K1999-27P-13085-01A and K2001-27P-13085-036
Ekström, 2014(152)	University of Sko¨vde, Central Hospital; Sweden	This study was supported by the Skaraborg Institute for Research and Development, School of Life Sciences of the University of Sko¨vde, Sweden, and by the Primary Care Unit in Skaraborg and the Science Committee, Central Hospital, Sko¨vde, Sweden
Fan, 2022(13) (*Suppl. Ref*)	Not reported, Not reported	Not reported
Feldman-Winter, 2010(176)	HRSA, CDC; USA	This study was supported in part by Health Resources and Services Administration Maternal and Child Health Bureau grant 4 H04 MD 00009-07-01 CFDA 93.110 and Centers for Disease Control American Medical Colleges Potential Extramural Research Topics grant MM-0993-07/07.
Ferreira, 2018(117)	Ministry of Health, Fundac~ao de Amparo a Pesquisa do Rio Grande do Sul; Brazil	Ministry of Health (No. 577/200) and Fundac~ao de Amparo a Pesquisa do Rio Grande do Sul (PPSUS/ 2006/1537-7).
Flax, 2022(164)	Bill and Melinda Gates Foundation, Irish Aid, Tanoto Foundation, UNICEF, World Bank; Nigeria	This research was funded by the Alive & Thrive initiative, managed by FHI Solutions, and currently funded by the Bill and Melinda Gates Foundation (OPP 1135932, INV-029432), Irish Aid, the Tanoto Foundation, UNICEF, and the World Bank.
French, 2012(146)	NICHD, NIH, Nationwide Children's Hospital; USA	Supported by Eunice Kennedy Shriver National Institute of Child Health and Human Development grant 1 R21HD050944-01 and The Research Institute at Nationwide Children's Hospital. Funded by the National Institutes of Health (NIH)
Gagnon, 1997 (147)	Le Fonds de ta Recherche en Sant~ du Quebec, Medical Research Council, National Health Research and Development Program of Canada; Canada	Funded by a grant from Le Fonds de ta Recherche en Sant~ du Quebec. A.J.G. was supported by the Medical Research Council and the National Health Research and Development Program of Canada.
Garmendia, 2020(145)	Chilean National Fund for Scientific and Technological Development; Chile	Funded by the Chilean National Fund for Scientific and Technological Development, FONDECYT no. 1130277
Gomes, 2019(87)	São Paulo Research Foundation; Brazil	This work was supported by the São Paulo Research Foundation (Grant Numbers: FAPESP 2014/06865-6; FAPESP 2011/18579-0).
Graça, 2011(180)	Not reported; Portugal	Not reported
Grant, 2018(184)	March of Dimes Foundation, Healthcare Georgia Foundation, CMS, Betty and Davis Fitzgerald Foundation; USA	March of Dimes Foundation, Healthcare Georgia Foundation, Centers for Medicare and Medicaid Services and Betty and Davis Fitzgerald Foundation.

Graziano, 2013(47)	Loyola University; USA	Loyola University
Gregory, 2016(109)	Thomas Wilson Sanitarium for the Children of Baltimore City, HRSA, NRSA Training for Careers in Pediatric Primary Care Research; USA	This project was supported by the Thomas Wilson Sanitarium for the Children of Baltimore City. In addition, E.F.G. was supported by the Health Resources and Services Administration (HRSA) of the US Department of Health and Human Services (HHS) under HRSA T32HP10004. NRSA Training for Careers in Pediatric Primary Care Research, $865,647.
Griffin, 2020(65)	Women and Infants Hospital of Rhode Island; USA	Women and Infants Hospital of Rhode Island
Gross, 2016(78)	NIFA-USDA, NIH, NICHD; USA	This work is supported by the National Institute of Food and Agriculture, U.S. Department of Agriculture, under award number 2011-68001-30207 and by the National Institute of Health/National Institute of Child Health and Human Development (NIH/NICHD) through a K23 Mentored Patient-Oriented Research Career Development Award (K23HD081077; PI Gross).
Gross, 2022(165)	HRSA; USA	This project was supported by the Health Resources and Services Administration (HRSA) of the U.S. Department of Health and Human Services (HHS) under grant number R40MC30762, Maternal and Child Health Field-initiated Innovative Research Studies Program.
Grossman, 2009(172)	Theodore Edson Parker Foundation, Drane Foundation, DHHS; USA	Support for this research was provided by the Theodore Edson Parker Foundation, Boston; the Drane Foundation, Boston; and the Office of Women's Health, Department of Health and Human Services, Boston, Massachusetts, USA.
Haby, 2015(124)	Research and Development Board for Gothenburg and Södra Bohuslän; Sweden	The authors wish to express their gratitude to ... the funders of the research, especially the local Research and Development Board for Gothenburg and Södra Bohuslän.
Hale, 2023(128)	Joint Standing Committee Rural Doctors' of BC Chair in Rural Health, Lloyd James Collins grant; Canada	The study was funded by an unrestricted grant from the Joint Standing Committee Rural Doctors' of BC Chair in Rural Health, the BC SUPPORT Unit P2P grant (No. 17473), and a Lloyd James Collins grant.
Hannula, 2014(113)	Finnish Ministry of Health and Welfare; Finland	This study has received funding from the Finnish Ministry of Health and Welfare from Health Promotion Funding.
Hawkins, 2015(166)	NIH, NICHD, National Bureau of Economic Research, NSF-IGERT programme; USA	This work was supported by grants from the National Institutes of Health (NIH) – NICHD R00HD068506 to S.S.H. and T32-AG000186 to the National Bureau of Economic Research and the NSF-IGERT programme, 'Multidisciplinary Program in Inequality & Social Policy' at Harvard University (grant no. 0333403) to A.D.S.
Heberlein, 2016(130)	Mississippi State University, AHRQ, Greenville Health System; USA	This project was supported the Research, Innovation, and Development Grants in Economics (RIDGE) Center for Targeted Studies, Southern Rural Development Center (SRDC), Mississippi State University, Dissertation Grant Program and by the Agency for Healthcare Research and Quality (Grant R36HS021975).This project also received support from the Institute for Advancement of Healthcare, Greenville Health System.
Heining, 2015(24) (*Suppl. Ref*)	University of California Davis; USA	University of California Davis
Hoch, 2023(90)	Metro North; Not reported	Susan de Jersey is supported by a Metro North Clinician Research Fellowship.
Hoffman, 2021(148)	Else Kröner-Fresenius Foundation, Bad Homburg, Technical University of Munich, Competence Centre for Nutrition (KErn) in Bavaria, Bavarian State Ministry of Food, Agriculture and Forestry, Bavarian State Ministry of Health and Care, the AOK Bayern, DEDIPAC consortium; Germany	The study is funded by Else Kröner-Fresenius Foundation, Bad Homburg (Grant number: 5140889), the Else KrönerFresenius Centre for Nutritional Medicine at the Technical University of Munich, the Competence Centre for Nutrition (KErn) in Bavaria, the Bavarian State Ministry of Food, Agriculture and Forestry, the Bavarian State Ministry of Health and Care (Health Initiative "Gesund. Leben.Bayern."), the AOK Bayern, the largest statutory health insurance in Bavaria, as well as the DEDIPAC consortium by the Joint Programming Initiative (JPI) "A Healthy Diet for a Healthy Life".
Holmes, 2012(185)	Pediatric Academic Society; USA	The authors are indebted to the Pediatric Academic Societies' Educational Scholars Program, which provided the stimulus for and oversight of the project
Hopkinson, 2009(114)	American Association of Medical Colleges, DHHS/CDC, USDA/ARS; USA	Support by the American Association of Medical Colleges; United States Department of Health and Human Services/Center for Disease Control and Prevention; and United States Department of Agriculture/Agricultural Research Service. Clinical Trials.gov registration NCT00474422.
Howard, 2018(25) (*Suppl. Ref*)	Not reported; Not reported	Not reported
Ickovics, 2016(150)	NIH, NIMH; USA	This research was supported by the National Institutes of Health (NIH), National Institute of Mental Health (NIMH; grants R01 MH074399 and R01 MH074394, linked R01's to J. R. I. and J. N. T.). V. Earnshaw's work was additionally supported by a postdoctoral fellowship from the NIH, NIMH (grant T32-MH20031).
Kair, 2013(133)	No external funding; USA	No external funding
Kellams, 2014(15) (*Suppl. Ref*)	University of Virgina; USA	The University of Virginia provided financial compensation to Ann Kellams, Jyoti Savla, Tamara Eberly, George Boutsalis, and Natasha Sriraman for their contributions to the project.
Kettrey, 2020(136)	March of Dimes; USA	This study was funded by March of Dimes.

Kim, 2019(126)	Bill & Melinda Gates Foundation, CGIAR Research Program on Agriculture for Nutrition and Health (A4NH); Ethiopia	We acknowledge funding support from the Bill & Melinda Gates Foundation (grant no. OPP1171874), through Alive & Thrive, managed by FHI Solutions LLC. Additional financial support was provided by CGIAR Research Program on Agriculture for Nutrition and Health (A4NH), led by the International Food Policy Research Institute.
Kingsland, 2021(76)	National Health and Medical Research Council Partnership project, Hunter New England Local Health District Clinical Services; Australia	This protocol is for a research project funded by the National Health and Medical Research Council (NHMRC) Partnership Project grant (APP1189756). As part of the NHMRC Partnership Grant funding arrangement, Hunter New England Local Health District Clinical Services Nursing and Midwifery contributes funds and in-kind support to the project.
Kinnuen, 2007(76) (*Suppl. Ref*)	NIH, Ministry of Education, Ministry of Social Affairs and Health; Finland	This study was supported by Doctoral Programs in Public Health (DPPH), Finland, and by grants from the National Institutes of Health in the US (1 U54 CA00100971, 5 RO1 CA89950 to Riitta Luoto and Leena Hilakivi-Clarke) and the Ministry of Education and the Ministry of Social Affairs and Health in Finland.
Kistin, 1994(137)	Not reported; USA	Not reported
Klima, 2009 (181)	Not reported; USA	Not reported
Kola, 2016(16) (*Suppl. Ref*)	Not reported; USA	Not reported
Kramer, 2001(149)	Thrasher Research Fund, National Health Research and Development Program (Health Canada), UNICEF, European Regional Office of WHO; Belarus	PROBIT is supported by grants from the Thrasher Research Fund, the National Health Research and Development Program (Health Canada), UNICEF, and the European Regional Office of WHO.
Laws, 2021(26) (*Suppl. Ref*)	The National Health and Medical Research Council, Victorian Health Promotion Foundation, Victorian Department of Health, Australian Research Council Future Fellowship, Alfred Deakin Postdoctoral Research Fellowship; Australia	This research is funded by The National Health and Medical Research Council (Grant number: GNT1161223), the Victorian Health Promotion Foundation, and the Victorian Department of Health. KH is supported by an Australian Research Council Future Fellowship (FT130100637). VB is supported by an Alfred Deakin Postdoctoral Research Fellowship.
Machuca, 2016(88)	Not reported; USA	Not reported
Maingi, 2018(96)	Pears Foundation, UK; Kenya	This study has been conducted with financial support from the Pears Foundation, UK through The Hebrew University of Jerusalem, International School of Agricultural Sciences, Rehovot, Israel.
Malta, 2021 (160)	Universidade Estadual Paulista Julho de Mesquita Filho, Universidade de São Paulo, Fundação de Alparo à pesquisa do Estado de São Paulo – FAPESP, Conselho Nacional de Desenvolvimento Científico e Tecnológico – CNPq; Brazil	Primary sponsor: Faculdade de Medicina, Universidade Estadual Paulista Julho de Mesquita Filho, Botucatu; Secondary sponsor: Institution: Faculdade de Saúde Pública, Universidade de São Paulo; Supporting source: Institution: Fundação de Alparo à pesquisa do Estado de São Paulo - FAPESP Conselho Nacional de Desenvolvimento Científico e Tecnológico - CNPq
Martin-Iglesias, 2011(27) (*Suppl. Ref*)	Spanish Ministry of Science and Innovation; Spain	This study was funded by the Spanish Ministry of Science and Innovation via the Instituto de Salud Carlos III (PI08/90680).
Matvienko-Sikar, 2019(67)	Health Research Board; Ireland	This research is supported by a Health Research Board Interdisciplinary Capacity Enhancement Award (HRB ICE 2015-1026).
McGiveron, 2015(110)	Public Health Lincolnshire County Council; United Kingdom	The Bumps and Beyond intervention was a clinical service funded by Public Health Lincolnshire County Council.
Merewood, 2019(129)	W.K. Kellogg Foundation; USA	Funded by a grant from the W.K. Kellogg Foundation (P3030006).
Merten, 2005(161)	Swiss Federal Office for Public Health; Switzerland	Swiss Federal Office for Public Health for financial support of the study
Metwally, 2022(140)	The Science, Technology & Innovation Funding Authority, The Egyptian Knowledge Bank; Egypt	Open access funding provided by The Science, Technology & Innovation Funding Authority (STDF) in cooperation with The Egyptian Knowledge Bank (EKB). We did not receive any grants for this work.
Middleton, 2019(110)	NHMRC; Australia	NHMRC funded research
Milinco, 2020(104)	Institute for Maternal and Child Health - IRCCS “Burlo Garofolo”; Italy	The study was supported by the Institute for Maternal and Child Health - IRCCS “Burlo Garofolo”, Trieste, Italy (RC 29/17).
Minkovitz, 2001(167)	Commonwealth Fund and local funders; USA	The HS (http://www.healthysteps.org) is a program of the Commonwealth Fund, New York, local funders, and health care providers across the nation. It is cosponsored by the American Academy of Pediatrics, Elk Grove, Ill. Funding for the Healthy Steps National Evaluation is being provided by the Commonwealth Fund and local funders.
Mottl-Santiago, 2007(97)	Unfunded; USA	This study was unfunded.
Mustila, 2013(79)	Foundation of Pediatric Research, Medical Research Fund of Vaasa Hospital District, Pediatric Research Centre; Finland	Foundation of Pediatric Research (Finland), the Medical Research Fund of Vaasa Hospital District and the Pediatric Research Centre (Tampere, Finland).

Nabulsi, 2014(58) (*Suppl. Ref*)	Not reported; Lebanon	Not reported
Nagle, 2011(59) (*Suppl. Ref*)	Deakin University; Australia	Funding has been obtained from the Central Research Grant Scheme, Deakin University.
Nickel, 2011(183)	Carolina Global Breastfeeding Institute Endowment, Duke Endowment, Kate B. Reynolds Charitable Trust, FDA, AHRQ; USA	This work was supported by the Carolina Global Breastfeeding Institute Endowment, the Duke Endowment, the Kate B. Reynolds Charitable Trust, the Food and Drug Administration, and the Agency for Healthcare Research and Quality.
Nommsen-Rivers, 2009(98)	Sutter Medical Center; USA	Funded by Sutter Medical Center, Sacramento.
O'Reilly, 2021(60) (*Suppl. Ref*)	Part funded by HSE Nursing and Midwifery Planning and Development Unit Dublin South, University College Dublin; Ireland	The project is part funded by the HSE Nursing and Midwifery Planning and Development Unit Dublin South, Kildare and Wicklow and sponsored by University College Dublin (UCD)
Olayiwola, 2013(111)	HRSA; USA	The Living Smart, Living Fit program was funded by the HRSA Maternal-Child Health Bureau, Healthy Behaviors in Women Grant, Grant Number H59MC09956.
Palacios, 2021(29) (*Suppl. Ref*)	Florida International University; USA	Florida International University
Parat, 2019(144)	French Ministry of Health; France	The sponsor was Assistance Publique – Hôpitaux de Paris (Clinical Research and Development Department) and this project was funded by a grant from Programme Hospitalier de Recherche Clinique (PHRC 2007, French Ministry of Health).
Patel, 2018(127)	World Bank- SARDM, The Bill and Melinda Gates Foundation; India	This trial was initially funded by World Bank- SARDM (South Asia Region Development Marketplace Grant ID - 806410) and subsequently by Alive and Thrive initiative, The Bill and Melinda Gates Foundation (Grant ID – 09-000076-AT10-4LMR).
Paul, 2012(139)	HRSA/DHHS, Children's Miracle Network; USA	This project was supported by grant R40 MC 06630 from the Maternal Child Health Bureau (Title V, Social Security Act), Health Resources and Services Administration, Department of Health and Human Services. Additional support was provided by the Children's Miracle Network.
Paul, 2005(30) (*Suppl. Ref*)	Not reported; USA	Not reported
Pugh, 2002(138)	NINR; USA	The National Institute of Nursing Research, Bethesda, Maryland, funded this study (R55 NR04958).
Rasmussen, 2011(119)	USDA, Medela, Inc.; USA	This research was supported in part by USDA/Hatch grant NYC-399430. The electric pumps were donated by Medela,Inc.
Redsell, 2017(102)	Medical Research Council; United Kingdom	This work was supported by the Medical Research Council – Public Health Intervention Development Scheme, grant number PHIND 01/14-15.
Robertson, 2009(173)	Sigma Theta Tau Alpha Epsilon Chapter; USA	Financial support for this project was received from Sigma Theta Tau Alpha Epsilon Chapter
Rosen, 2008(174)	Not reported; USA	Not reported
Rosen-Carole, 2016(168)	No financial support; USA	The authors received no financial support for the research, authorship, and/or publication of this article.
Rybak, 2023(143)	NIDDK; USA	This work was supported by funding from the National Institute of Diabetes and Digestive Kidney Diseases (NIDDK T32 DK063929).
Sabo, 2018(32) (*Suppl. Ref*)	Not reported; USA	Not reported
Sanders, 2021(153)	NICHD, CDC, NIH-NCATS, Robert Wood Johnson Foundation, HRSA, KiDS of NYU Langone Foundation, NIH, PCORI; USA	FUNDING: Supported by the Eunice Kennedy Shriver Institute for Child Health and Development, National Institute of Child Health and Human Development (grant R01 HD049794), with supplemental funding from Centers for Disease Control and Prevention and Office of Behavioral and Social Sciences Research (grant R01HD059794-04S1, R01HD059794-04S2). Parts of the study were supported the National Institutes of Health's National Center for Advancing Translational Sciences through its Clinical and Translational Science Awards Program (grants 1UL1RR029893, UL1TR000445, and UL1RR025747). During the time the study was conducted, Dr Yin was supported by a grant under the Robert Wood Johnson Foundation Physician Faculty Scholars Program and Health Resources and Services Administration (12-191-1077-Academic Administrative Units in Primary Care) and by funding from the KiDS of NYU Langone Foundation. During the beginning of the study, Dr Perrin was supported by a training grant from National Institute of Child Health and Human Development (grant K23 HD051817). Funded by the National Institutes of Health (NIH)<br>This work was supported by the Patient Centered Outcomes Research Institute (PCORI) [contract number AD-2018C1-11238].

Sangalli, 2021(116)	Fundação de Amparo à Pesquisa do Estado do Rio Grande do Sul, Coordenação de Aperfeiçoamento de Pessoal de Nível Superior, Conselho Nacional de Desenvolvimento Científico e Tecnológico, Ministério da Saúde, NIH; Brazil	Fundação de Amparo à Pesquisa do Estado do Rio Grande do Sul, Grant/Award Number: PPSUS/2006/1537- 7; Coordenação de Aperfeiçoamento de Pessoal de Nível Superior, Grant/Award Number: 88881.132790/2016- 01; Conselho Nacional de Desenvolvimento Científico e Tecnológico, Grant/Award Number: 14/2013- 47731/2013- 8; Ministério da Saúde, Grant/Award Number: 577/200; US National Institutes of Health, Grant/Award Number: R01AG066887
Savage, 2022(108)	HRSA/DHHS; USA	This project is supported by the Health Resources and Services Administration (HRSA) of the U.S. Department of Health and Human Services (HHS) under grant number R40MC28317, Maternal and Child Health Field-initiated Innovative Research Studies Program.
Savitri, 2016(106)	Nutricia Indonesia Fund, UMC Utrecht Global Health Support Program Faculty of Medicine University of Indonesia/Cipto Mangunkusumo General Hospital, Budi Kemuliaan Hospital, University Medical Center Utrecht; Indonesia	BRAVO was made possible by an unrestricted grant from Nutricia Indonesia Fund, by several UMC Utrecht Global Health Support Program grants for PhDs, and by in-kind provisions by the center for Clinical Epidemiology and Evidence-Based Medicine and Department of Child Health at Faculty of Medicine University of Indonesia/Cipto Mangunkusumo General Hospital, Jakarta, Indonesia, by Budi Kemuliaan Hospital, Jakarta, Indonesia, and by the Julius Center for Health Sciences and Primary Care, University Medical Center Utrecht, Utrecht, the Netherlands
Schroeder, 2015(157)	Dannon Institute; USA	This study was funded by a competitive grant from the Dannon Institute (USA).
Scott, 2015(105)	Not reported; USA	Not reported
Sharma, 2018(85)	1115 Medicaid Transformation Waiver program; USA	1115 Medicaid Transformation Waiver program called the Delivery System Reform Incentive Payment Program (DSRIP)
Simpson, 2021(86)	National Prevention Research Initiative, Slimming World, Medical Research Council Chief Scientist Office of the Scottish Government Health and Social Care Directorates; United Kingdom	The study was funded by the National Prevention Research Initiative (NPRI). Slimming World provided some of the intervention delivery costs which included staff time at Slimming World Head Office and materials for participants. SS was supported by the Medical Research Council and the Chief Scientist Office of the Scottish Government Health and Social Care Directorates (MC-PC-13027, MC_UU_12017_14 and SPHSU14).
Su, 2007(106)	National Healthcare Group; Singapore	National Healthcare Group (grant No NHG-RPR 03002)
Tanner-Smith, 2013(159)	Tennessee Governor's Office of Children's Care Coordination, Tennessee Department of Health, NICHD/NIH, NCATS; USA	This work was supported by contract #19199-GR1030830 from the Tennessee Governor's Office of Children's Care Coordination (GOCCC) and the Tennessee Department of Health (DOH). S.B. Gesell was supported by NICHD/NIH grant K23HD064700. The secondary data analysis project described was supported by CTSA award No. UL1TR000445 from the National Center for Advancing Translational Sciences.
Tarrant, 2011(175)	Not reported; Hong Kong	Not reported
Tarrant, 2015(162)	Food and Health Bureau, Government of the Hong Kong Special Administration Region, University of Hong Kong; Hong Kong	This study was supported by the Health and Medical Research Fund (grant number 05060721) from the Food and Health Bureau, Government of the Hong Kong Special Administration Region; the University of Hong Kong (grant #10207306); the University of Hong Kong's Strategic Research Theme of Public Health; and the School of Nursing of the University of Hong Kong.
Taveras, 2011(178)	Harvard Medical School, Harvard Pilgrim Health Care, Robert Wood Johnson Foundation; USA	Harvard Medical School, Harvard Pilgrim Health Care, and by the Physician Faculty Scholars Program of the Robert Wood Johnson Foundation.
Taveras, 2021(80)	Boston Foundation, NIDDK-NIH, Massachusetts General Hospital; USA	The Boston Foundation (G2015-0007), the National Institute of Diabetes and Digestive and Kidney Diseases of the National Institutes of Health (K24DK105989 and K01DK114383), and Massachusetts General Hospital.
Trotman, 2015(169)	March of Dimes; USA	Dr. Renuka Darolia has received a grant from the March of Dimes in support of performing Centering Pregnancy groups at MedStar Washington Hospital Center, but has received no financial support from this organization in support of this research project.
Trudnak, 2011(134)	Not reported; USA	Not reported
Tubay, 2019(154)	60th Medical Group Clinical Investigations Facility, March of Dimes; USA	This study was funded by the 60th Medical Group Clinical Investigations Facility, and was supported in part by a generous grant from the March of Dimes.
Virtanen, 2010(36) (*Suppl. Ref*)	Not reported; Finland	Not reported
Vlasblom, 2020(135)	Netherlands Organization for Health Research and Development; Netherlands	A grant from ZonMW, the Netherlands Organization for Health Research and Development (grant number 50-50110-96-491).
Walton, 2015 (170)	Not reported; USA	Not reported
Wang, 2019 (151)	National Natural Science Foundation; China	This study was supported by a grant from the National Natural Science Foundation of China (Nos. 81172686, 81302446, and 81703249).
Watt, 2015(125)	Not reported; USA	Not reported

Wilkinson, 2018(112)	Queensland Government Department of Health, Queensland Health, Queensland Government; Australia	SAW was supported by a Queensland Government Department of Health—Health Research Fellowship. The authors wish to acknowledge Queensland Health—Health Research Fellowship (2015–2020) funding; Maternity leave RA sup- port from Queensland Government—Advance Queensland grant
Winterburn, 2000(158)	Northern General Hospital; United Kingdom	The study was supported by a grant from the Northern General Hospital Trust Research Committee.
Witt, 2021(107)	Partially funded by CDC; USA	This article is based in part on work from a project for breastfeeding support and training partially funded by the Centers for Disease Control and Prevention; National Center for Chronic Disease Prevention and Health Promotion; Division of Nutrition, Physical Activity, and Obesity; and Racial and Ethnic Approaches to Community Health (REACH) Program, Grant Number: 5 NU58DP006586-03-00
Witt, 2012(94)	DHHS/HRSA; USA	This project was completed in part by the Culture of Inquiry Fellowship, funded by a Title VII grant from the Health Resources and Services Administration (DHHS/HRSA D54HP05444-01-00, 2008).
Zielinski, 2014(93)	Not reported; USA	Not reported

Supplementary Data

Supplemental References

1. Holmes AV, Yerdon A, Kramer S, Wells M, Howard CR. Physician breastfeeding education leads to practice changes, but not improved clinical outcomes. Breastfeed Med. 2009;4(4):235-6. doi: 10.1089/bfm.2009.7000.
2. Nct. A feasibility study of integrating maternal nutrition interventions into antenatal care services in Ethiopia. https://clinicaltrialsgov/show/NCT04125368. 2019.
3. Savage JS, Kling SMR, Cook A, Hess L, Lutcher S, Marini M, Mowery J, Hayward S, Hassink S, Hosterman J, et al. A patient-centered, coordinated care approach delivered by community and pediatric primary care providers to promote responsive parenting: pragmatic randomized clinical trial rationale and protocol. BMC Pediatr. 2018;18(1) (no pagination). doi: 10.1186/s12887-018-1263-z.
4. Nct. Coordination of care between pediatricians and Women Infants & Children nutritionists. https://clinicaltrialsgov/show/NCT03482908. 2018.
5. Rinehart DN. Evaluating the impact of HEAL pregnancy intervention on breastfeeding initiation and duration [master's thesis]. [Houston]: University of Texas; 2019. 37 p.
6. John E, Cassidy DM, Playle R, Jewell K, Cohen D, Duncan D, Newcombe RG, Busse M, Owen-Jones E, Williams N, et al. Healthy eating and lifestyle in pregnancy (HELP): a protocol for a cluster randomised trial to evaluate the effectiveness of a weight management intervention in pregnancy. BMC Public Health. 2014;14:439. doi: 10.1186/1471-2458-14-439.
7. Jewell K, Avery A, Barber J, Simpson S. The healthy eating and lifestyle in pregnancy (HELP) feasibility study. Br J Midwifery. 2014;22(10):727-36.
8. Gallagher D. The Healthy Eating and Lifestyle in Pregnancy cluster randomised controlled trial: A 24 months postpartum follow-up study : An evaluation of the effect of a weight management intervention for maternal obesity, on maternal and child outcomes at 24 months following birth. [dissertation]. [Cardiff]: Cardiff University; 2018. 370 p.
9. Simione M, Moreno-Galarraga L, Perkins M, Price SN, Luo M, Kotelchuck M, Blake-Lamb TL, Taveras EM. Effects of the First 1000 Days Program, a systems-change intervention, on obesity risk factors during pregnancy. BMC Pregnancy and Childbirth. 2021;21(1). doi: 10.1186/s12884-021-04210-9.
10. Blake-Lamb T, Boudreau AA, Matathia S, Perkins ME, Roche B, Cheng ER, Kotelchuck M, Shtasel D, Taveras EM. Association of the First 1,000 Days Systems-Change Intervention on Maternal Gestational Weight Gain. Obstet Gynecol. 2020;135(5):1047-57. doi: 10.1097/aog.0000000000003752.
11. Blake-Lamb T, Boudreau AA, Matathia S, Tiburcio E, Perkins ME, Roche B, Kotelchuck M, Shtasel D, Price SN, Taveras EM. Strengthening integration of clinical and public health systems to prevent maternal-child obesity in the First 1,000 Days: A Collective Impact approach. Contemp Clin Trials. 2018;65:46-52. doi: 10.1016/j.cct.2017.12.001.
12. Nct. The First 1,000 Days Program: maternal-child obesity prevention in early life. https://clinicaltrialsgov/show/NCT03191591. 2017.
13. Fan WQ, Chan C, Paterson S, Foster K, Morrow M, Bourne D, Ashworth J. Weekly lactation consultant led telephone calls in the first month postpartum improves breast feeding rates over standard care - A randomized controlled trial. Journal of Paediatr Child Health. 2022;58(SUPPL 2):11. doi: 10.1111/jpc.15945.
14. Chapman DJ, Wetzel K, Bermudez-Millan A, Young S, Damio G, Pérez-Escamilla R. Effects of breastfeeding peer counseling for obese women on infant health outcomes. FASEB J. 2010;24.
15. Kellams A, Savla J, Akers LH, Eberly TW, Boutsalis G, Sriraman NK. The impact of a physician performance improvement project for maintenance of certification on an important clinical outcome: Exclusive breastfeeding. J Contin Educ Health Prof. 2014;34:S30-S1. doi: 10.1002/chp.21233.
16. Kola B. Small changes making big impact. Cogent Med. 2016;3(1). doi: 10.1080/2331205X.2016.1265203.
17. Burnham L, Knapp R, Bugg K, Nickel N, Beliveau P, Feldman-Winter L, Merewood A. Mississippi CHAMPS: Decreasing racial inequities in breastfeeding. Pediatrics. 2022;149(2). doi: 10.1542/peds.2020-030502.

18. Bugg K, Edwards R, Burnham L, Serano A, Merewood A. The champs initiative: Integrating communities for reducing breastfeeding disparities. J Womens Health (Larchmt). 2017;26(4):A9-A10. doi: 10.1089/jwh.2017.29011.abstracts.
19. Nct. Best Beginnings for Babies Birth Sister program evaluation. https://clinicaltrialsgov/show/NCT02550730. 2015.
20. Tarrant M, Lok KYW, Fong DYT, Wu KM, Lee ILY, Sham A, Lam C, Bai DL, Wong KL, Wong EMY, et al. Effect on Baby-Friendly Hospital steps when hospitals implement a policy to pay for infant formula. J Hum Lact. 2015;32:238-49. doi: 10.1177/0890334415599399.
21. Doring N, Ghaderi A, Bohman B, Heitmann B, Larsson C, Berglind D, et al. Primary prevention of childhood obesity within child health services: the PRIMROSE cluster-RCT. Obesity facts Conference: european obesity summit (EOS): 1st joint congress of EASO and IFSO-EC Gothenburg sweden Conference start: 20160601 Conference end: 20160604 Conference publication: (varpagings). 2016;9:35. doi: 10.1159/000446744. PubMed PMID: CN-01266581.
22. Döring N, Hansson LM, Andersson ES, Bohman B, Westin M, Magnusson M, Hansson L, Sundblom E, Magnusson M, Blennow M, et al. Primary prevention of childhood obesity through counselling sessions at Swedish child health centres: Design, methods and baseline sample characteristics of the PRIMROSE cluster-randomised trial. BMC Public Health. 2014;14:335. doi: 10.1186/1471-2458-14-335.
23. Groner JA, Skybo T, Murray-Johnson L, Schwirian P, Eneli I, Sternstein A, Klein E, French G. Anticipatory guidance for prevention of childhood obesity: Design of the MOMS Project. Clin Pediatr (Phila). 2009;48(5):483-92. doi: 10.1177/0009922809331799.
24. Nct. Supporting Baby Behavior through pediatric offices. https://clinicaltrialsgov/show/NCT02438878. 2015.
25. Howard KA, Tucker J, DeFrang R, Orth J, Wakefield S. Primary care obesity prevention in 0-2 year olds through parent nutritional counseling: Evaluation of child behaviors and parent feeding styles. Pediatrics. 2018;141(1). doi: 10.1542/peds.141.1-MeetingAbstract.591.
26. Laws R, Love P, Hesketh KD, Koorts H, Denney-Wilson E, Moodie M, Brown V, Ong KL, Browne J, Marshall S, et al. Protocol for an effectiveness-implementation hybrid trial to evaluate scale up of an evidence-based intervention addressing lifestyle behaviours from the start of life: INFANT. Front Endocrinol (Lausanne). 2021;12:717468. doi: 10.3389/fendo.2021.717468.
27. Martín-Iglesias S, del-Cura-González I, Sanz-Cuesta T, Arana-Cañedo_Argüelles C, Rumayor-Zarzuelo M, la Riva M, Lloret-Sáez_Bravo AM, Férnandez-Arroyo RM, Aréjula-Torres JL, Aguado-Arroyo Ó, et al. Effectiveness of an implementation strategy for a breastfeeding guideline in primary care: Cluster randomised trial. BMC Fam Pract. 2011;12:144-. doi: 10.1186/1471-2296-12-144.
28. Matvienko Sikar K, Toomey E, Queally M, Flannery C, O'Neill K, Doherty E, Harrington J, Hayes C, Heary C, Heffernan T, et al. Developing, implementing and evaluating the choosing healthy eating for infant health (CHErIsH) intervention to prevent childhood obesity in primary care: A feasibility study. Obesity Facts. 2019;12:109-10. doi: 10.1159/000489691.
29. Nct. Pilot testing of Baby Feed: A web application for health professionals and parents to improve infant eiets. https://clinicaltrialsgov/show/NCT05062824. 2021.
30. Nct. Healthy sleeping and feeding during infancy. https://clinicaltrialsgov/show/NCT00125580. 2005.
31. Nct. Teaching Tealthy Responsive parenting during Infancy to promote Vital growth and dEvelopment (THRIVE) study. https://clinicaltrialsgov/show/NCT04989738. 2021.
32. Sabo AC, Patel SM. Enhancing breastfeeding rates using cross-sectional data, surveys, and a population-based need intervention: A quality improvement project. Acad Pediatr. 2018;18(5):e43-e4.
33. Sanders LM, Perrin EM, Yin HS, Bronaugh A, Rothman RL. "Greenlight study": A controlled trial of low-literacy, early childhood obesity prevention. Pediatrics. 2014;133(6):e1724-37. doi: 10.1542/peds.2013-3867.
34. Nct. Greenlight Plus Study: Approaches to early childhood obesity prevention. https://clinicaltrialsgov/show/NCT04042467. 2019.
35. Schroeder N, Rushovich B, Bartlett E, Gittelsohn J, Caballero B. Early obesity prevention: A randomized trial of a practice-based intervention in 0-24 months infants. FASEB J. 2012;26.

36. Nct. Lifestyle intervention for toddlers pilot study. https://clinicaltrialsgov/show/NCT01204489. 2010.
37. Eline V, L'Hoir M, Van Grieken A, Hein R, Magda BB. Effectiveness of a primary prevention program of overweight in 0-3 year old children, the BBOFT+ study; a cluster randomized trial. J Pediatr Gastroenterol Nutr. 2016;62:725. doi: 10.1097/01.mpg.0000484500.48517.e7.
38. O'Sullivan EJ, Alberdi G, Scully H, Kelly NM, Kincaid R, Murtagh R, Murray S, McGuinness D, Clive A, Brosnan M, et al. Antenatal breastfeeding self-efficacy and breastfeeding outcomes among mothers participating in a feasibility breastfeeding-support intervention. Ir J Med Sci. 2018;188:569-78. doi: 10.1007/s11845-018-1884-0.
39. Bennett WL, Coughlin JW, Henderson J, Martin S, Yazdy GM, Drabo EF, Showell NN, McKinney C, Martin L, Dalcin A, et al. Healthy for Two/Healthy for You: Design and methods for a pragmatic randomized clinical trial to limit gestational weight gain and prevent obesity in the prenatal care setting. Contemp Clin Trials. 2022;113:106647. doi: 10.1016/j.cct.2021.106647.
40. Nct. Boosting breastfeeding in low-income, multi-ethnic women: A primary care based RCT. https://clinicaltrialsgov/show/NCT00619632. 2008.
41. Nct. Provider approaches to improved rates of infant nutrition and growth study. https://clinicaltrialsgov/show/NCT00643253. 2008.
42. Brownfoot FC, Davey M, Kornman L. Weighing in pregnancy study (WIP): A randomised controlled trial of the effect of routine weighing to reduce excessive antenatal weight gain. BJOG. 2015;122:260. doi: 10.1111/14710528.13383.
43. Clements V, Kit L, Khanal S, Raymond J, Maxwell M, Rissel C, Leung K. Pragmatic cluster randomised trial of a free telephone-based health coaching program to support women in managing weight gain during pregnancy: The Get Healthy in Pregnancy Trial. BMC Health Serv Res. 2016;16:1-9. doi: 10.1186/s12913-016-1704-z.
44. Daley A, Jolly K, Lewis A, Clifford S, Kenyon S, Roalfe AK, Jebb S, Aveyard P. The feasibility and acceptability of regular weighing of pregnant women by community midwives to prevent excessive weight gain: RCT. Pregnancy Hypertens. 2014;4(3):233-4. doi: 10.1016/j.preghy.2014.03.014.
45. Garmendia ML, Corvalan C, Araya M, Casanello P, Kusanovic JP, Uauy R. Effectiveness of a normative nutrition intervention (diet, physical activity and breastfeeding) on maternal nutrition and offspring growth: The Chilean maternal and infant nutrition cohort study (CHiMINCs). BMC Pregnancy Childbirth. 2015;15:175. doi: 10.1186/s12884-015-0605-1.
46. Nct. Diet, physical activity and breastfeeding intervention on maternal nutrition, offspring growth and development. https://clinicaltrialsgov/show/NCT01916603. 2013.
47. Nct. Gestational weight gain and the electronic medical record. https://clinicaltrialsgov/show/NCT01987141. 2013.
48. Kunath J, Günther J, Rauh K, Hoffmann J, Stecher L, Rosenfeld E, Kick L, Ulm K, Hauner H. Effects of a lifestyle intervention during pregnancy to prevent excessive gestational weight gain in routine care - the cluster-randomised GeliS trial. BMC Med. 2019;17(1):5. Epub 20190114. doi: 10.1186/s12916-018-1235-z.
49. Hoffmann J, Günther J, Stecher L, Spies M, Meyer D, Kunath J, Raab R, Rauh K, Hauner H. Effects of a lifestyle intervention in routine care on short-and long-term maternal weight retention and breastfeeding behavior—12 months follow-up of the cluster-randomized GeliS trial. J Clin Med. 2019;8(6). doi: 10.3390/jcm8060876.
50. Rauh K, Kunath J, Rosenfeld E, Kick L, Ulm K, Hauner H. Healthy living in pregnancy: A cluster-randomized controlled trial to prevent excessive gestational weight gain - rationale and design of the GeliS study. BMC Pregnancy Childbirth. 2014;14:119. doi: 10.1186/1471-2393-14-119.
51. Hoffmann J, Günther J, Stecher L, Spies M, Meyer D, Raab R, Geyer K, Kunath J, Rauh K, Hauner H. Does an antenatal lifestyle intervention in routine care improve maternal and infant health outcomes in the first year postpartum-12 months follow-up of the cluster-randomised GeliS trial. Obes Rev. 2020;21(SUPPL 1). doi: 10.1111/obr.13118.
52. Nct. Healthy living in pregnancy. https://clinicaltrialsgov/show/NCT01958307. 2013.

Supplementary Data

53. Rauh K, Gabriel E, Kerschbaum E, Schuster T, von Kries R, Amann-Gassner U, Hauner H. Safety and efficacy of a lifestyle intervention for pregnant women to prevent excessive maternal weight gain: A cluster-randomized controlled trial. BMC Pregnancy Childbirth. 2013;13:151. doi: 10.1186/1471-2393-13-151.

54. Magriples U, Boynton MH, Kershaw T, Lewis JB, Rising SS, Tobin JN, Epel ES, Ickovics JR. The impact of group prenatal care on pregnancy and postpartum weight trajectories. Am J Obstet Gynecol. 2015;213:6-8.

55. mkg RBR. Lifestyle intervention in pregnant women. https://trialsearchwhoint/Trial2aspx?TrialID=RBR-4mkg73. 2017.

56. Mustila T, Keskinen P, Luoto R. Behavioral counseling to prevent childhood obesity - study protocol of a pragmatic trial in maternity and child health care. BMC Pediatr. 2012;12. doi: 10.1186/1471-2431-12-93.

57. Nct. Vaasa childhood obesity primary prevention study. https://clinicaltrialsgov/show/NCT00970710. 2009.

58. Nabulsi M, Hamadeh H, Tamim H, Kabakian T, Charafeddine, Yehya NA, Sinno D, Sidani S. A complex breastfeeding promotion and support intervention in a developing country: Study protocol for a randomized clinical trial. BMC Public Health. 2014;14:36-. doi: 10.1186/1471-2458-14-36.

59. Nagle C, Skouteris H, Hotchin A, Bruce L, Patterson D, Teale G. Continuity of midwifery care and gestational weight gain in obese women: a randomised controlled trial. BMC Public Health. 2011;11:174. doi: 10.1186/1471-2458-11-174.

60. O'Reilly SL, O'Brien EC, McGuinness D, Mehegan J, Coughlan B, O'Brien D, Szafranska M, Callanan S, Hughes S, Conway MC, et al. Latch On: A protocol for a multi-centre, randomised controlled trial of perinatal support to improve breastfeeding outcomes in women with a raised BMI. Contemporary Clinical Trials Communications. 2021;22. doi: 10.1016/j.conctc.2021.100767.

61. Obrien E, Oreilly S, Sheehy L, Ohagan L, Yelverton C, McGuinness D, Coughlan B, Obrien D, Murtagh R, Corbett M, et al. LatchOn: A protocol for a multi-centre, randomised controlled trial of perinatal support to improve breastfeeding outcomes in women with overweight and obesity. Obesity Facts. 2019;12:105-6. doi: 10.1159/000489691.

62. O'Brien E, O'Reilly S, Sheehy L, O'Hagan L, McGuinness D, Coughlan B, O'Brien D, Murtagh R, Corbett M, Walsh M, et al. Latchon: a multi-centre, randomised controlled trial of perinatal support to improve Breastfeeding outcomes in women with overweight and obesity. Arch Dis Child. 2019;104:A359-. doi: 10.1136/archdischild-2019-epa.850.

63. Isrctn. LatchOn: a breastfeeding support study. https://trialsearchwhoint/Trial2aspx?TrialID=ISRCTN14819650. 2019.

64. Tanner-Smith EE, Steinka-Fry KT, Gesell SB. Comparative effectiveness of group and individual prenatal care on gestational weight gain. Matern Child Health J. 2013;18:1711-20. doi: 10.1007/s10995-013-1413-8.

65. Trudnak TE, Arboleda E, Kirby RS, Perrin K. Outcomes of Latina women in CenteringPregnancy group prenatal care compared with individual prenatal care. J Midwifery Womens Health. 2013;58:396-403. doi: 10.1111/jmwh.12000.

66. Wilkinson SA, Walker A, Tolcher D. Re-evaluation of women's nutritional needs, knowledge and behaviours in a tertiary maternity service: are we meeting women's needs yet? Nutrition & Dietetics. 2013;70(3):181-7. doi: 10.1111/1747-0080.12010.

67. Gogel L, Zielinski R, Deibel M, Kothari C. Improving maternal and infant health through CenteringPregnancy: Results of a 2-year retrospective chart review using a matched comparison design. J Midwifery Womens Health. 2013;58(5):584-5. doi: 10.1111/jmwh.12118.

68. Messito MJ, Katzow MW, Mendelsohn AL, Gross RS. Starting Early program impacts on feeding at infant 10 months age: A randomized controlled trial. Child Obes. 2020;16(S1):S4-s13. doi: 10.1089/chi.2019.0236.

69. Messito MJ, Mendelsohn AL, Katzow MW, Scott MA, Vandyousefi S, Gross RS. Prenatal and pediatric primary care–based child obesity prevention program: A randomized trial. Pediatrics. 2020;146(4). doi: 10.1542/peds.2020-022103.

Supplementary Data

70. Messito MJ, Gross R, Katzow M, Mendelsohn A. NP20 Starting Early: Expansion of a primary care-based early child obesity prevention program. J Nutr Educ Behav. 2019;51(7):S19-S. doi: 10.1016/j.jneb.2019.05.344.
71. Messito MJ, Mendelsohn A, Scheinmann R, Gross R. Starting Early/Empezando Temprano: randomized control trial to test the effectiveness of a child obesity prevention program. J Nutr Educ Behav. 2018;50(7):S120-S1.
72. Nct. Starting Early obesity prevention program. https://clinicaltrialsgov/show/NCT01541761. 2012.
73. Kinnunen TI, Pasanen M, Aittasalo M, Fogelholm M, Weiderpass E, Luoto R. Reducing postpartum weight retention - A pilot trial in primary health care. Nutrition Journal. 2007;6. doi: 10.1186/1475-2891-6-21.
74. Mustila T, Raitanen J, Keskinen P, Saari A, Luoto R. Lifestyle counseling during pregnancy and offspring weight development until four years of age: Follow-up study of a controlled trial. J Negat Results Biomed. 2012;11:11. doi: 10.1186/1477-5751-11-11.
75. Mustila T, Raitanen J, Keskinen P, Saari A, Luoto R. Lifestyle counselling targeting infant's mother during the child's first year and offspring weight development until 4 years of age: A follow-up study of a cluster RCT. BMJ Open. 2012;2(1):e000624. doi: 10.1136/bmjopen-2011-000624.
76. Kinnunen TI, Pasanen M, Aittasalo M, Fogelholm M, Hilakivi-Clarke L, Weiderpass E, Luoto R. Preventing excessive weight gain during pregnancy - A controlled trial in primary health care. Eur J Clin Nutr. 2007;61(7):884-91. doi: 10.1038/sj.ejcn.1602602.
